# Phylogenomics analysis of velvet regulators in the fungal kingdom

**DOI:** 10.1128/spectrum.03717-23

**Published:** 2024-01-05

**Authors:** Wanping Chen, Ye-Eun Son, He-Jin Cho, Dasol Choi, Hee-Soo Park, Jae-Hyuk Yu

**Affiliations:** 1 School of Food Science and Biotechnology, Kyungpook National University, Daegu, South Korea; 2 Department of Bacteriology, University of Wisconsin, Madison, Wisconsin, USA; 3 Department of Integrative Biology, Kyungpook National University, Daegu, South Korea; Centro de Investigaciones Biologicas CSIC, Madrid, Spain

**Keywords:** velvet regulators, fungi, DNA-binding, Rel homology domain, nuclear factor kappa-light-chain-enhancer of activated B cells (NF-κB), fungal development, secondary metabolism

## Abstract

All life forms have evolved to respond appropriately to various environmental and internal cues. In the animal kingdom, the prototypical regulator class of such cellular responses is the Rel homology domain proteins including nuclear factor kappa-light-chain-enhancer of activated B cells (NF-κB). Fungi, the close relatives of animals, have also evolved with their own NF-κB-like regulators called velvet family proteins to govern cellular and chemical development. Here, we conducted a detailed investigation of the taxonomic broad presence of velvet proteins. We observed that velvet proteins are widely distributed in the fungal kingdom. Moreover, we have identified and characterized 21 major velvet clades in fungi. We have further revealed that the highly conserved velvet domain is composed of three distinct motifs and acts as an evolutionarily independent domain, which can be shuffled with various functional domains. Such rearrangements of the velvet domain have resulted in the functional and type diversity of the present velvet regulators. Importantly, our in-deep analyses of the primary and 3D structures of the various velvet domains showed that the fungal velvet domains can be divided into two major clans: the VelB and the VosA clans. The 3D structure comparisons revealed a close similarity of the velvet domain with many other eukaryotic DNA-binding proteins, including those of the Rel, Runt, and signal transducer and activator of transcription families, sharing a common β-sandwich fold. Altogether, this study improves our understanding of velvet regulators in the fungal kingdom.

**IMPORTANCE:**

Fungi are the relatives of animals in Opisthokonta and closely associated with human life by interactive ways such as pathogenicity, food, and secondary metabolites including beneficial ones like penicillin and harmful ones like the carcinogenic aflatoxins. Similar to animals, fungi have also evolved with NF-κB-like velvet family regulators. The velvet proteins constitute a large protein family of fungal transcription factors sharing a common velvet domain and play a key role in coordinating fungal secondary metabolism, developmental and differentiation processes. Our current understanding on velvet regulators is mostly from Ascomycota fungi; however, they remain largely unknown outside Ascomycota. Therefore, this study performed a taxonomic broad investigation of velvet proteins across the fungal kingdom and conducted a detailed analysis on velvet distribution, structure, diversity, and evolution. The results provide a holistic view of velvet regulatory system in the fungal kingdom.

## INTRODUCTION

Life forms have evolved complex adaptations and sensory mechanisms to respond appropriately to various environmental and internal cues (1, 2). For instance, animals have evolved with an elaborate development, inflammation, and immune system for self-defense controlled by various mono- and multi-protein assemblies of the Rel homology domain proteins, including the well-known NF-κB (nuclear factor kappa-light-chain-enhancer of activated B cells) family (3, 4). The NF-κB family consisting of many members has been implicated in a wide range of cellular processes in animals by forming a variety of homodimers or heterodimers to respond to external stimuli (3, 5, 6). Fungi, the close relatives of animals, as they both belong to Opisthokonta with a common ancestor existing approximately 1 billion years ago, have also evolved with specific velvet family regulators with an NF-κB-like DNA-binding domain (4, 7).

**Fig 1:**
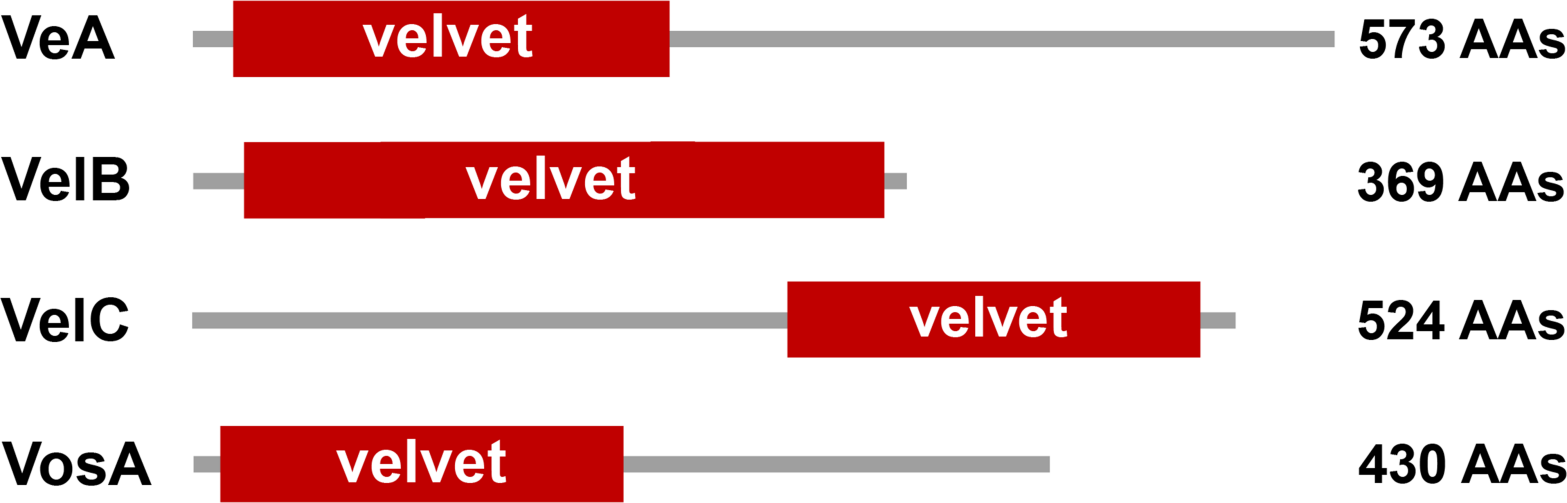
The domain architectures of the four velvet proteins in *A. nidulans*. AAs, amino acid residues.

The velvet proteins constitute a large protein family of fungal transcription factors sharing a common velvet domain and play varied roles in coordinating fungal secondary metabolism, developmental and differentiation processes (8–11). In the model organism *Aspergillus nidulans*, the four well-known members VeA, VelB, VelC, and VosA have been identified and characterized (Fig. 1). The phenotypic outcomes of the four velvet members in *A. nidulans* were summarized in Table 1. Briefly, the founding member VeA was initially described in the model *A. nidulans* in the 1960s as a strain harboring the *veA1* point mutation producing more conidia and fewer fruiting bodies than the wild-type strain (12). Moreover, *veA*-deletion mutant failed to produce any sexual fruiting bodies even under favorable dark conditions, while *veA* overexpression resulted in the constitutive formation of sexual fruiting bodies (13). Much later, the second characterized member VosA was found to be essential for the viability of spores (14). Soon after, the deletion mutant of *velB* (velvet-like protein B) was reported with a similar phenotype to that of *veA* (15, 16). A functional study of VelC, the fourth member of the velvet family in *A. nidulans*, suggested that it functioned as an activator of sexual development (17).

**TABLE 1:** Summary for the phenotypic outcomes of the *velvet* mutants in *A. nidulans^a^*

Velvet member		Asexual development	Sexual development	Secondary metabolism	References
*veA*	KO	Increased conidia production	No sexual structures	Decreased penicillin production; lack of sterigmatocystin (ST) production; increased brownish pigment accumulation	(13, 18, 19)
	OE	Decreased conidia production	Increased sexual structure formation	Decreased penicillin production	
*velB*	KO	Reduced conidia production; increased conidial germination rates; defect of conidia viability; lack of trehalose in conidia	No sexual fruit bodies	Reduced and delayed ST production; increased brownish pigment accumulation	(15, 16, 20)
	OE	A twofold increase of asexual spore production	No excessive production of cleistothecia		
*velC*	KO	Increased conidia production	Reduced production of sexual fruiting bodies (cleistothecia)		(17)
	OE	Equivalent amounts of asexual spores	Increased formation of cleistothecia		
*vosA*	KO	Defect of conidia viability; lack of trehalose in conidia; sensitive to various stresses; increased β-glucan accumulation in conidia; uncontrolled activation of asexual development	Defective sexual fruiting bodies; decreased ascospore viability; lack of trehalose biogenesis; decreased tolerance of ascospores to thermal and oxidative stresses	A slight increase of ST production in ascospores	(14, 21)
	OE	Inhibition of sporulation			

^*a*^ The deletion and overexpression phenotypes of mutants were compared to the wild type. KO, knockout mutants; OE, overexpression mutants.

Additionally, a series of studies involving the characterization of velvet homologs also revealed the importance of the velvet regulatory system in various biological processes in a wide range of fungal species. The functional study of *FvVE1* in *Fusarium verticillioides* was the first characterization of a *veA* homolog gene in a fungal species outside the genus *Aspergillus* (22). Later, lots of related studies were performed in other Ascomycetes, such as *Acremonium chrysogenum* (23), *Cochliobolus heterostrophus* (24), *Histoplasma capsulatum* (25), *Mycosphaerella graminicola* (26), *Neurospora crassa* (27, 28), and *Penicillium chrysogenum* (29). In Basidiomycetes, three *velvet* homologs *umv1*, *umv2*, and *umv3* have been functionally characterized in *Ustilago maydis* (30). Besides, *in silico* analysis of fungal genomes showed that velvet proteins are present across several different fungal taxa (31, 32).

Crystal structure analysis of the VosA velvet domain revealed a structural similarity with the Rel homology domain of the mammalian transcription factor NF-κB (4). In addition, similar to NF-κB members, velvet proteins can form a variety of homodimers, heterodimers, or complexes with various partners having distinct roles in fungal biology. For instance, the heterotrimeric complex VelB-VeA-LaeA governs sexual development and secondary metabolism in *A. nidulans* (15). The VelB homodimer functions as a positive regulator of asexual development, whereas the VosA homodimer plays a negative regulatory role in conidiation during vegetative growth and the early phase of conidiophore formation in *A. nidulans* (16). The VosA-VelB complex is a master regulatory unit for structure, metabolism, and physiology in both asexual and sexual spores in *A. nidulans* (21, 33). Currently, numerous velvet complexes in the genus *Aspergillus* have been reported and summarized (34). Outside the genus *Aspergillus*, the velvet homodimers, heterodimers, or complexes have also been identified in a wide range of fungi, such as *Botrytis cinerea* (35), *Neurospora crassa* (28), *Penicillium chrysogenum* (29, 36), and *Verticillium dahliae* (37).

In this study, we performed a taxonomically broad survey of velvet proteins in the fungal kingdom to reveal their distribution, protein size, domain architecture, etc. Then, we classified the velvet proteins into different clades based on their phylogenetic relationship, and compared the conserved motifs, and 3D structures among the different velvet clades. Results suggested that velvet proteins are blooming in the fungal kingdom but also beyond the kingdom. The velvet domain is highly conserved with three characteristic motifs and could combine with different functional domains to form various velvet proteins. We further revealed that the fungal velvet domains could be divided into two clans (VelB clan and VosA clan). At last, we propose that the velvet domain together with the DNA-binding domains of the Rel, Runt, and signal transducer and activator of transcription (STAT) families sharing a similar β-sandwich fold should belong to the same DNA-binding domain superfamily. Altogether, this study presents a holistic view on the diversity, structure, and evolution of velvet proteins.

## RESULTS

### Diversification of velvet proteins in the fungal kingdom

**Fig 2:**
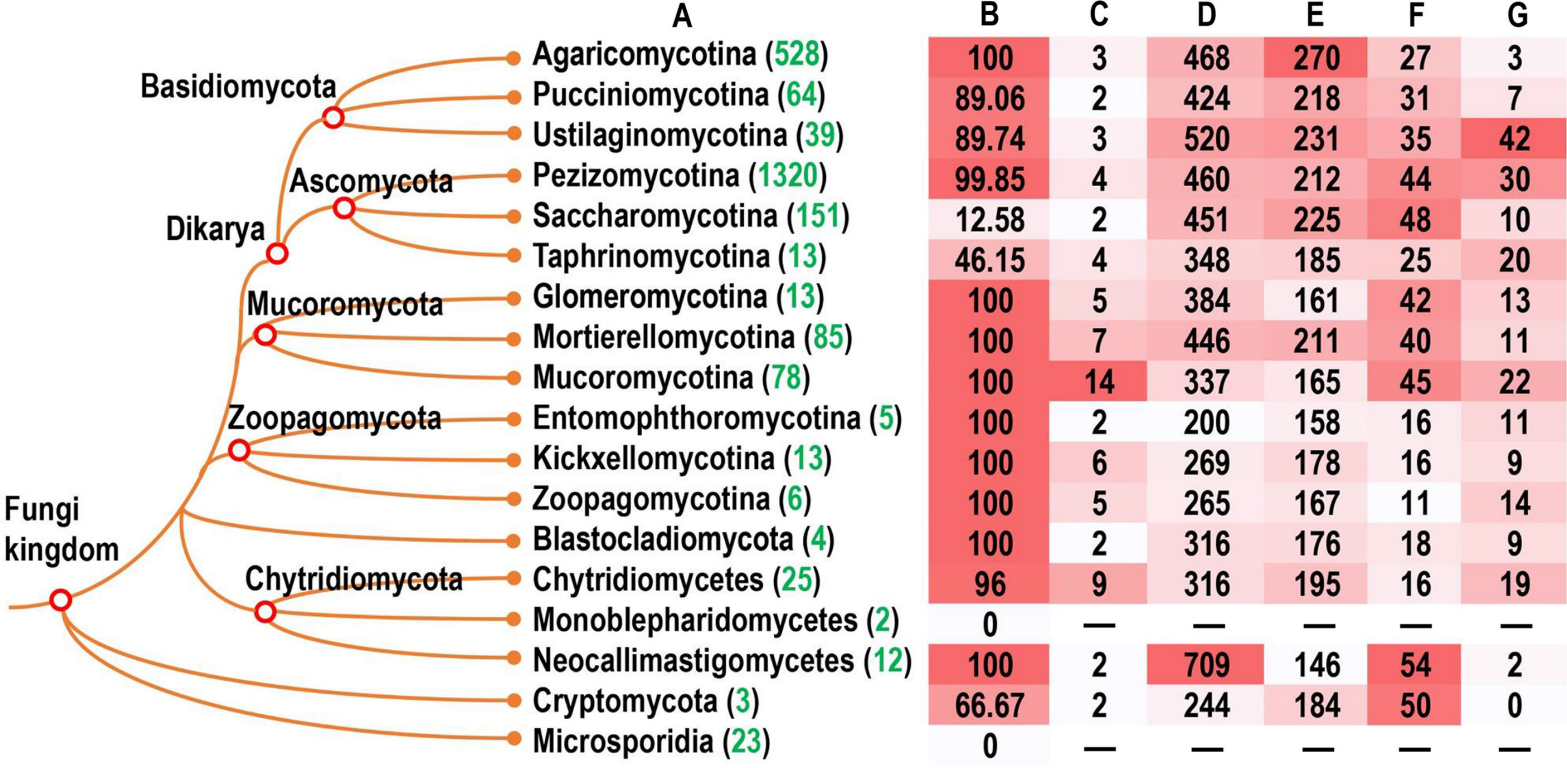
Distribution features of the velvet family in the fungal kingdom. (A) The genome numbers of each fungal group accessed in MycoCosm (38) are highlighted in green. (B) The percentage of genomes having *velvet* genes. (C) The mode of *velvet* gene numbers per genome in each fungal group. The detailed information is provided in Fig. 3. (D) The average length of velvet proteins (amino acid residues) in each fungal group. The detailed information is provided in Fig. 4. (E) The average length of velvet domains (amino acid residues) in each fungal group. (F) The percentage of N-terminal side located velvet domains in each fungal group. The detailed information is provided in Fig. 5. (G) The percentage of C-terminal side located velvet domains in each fungal group.

To address the diversity of velvet proteins in the fungal kingdom, their homologs were investigated in a wide range of fungi covering the phyla Ascomycota, Basidiomycota, Blastocladiomycota, Chytridiomycota, Cryptomycota, Microsporidia, Mucoromycota, and Zoopagomycota. The distribution information of *velvet* genes in terms of their frequency and family diversity in genomes was summarized in the tested fungi (Fig. 2).

**Fig 3:**
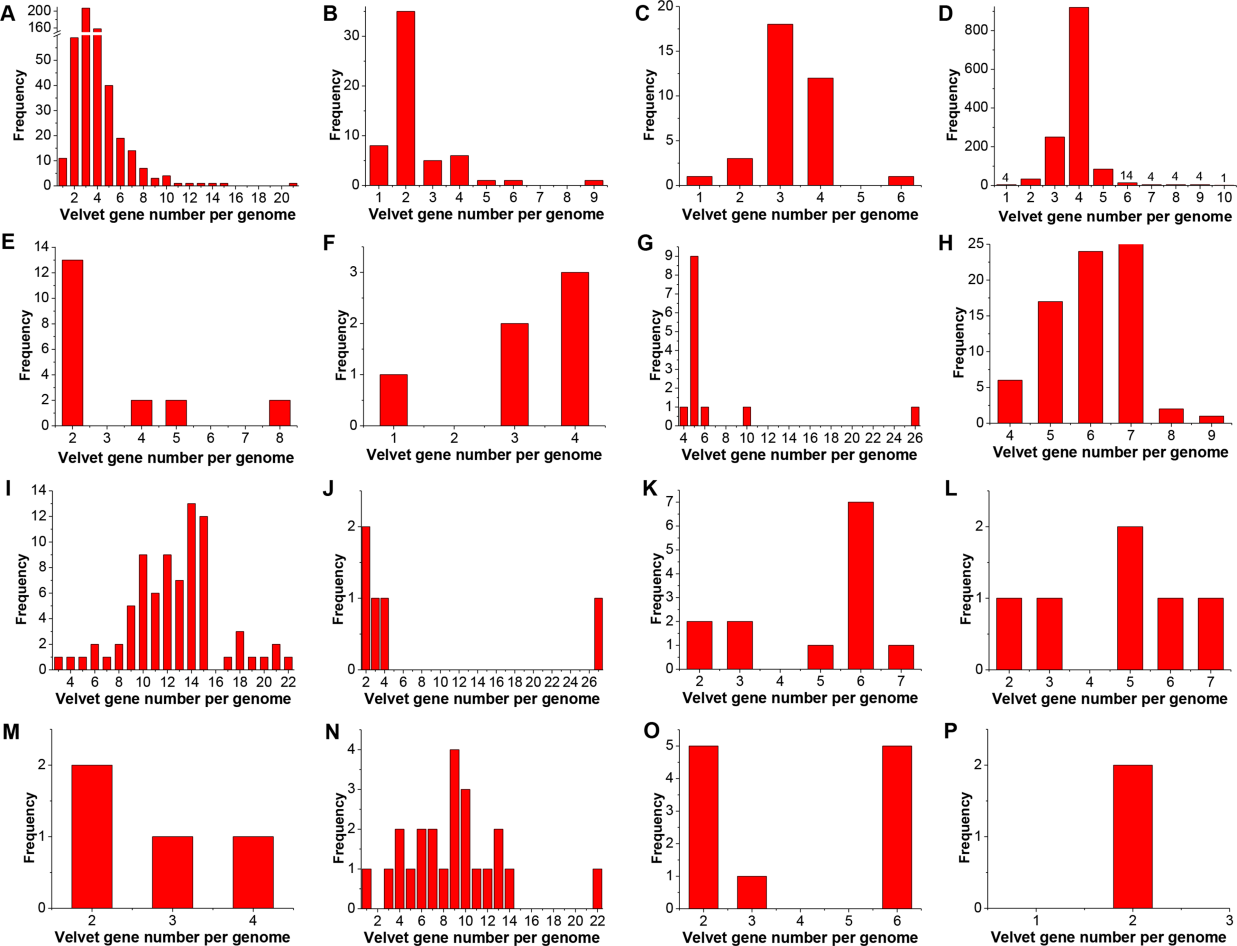
The frequency distribution of *velvet* gene number per genome in different fungal groups. The genomes without *velvet* genes were not counted. A, B, and C, respectively, correspond to the groups Agaricomycotina, Pucciniomycotina, and Ustilaginomycotina in the phylum Basidiomycota. D, E, and F, respectively, correspond to the groups Pezizomycotina, Saccharomycotina, and Taphrinomycotina in the phylum Ascomycota. G, H, and I, respectively, correspond to the groups Glomeromycotina, Mortierellomycotina, and Mucoromycotina in the phylum Mucoromycota. J, K, and L, respectively, correspond to the groups Entomophthoromycotina, Kickxellomycotina, and Zoopagomycotina in the phylum Zoopagomycota. M corresponds to the phylum Blastocladiomycota. N and O, respectively, correspond to the groups Chytridiomycetes and Neocallimastigomycetes in the phylum Chytridiomycota. P corresponds to the phylum Cryptomycota.

In general, velvet proteins are widespread in the tested fungal phyla from higher fungi to lower fungi, except for their absence in Microsporidia (Fig. 2A and B). Among the tested taxonomic groups, most genomes contained *velvet* genes, but it was observed that no *velvet* genes were detected in the current two Monoblepharidomycete genomes, and only part of Ascomycota yeasts contained velvet genes. The mode of *velvet* gene number per genome varied among the taxonomic groups with the count ranging from 2 to 14 (Fig. 2C). Furthermore, the frequency distribution of *velvet* gene number per genome was also compared among different fungal taxonomic groups (Fig. 3). In general, the distribution varies greatly by the taxonomic groups. For instance, among the 1,320 Pezizomycotina genomes, approximately 70% contained four velvet genes and 19% contained three velvet genes, whereas approximately 39% of the 528 Agaricomycotina genomes harbored three velvet genes and 30% contained four velvet genes. In particular, the quantity of velvet genes outbreaks in the Mucoromycotina genomes and approximately 82% harbored more than 10 *velvet* genes.

**Fig 4:**
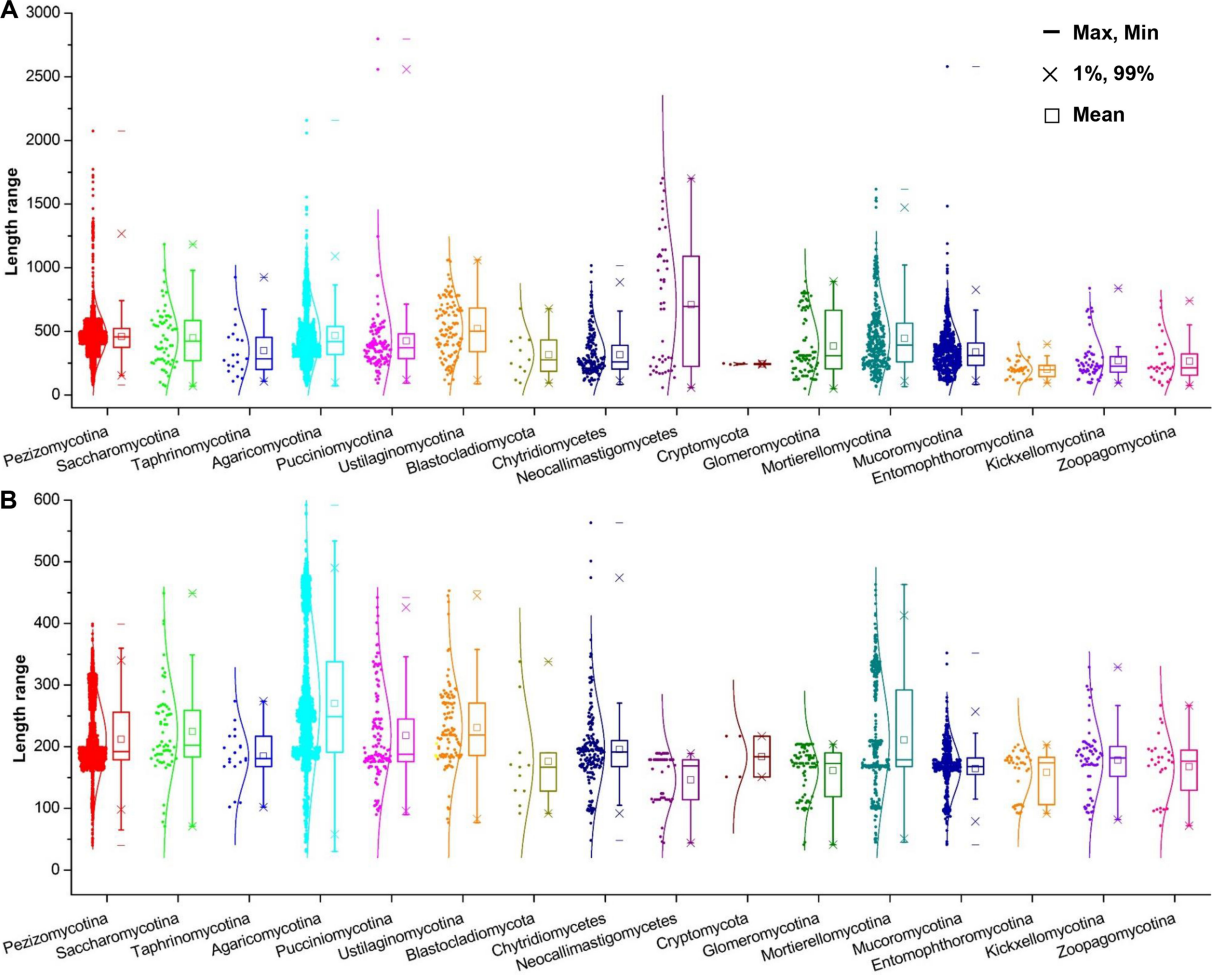
Length box charts of velvet proteins and domains in different fungal groups. The length was calculated as the number of amino acid residues. Normal distribution was used to fit the length distribution. A and B, respectively, correspond to the charts of velvet proteins and domains.

The length distribution of velvet proteins varied significantly both within and among the taxonomic groups, ranging from several hundreds to thousands (Fig. 2D and 4A). The Neocallimastigomycetes velvet proteins showed the longest average length of 709 amino acid residues (AAs); however, a general trend was observed that the average length in higher fungi is longer than that in lower fungi. The velvet domain length is around 200 AAs (Fig. 2E and 4B). The Agaricomycotina velvet proteins harbored velvet domains with the longest average length of 270 AAs. In general, the average length of the velvet domain in higher fungi is longer than that in lower fungi.

**Fig 5:**
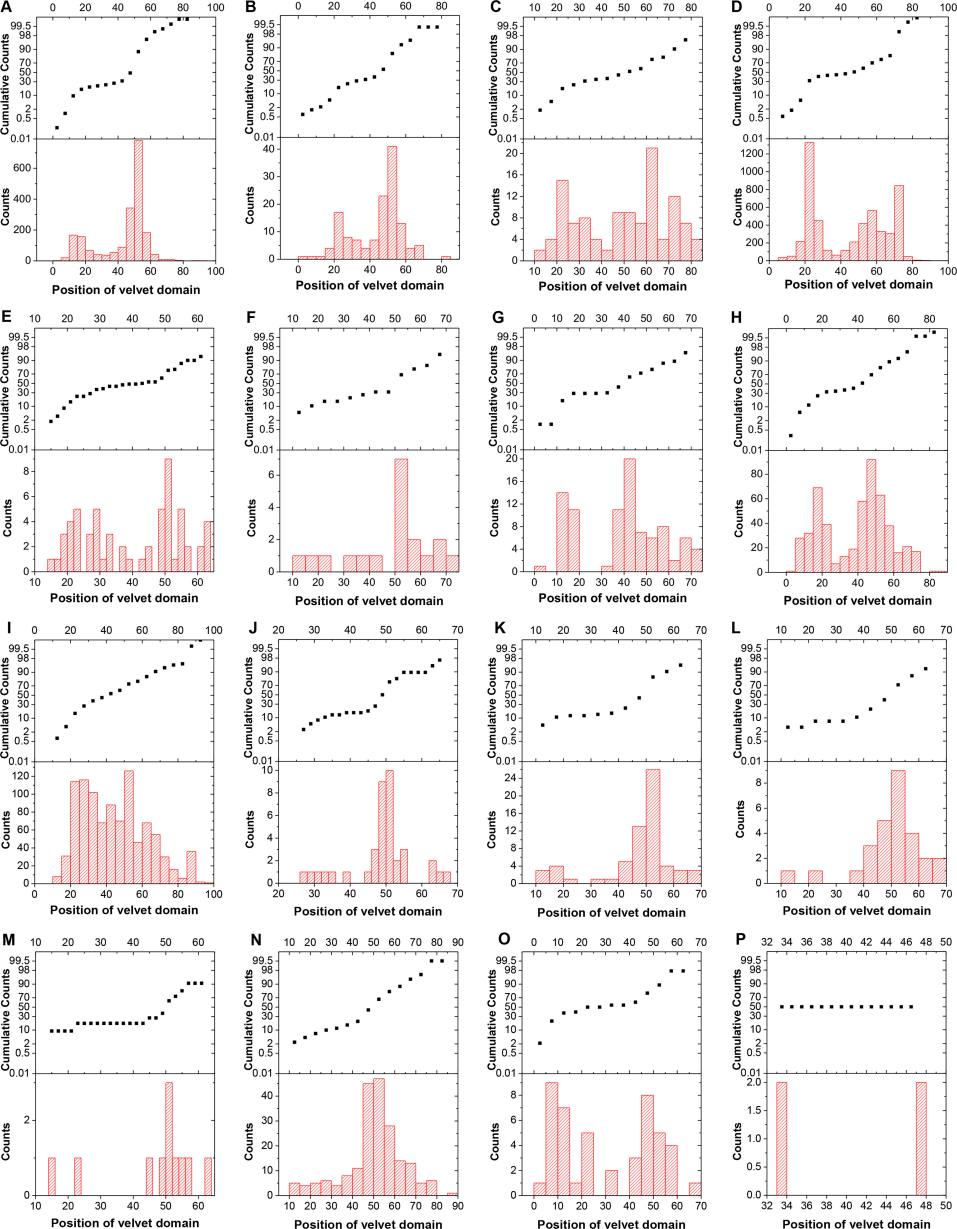
The distribution of velvet domain position in different fungal groups. The position of velvet domain in a protein was calculated as the midpoint of velvet domain divided by the protein length (the number of amino acid residues). The position of velvet domain locating before 40% was defined as N-terminal side of the protein, while that locating after 60% was defined as C-terminal side of the protein and others are middle part of the protein. A, B, and C, respectively, correspond to the groups Agaricomycotina, Pucciniomycotina, and Ustilaginomycotina in the phylum Basidiomycota. D, E, and F, respectively, correspond to the groups Pezizomycotina, Saccharomycotina, and Taphrinomycotina in the phylum Ascomycota. G, H, and I, respectively, correspond to the groups Glomeromycotina, Mortierellomycotina, and Mucoromycotina in the phylum Mucoromycota. J, K, and L, respectively, correspond to the groups Entomophthoromycotina, Kickxellomycotina, and Zoopagomycotina in the phylum Zoopagomycota. M corresponds to the phylum Blastocladiomycota. N and O, respectively, correspond to the groups Chytridiomycetes and Neocallimastigomycetes in the phylum Chytridiomycota. P corresponds to the phylum Cryptomycota.

The position of velvet domains was investigated and compared among the taxonomic groups (Fig. 2F, G and 5). As revealed, the velvet domain could be located in the N-terminal side, middle, or C-terminal side of proteins, but its position distribution varies among different fungal taxonomic groups. In Ustilaginomycotina and Pezizomycotina, the velvet domains located in the C-terminal side occupy a large proportion with the percentage over 30%; however, on the whole, the percentage of velvet domains in the N-terminal side is higher than that in the C-terminal side in the phyla Ascomycota, Basidiomycota, and Mucoromycota. In the phyla Blastocladiomycota and Zoopagomycota, the velvet proteins are shorter and the velvet domains occupy a large part of the proteins.

### Grouping of Ascomycota velvet proteins and their features

**Fig 6:**
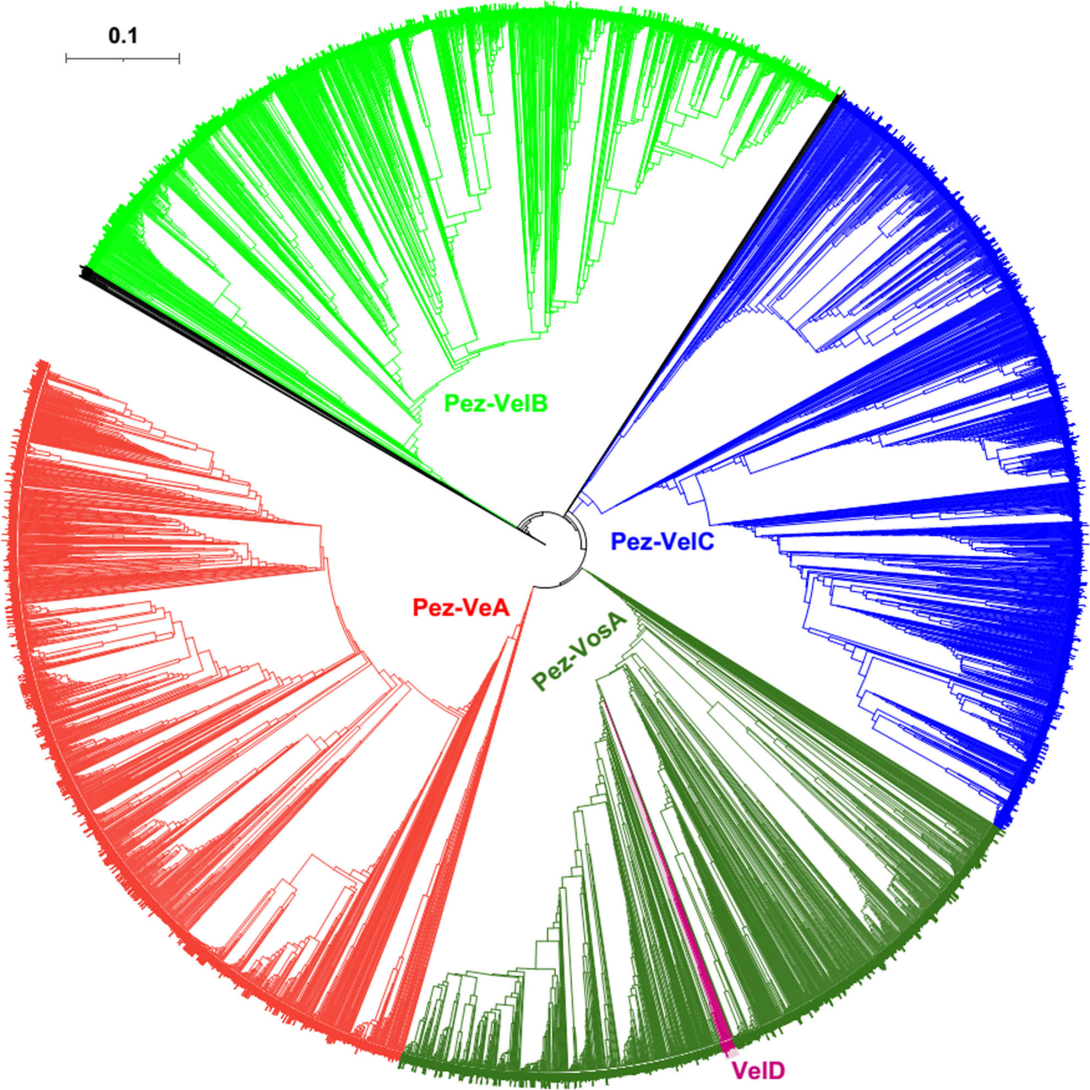
Phylogenetic relationship of the Pezizomycotina velvet proteins. The branch length of the tree is indicated by the scale bar in the upper left corner. The clades of these velvet proteins are indicated by their colors. The figure in high definition is provided as Fig. S1.

**Fig 7:**
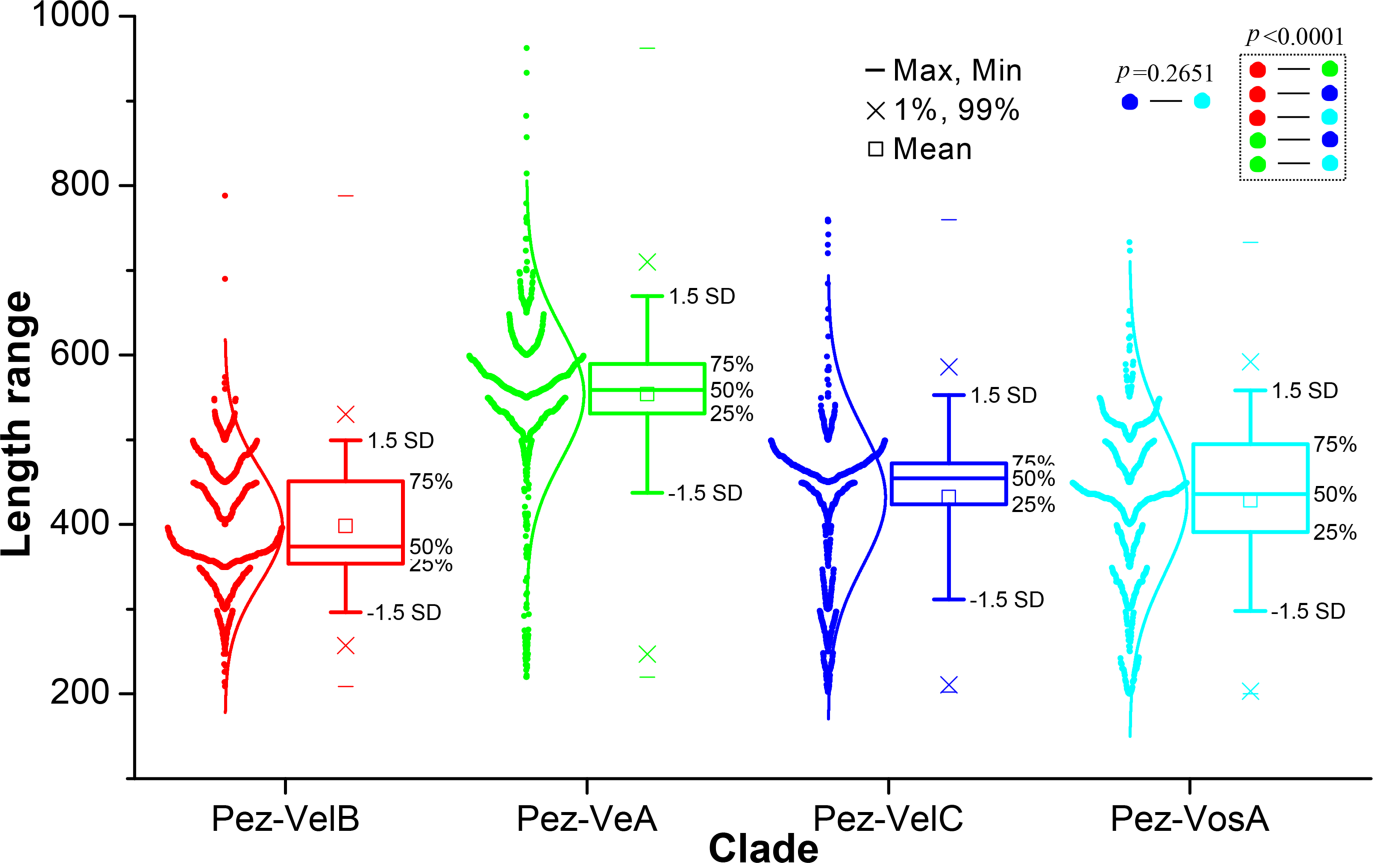
Length distribution of clades Pez-VeA, Pez-VelB, Pez-VelC, and Pez-VosA in Pezizomycotina shown as box plots. The clades were based on the phylogenetic relationship shown in Fig. 6. The length was calculated as the number of amino acid residues of velvet proteins. The normal distribution was used to fit the length distribution. Two-group comparisons were performed using the *t*-test.

Pezizomycotina constitutes the majority (approximately 90%) of Ascomycota fungi (39). The phylogeny of the Pezizomycotina velvet proteins was analyzed and they were clearly classified into four main clades, Pez-VeA, Pez-VelB, Pez-VelC, and Pez-VosA, based on their phylogenetic relationships (Fig. 6). VelDs, as the fifth velvet member found in most species of *Aspergillus* section Flavi (40), are gathered in a branch inside the Pez-VosA clade based on the phylogenetic relationship. The length distribution of velvet proteins among the four clades was compared (Fig. 7). It showed that the average protein lengths were 554 AAs in the Pez-VeA clade, 398 AAs in the Pez-VelB clade, 432 AAs in the Pez-VelC clade, and 428 AAs in the Pez-VosA clade. Based on the statistical analysis, except for the comparison between the Pez-VelC clade and the Pez-VosA clade, other two-group comparisons of length distribution indicated an extremely significant departure (*P* < 0.0001).

**Fig 8:**
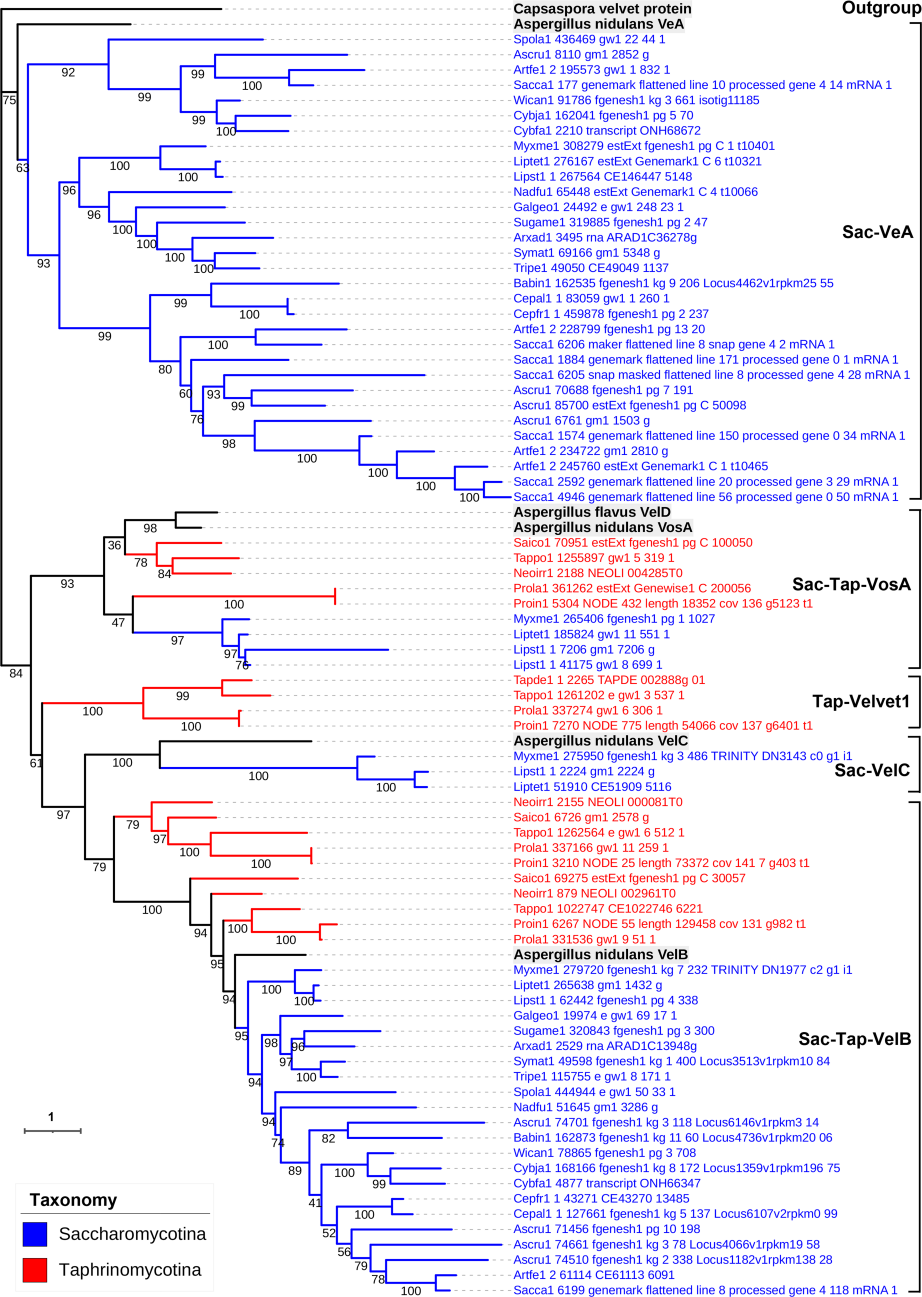
Phylogenetic relationship of Saccharomycotina and Taphrinomycotina velvet proteins. The *Capsaspora* velvet protein was used as an outgroup. *Aspergillus flavus* VelD and *A. nidulans* VeA, VelB, VelC, and VosA were used as references. Bootstrap values for each node are presented. The branch length of each tree is indicated by the scale bar in the lower left corner. The taxonomic groups of these velvet proteins are indicated by their colors paraphrasing in the lower left corner. The clades are marked on the right.

Unlike in Pezizomycotina, *velvet* genes were detected only in a part of the genomes in Saccharomycotina and Taphrinomycotina (Fig. 2B). The phylogenetic tree of velvet proteins of Saccharomycotina and Taphrinomycotina was constructed (Fig. 8). In general, although Saccharomycotina and Taphrinomycotina are relatives of Pezizomycotina within the phylum Ascomycota, they showed a significant difference in the distribution of velvet clades. As shown in Fig. 8, the Saccharomycotina velvet proteins could also be classified into the clades Sac-VeA, Sac-Tap-VelB, Sac-VelC, and Sac-Tap-VosA, but the clades Sac-VeA and Sac-Tap-VelB are dominant. The Taphrinomycotina velvet proteins were classified into the clades Sac-Tap-VelB, Sac-Tap-VosA, and a new clade named Tap-Velevt1. Unexpectedly, no Taphrinomycotina velvet proteins were detected in the clades VeA and VelC.

### Grouping of Basidiomycota velvet proteins and their features

**Fig 9:**
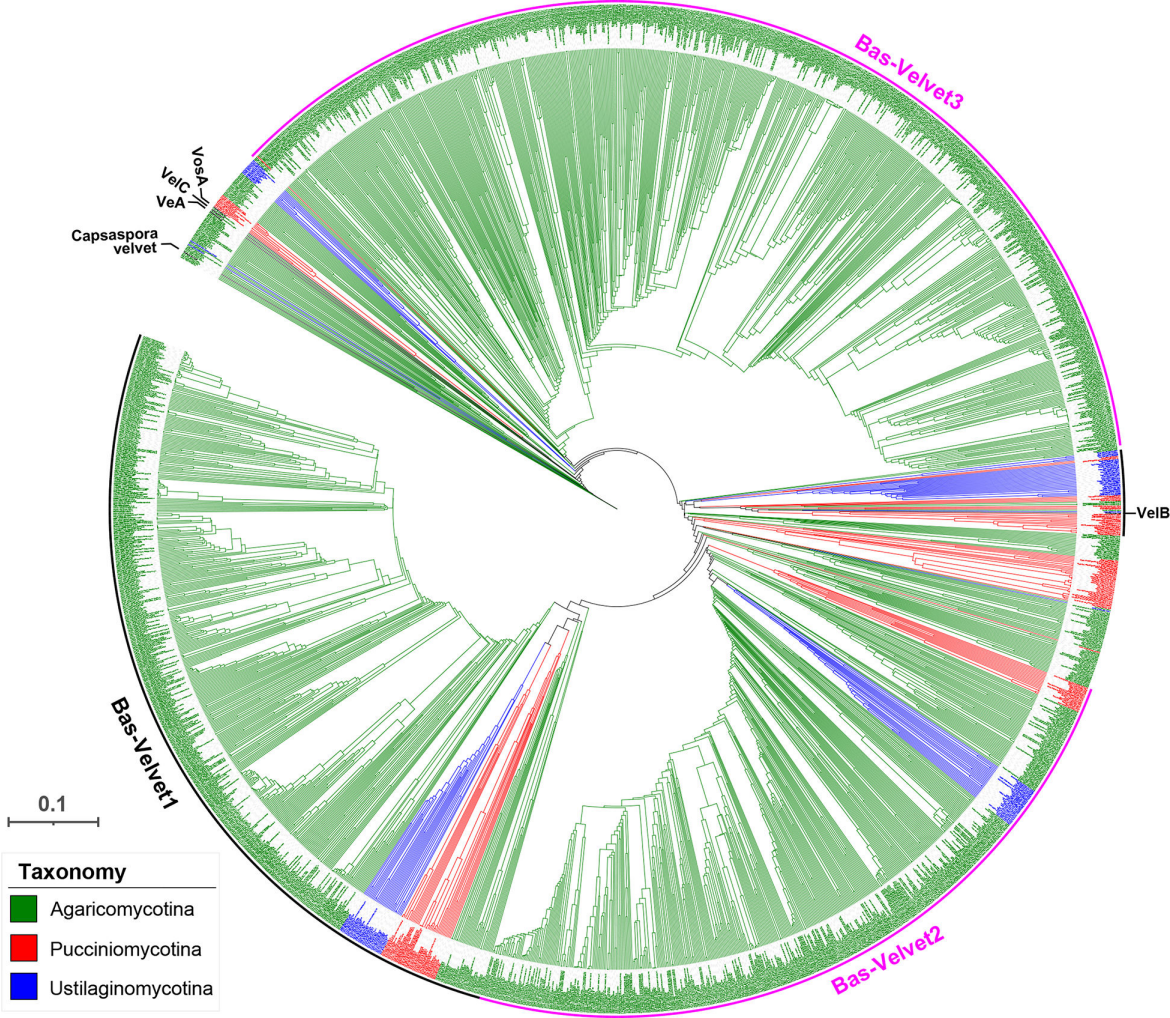
Phylogenetic relationship of the Basidiomycota velvet proteins. The position of the references *Capsaspora* velvet protein, *A. nidulans* VeA, VelB, VelC, and VosA is indicated on the outer. The tree branch length is indicated by the scale bar in the lower left corner. The taxonomic groups of these velvet proteins are indicated by their colors paraphrasing in the lower left corner. The clades are marked on the outer. The figure in high definition is provided as Fig. S2 to S7.

A phylogenetic tree of the Basidiomycota velvet proteins was constructed (Fig. 9). Basidiomycota together with Ascomycota constitutes the subkingdom Dikarya; however, they show extremely different repertoires of velvet members. Based on the phylogenetic relationship, except for a small VelB clade found among the Basidiomycota velvet proteins, no VeA, VelC, and VosA clades formed in the tree. In contrast, three new major clades (Bas-Velvet1, Bas-Velvet2, and Bas-Velvet3) were assigned for the Basidiomycota velvet proteins. The clades Bas-Velvet1 and Bas-Velvet2 consisted of members from the three subphyla Agaricomycotina, Pucciniomycotina, and Ustilaginomycotina. However, the vast majority of members of the clade Bas-Velvet3 are from the subphylum Agaricomycotina. In the VelB clade, the members are primarily from the subphyla Pucciniomycotina and Ustilaginomycotina.

**Fig 10:**
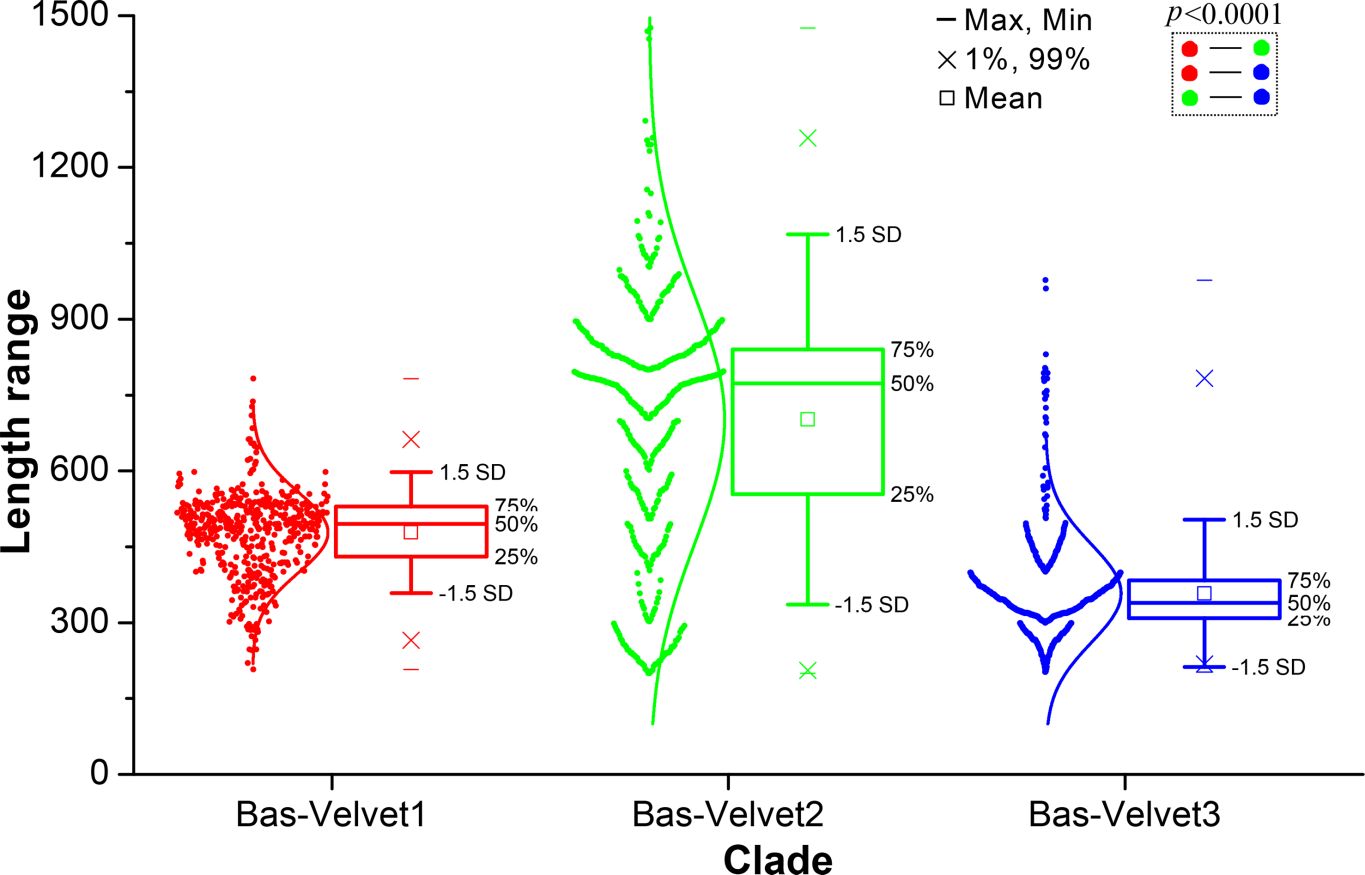
Length distribution of clades Bas-Velvet1, Bas-Velvet2, and Bas-Velvet3 in Basidiomycota shown as box plots. The clades were based on the phylogenetic relationship shown in Fig. 9. The length was calculated as the number of amino acid residues of velvet proteins. The normal distribution was used to fit the length distribution. Two-group comparisons were performed using the *t*-test.

The length distribution of velvet proteins in the three major clades was investigated (Fig. 10). The average protein lengths were 478 AAs in the Bas-Velvet1 clade, 702 AAs in the Bas-Velvet2 clade, and 358 AAs in the Bas-Velvet3 clade. The two-group comparisons of length distribution among the clades indicated an extremely significant departure (*P* < 0.0001) in the statistical analysis.

### Grouping of Mucoromycota velvet proteins and their features

**Fig 11:**
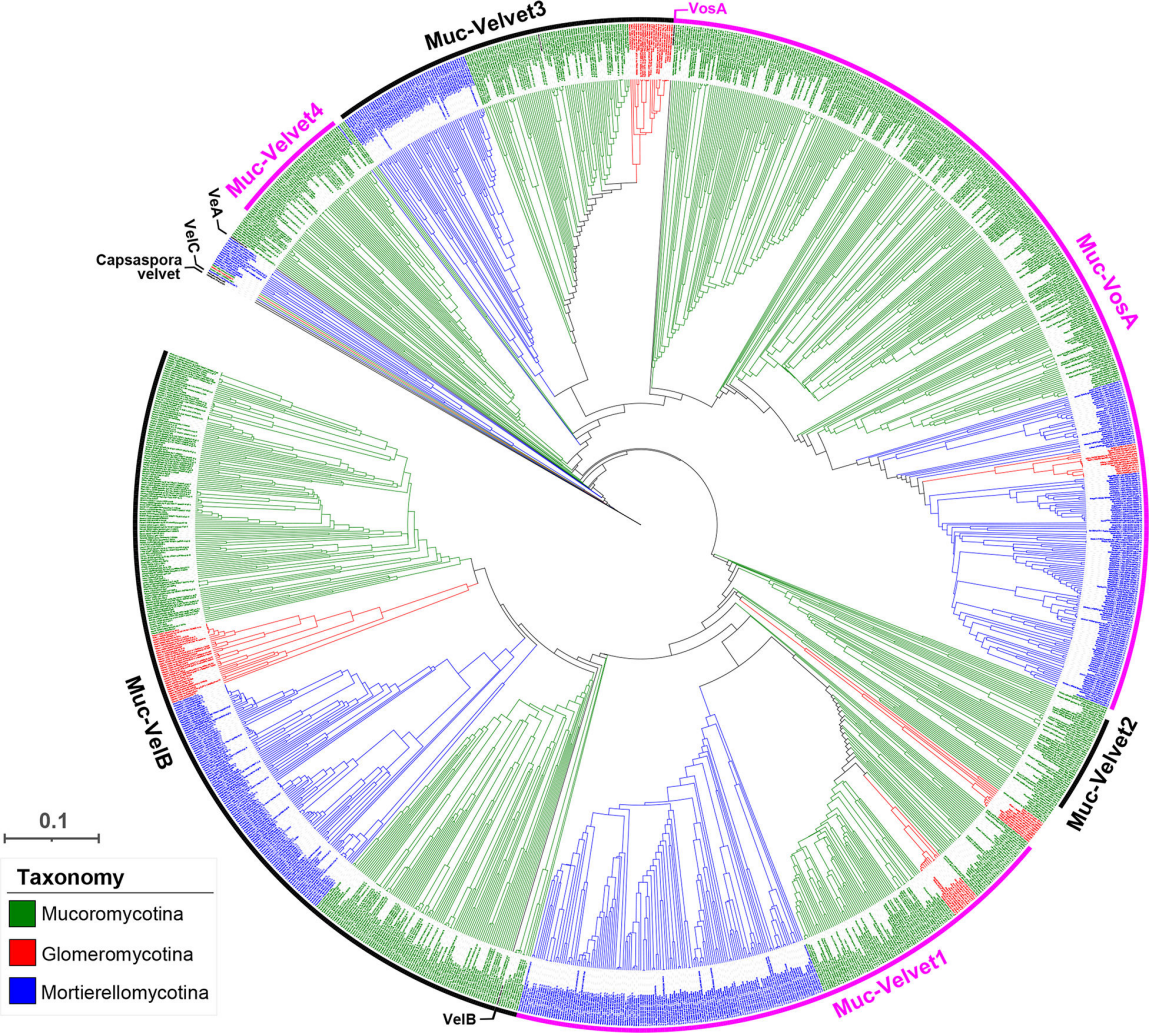
Phylogenetic relationship of the Mucoromycota velvet proteins. The *Capsaspora* velvet protein was used as the outgroup. *A. nidulans* VeA, VelB, VelC, and VosA were used as references. The branch length of each tree is indicated by the scale bar in the lower left corner. The taxonomic groups of these velvet proteins are indicated by their colors paraphrasing in the lower left corner. The clades are marked on the outer. The figure in high definition is provided as Fig. S3.

A phylogenetic tree of the Mucoromycota velvet proteins was constructed (Fig. 11). As shown, six major clades (Muc-Velvet1, Muc-Velvet2, Muc-Velvet3, Muc-Velvet4, Muc-VelB, and Muc-VosA) were formed in the tree. The clades Muc-VelB and Muc-VosA are the two large divisions of the Mucoromycota velvet proteins, covering the subphyla Glomeromycotina, Mortierellomycotina, and Mucoromycotina. However, no VeA and VelC clades were formed in the tree. Muc-Velvet1 and Muc-Velvet3 are the two newly allocated clades for the Mucoromycota velvet proteins, and both cover the three subphyla. Muc-Velvet2 and Muc-Velvet4 are the two Mucoromycotina-specific clades.

**Fig 12:**
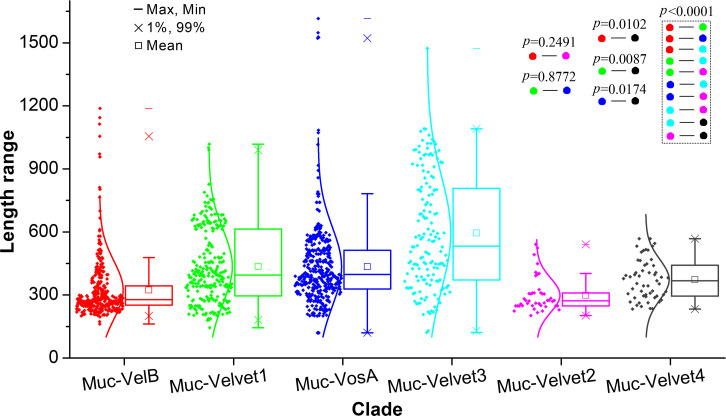
Length distribution of the clades Muc-Velvet1, Muc-Velvet2, Muc-Velvet3, Muc-Velvet4, Muc-VelB, and Muc-VosA in Mucoromycota shown as box plots. The clades were based on the phylogenetic relationship shown in Fig. 11. The length was calculated as the number of amino acid residues of velvet proteins. The normal distribution was used to fit the length distribution. Two-group comparisons were performed using the *t*-test.

The length distribution of velvet proteins in the six major clades was compared (Fig. 12). The average protein lengths were 436 AAs in the Muc-Velvet1 clade, 299 AAs in the Muc-Velvet2 clade, 595 AAs in the Muc-Velvet3 clade, 373 AAs in the Muc-Velvet4 clade, 323 AAs in the Muc-VelB clade, and 435AAs in the Muc-VosA clade. Based on the two-group comparisons, the length distributions of the Muc-VelB clade vs the Muc-Velvet2 clade and the Muc-Velvet1 clade vs the Muc-VosA clade showed no statistically significant difference, whereas other two-group comparisons revealed statistically significant differences.

### Grouping of Blastocladiomycota, Chytridiomycota, Cryptomycota, and Zoopagomycota velvet proteins and their features

**Fig 13:**
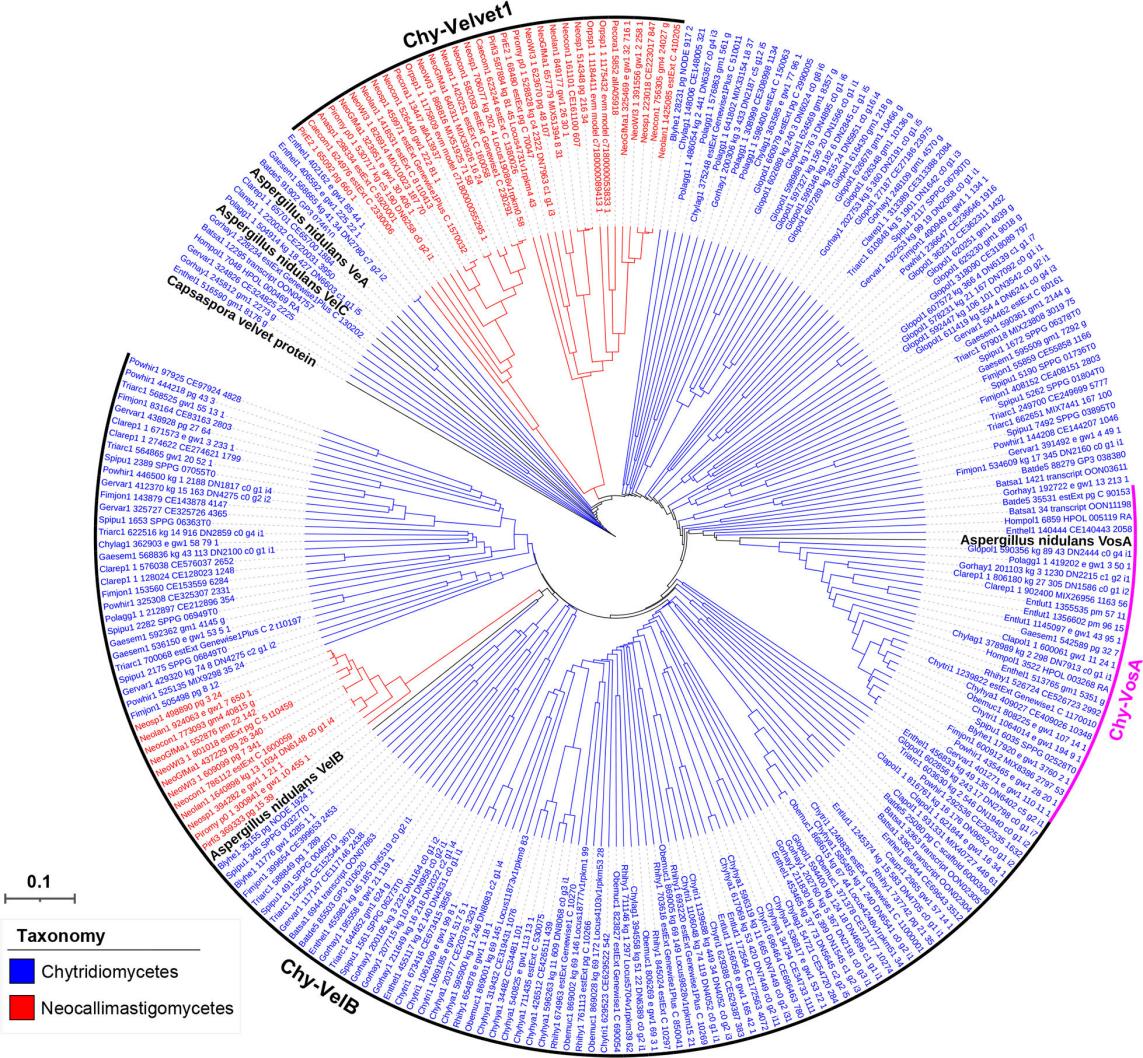
Phylogenetic relationship of Chytridiomycota velvet proteins. The *Capsaspora* velvet protein was used as the outgroup. *A. nidulans* VeA, VelB, VelC, and VosA were used as references. They are highlighted in bold. The branch length of each tree is indicated by the scale bar in the lower left corner. The taxonomic groups of these velvet proteins are indicated by their colors paraphrasing in the lower left corner. The clades are marked on the right.

A phylogenetic tree of the Chytridiomycota velvet proteins was constructed (Fig. 13). Based on the phylogenetic relationship, three major clades (Chy-VelB, Chy-VosA, and Chy-Velvet1) were formed among the Chytridiomycota velvet proteins. Chy-VelB is a large clade containing proteins from the classes Chytridiomycetes and Neocallimastigomycetes. The Chy-VosA clade is Chytridiomycetes-specific, whereas the Chy-Velvet1 clade is Neocallimastigomycetes-specific. No VeA and VelC clades were formed in the tree.

**Fig 14:**
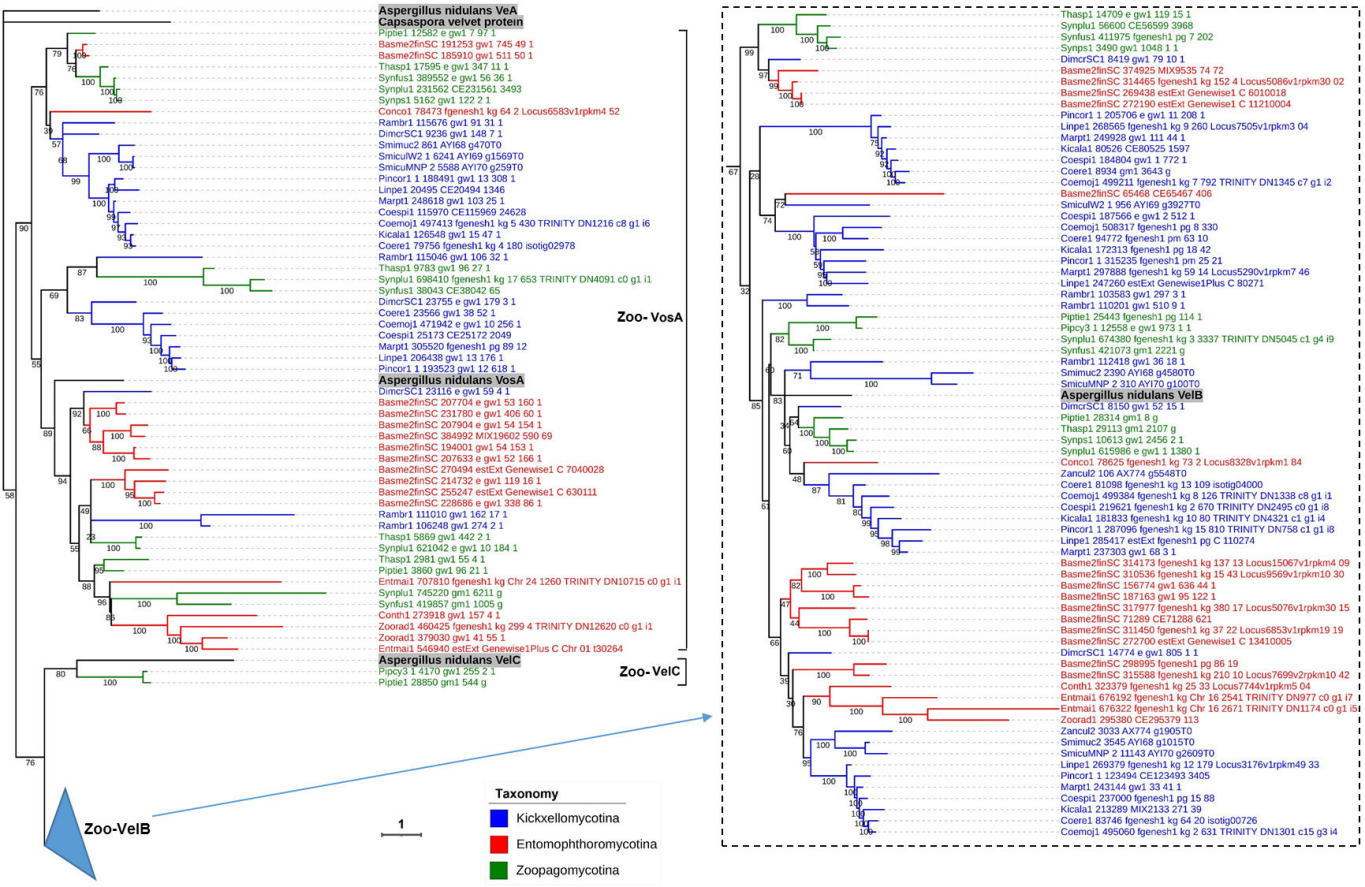
Phylogenetic relationship of Zoopagomycota velvet proteins. The *Capsaspora* velvet protein was used as the outgroup. *A. nidulans* VeA, VelB, VelC, and VosA were used as references. They are highlighted in bold. Bootstrap values for each node are presented. The branch length of each tree is indicated by the scale bar in the lower left corner. The taxonomic groups of these velvet proteins are indicated by their colors paraphrasing in the lower middle. The clades are marked on the right of protein IDs. The VelB clade is collapsed in the tree, and expands on the right.

The Zoopagomycota velvet proteins could be grouped into three clades (Zoo-VelB, Zoo-VosA, and Zoo-VelC) (Fig. 14). The clades Zoo-VelB and Zoo-VosA are two large divisions of the Zoopagomycota velvet proteins, covering the subphyla Entomophthoromycotina, Kickxellomycotina, and Zoopagomycotina. The clade Zoo-VelC is a small branch and Zoopagomycotina-specific.

**Fig 15:**
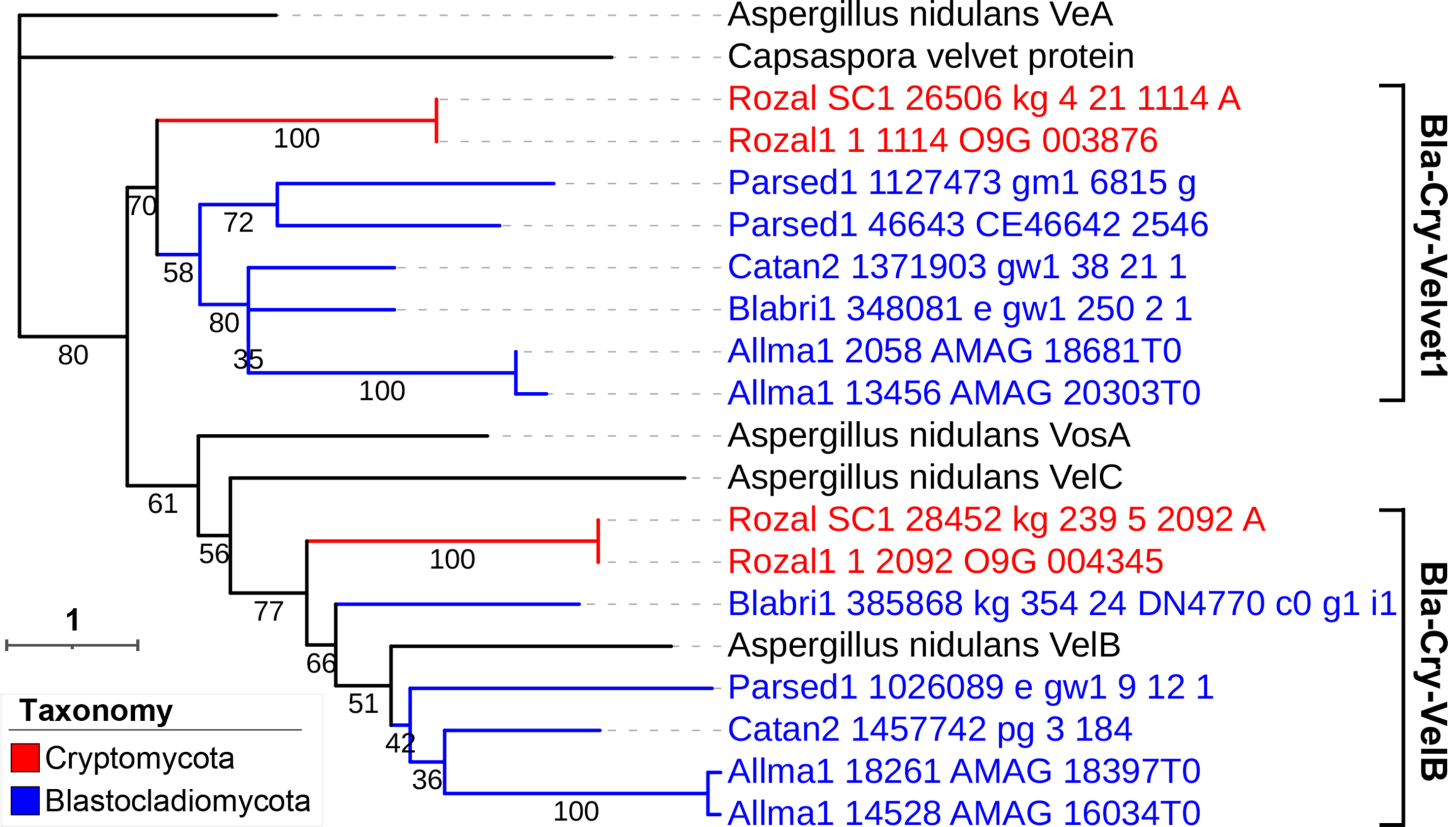
Phylogenetic relationship of Blastocladiomycota and Cryptomycota velvet proteins. The *Capsaspora* velvet protein was used as the outgroup. *A. nidulans* VeA, VelB, VelC, and VosA were used as references. Bootstrap values for each node are presented. The branch length of each tree is indicated by the scale bar in the lower left corner. The taxonomic groups of these velvet proteins are indicated by their colors paraphrasing in the lower middle. The clades are marked on the right of protein IDs.

The Blastocladiomycota and Cryptomycota velvet proteins were presented on the same tree (Fig. 15). The velvet proteins were grouped into two clades (Bla-Cry-Velvet1 and Bla-Cry-VelB). Bla-Cry-Velvet1 is a new clade and no VeA, VelC, and VosA clades were found in the tree.

### Comparison of velvet domain features of the 21 clades

**Fig 16:**
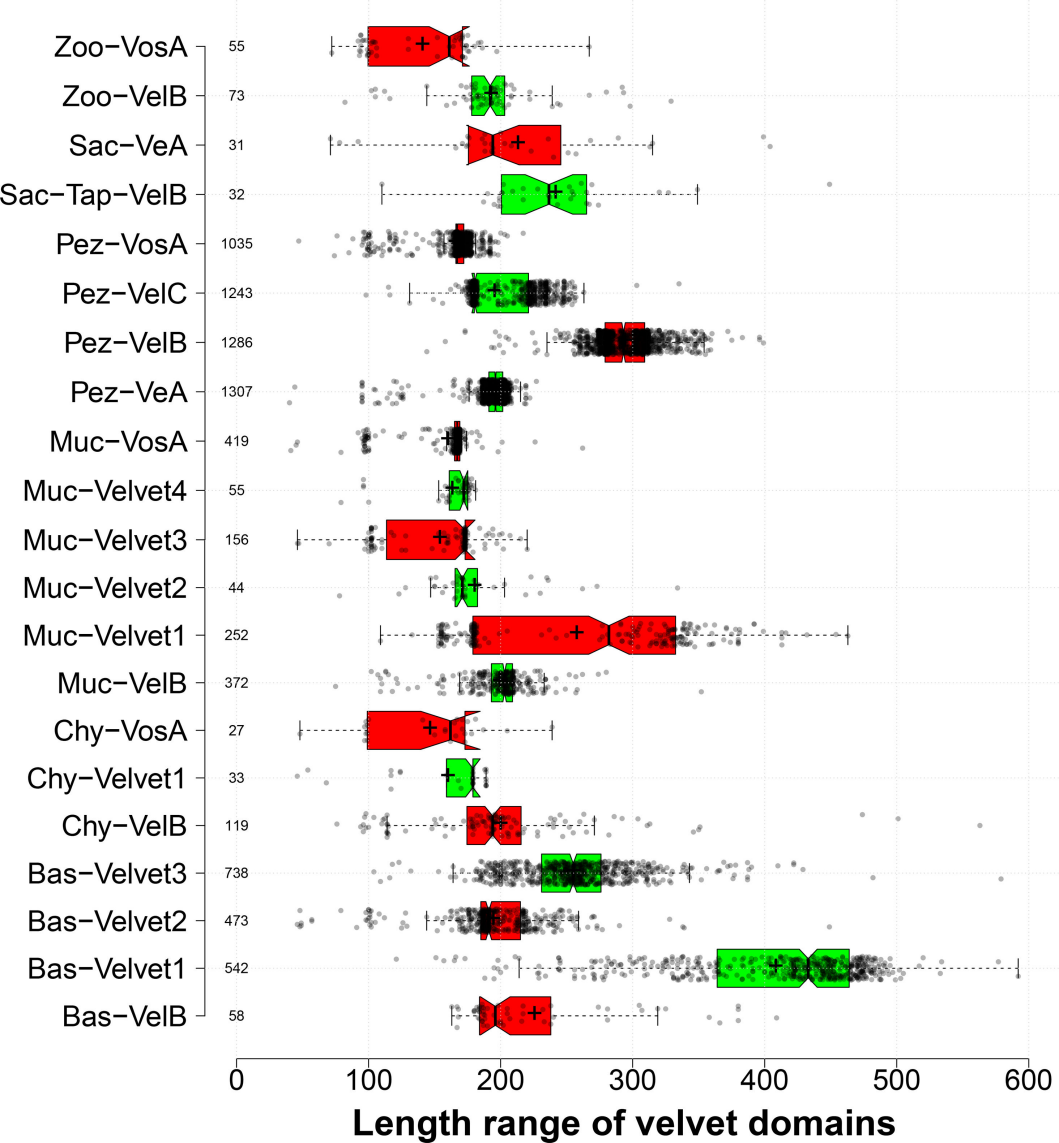
Length distribution of velvet domains from the 21 major clades shown as box plots. The clades were based on the aforementioned phylogenetic analysis. The length was calculated as the number of amino acid residues of velvet domains. The figure was generated by BoxPlotR (41). Data points are shown in a jittered mode with the Tukey whisker extent. The notches were added to the boxes in the presence of medians, and the symbol + indicates the mean value.

The length distribution of velvet domains from the 21 major clades was compared (Fig. 16). In general, most velvet domains are around 200 AAs, but there are also some individual differences. For example, among the four well-known members in Pezizomycotina, the average length of Pez-VeA velvet domains was 194 AAs, extremely close to that of Pez-VelC velvet domains of 195 AAs, whereas the average length of Pez-VosA velvet domains was much shorter at around 166 AAs, but that of Pez-VelB velvet domains was much longer at around 294 AAs. In particular, the Bas-Velvet1 clade possessed a very long velvet domain with an average length of 408 AAs.

**Fig 17:**
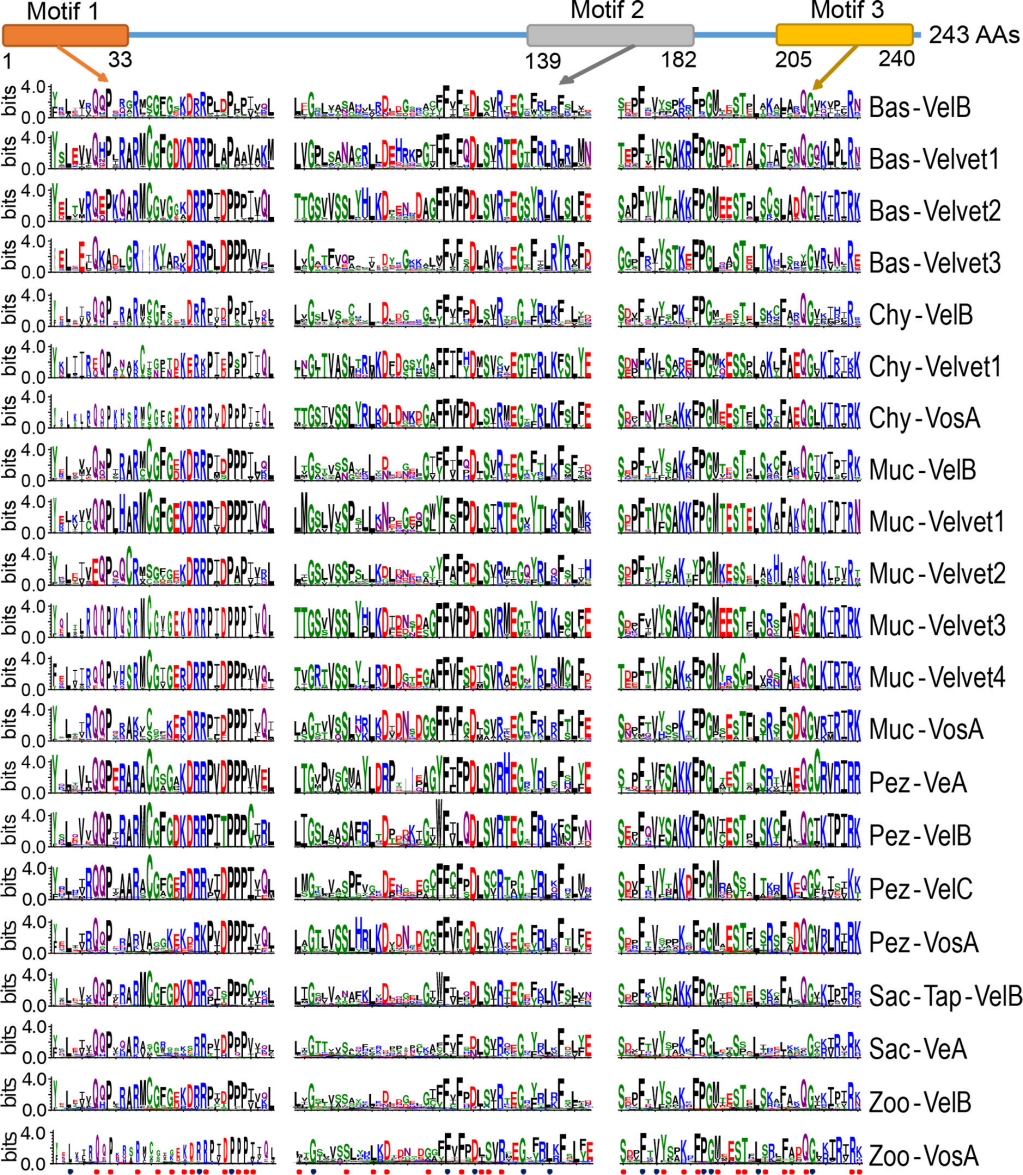
Comparison of the three characteristic motifs of velvet domains among the 21 major clades. The alignment of velvet domains was performed against the profile hidden Markov model of velvet domain PF11754 with 243 residues (https://www.ebi.ac.uk/interpro/entry/pfam/PF11754/) and then subjected to WebLogo (https://weblogo.threeplusone.com/) to generate sequence logos. In the logo, the total stack height represents the information content of residues at that position. The relative height of each residue in the stack is proportional to its frequency at the position, and the residues were sorted so that the most common one was on the top of the stack. The full sequence logos of velvet domains are provided in Fig. S4. The residues are colored according to their chemical properties, of which polar ones G, S, T, Y, and C are in green; neutral ones Q and N are in purple; basic ones K, R, and H are in blue; acidic ones D and E are in red; and hydrophobic ones A, V, L, I, P, W, F, and M are in black. The black balls at the bottom indicate the consensus dominant residues in the 21 clades, and the red balls indicate the other conserved residues revealed by the ConSurf analysis.

Then, the conserved residues of velvet domains among the 21 major clades were compared (Fig. S4) and the three characteristic motifs were revealed (Fig. 17). In general, the N-terminal region harbors a conserved motif of around 33 residues (termed motif 1) and the C-terminal region contains a characteristic motif of around 36 residues (termed motif 3). The characteristic motif 2 with around 44 residues is close motif 3. The large region between motif 1 and 2 is not conserved in terms of both sequence and length.

The three characteristic motifs embody both commonalities and differences across the 21 velvet domains. On the one hand, the 21 velvet domains share up to 48 conserved sites in their characteristic motifs, 14 of which are with consensus dominant residues across the 21 velvet domains. On the other hand, different velvet members may also be distinguishable by their specific motifs or residues. For example, among the four members of Pezizomycotina, their signatures from the position 140 to 149 are quite different.

**Fig 18:**
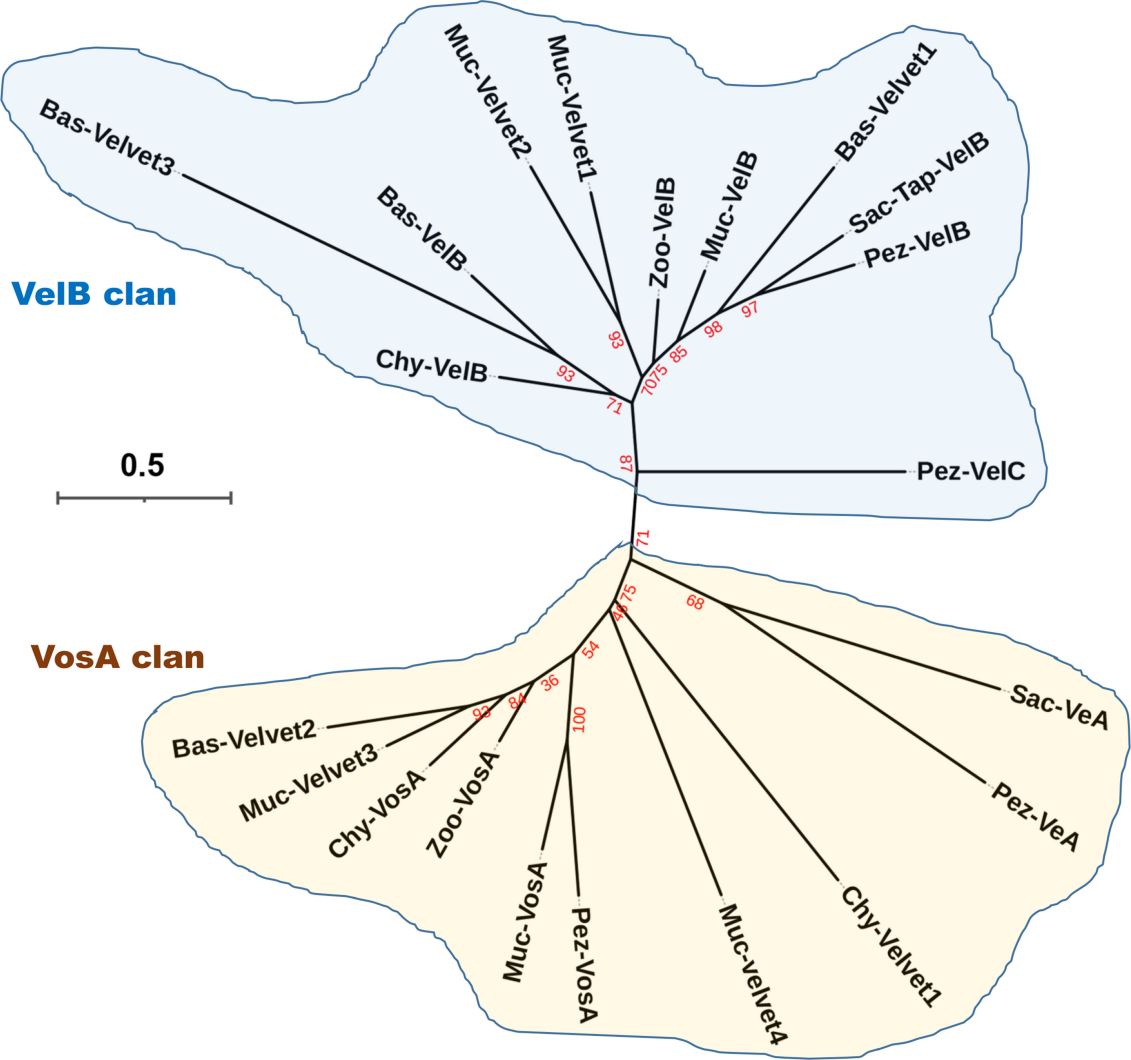
Phylogenetic relationship of the 21 velvet domains based on their consensus sequences. Bootstrap values for each node are highlighted in red.

The phylogenetic relationship of the 21 velvet domains was analyzed based on their consensus sequences (Fig. 18). In phylogeny, there are two major clans (VelB and VosA clans) for the fungal velvet domains. Prediction of the subcellular localization of the 21 velvet domains with their consensus sequences using WoLF PSORT suggested their presence in the nucleus or dual localization shuttling between the cytosol and nucleus, and nuclear export signal (NES) motifs were detected in 12 of 21 velvet domain consensus sequences by NESmapper (Fig. S4). However, no nuclear localization signal (NLS) motif was detected in the 21 consensus sequences by NLStradamus.

### 3D structure modeling and comparison of the 21 velvet domains

The 3D structures of the 21 velvet domains were modeled using AlphaFold 2 with their consensus sequences (Fig. S5). The multiple sequence alignment depth and diversity of the 21 consensus sequences generated by ColabFold (Fig. S6) also suggested that the three characteristic motifs (Fig. 17) are much conserved. The predicted lDDT-Cα score per residue of the 21 consensus sequences was used as a measure of their AlphaFold 2 confidence (Fig. S7). In general, the scores of the conserved N- and C-terminal regions are higher than those of the unconserved middle regions.

**Fig 19:**
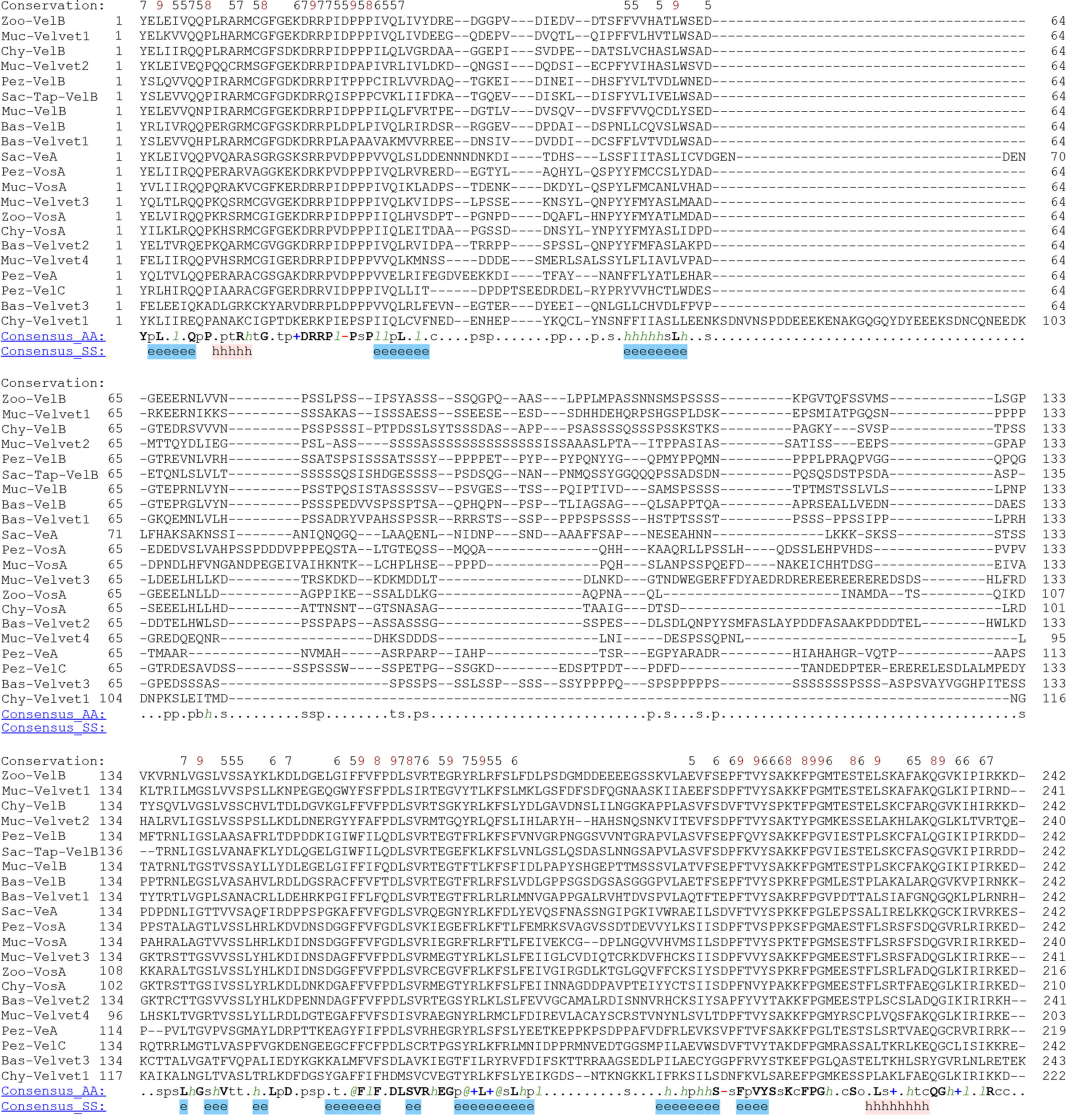
Alignment of the 21 velvet domains based on their secondary structures. The predicted 3D structures of the 21 velvet domains (Fig. **S5**) modeled by AlphaFold 2 with their consensus sequences were submitted to PROMALS3D for structure alignment. Consensus secondary structure (SS) symbols: alpha-helix: h; beta-strand: e. Consensus AA symbols: conserved amino acids are in bold and uppercase letters; aliphatic (**I, V, L**): l; aromatic (**Y, H, W, F**): @; hydrophobic (**W, F, Y, M, L, I, V, A, C, T, H**): h; alcohol (**S, T**): o; polar residues (**D, E, H, K, N, Q, R, S, T**): p; tiny (**A, G, C, S**): t; small (**A, G, C, S, V, N, D, T, *P***): s; bulky residues (**E, F, I, K, L, M, Q, R, W, Y**): b; positively charged (**K, R, H**): +; negatively charged (**D, E**): -; charged (**D, E, K, R, H**): c.

**Fig 20:**
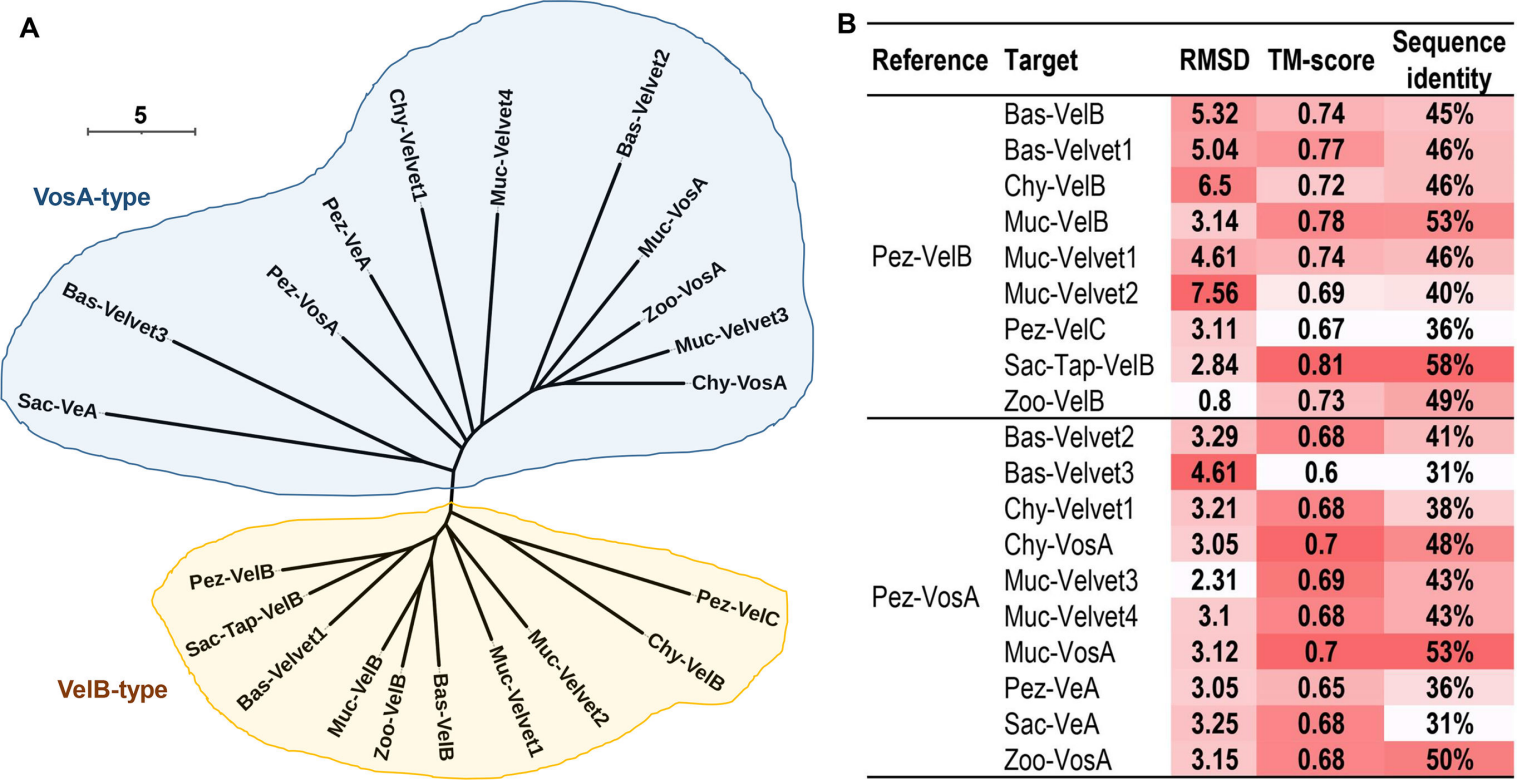
3D structure comparison of the 21 velvet domains. (A) The structural similarity dendrogram of the 21 velvet domains. The 3D structures of the 21 velvet domains were submitted to the Dali server with all against all structure comparison for generating their structural similarity dendrogram. (B) The pairwise structure alignment summary of velvet domains with Pez-VelB and Pez-VosA as references. The detailed comparison was given in Fig. 21. For measuring the alignments, the lower the root mean square deviation (RMSD), the better the structure alignment between the pair of structures. TM-score ranges between 0 and 1, and scores >0.5 generally indicate that the proteins have the same fold (42).

**Fig 21:**
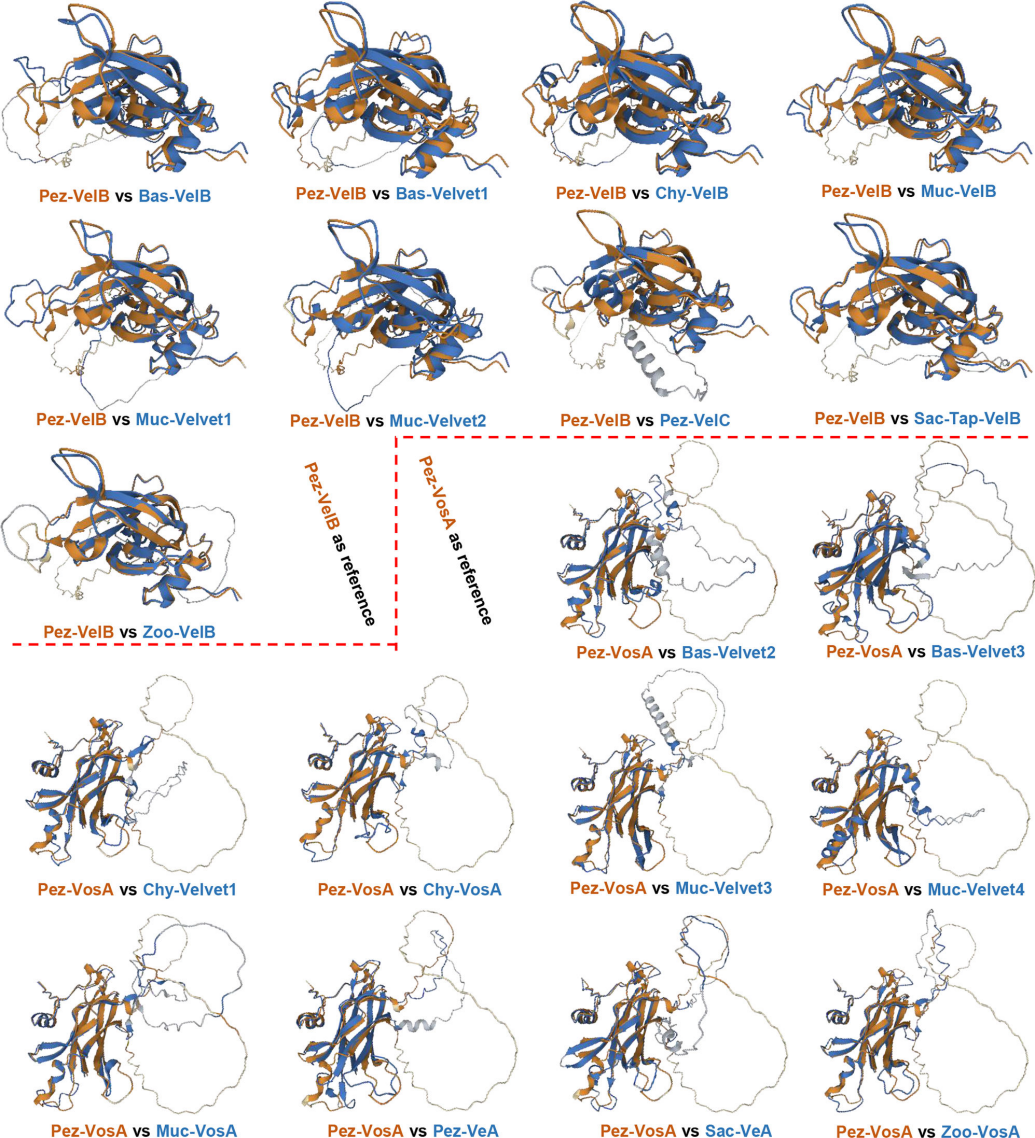
The pairwise structure alignment of velvet domains with Pez-VelB and Pez-VosA as references. The 3D structures of velvet domains were submitted to the Protein Data Bank (PDB) server (https://www.rcsb.org/alignment) for pairwise structure alignment with the jFATCT (rigid) method. The comparison was summarized in Fig. 20B.

The secondary structures of the 21 velvet domains were aligned (Fig. 19). As the results revealed, the three characteristic motifs of velvet domains are conserved not only in their primary sequences but also in their secondary structures. Then, the global structural similarity of the 21 velvet domains was compared and based on the dendrogram (Fig. 20), the 3D structures of the 21 velvet domains could be divided into two types viz., VelB-type and VosA-type. For further determining the topological similarity among the structures within the VelB-type or VosA-type, the pairwise structure alignment was performed with Pez-VelB and Pez-VosA as references (Fig. 20B and 21). As revealed, in general, the N- and C-terminal regions of the velvet domains share a highly similar protein fold, but the middle regions harbor different loops (Fig. 21). Regarding the TM-scores (template modeling scores) between the reference and target structures (Fig. 20B), all scores are greater than the threshold 0.5, which generally indicates that the proteins have the same fold (42). Meanwhile, a certain positive linear correlation was observed between the TM-score and sequence identity (*r* = 0.7793).

### Other functional domains of velvet proteins

It is noted that many velvet proteins are much longer than their velvet domains and may also include other functional domains. Therefore, besides velvet domains, other functional domains of velvet proteins were investigated among the 21 clades (Table 2; Table S1). In general, the distribution of functional domains differs by clade. For example, among the four well-known members in Pezizomycotina, 58 types of functional domains were detected in approximately 58.8% of proteins in the Pez-VeA clade, and approximately 40 types of functional domains were detected in >30% of proteins in the clades Pez-VelC and Pez-VosA. However, probably due to the shorter length, only 11.9% of proteins in the Pez-VelB clade detected functional domains. PHA03247 (large tegument protein UL36) was the most frequent domain found in many clades. In the Muc-Velvet1 clade, 16.7% of velvet proteins contained the domain Glyco_transf_49 (glycosyl-transferase for dystroglycan).

**TABLE 2:** Summary of the detected functional domains among the 21 major clades^*a*^

Clade (protein count)	Percentage with detected domains	Type total of detected domains	Main domains and percentages
Bas-VelB (58)	15.5%	6	PHA03247 (3.4%), PHA03307 (3.4%), PLN02217 (3.4%)
Bas-Velvet1 (542)	34.7%	24	PHA03247 (15.7%), dnaA (4.2%), Herpes_BLLF1 (2.2%)
Bas-Velvet2 (473)	37.2%	36	PHA03247 (14.6%), PRK07764 (5.5%), PRK10263 (2.1%)
Bas-Velvet3 (738)	2.7%	11	PHA03247 (0.7%)
Chy-VelB (119)	5.9%	5	PHA03247 (2.5%)
Chy-Velvet1 (33)	6.1%	2	PRK12678 (3.0%), PTZ00121(3.0%)
Chy-VosA (27)	33.3%	7	PHA03247 (11.1%)
Muc-VelB (372)	7.3%	15	AdoMet_MTases (1.6%), Smc (1.3%)
Muc-Velvet1 (252)	24.6%	15	Glyco_transf_49 (16.7%)
Muc-Velvet2 (44)	4.5%	1	PTZ00173 (4.5%)
Muc-Velvet3 (156)	26.3%	12	PHA03247 (11.5%), PHA03307 (4.5%)
Muc-Velvet4 (55)	0	0	
Muc-VosA (419)	15.3%	17	dnaA (2.9%), PHA03307 (1.9%)
Pez-VeA (1307)	58.8%	58	PHA03247 (26.7%), PRK10263 (11.8%), dnaA (2.8%)
Pez-VelB (1286)	11.9%	28	PHA03247 (2.3%), PABP-1234 (1.7%), PRK10263 (1.7%)
Pez-VelC (1243)	35.2%	37	PHA03247 (15.8%), PRK10263 (6.8%), dnaA (4.4%)
Pez-VosA (1035)	31.4%	42	PHA03247 (7.0%), PTZ00395 (4.4%), PABP-1234 (4.1%)
Sac-Tap-VelB (32)	6.3%	2	DUF5695 (3.1%), PRK14971 (3.1%)
Sac-VeA (31)	6.5%	1	PRK10263 (6.5%)
Zoo-VelB (73)	0	0	
Zoo-VosA (55)	0	0	

^*a*^ The functional domains of each clade were annotated based on the NCBI batch CD-Search (43), and velvet domains were excluded from the results. The detailed list is provided in Table S1.

### Distribution of the velvet proteins outside the fungal kingdom

**Fig 22:**
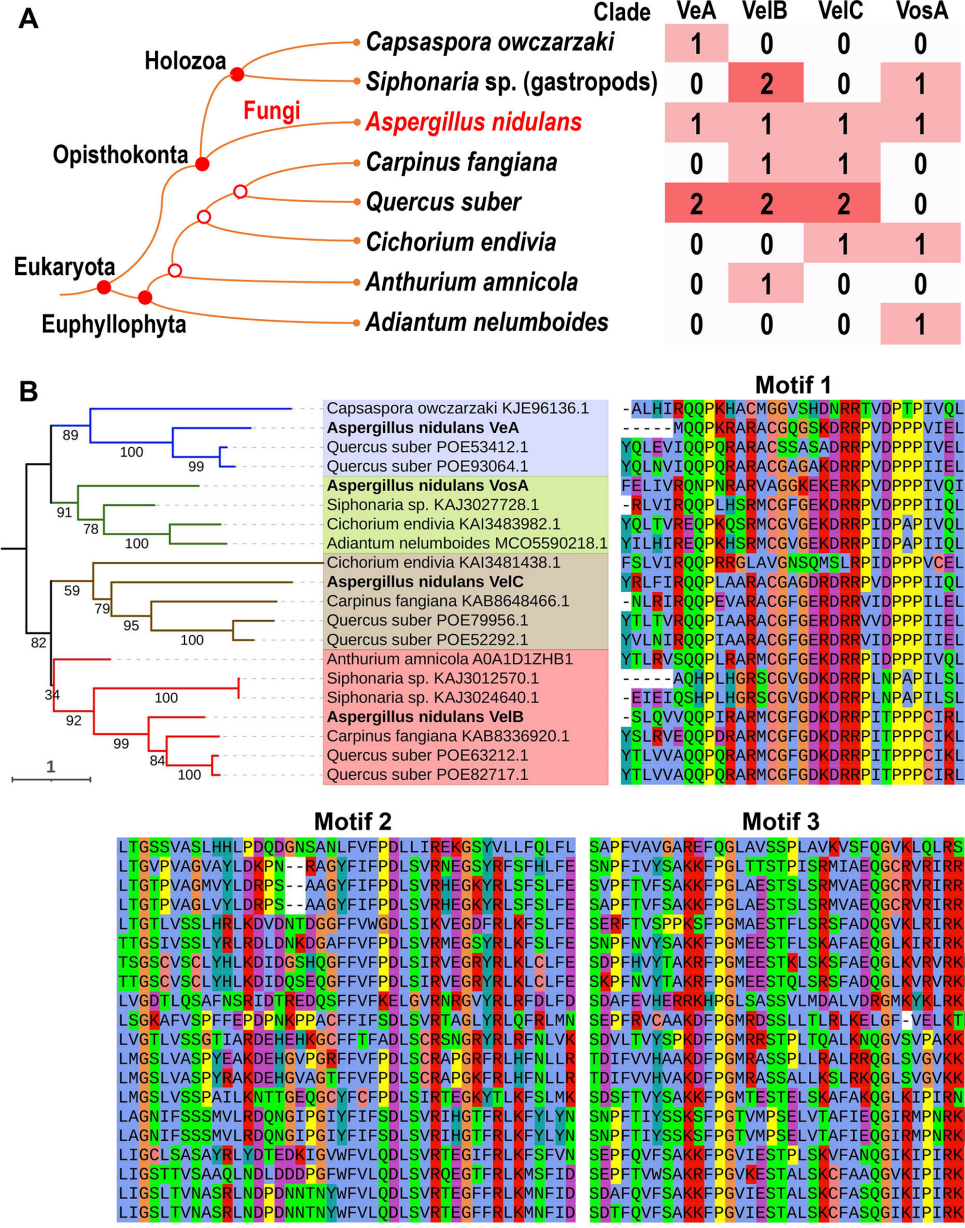
Features of velvet proteins outside the fungal kingdom. (A) A cladogram of species beyond the fungi harboring velvet proteins and their distribution. The taxonomic relationship was based on the NCBI taxonomy database (45), and *A. nidulans* was used as a representative fungus. (B) Phylogenetic relationship of velvet proteins outside the fungal kingdom and their three velvet characteristic motifs. *A. nidulans* VeA, VelB, VelC, and VosA were used as references and highlighted in bold on the tree. The velvet proteins in the same clade are highlighted with the same background color. The residues are colored with the Clustal X default coloring scheme.

The homologs of velvet proteins outside the fungal kingdom were queried on the protein databases of NCBI (https://www.ncbi.nlm.nih.gov/) and UniProt (https://www.uniprot.org/). Results showed that complete velvet domains were also detected outside the fungal kingdom and the proteins were clearly grouped into the clades VeA, VelB, VelC, and VosA (Fig. 22). In Holozoa, a relative branch of fungi under Opisthokonta, two species *Capsaspora owczarzaki* (44) and *Siphonaria* sp. harbor velvet proteins. In Euphyllophyta, velvet proteins were detected in several plant species. Especially in *Quercus suber*, six velvet proteins classified into three clades were detected. Beyond Eukaryota, several homologs of velvet domains have been detected in Archaea and Bacteria, but their velvet domains are not complete and not considered further.

## DISCUSSION

### A possible evolutionary scenario for the velvet family was reconstructed

Velvet proteins were once considered specific for the fungal kingdom (8, 33, 34). According to our results (Fig. 2), velvet proteins are widely distributed in the fungal kingdom. Furthermore, beyond the fungal kingdom, velvet proteins reach as far as Euphyllophyta. However, at present, it was still difficult to infer the earliest occurrence node of velvet proteins. It was not quite sure whether velvet members of Euphyllophyta are indigenous genes or originated via horizontal gene transfer or contaminated by fungal DNA. Based on the BlastP analysis in the NCBI database, the Euphyllophyta velvet proteins exhibit the closest similarity to their Ascomycete counterparts.

In the fungal kingdom, 21 major clades were classified in this study. Along the evolutionary course of the fungal kingdom, the velvet family underwent gene loss, duplication, and divergence, resulting in its diversification. For instance, most Saccharomycotina genomes showed no *velvet* genes (Fig. 2), but the Saccharomycotina ancestor should have contained *velvet* genes and subsequently lost them. Most Mucoromycotina genomes contained more than 10 *velvet* genes (Fig. 3), and based on their phylogenetic relationship (Fig. 11), the multiple *velvet* genes probably originated via gene duplications. In general, velvet types vary in different fungal taxa. Based on the phylogenetic analysis, VelB and VosA clades may be very ancient in the fungal kingdom due to their wide presence. The VeA clade appears Ascomycota-specific. The VelD branch of some *Aspergillus* spp. belongs to the Pez-VosA clade, suggesting that VelD is a variant of Pez-VosA. VelD may have originated from a duplication of *vosA* in the ancestor of *Aspergillus* section Flavi, and subsequently diverged with VosA. The VelC clade is flourishing in Ascomycota, but it also presented outside Ascomycota, suggesting its possible earlier presence than Ascomycota.

Velvet proteins are generally constituted by a velvet domain and optional other regions. Based on our results (Table 2), the additional regions of velvet proteins (excluding the velvet domain region) could harbor various functional domains. In other words, the velvet domain could be in the N-terminal side, middle, or C-terminal side and combine with various functional domains to form various velvet proteins, resulting in their diversification in the fungal kingdom. This evolutionary plasticity could serve the specific biological requirements of different fungi adapted to their respective habitats.

**Fig 23:**
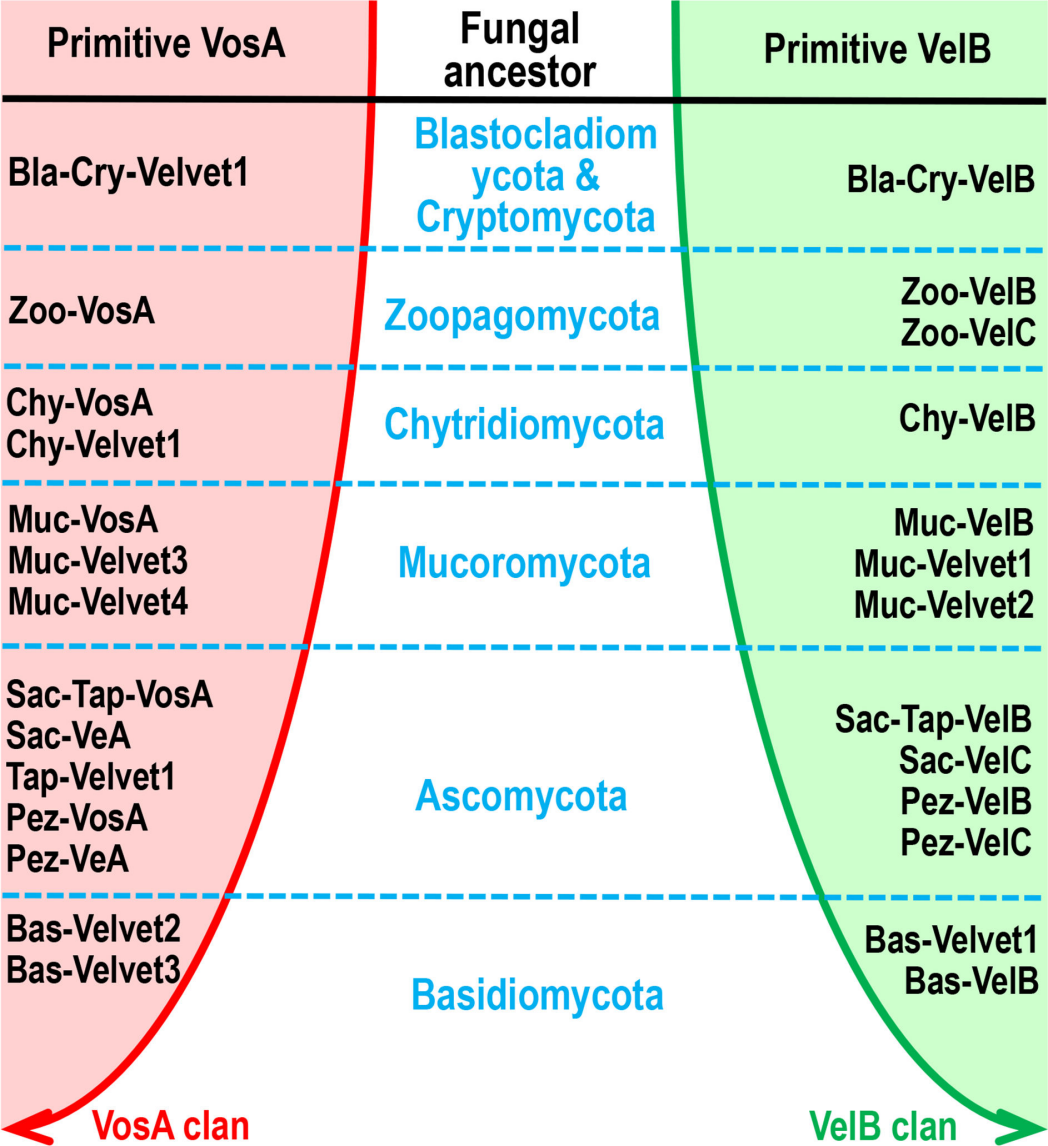
Two evolutionary clans of velvet clades in the fungal kingdom. The phylogenetic relationship of fungal velvet clades was based on their velvet domains.

In view of the fact that the conserved velvet domain is shared by the entire velvet family, the velvet domains could be used for tracking their long-term evolution. Therefore, the phylogenetic relationship among fungal velvet clades was analyzed based on their velvet domain primary sequences (Fig. 18) and 3D structures (Fig. 20A). The results of both analyses indicated that the current fungal velvet domains are clearly classified into two clans (named VelB and VosA clans). The list of VelB and VosA clans across the fungal kingdom is summarized in Fig. 23. It shows that all the tested phyla contain members of the VelB and VosA clans. To summarize, primitive VelB and VosA may have existed in the fungal ancestor. Along with the expansion of the fungal kingdom, these two clans were expanding out various velvet clades (Fig. 23).

### The velvet domain is constituted by three characteristic motifs

It is widely recognized that velvet proteins are diverse but share a highly conserved velvet domain (8). The Pfam database (https://www.ebi.ac.uk/interpro/entry/pfam/PF11754/) provides a velvet domain model with 243 characterized residues. Based on the statistics of velvet domain lengths among different clades (Fig. 16), most velvet domains are of approximate 200 AAs, but some can be even of more than 500 AAs. In this study, all the tested velvet proteins carried only one single velvet domain.

The primary residues of velvet domains were compared among the 21 major fungal velvet clades (Fig. 17 and Fig. S4). Obviously, the three conserved characteristic motifs were found in the velvet domains across different clades. The secondary and 3D structures of the 21 velvet domains were also compared (Fig. 19 to 21). As the results revealed, the three characteristic motifs of velvet domains are conserved not only in their primary sequences but also in their secondary and 3D structures. In other words, these three characteristic motifs constitute the basic skeleton of velvet domains and are probably related to their general functions.

For example, *in vivo* and *in vitro* analyses of the *A. nidulans* VosA velvet domain revealed that the motif 1 region is involved in DNA-binding and several positively charged residues (Lys, Arg) are susceptible to DNA-binding activity (4). As shown, the positively charged residues (Lys, Arg) frequently occupy the 13th, 20th, 22nd, and 23rd positions of motif 1 across different velvet domains (Fig. 17). Currently, much remains to be elucidated regarding the relationship between velvet structure and function, and an analysis of conserved residues or motifs could direct the functional analysis of velvet domains.

### The velvet domain exhibits a structural similarity to many DNA-binding proteins

To date, the crystal structures of the VosA homodimer and VosA-VelB complex from *A. nidulans* have been characterized and the comparison revealed an unexpected structural similarity of the velvet domain with the Rel homology domain of the mammalian transcription factor NF-κB (4). Therefore, we searched the available proteins with 3D structures to examine the significant hits with structural similarities to the velvet domain. In the database of Protein Data Bank (PDB) (https://www.rcsb.org/), hundreds of structurally similar proteins were found using the Dali server by querying the VosA velvet domain (PDB ID: 4N6Q chain A) (Table S2). In the NCBI protein structure database (https://www.ncbi.nlm.nih.gov/Structure/), 132 similar structures of the VosA velvet domain were found using the Vector Alignment Search Tool Plus (VAST+) (Table S3).

**Fig 24:**
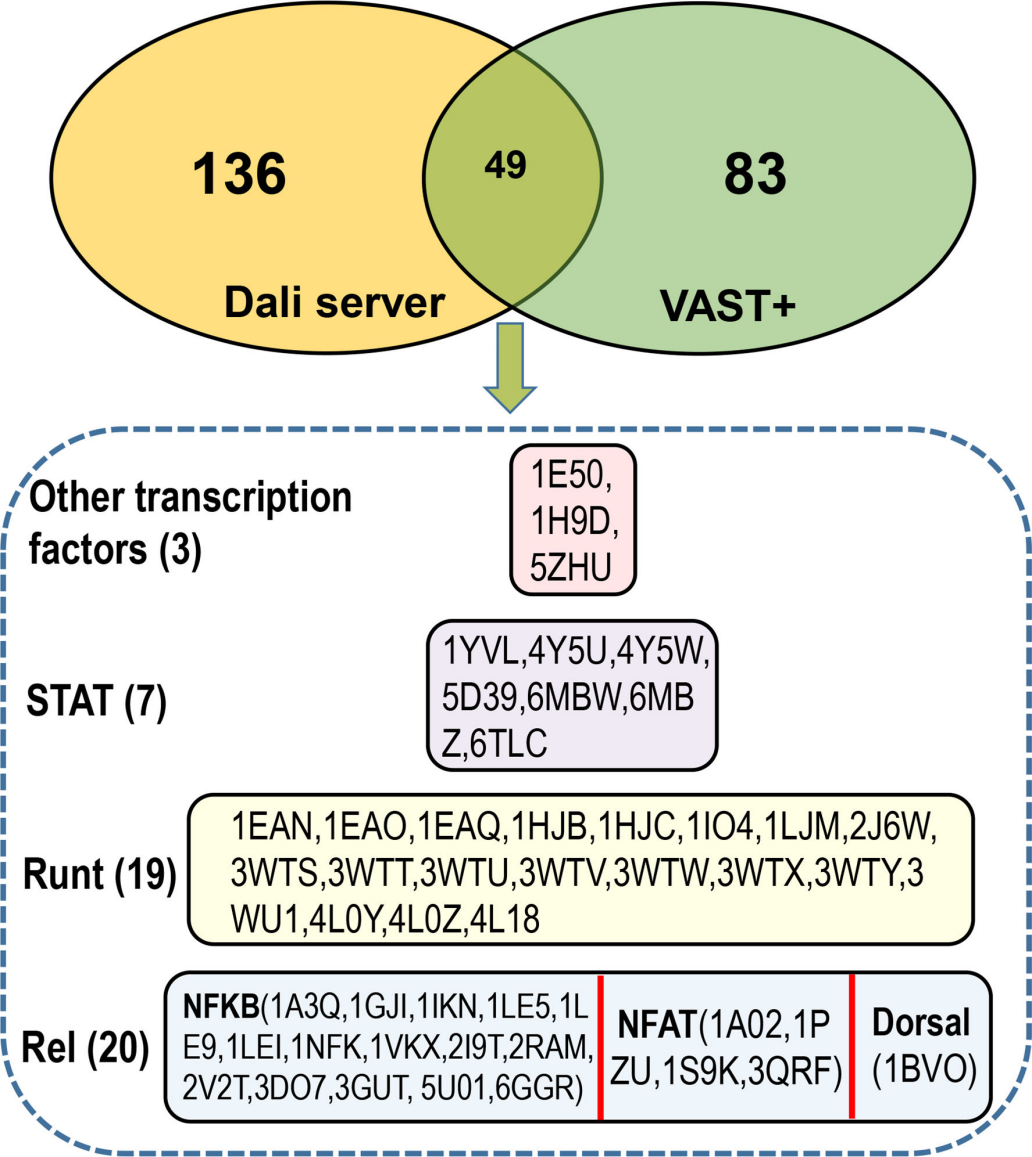
Comparison of structurally similar proteins of the VosA velvet domain by the Dali server and VAST+. The detailed lists of structurally similar proteins by the Dali server and the VAST+ analysis are provided in Table S2 and S3. The upper part is a Venn diagram between the Dali server analysis and the VAST+ analysis. The lower part is a detailed list of the 49 shared proteins by the Dali server and the VAST+ analysis. The PDB IDs are listed inside the box.

The lists of structurally similar proteins were compared between the Dali server and the VAST+ analysis (Fig. 24). The 49 shared proteins were primarily classified into three types of DNA-binding domains (Fig. 24). Consistent with a previous report (4), 20 Rel homology domains from a wide variety of eukaryotic transcription factors such as NF-κB, dorsal, and nuclear factor of activated T-cells (46, 47) were found to have similar 3D structures with the VosA velvet domain. Other remarkable hits included 19 Runx1 Runt domains and 7 DNA-binding domains of the STAT proteins.

Similar to velvet proteins, these three types of transcription factors are also large families with various members that can form different dimers and play diverse roles in the regulation of cellular functions (3, 48, 49). In the database of InterPro (50), the DNA-binding domains of the Rel, Runt, and STAT families belong to the same β-sandwich type superfamily (https://www.ebi.ac.uk/interpro/entry/InterPro/IPR008967/). Therefore, the velvet domain with a common β-sandwich fold (4) should also belong to this DNA-binding domain superfamily. However, they may be not phylogenetically related because of their low amino acid sequence similarities with sequence identities ranging from 9% to 22 %. Probably, the shared 3D structure similarity among the velvet domain and other DNA-binding domains originated from the directed structure convergent evolution in their long-term independent interaction with DNA.

In summary, we conducted a taxonomically broad survey of velvet proteins in the fungal kingdom and beyond to reveal their distribution, protein size, and domain architecture. We then grouped the 21 major clades of velvet proteins in fungi based on the phylogenetic analysis and compared their conserved motifs and 3D structures. Altogether, our results suggest that velvet proteins are widely distributed in the fungal kingdom but also outside the kingdom. The velvet domain is highly conserved with three characteristic motifs and could combine with different functional domains, resulting in the diversity of velvet proteins. By analyzing the primary and 3D structures of various velvet domains across the fungal kingdom, we found that fungal velvet domains can be divided into two clans (VelB clan and VosA clan). Based on the structural comparison, we proposed that the velvet domain, together with the DNA-binding domains of the Rel, Runt, and STAT families sharing a similar β-sandwich fold, should belong to the same DNA-binding domain superfamily.

## MATERIALS AND METHODS

### Species selection and their sequence data

In this study, to address the diversity of velvet proteins in the fungal kingdom, the fungal genomic database of MycoCosm (https://mycocosm.jgi.doe.gov/mycocosm/home) (38) (2384 fungal genomes, accessed on 5 December 2022) covering the phyla Ascomycota, Basidiomycota, Blastocladiomycota, Chytridiomycota, Cryptomycota, Microsporidia, Mucoromycota, and Zoopagomycota was queried. And then, the gene catalog proteins of these fungal species/strains were used for surveying the velvet distribution on the genomic scale.

The protein sequence databases of NCBI (51) and UniProt (52) were also accessed for searching velvet homologs outside the fungal kingdom.

### Identification and annotation of velvet proteins

Velvet proteins are diverse but share a common and conserved velvet domain (8). Therefore, the rule for the identification of Velvet proteins is whether they contain velvet domains. In this study, the *A. nidulans* VeA velvet domain [position 34-231 of VeA protein GenBank: AAD42946.1 (13)] was used as a query to search for the homologs in the databases MycoCosm, NCBI, and UniProt by BlastP (53) with default parameters. Candidates close to the threshold were further confirmed in the NCBI conserved domain database (https://www.ncbi.nlm.nih.gov/Structure/cdd/wrpsb.cgi) (43) for validating whether they hold full velvet domains and those with incomplete velvet domains were filtered. This approach is simple but highly effective because the velvet domain is highly conserved and unique and displays no sequence similarity to other known domains.

For annotation of the velvet candidates, they, together with the references *A. nidulans* VeA (GenBank: AAD42946.1), VelB (GenBank: ABQ17967.1), VelC (GenBank: ABQ17968.1), and VosA (GenBank: ABI51618.1), were subjected to phylogenetic analysis. The putative velvet proteins were classified into different velvet clades based on their phylogenetic relationship. The clades were named as the species taxonomic group followed by the velvet name. The clade taxonomic group is named by the first three letters of corresponding species taxonomic name. When a velvet clade contained a reference velvet, the clade was assigned to the reference velvet name. When there was a clear distinction between a velvet clade and the four references in phylogeny, the clade was assigned to a new member named as Velvet1, Velvet2, etc.

### Phylogenetic analysis of velvet proteins or domains

The phylogenetic analysis was performed as follows. First, multiple alignments of the velvet proteins or domains were carried out by the MAFFT online service with its default parameters (54). Second, the multiple alignments were used to infer their trees. When the alignments had less than 200 sequences, they were submitted to the IQ-TREE web server (http://iqtree.cibiv.univie.ac.at/) for estimating the maximum likelihood tree with the best-fit model (55). When the alignments had more than 200 sequences, they were submitted to the T-REX web server (http://www.trex.uqam.ca/) for inferring phylogenetic trees with the best-fit method (56). Finally, the figures of phylogenetic trees were edited and generated by iTOL (https://itol.embl.de/) (57).

### Analysis of characteristic motifs and residues of velvet domains

The characteristic motifs of the velvet domains were compared among different velvet clades to reveal their clade-shared or -specific conserved residues. First, multiple alignments of protein sequences within each clade were constructed using HMMER 3.1 (http://hmmer.org/) against the profile velvet hidden Markov model (https://www.ebi.ac.uk/interpro/entry/pfam/PF11754/) in InterPro (50). The residues assigned to match states that were conserved against the profile velvet hidden Markov model were reserved for constructing their consensus sequence logos.

Then, the consensus logos were generated from the alignments by WebLogo 3.7.12 (http://weblogo.threeplusone.com/create.cgi) for visualization of the conservation of the primary structure by plotting a stack of amino acids for each position (58, 59). The evolutionary conservation of each amino acid position in the alignment was determined using the ConSurf web-server (60, 61).

### Survey of functional domains and motifs among velvet proteins

Putative functional domains of velvet proteins were surveyed among different velvet clades. The protein sequences were submitted to the NCBI conserved domain database (https://www.ncbi.nlm.nih.gov/Structure/bwrpsb/bwrpsb.cgi) to annotate their functional domains (43).

Furthermore, NES of velvet domains were predicted using NESmapper 1.1 (62), and their NLS were mined using NLStradamus (63). Prediction of the subcellular localization of velvet domains was performed using WoLF PSORT targeting fungal species (64).

### 3D structure modeling and comparison

The 3D structure modeling of velvet domains was accomplished using AlphaFold v2.3.1 (65) pipelined in ColabFold v1.5.2 (66). Then, the 3D structures were displayed by the viewer Jmol (www.jmol.org). The comparison of protein structures was performed by PROMALS3D (67), the Dali server (68), and the Pairwise Structure Alignment server of PDB (https://www.rcsb.org/alignment). The search for similar structures of velvet domains against the database of Protein Data Bank (https://www.rcsb.org/) was performed in the Dali server (68) with the query structure of VosA velvet domain chain A (PDB ID: 4N6Q) (4). Meanwhile, the VosA velvet domain was also used as a query to search for similar structures in the NCBI protein structure database (https://www.ncbi.nlm.nih.gov/Structure/) using the Vector Alignment Search Tool Plus (69).

### Statistical analysis

Boxplots of lengths of velvet proteins or domains were generated using OriginPro 8 (Massachusetts, USA) or the BoxPlotR (41). Two-group comparisons were performed using the unpaired *t*-test. *P*-values less than 0.05 were considered statistically significant, and those less than 0.001 were considered statistically highly significant.

## SUPPLEMENTAL MATERIAL

Supplemental figures spectrum.03717-23-s0001.pdf Figures S1 to S7.

Table S1 spectrum.03717-23-s0002.pdf Summary of functional domains in the 21 velvet clades.

Table S2 spectrum.03717-23-s0003.pdf Top 300 similar structures of VosA velvet domain in the PDB database by the Dali server.

Table S3 spectrum.03717-23-s0004.pdf Similar structures of VosA velvet domain in the 3D macromolecular structures database of NCBI by VAST+.

ASM does not own the copyrights to Supplemental Material that may be linked to, or accessed through, an article. The authors have granted ASM a non-exclusive, world-wide license to publish the Supplemental Material files. Please contact the corresponding author directly for reuse.
