# Supplementary material for: Phylogenomics analysis of velvet regulators in the fungal kingdom
Source: Microbiol Spectr. 2024 Jan 5;12(2):e03717-23. doi: 10.1128/spectrum.03717-23 (PMC10845976; doi:10.1128/spectrum.03717-23)
Supplement: Supplemental figures — Figures S1 to S7. [file spectrum.03717-23-s0001.pdf]

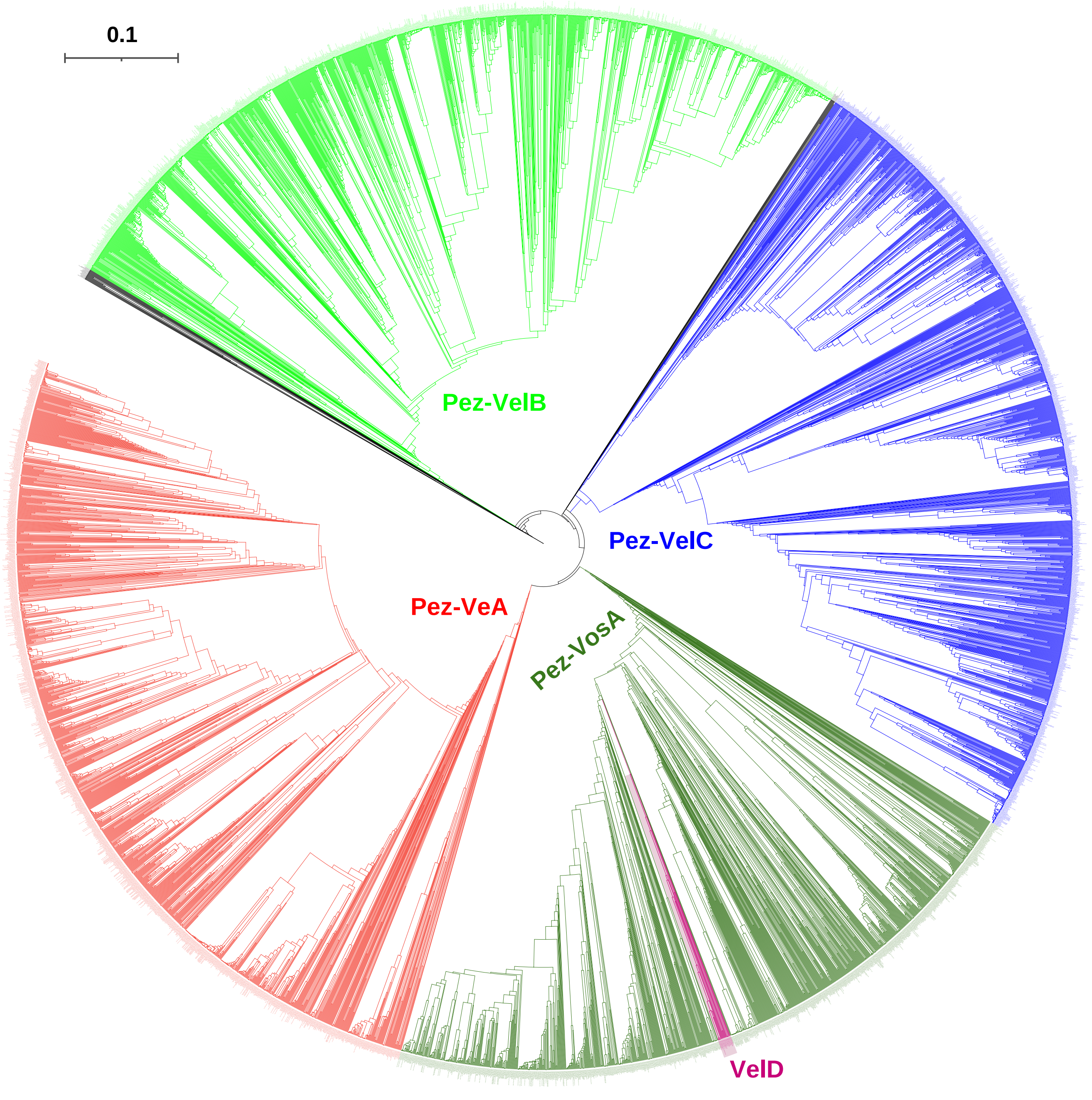

Fig. S1. Phylogenetic relationship of the Pezizomycotina velvet proteins. The figure is a high definition version of **Fig. 6** in the maintext.

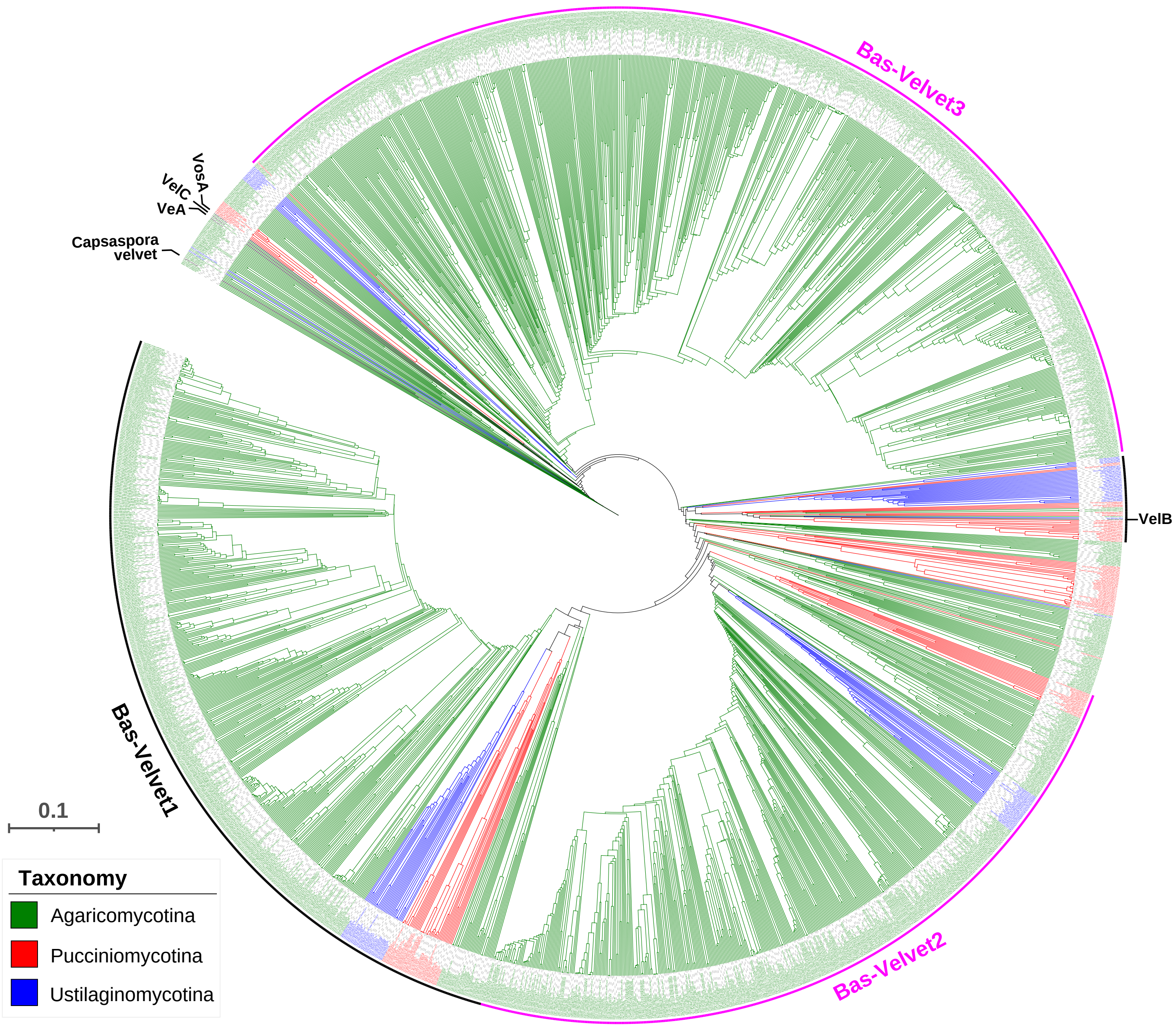

Fig. S2. Phylogenetic relationship of the Basidiomycota velvet proteins. The figure is a high definition version of **Fig. 9** in the maintext.

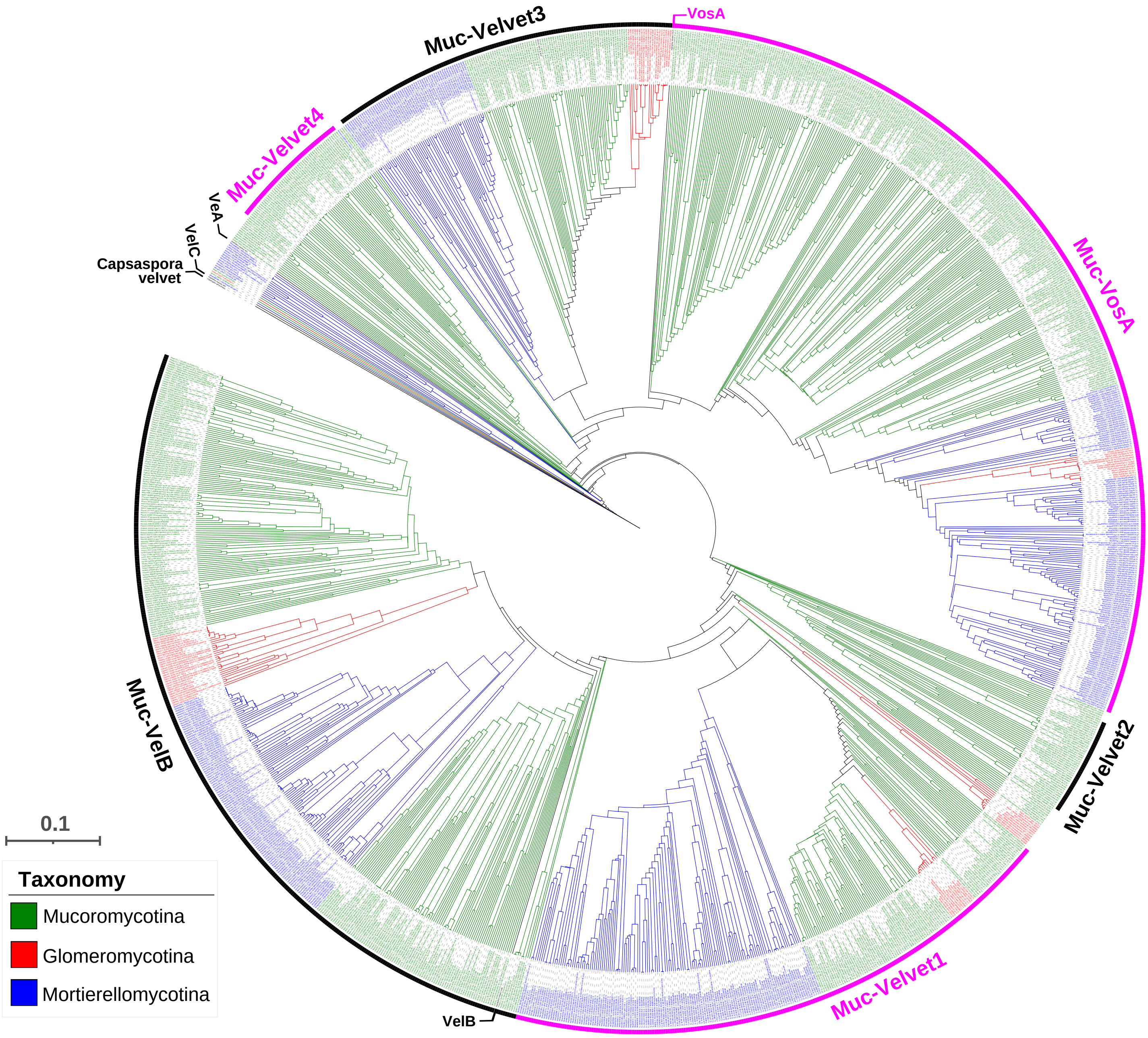

Fig. S3. Phylogenetic relationship of the Mucoromycota velvet proteins. The figure is a high definition version of **Fig. 11** in the maintext.

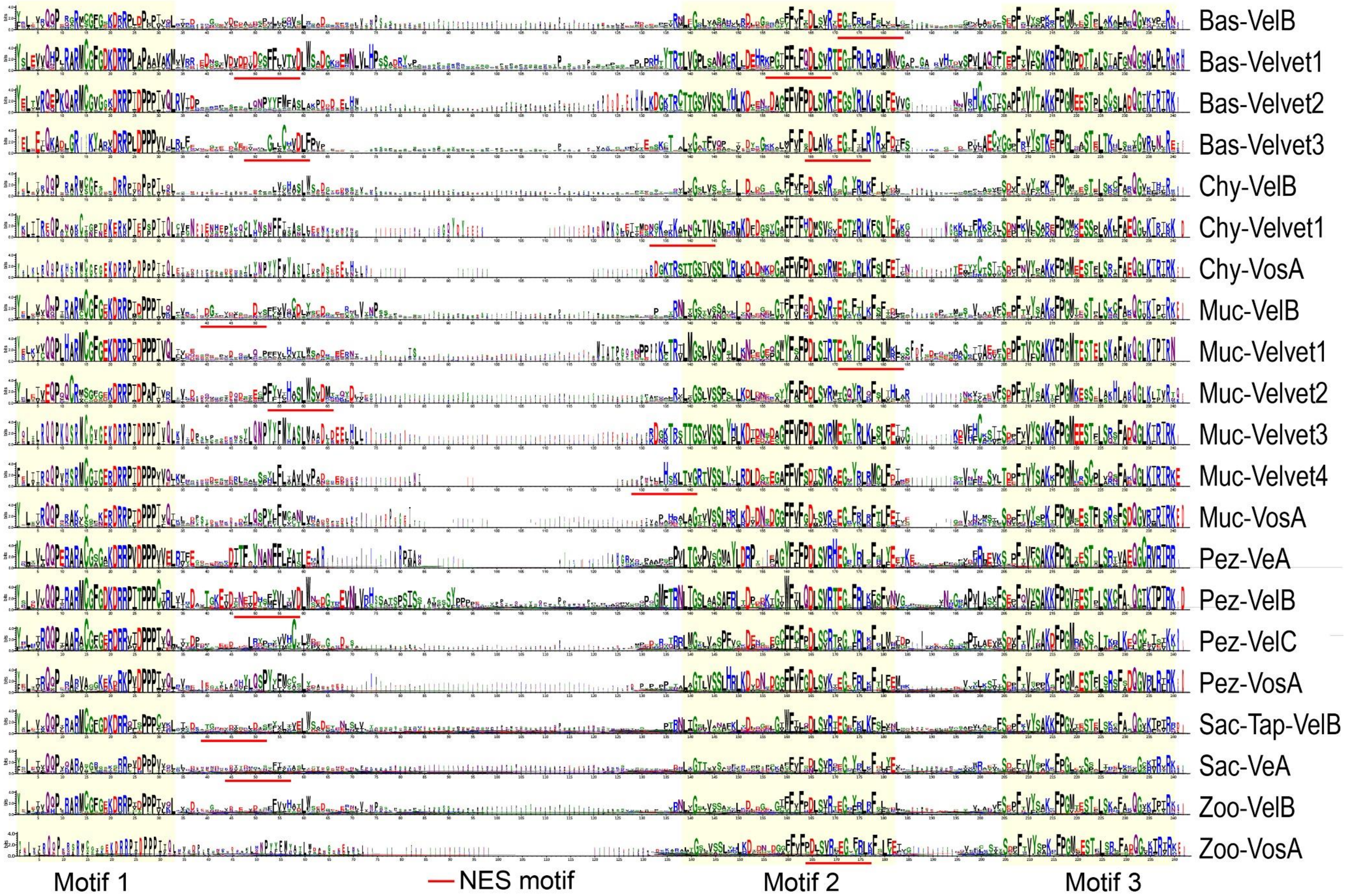

Fig. S4. Sequence logo of velvet domains of the 21 major clades. The alignment of velvet domains was performed by against the profile hidden Markov model of velvet domain PF11754 with 243 residues (https://www.ebi.ac.uk/interpro/entry/pfam/PF11754/). The three characteristic motifs are highlight in yellow and also present in Fig. 17. The predicted NES motifs are underlined by red lines

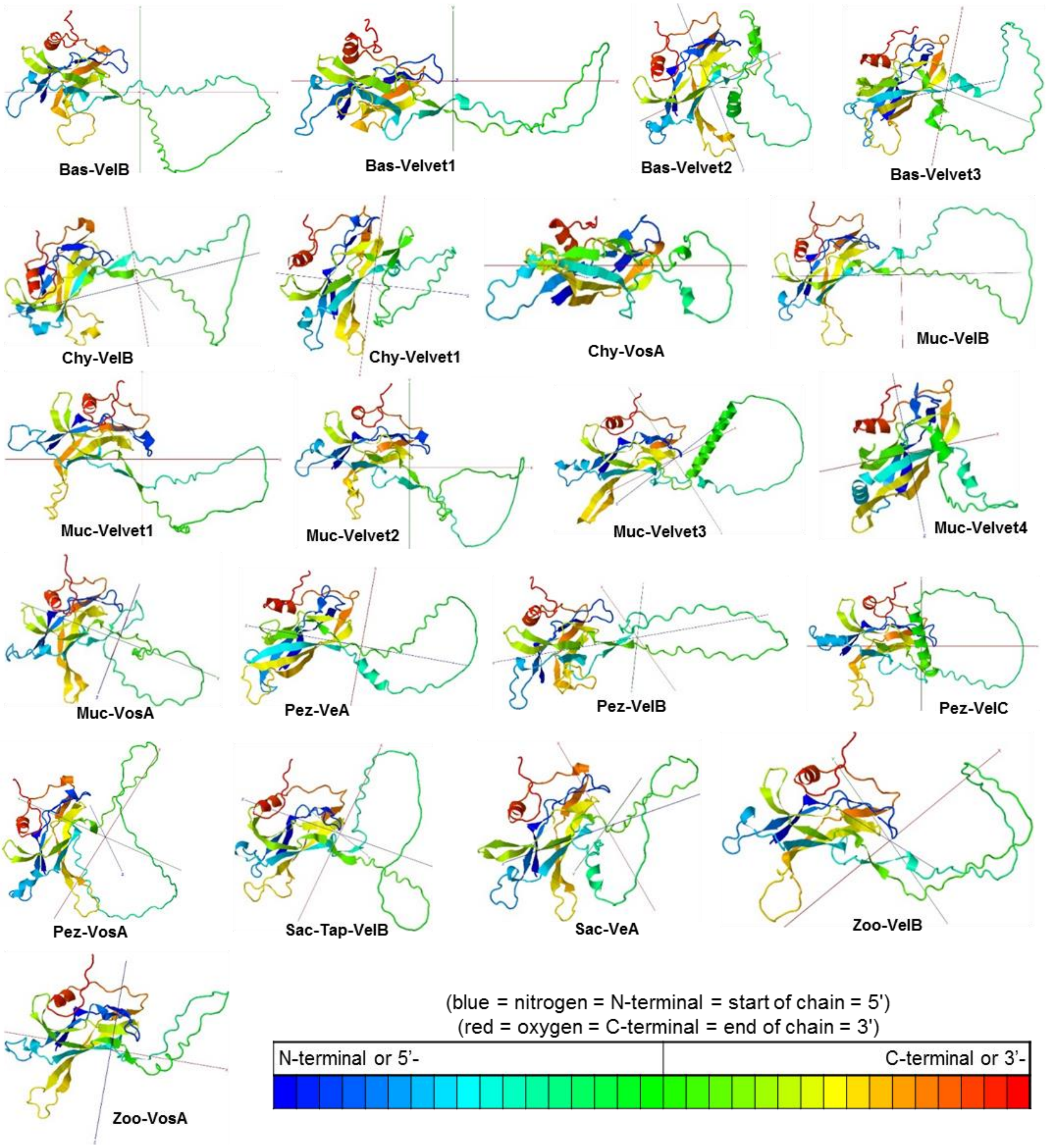

Fig. S5. 3D structures of the 21 velvet domains modelled by AlphaFold 2. Consensus sequences of velvet domains of the 21 clades revealed in Fig. S4 were used for AlphaFold modelling and the top-ranked 3D structure figures were outputted by Jmol. A reverse rainbow gradient (bgyor) as below is used to color according to position of the corresponding residues along a chain.

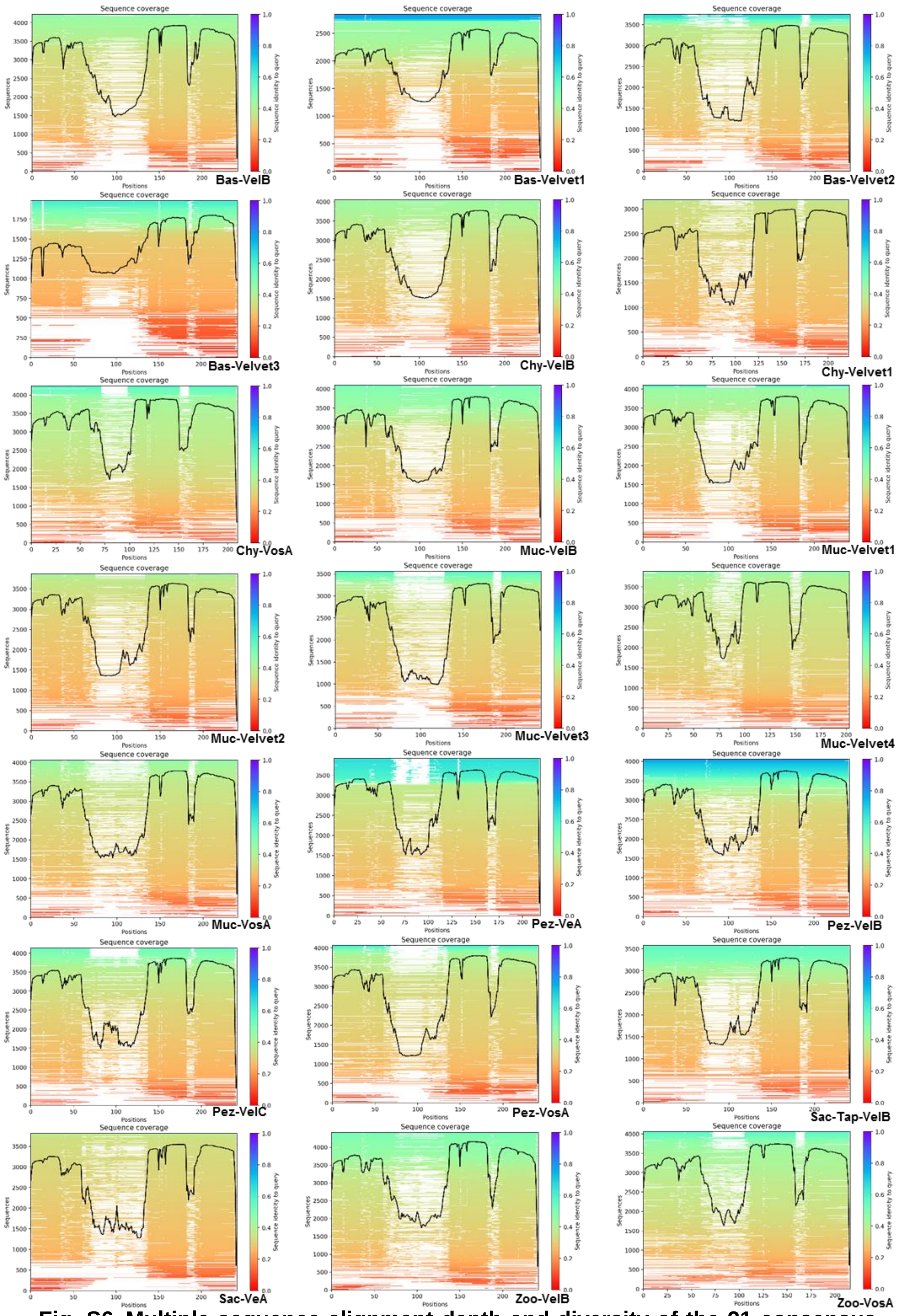

Fig. S6. Multiple sequence alignment depth and diversity of the 21 consensus sequences generated by the ColabFold.

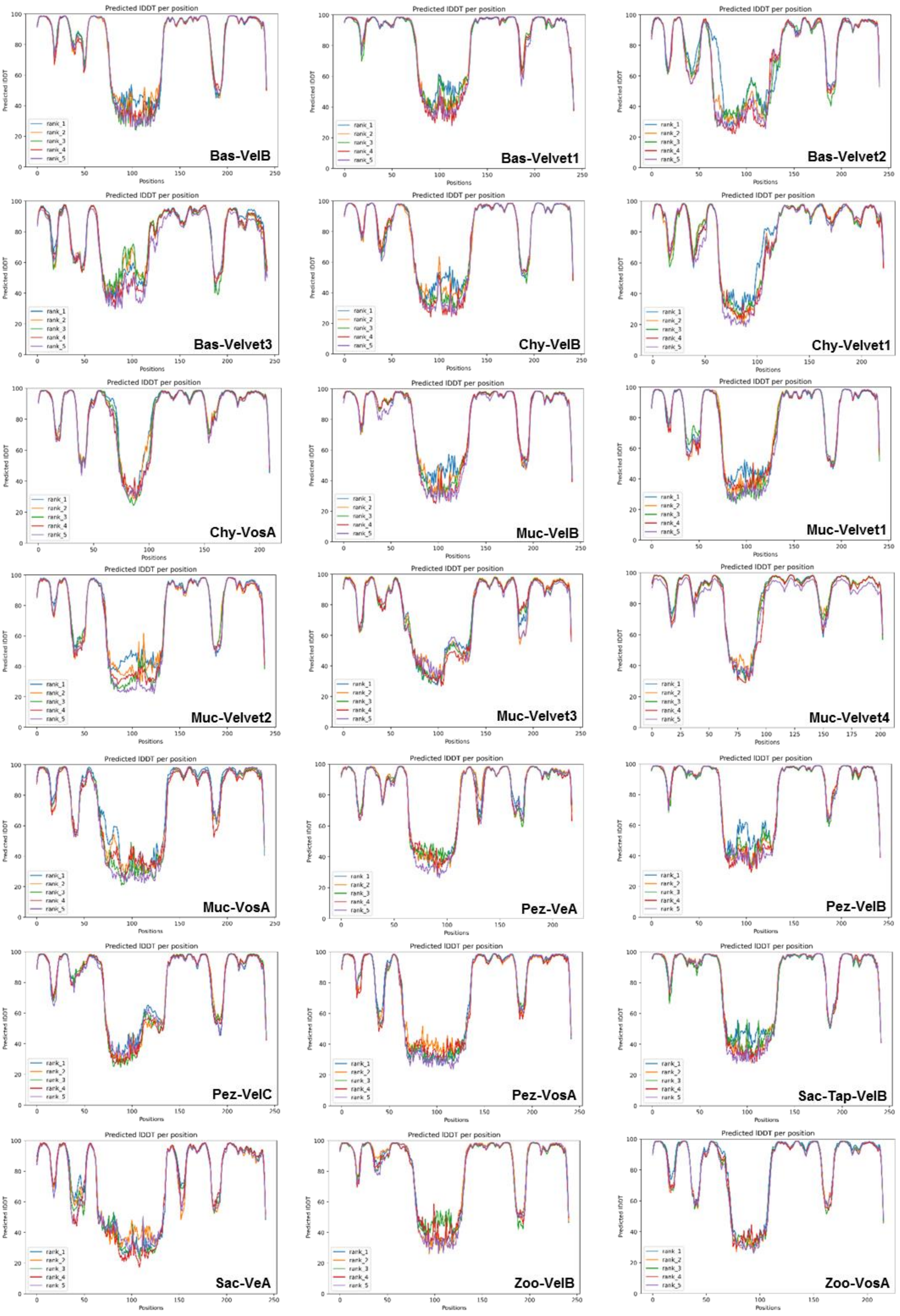

Fig. S7. The predicted lDDT-Cα score (pLDDT) per residue of the 21 consensus sequences to show their AlphaFold2 confidence measures. pLDDT is between 0

and 100. Model confidence: very high (pLDDT>90), confident (90 > pLDDT > 70), low (70 > pLDDT > 50) and very low (pLDDT < 50).
