# Supplementary material for: Phylogenomics analysis of velvet regulators in the fungal kingdom
Source: Microbiol Spectr. 2024 Jan 5;12(2):e03717-23. doi: 10.1128/spectrum.03717-23 (PMC10845976; doi:10.1128/spectrum.03717-23)
Supplement: Table S1 — Summary of functional domains in the 21 velvet clades. [file spectrum.03717-23-s0002.pdf]

Table S1.Summary of functional domains in the 21 velvet clades. The predicted functional domains except velvet domains of velvet proteins are listed

Clade	Query	Hit type	PSSM-ID	From	To	E-Value	Bitscore	Accession	Short name
Bas-VelB	Naifl1_59590_CE59589_2111	superfamily	223021	305	686	1.35E-05	48.7813	cl33720	PHA03247 superfamily
	Tilco1_3729_transcript_OAJ30042	superfamily	223021	1	276	0.003518	39.9217	cl33720	PHA03247 superfamily
	Psean1_1_83181_PANT_18c00055	superfamily	223039	61	299	0.002799	40.5397	cl33723	PHA03307 superfamily
	Tilwal1_7704_transcript_OAJ18296	superfamily	223039	24	221	0.002256	40.5397	cl33723	PHA03307 superfamily
	Tilco1_3729_transcript_OAJ30042	superfamily	215130	85	197	9.54E-05	44.6948	cl33436	PLN02217 superfamily
	Tilcar1_5938_transcript_OAJ15607	superfamily	215130	90	197	0.000239	43.5392	cl33436	PLN02217 superfamily
	Uroocc1_210673_gm1_3072_g	superfamily	237057	4	209	0.003188	39.4739	cl36108	PRK12323 superfamily
	Naifl1_59590_CE59589_2111	superfamily	185594	225	373	0.006251	40.0584	cl33180	PTZ00395 superfamily
	Mixos1_95822_fgenesh1_pm_8_147	specific	238958	159	457	1E-109	340.242	cd02000	TPP_E1_PDC_ADC_BCADC
Bas-Velvet1	Fomro1_992371_e_gw1_56_80_1	superfamily	427171	130	254	0.000985	42.061	cl38111	Atrophin-1 superfamily
	Kocim1_651730_gm1_4356_g	superfamily	455732	278	371	0.000127	41.904	cl42387	dermokine superfamily
	Trich1_6062_CE6061_4679	superfamily	455861	256	425	0.000698	42.5052	cl42516	dnaA superfamily
	Crycu1_388151_estExt_fgenesh1_pg_C_1_t30268	superfamily	455861	258	462	1.28E-05	48.2832	cl42516	dnaA superfamily
	Triol1_103035_CE103034_993	superfamily	455861	253	457	1.26E-05	48.2832	cl42516	dnaA superfamily
	Rhitru1_810645_e_gw1_2_1846_1	superfamily	455861	123	337	0.000202	44.046	cl42516	dnaA superfamily
	Crudry1_2802273_fgenesh1_pg_27_42	superfamily	455861	157	295	2.29E-05	47.1276	cl42516	dnaA superfamily
	SuiFC429_1_1170256_e_gw1_21_9_1	superfamily	455861	116	322	0.003929	39.8088	cl42516	dnaA superfamily
	Amath1_75034_fgenesh1_pm_21_29	superfamily	455861	159	333	0.006699	39.0384	cl42516	dnaA superfamily
	Suisub1_1272999_e_gw1_18_152_1	superfamily	455861	128	321	0.004524	39.4236	cl42516	dnaA superfamily
	Suisubl1_1565023_e_gw1_1_699_1	superfamily	455861	116	322	0.006542	39.0384	cl42516	dnaA superfamily
	Suilak1_1625909_e_gw1_2_1841_1	superfamily	455861	116	322	0.001273	41.3496	cl42516	dnaA superfamily
	Rhisa1_724461_e_gw1_6_176_1	superfamily	455861	123	279	0.002764	40.194	cl42516	dnaA superfamily
	Suiame1_1069328_MIX49214_1965_32	superfamily	455861	129	322	0.000467	42.5052	cl42516	dnaA superfamily
	Tripop1_2534769_gm1_2547_g	superfamily	455861	109	265	0.001115	41.3496	cl42516	dnaA superfamily
	LacbiCham3_1_103280_fgenesh1_pg_33_11	superfamily	455861	115	349	4.37E-07	52.5204	cl42516	dnaA superfamily
	LacbiD101_1_897221_MIX48834_1631_39	superfamily	455861	115	304	8.02E-07	51.3648	cl42516	dnaA superfamily
	LacbiN203_1_634321_e_gw1_5_142_1	superfamily	455861	115	349	1.35E-06	50.9796	cl42516	dnaA superfamily
	LacbiS238O_1_124852_fgenesh1_pg_2_139	superfamily	455861	115	349	4.66E-06	49.0536	cl42516	dnaA superfamily
	Lacbi59489_1_920977_e_gw1_39_65_1	superfamily	455861	115	349	3.94E-07	52.5204	cl42516	dnaA superfamily
	Suiocc1_958472_gm1_11842_g	superfamily	455861	116	323	0.008864	38.6532	cl42516	dnaA superfamily
	Fishe1_72812_gm1_4506_g	superfamily	455861	98	299	0.001253	40.9644	cl42516	dnaA superfamily
	Ustbr1_4655_transcript_SAM77169	superfamily	455861	18	139	0.000469	42.5052	cl42516	dnaA superfamily
	Spore1_3185_sr12445m_01	superfamily	455861	2	146	0.008143	38.6532	cl42516	dnaA superfamily
	Usthor1_81026_UHOR_01729	superfamily	455861	18	139	0.000171	44.046	cl42516	dnaA superfamily
	SerlaS7_3_2_86143_estExt_Genewise1_C_41262	superfamily	282904	112	368	0.000784	42.2108	cl37540	Herpes_BLLF1 superfamily
	Denbi1_958870_estExt_Genemark1_C_1_t10159	superfamily	282904	108	378	0.00367	39.8996	cl37540	Herpes_BLLF1 superfamily
	Anoalb1_2246155_MIX1989_8492_70	superfamily	282904	109	349	7.24E-05	45.6776	cl37540	Herpes_BLLF1 superfamily
	Plucer1_950568_MIX42980_1143_47	superfamily	282904	110	365	0.00264	40.2848	cl37540	Herpes_BLLF1 superfamily
	Hydfim1_122796_CE122795_4920	superfamily	282904	106	344	0.001297	41.4404	cl37540	Herpes_BLLF1 superfamily
	Amaapr1_3019461_gm1_10019_g	superfamily	282904	85	269	0.002862	40.2848	cl37540	Herpes_BLLF1 superfamily
	LacbiH53_1_496399_estExt_fgenesh1_pg_C_10204	superfamily	282904	118	359	1.14E-05	47.9888	cl37540	Herpes_BLLF1 superfamily
	LacbiH70_1_526232_estExt_fgenesh1_pg_C_20118	superfamily	282904	118	358	5.66E-06	49.1444	cl37540	Herpes_BLLF1 superfamily
	LacbiH82_1_420993_estExt_Genewise1Plus_C_1_t10386	superfamily	282904	118	359	1.09E-05	47.9888	cl37540	Herpes_BLLF1 superfamily
	LacbiH82xH70_1_524692_estExt_fgenesh1_pm_C_290017	superfamily	282904	118	359	1.14E-05	47.9888	cl37540	Herpes_BLLF1 superfamily
	Lacbi2_586995_e_gw1_3_9234_1	superfamily	282904	118	358	5.67E-06	49.1444	cl37540	Herpes_BLLF1 superfamily
	Cerbo1_2712_transcript_CEH14671	superfamily	282904	113	411	0.001365	41.8256	cl37540	Herpes_BLLF1 superfamily
	Mersum1_1115547_fgenesh1_pg_2_862	superfamily	183756	206	324	0.000254	43.5509	cl32828	motB superfamily
	Hygaur1_995974_e_gw1_4_168_1	superfamily	130689	136	249	0.000203	44.0274	cl31127	PABP-1234 superfamily
	Diohu1_40973_gm1_3395_g	superfamily	223021	165	498	3.39E-05	47.2405	cl33720	PHA03247 superfamily
	Diocr1_384589_gm1_8902_g	superfamily	223021	14	457	1.01E-06	52.2481	cl33720	PHA03247 superfamily
	Diocr1_385403_gm1_9716_g	superfamily	223021	14	488	4.37E-06	50.3221	cl33720	PHA03247 superfamily
	Papla1_31175_CE31174_7731	superfamily	223021	172	419	0.004112	40.3069	cl33720	PHA03247 superfamily
	CgaCA1873_1_272_transcript_KIR69173	superfamily	223021	123	366	0.009432	39.1513	cl33720	PHA03247 superfamily
	Lenvul1_1067860_gm1_1373_g	superfamily	223021	112	370	0.000198	44.1589	cl33720	PHA03247 superfamily
	Copci1_17752_fgeneshDR_te_pg_Chr_5_593	superfamily	223021	143	312	8.43E-05	45.3145	cl33720	PHA03247 superfamily
	Mycreb1_753034_CE753033_3934	superfamily	223021	129	353	4.97E-06	49.1665	cl33720	PHA03247 superfamily
	Treme1_74694_estExt_fgeneshTM_pg_C_130186	superfamily	223021	56	413	7.09E-05	46.0849	cl33720	PHA03247 superfamily
	Mycvit1_463514_CE463513_8015	superfamily	223021	128	315	0.002089	41.0773	cl33720	PHA03247 superfamily
	Spalat1_816697_fgenesh1_pm_21_28	superfamily	223021	112	414	5.54E-05	46.4701	cl33720	PHA03247 superfamily
	Mycfil1_250871_CE250870_3308	superfamily	223021	178	341	0.00404	39.9217	cl33720	PHA03247 superfamily
	Treen1_559038_gm1_3583_g	superfamily	223021	10	460	0.002336	41.4625	cl33720	PHA03247 superfamily
	Anomyc1_1589155_e_gw1_3_1866_1	superfamily	223021	136	338	0.001944	41.0773	cl33720	PHA03247 superfamily
	Hercor1_1045299_gm1_3848_g	superfamily	223021	146	407	4.87E-09	59.1817	cl33720	PHA03247 superfamily
	Dicsqu464_1_955638_estExt_fgenesh1_pm_C_70025	superfamily	223021	119	403	1.79E-06	51.0925	cl33720	PHA03247 superfamily
	Dicsq1_161205_estExt_fgenesh1_pg_C_100189	superfamily	223021	119	403	1.79E-06	51.0925	cl33720	PHA03247 superfamily
	Albpec1_1527448_e_gw1_5_147_1	superfamily	223021	113	431	0.000391	43.3885	cl33720	PHA03247 superfamily

Rusvin1_959169_fgenesh1_pg_4_145	superfamily	223021	109	369	0.0037	40.3069	cl33720	PHA03247 superfamily
Leumo1_1054359_estExt_Genewise1Plus_C_000040098	superfamily	223021	109	362	5.62E-05	46.0849	cl33720	PHA03247 superfamily
Phchr2_3002211_fgenesh1_pm_2_463	superfamily	223021	105	447	9.26E-08	55.3297	cl33720	PHA03247 superfamily
Serbor1_70075_CE70074_7872	superfamily	223021	108	318	1.77E-05	47.6257	cl33720	PHA03247 superfamily
Cligib1_1608310_fgenesh1_pm_2_442	superfamily	223021	111	312	5.42E-05	45.6997	cl33720	PHA03247 superfamily
Jaaar1_225099_CE12727_973	superfamily	223021	114	419	2.71E-06	50.3221	cl33720	PHA03247 superfamily
Polar1_622243_MIX14193_654_22	superfamily	223021	119	398	0.000176	44.5441	cl33720	PHA03247 superfamily
Polsqu1_736070_gm1_2232_g	superfamily	223021	119	410	5.93E-06	49.5517	cl33720	PHA03247 superfamily
Amylap1_1_847318_MIX17964_2136_22	superfamily	223021	112	368	9.13E-05	45.3145	cl33720	PHA03247 superfamily
Rhiso1_2534_g2534_t1	superfamily	223021	205	478	0.003484	40.6921	cl33720	PHA03247 superfamily
Densp1_925963_estExt_Genemark1_C_1_t20071	superfamily	223021	125	409	0.003802	40.3069	cl33720	PHA03247 superfamily
Possti1_1293113_fgenesh1_pm_9_51	superfamily	223021	111	395	0.000236	44.1589	cl33720	PHA03247 superfamily
Trapub1_3791_scaffold_1479_31_No_Blast_Hit	superfamily	223021	111	350	5.25E-08	56.1001	cl33720	PHA03247 superfamily
Pycpun1_518988_estExt_fgenesh1_pm_C_1_t10299	superfamily	223021	135	408	1.42E-07	54.5593	cl33720	PHA03247 superfamily
Pycco1_1466085_gm1_3594_g	superfamily	223021	111	403	9.05E-08	55.3297	cl33720	PHA03247 superfamily
Trabet1_734505_e_gw1_30_162_1	superfamily	223021	110	411	1.01E-06	51.8629	cl33720	PHA03247 superfamily
Tragib1_1441534_MIX17121_2300_23	superfamily	223021	111	411	1.93E-05	47.6257	cl33720	PHA03247 superfamily
Tramax1_961474_e_gw1_1_3373_1	superfamily	223021	111	409	1.96E-09	60.7225	cl33720	PHA03247 superfamily
Crula1_194471_CE194470_3248	superfamily	223021	110	360	1.71E-05	47.6257	cl33720	PHA03247 superfamily
Cyapal1_123313_CE123312_1855	superfamily	223021	116	371	1.07E-06	51.4777	cl33720	PHA03247 superfamily
Coraus1_820769_e_gw1_66_168_1	superfamily	223021	124	356	2.54E-06	50.3221	cl33720	PHA03247 superfamily
CorKIS3_1_1125032_e_gw1_5_906_1	superfamily	223021	118	270	0.003972	39.9217	cl33720	PHA03247 superfamily
Ruseme1_1798846_gm1_1067_g	superfamily	223021	112	350	5.28E-06	49.5517	cl33720	PHA03247 superfamily
Laesu1_175547_CE175546_5608	superfamily	223021	151	389	5.53E-05	46.0849	cl33720	PHA03247 superfamily
Onnsc1_971022_estExt_Genemark1_C_600012	superfamily	223021	115	375	7.21E-07	52.2481	cl33720	PHA03247 superfamily
Gymjun1_1811598_fgenesh1_pm_1_340	superfamily	223021	116	345	0.000316	43.3885	cl33720	PHA03247 superfamily
Ganluc1_347232_e_gw1_6_832_1	superfamily	223021	119	405	3.64E-09	59.9521	cl33720	PHA03247 superfamily
Pycsa1_1561522_e_gw1_7180000650711_72_1	superfamily	223021	111	408	1.16E-07	54.9445	cl33720	PHA03247 superfamily
Fibsp1_543990_CE543989_745	superfamily	223021	111	368	0.003003	40.6921	cl33720	PHA03247 superfamily
Cyastr2_1238910_e_gw1_15_222_1	superfamily	223021	123	271	0.00107	41.8477	cl33720	PHA03247 superfamily
Flafl1_1321536_fgenesh1_pg_4_273	superfamily	223021	114	375	5.34E-05	46.0849	cl33720	PHA03247 superfamily
Epityp1_40807_e_gw1_4_603_1	superfamily	223021	110	306	0.000716	42.6181	cl33720	PHA03247 superfamily
Cervir1_2011117_fgenesh1_pm_2_184	superfamily	223021	100	347	1.74E-07	53.7889	cl33720	PHA03247 superfamily
Persub1_2854441_fgenesh1_pm_12_251	superfamily	223021	124	409	0.000106	45.3145	cl33720	PHA03247 superfamily
Niavib1_997546_e_gw1_12_252_1	superfamily	223021	118	288	3.45E-05	46.4701	cl33720	PHA03247 superfamily
Tralj1_1008738_estExt_Genewise1_C_1_t20331	superfamily	223021	110	407	8.04E-07	52.2481	cl33720	PHA03247 superfamily
Cytmel1_1407068_fgenesh1_pm_109_5	superfamily	223021	106	414	2.73E-05	47.2405	cl33720	PHA03247 superfamily
Borrad1_1876818_MIX63913_2543_69	superfamily	223021	117	382	9.61E-06	48.7813	cl33720	PHA03247 superfamily
Perfra1_266615_CE266614_12390	superfamily	223021	163	439	5.42E-05	46.4701	cl33720	PHA03247 superfamily
Fomfom1_1201309_e_gw1_6_2153_1	superfamily	223021	111	402	5.82E-09	59.1817	cl33720	PHA03247 superfamily
Pyccin1_920900_e_gw1_5_416_1	superfamily	223021	111	404	4.69E-07	53.0185	cl33720	PHA03247 superfamily
Traci1_1407726_e_gw1_29_242_1	superfamily	223021	145	407	3.72E-06	49.9369	cl33720	PHA03247 superfamily
Trave1_116956_e_gw1_3_3124_1	superfamily	223021	110	412	1.78E-06	51.0925	cl33720	PHA03247 superfamily
Stehi1_140265_estExt_fgenesh1_pg_C_90406	superfamily	223021	145	271	0.002902	41.0773	cl33720	PHA03247 superfamily
Fompi3_1032620_fgenesh1_pm_75_24	superfamily	223021	110	410	0.000415	43.3885	cl33720	PHA03247 superfamily
LeugonAS2_1_1860172_e_gw1_336_27_1	superfamily	223021	141	358	9.67E-05	45.3145	cl33720	PHA03247 superfamily
Phlsub1_2174547_gm1_7691_g	superfamily	223021	104	354	0.006842	39.5365	cl33720	PHA03247 superfamily
Lerer1_982024_e_gw1_13_205_1	superfamily	223021	115	337	2.74E-05	46.8553	cl33720	PHA03247 superfamily
Lerce1_960489_e_gw1_43_148_1	superfamily	223021	115	345	7.24E-07	51.8629	cl33720	PHA03247 superfamily
Trace1_466207_CE466206_9027	superfamily	223021	113	399	0.002872	40.6921	cl33720	PHA03247 superfamily
Leugon1_1374220_fgenesh1_pm_27_6	superfamily	223021	129	359	0.002768	40.6921	cl33720	PHA03247 superfamily
Hebvel2_1216527_gm1_6164_g	superfamily	223021	114	345	2.80E-07	53.4037	cl33720	PHA03247 superfamily
Phoaln1_1653877_e_gw1_15_137_1	superfamily	223021	111	368	0.000728	42.2329	cl33720	PHA03247 superfamily
Phohig1_7480_CE7479_3402	superfamily	223021	182	361	2.48E-06	50.7073	cl33720	PHA03247 superfamily
Exigl1_758164_gm1_145_g	superfamily	223021	120	376	5.19E-07	52.6333	cl33720	PHA03247 superfamily
Crafun1_831580_MIX19268_1425_38	superfamily	223021	106	352	0.002177	40.6921	cl33720	PHA03247 superfamily
Psiser1_82643_fgenesh1_pm_99_111	superfamily	223021	134	375	0.000192	44.1589	cl33720	PHA03247 superfamily
Pornie1_2_1043763_estExt_Genemark1_C_40178	superfamily	223021	114	367	0.004251	39.9217	cl33720	PHA03247 superfamily
Rusdis1_1264899_gm1_2212_g	superfamily	223021	215	367	0.00697	39.1513	cl33720	PHA03247 superfamily
Conapa1_666483_e_gw1_11_85_1	superfamily	223021	124	361	0.000383	43.3885	cl33720	PHA03247 superfamily
Varmin1_85404_gm1_4068_g	superfamily	223021	113	364	5.65E-06	49.5517	cl33720	PHA03247 superfamily
Copci1_1148_CC1G_01332T0	superfamily	223021	43	212	0.000121	44.1589	cl33720	PHA03247 superfamily
Copci_AmutBmut1_365674_e_gw1_49_72_1	superfamily	223021	43	212	1.28E-05	47.2405	cl33720	PHA03247 superfamily
Copmar1_599891_e_gw1_158_41_1	superfamily	223021	47	232	1.12E-05	47.6257	cl33720	PHA03247 superfamily
Pycci1_9528_scf185015_g89	superfamily	223021	7	294	4.98E-06	49.1665	cl33720	PHA03247 superfamily

	Rhoba1_1_51058_estExt_Genemark1_C_1_t20221	superfamily	223021	116	240	0.001126	41.0773	cl33720	PHA03247 superfamily
	Spoli1_198007_gm1_2903_g	superfamily	223021	156	428	1.50E-10	64.1893	cl33720	PHA03247 superfamily
	CgaWM276_1_488_transcript_ADV19817	superfamily	165527	243	389	0.000326	43.1784	cl29788	PHA03269 superfamily
	Schco2251_1_201178_e_gw1_6_266_1	superfamily	223039	103	415	2.50E-05	47.0881	cl33723	PHA03307 superfamily
	SchcoUNK_1_80868_fgenesh1_pm_32_48	superfamily	223039	103	415	3.90E-05	46.7029	cl33723	PHA03307 superfamily
	SchcoZB2_119624_gm1_10660_g	superfamily	223039	106	415	5.08E-06	49.3993	cl33723	PHA03307 superfamily
	Schco2231_1_286364_gm1_1083_g	superfamily	223039	103	414	0.002969	40.5397	cl33723	PHA03307 superfamily
	Schrad1_233334_gm1_11449_g	superfamily	223039	114	342	1.71E-06	50.9401	cl33723	PHA03307 superfamily
	Schrad1_97742_e_gw1_1066_4_1	superfamily	223039	106	417	2.25E-05	47.4733	cl33723	PHA03307 superfamily
	Stepol1_72792_gm1_4005_g	superfamily	223039	187	443	6.86E-05	45.9325	cl33723	PHA03307 superfamily
	Cryne_H99_1_488_CNAG_00564T0	superfamily	223039	101	315	0.002651	40.9249	cl33723	PHA03307 superfamily
	Mycpur1_612267_MIX30984_1_4	superfamily	223039	102	372	1.42E-05	47.8585	cl33723	PHA03307 superfamily
	Rhodsp1_298602_gm1_2810_g	superfamily	223039	14	303	2.09E-05	47.8585	cl33723	PHA03307 superfamily
	Tilco1_2241_transcript_OAJ08835	superfamily	223039	57	311	0.000831	42.0805	cl33723	PHA03307 superfamily
	Skebig1_395698_CE395697_12679	superfamily	223061	137	236	0.00056	42.6789	cl25753	PHA03369 superfamily
	Cyaste1_240823_gm4_16508_g	superfamily	223065	114	244	4.13E-05	46.2136	cl33729	PHA03378 superfamily
	Ompol1_5503_MUStwsD_GLEAN_10003413	superfamily	223065	50	150	0.006293	38.5096	cl33729	PHA03378 superfamily
	Glocon1_677921_CE677920_8110	superfamily	215130	240	360	0.00421	39.6872	cl33436	PLN02217 superfamily
	Chrvin1_2460327_e_gw1_6_547_1	superfamily	178748	122	240	0.000391	42.9911	cl25752	PLN03209 superfamily
	Antsi1_720013_fgenesh1_pm_19_5	superfamily	178748	147	359	0.00799	39.1391	cl25752	PLN03209 superfamily
	Schco_TatD_1_277283_estExt_Genewise1_C_2330009	superfamily	236090	173	398	8.36E-05	45.3621	cl35613	PRK07764 superfamily
	Mycsan1_212082_CE212081_4619	superfamily	236090	131	322	0.000206	43.8213	cl35613	PRK07764 superfamily
	Cryam1_9298_ODN76812	superfamily	236090	216	366	0.004591	39.9693	cl35613	PRK07764 superfamily
	Cryam1_9299_ODN76813	superfamily	236090	216	366	0.004591	39.9693	cl35613	PRK07764 superfamily
	Polbr1_1431927_estExt_Genewise1Plus_C_8_t20203	superfamily	236090	149	329	0.008493	38.8137	cl35613	PRK07764 superfamily
	Bolvit1_73438_CE73437_5154	superfamily	236090	80	256	1.15E-06	50.7549	cl35613	PRK07764 superfamily
	Gansi1_1032_transcript_PIL37344	superfamily	236090	130	338	1.39E-08	57.6885	cl35613	PRK07764 superfamily
	Leisp1_1430809_estExt_Genemark1_C_50224	superfamily	236090	99	315	7.50E-05	45.3621	cl35613	PRK07764 superfamily
	Hypsu1_238534_CE17528_15200	superfamily	236090	97	266	0.000181	43.8213	cl35613	PRK07764 superfamily
	Rusoch1_623378_e_gw1_3_1790_1	superfamily	236090	136	351	0.002706	40.3545	cl35613	PRK07764 superfamily
	Creces1_1129101_gm1_269_g	superfamily	236090	136	360	0.001765	41.1249	cl35613	PRK07764 superfamily
	Mycgal1_988641_CE988640_1782	superfamily	236090	130	367	4.11E-05	46.1325	cl35613	PRK07764 superfamily
	Pycful1_2588213_e_gw1_5_739_1	superfamily	236138	154	287	0.000708	42.1602	cl35634	PRK07994 superfamily
	Ganleu1_656308_CE656307_8618	superfamily	236333	117	337	6.80E-08	55.0998	cl35739	PRK08691 superfamily
	TerJ132_1_5215_transcript_KNZ77203	superfamily	236333	126	327	0.000139	44.3142	cl35739	PRK08691 superfamily
	Panpap1_1334481_e_gw1_1_2267_1	superfamily	236333	103	345	0.000649	42.3882	cl35739	PRK08691 superfamily
	Irplac1_1594977_gm1_8647_g	superfamily	236333	70	222	4.57E-05	45.855	cl35739	PRK08691 superfamily
	Mycalb1_947558_gm1_1122_g	superfamily	236669	131	286	1.36E-06	51.2391	cl35903	PRK10263 superfamily
	Corsan1_1_2852071_MIX178957_5377_95	superfamily	236669	137	328	2.45E-06	50.0835	cl35903	PRK10263 superfamily
	Hydpi2_79354_e_gw1_1_862_1	superfamily	236669	235	349	0.004178	40.0683	cl35903	PRK10263 superfamily
	Rhivul1_670292_e_gw1_1_522_1	superfamily	236669	142	342	0.002728	40.4535	cl35903	PRK10263 superfamily
	Phlrad1_156433_minus_g12884_t1	superfamily	236669	126	278	0.009318	38.9127	cl35903	PRK10263 superfamily
	Lepmol1_2344755_fgenesh1_pg_2_243	superfamily	236669	146	288	0.000984	41.9943	cl35903	PRK10263 superfamily
	Cysmur1_882604_CE882603_11298	superfamily	236669	152	334	0.001885	40.8387	cl35903	PRK10263 superfamily
	Psicub1_1_30911_e_gw1_1085_51_1	superfamily	236669	222	333	0.001923	40.8387	cl35903	PRK10263 superfamily
	Galinc1_732502_CE732501_4080	superfamily	236669	274	445	0.00085	42.3795	cl35903	PRK10263 superfamily
	Pirin1_72802_mRNA_PIIN_02008	superfamily	236669	133	301	0.004486	39.6831	cl35903	PRK10263 superfamily
	Xenvag1_1828531_gm1_4803_g	superfamily	237057	166	372	0.002907	41.0147	cl36108	PRK12323 superfamily
	Lepnud1_1161222_e_gw1_23_119_1	superfamily	237057	117	334	0.000668	42.1703	cl36108	PRK12323 superfamily
	Gansp1_41937_e_gw1_2_1914_1	superfamily	237057	111	292	4.75E-05	46.0223	cl36108	PRK12323 superfamily
	Wolco1_131571_estExt_fgenesh1_pm_C_4_t10119	superfamily	237057	130	350	0.000136	44.8667	cl36108	PRK12323 superfamily
	Lacbi55996_1_959084_e_gw1_1_101_1	superfamily	184281	136	242	7.14E-07	51.3623	cl42933	PRK13729 superfamily
	Fomros1_171720_CE171719_3257	superfamily	237864	143	229	0.000194	44.03	cl36446	PRK14950 superfamily
	Pospl1_129507_estExt_fgenesh3_pg_C_500065	superfamily	184923	237	374	0.001	41.9743	cl33044	PRK14959 superfamily
	Mycpol1_1314676_gm1_2130_g	superfamily	184923	151	295	0.000401	43.1299	cl33044	PRK14959 superfamily
	Corgl3_7132530_estExt_Genewise1_C_257_t10021	superfamily	184923	142	254	0.000452	42.7447	cl33044	PRK14959 superfamily
	Corcam1_550669_e_gw1_4_729_1	superfamily	184923	92	276	0.00109	41.2039	cl33044	PRK14959 superfamily
	Coppel1_470004_e_gw1_22_91_1	superfamily	184923	80	174	0.000785	41.2039	cl33044	PRK14959 superfamily
	Volvo1_111900_nucleoside	superfamily	185594	121	326	0.000231	43.9103	cl33180	PTZ00395 superfamily
	MroMCA2997_1_5809_Moror_13896	superfamily	185594	134	323	0.00012	44.6807	cl33180	PTZ00395 superfamily
	Paxam1_1030044_MIX11864_1269_24	superfamily	185594	174	320	1.17E-05	48.1475	cl33180	PTZ00395 superfamily
	Amysub1_4614752_gm1_2586_g	superfamily	185594	223	411	0.001747	41.5991	cl33180	PTZ00395 superfamily
	Theter1_1012593_e_gw1_17_52_1	superfamily	227709	121	392	0.000982	41.8003	cl34999	ROM1 superfamily
	Resbic1_1200123_estExt_Genemark1_C_4_t10384	superfamily	227709	119	354	2.05E-05	47.1931	cl34999	ROM1 superfamily
	Agabi_varbur_1_113120_estExt_fgenesh1_kg_C_50088	superfamily	227709	207	378	0.001003	41.8003	cl34999	ROM1 superfamily
	Anobom1_1211083_e_gw1_83_1_1	superfamily	425992	161	250	0.00888	38.3998	cl03073	TYA superfamily
Bas-Velvet2	Pospl1_127743_estExt_fgenesh3_pg_C_460027	superfamily	427171	1584	1973	0.008144	41.2906	cl38111	Atrophin-1 superfamily
	Lened1_2737_LENED_012286	superfamily	427171	769	1028	0.000522	44.7574	cl38111	Atrophin-1 superfamily
	Lened1_2736_LENED_012287	superfamily	427171	769	1028	0.000335	45.1426	cl38111	Atrophin-1 superfamily
	LedB17_3_135991_GENE07118_1	superfamily	427171	618	772	0.00366	40.9054	cl38111	Atrophin-1 superfamily
	Sclyun1_2006976_gm4_5516_g	superfamily	455732	663	834	0.008431	39.5976	cl42387	dermokine superfamily

Pospl1_127743_estExt_fgenesh3_pg_C_460027	superfamily	455732	1826	2017	0.006493	41.1384	cl42387	dermokine superfamily
Lened1_2737_LENED_012286	superfamily	455861	960	1137	0.008894	40.5792	cl42516	dnaA superfamily
Lened1_2736_LENED_012287	superfamily	455861	960	1137	0.008718	40.5792	cl42516	dnaA superfamily
Lentinedodes1_10281_LE01Gene05754_t1	superfamily	455861	950	1127	0.007644	40.5792	cl42516	dnaA superfamily
Chrvin1_2037576_CE2037575_10688	superfamily	455861	365	602	7.75E-08	55.9871	cl42516	dnaA superfamily
Cysmur1_1282261_gm1_5977_g	superfamily	455861	338	466	0.00051	42.5052	cl42516	dnaA superfamily
Suiqui1_928227_estExt_Genemark1_C_9_t10020	superfamily	455861	363	535	3.13E-05	47.1276	cl42516	dnaA superfamily
Pismi2_4495247_gm1_10285_g	superfamily	455861	548	730	0.000396	44.046	cl42516	dnaA superfamily
Pismi2_4495596_gm1_10634_g	superfamily	455861	548	730	0.000396	44.046	cl42516	dnaA superfamily
Paxam1_983512_fgenesh1_pg_171_7	superfamily	455861	616	819	0.000125	45.5868	cl42516	dnaA superfamily
Suilak1_1189890_CE1189889_11290	superfamily	436069	211	532	0.009935	39.1296	cl39316	DUF5585 superfamily
Pismar1_3058664_estExt_Genewise1_C_310038	superfamily	282904	265	508	0.000569	42.9812	cl37540	Herpes_BLLF1 superfamily
Cyaste1_172897_fgenesh1_pg_2_702	specific	433309	257	307	0.006761	34.8111	pfam13560	HTH_31
Suifus1_986343_fgenesh1_pg_114_15	superfamily	430088	461	685	0.001639	41.7393	cl25816	KAR9 superfamily
Pospl1_104508_fgenesh3_pg_64_29	superfamily	237030	737	821	0.001158	42.9554	cl36091	kgd superfamily
Aphpse1_56533_CE56532_1964	superfamily	425360	419	522	0.001985	41.1826	cl41729	KLF1_2_4_N superfamily
Ompol1_6403_MUStwsD_GLEAN_10002796	superfamily	425360	395	461	0.001983	41.1826	cl41729	KLF1_2_4_N superfamily
Rhisol1_607_transcript_EUC54060	superfamily	425360	282	392	3.45E-07	53.509	cl41729	KLF1_2_4_N superfamily
Rhoto_IFO1236_1_45954_mRNA_14_16	superfamily	452895	66	435	3.79E-43	163.279	cl28910	MFS superfamily
Sporo1_31687_estExt_fgenesh1_pg_C_20107	superfamily	452895	279	689	3.85E-78	262.125	cl28910	MFS superfamily
Rhiso1_7439_g7439_t1	superfamily	401645	274	403	0.0012	43.1008	cl37801	PAT1 superfamily
Pirin1_78153_mRNA_PIIN_07346	superfamily	401645	528	693	7.63E-05	46.1824	cl37801	PAT1 superfamily
LboTFB7829_1_340207_CE340206_20846	superfamily	401645	322	458	3.36E-05	47.7232	cl37801	PAT1 superfamily
Amamu1_24595_estExt_Genemark1_C_290019	superfamily	401645	493	641	0.003042	41.1748	cl37801	PAT1 superfamily
Thega1_1814795_CE1814794_5202	superfamily	401645	452	527	8.53E-05	45.7972	cl37801	PAT1 superfamily
Thacu1_134709_CE134708_7655	superfamily	401645	189	273	0.000313	44.6416	cl37801	PAT1 superfamily
Leisp1_1312433_estExt_Genewise1Plus_C_1480004	superfamily	226193	323	424	0.00769	39.0897	cl43925	PcoB superfamily
Mychae1_120655_CE120654_2315	superfamily	223020	200	294	0.002944	40.345	cl33719	PHA03246 superfamily
Sismus1_1194206_CE1194205_32373	superfamily	223021	532	788	3.34E-05	47.6257	cl33720	PHA03247 superfamily
Tulinq1_1669787_gm1_18548_g	superfamily	223021	290	814	2.79E-13	74.2044	cl33720	PHA03247 superfamily
Sebvebe1_816047_gm1_6487_g	superfamily	223021	399	887	1.27E-07	56.1001	cl33720	PHA03247 superfamily
Conol1_795459_gw1_329_10_1	superfamily	223021	269	546	6.81E-11	65.73	cl33720	PHA03247 superfamily
Pirin1_78153_mRNA_PIIN_07346	superfamily	223021	334	606	1.57E-08	58.7965	cl33720	PHA03247 superfamily
Niavib1_182722_CE182721_6749	superfamily	223021	336	683	4.66E-05	47.2405	cl33720	PHA03247 superfamily
Conpu1_168681_estExt_Genemark1_C_130391	superfamily	223021	246	542	2.68E-06	50.7073	cl33720	PHA03247 superfamily
LacbiN203_1_486613_CE486612_7173	superfamily	223021	313	529	2.68E-05	47.2405	cl33720	PHA03247 superfamily
LacbiS238N_1_509427_fgenesh1_pg_9_194	superfamily	223021	400	617	0.001923	42.2329	cl33720	PHA03247 superfamily
LacbiH70_1_172399_CE172398_3547	superfamily	223021	313	530	0.001876	41.8477	cl33720	PHA03247 superfamily
LacbiH82_1_200880_CE200879_2495	superfamily	223021	313	530	0.001876	41.8477	cl33720	PHA03247 superfamily
LacbiH82xH70_1_14547_CE14546_4054	superfamily	223021	313	530	0.001876	41.8477	cl33720	PHA03247 superfamily
LacbiS238O_1_163330_gm1_11450_g	superfamily	223021	298	515	0.003282	41.4625	cl33720	PHA03247 superfamily
Lened1_2737_LENED_012286	superfamily	223021	357	430	0.008835	40.6921	cl33720	PHA03247 superfamily
Lened1_2736_LENED_012287	superfamily	223021	357	430	0.007699	41.0773	cl33720	PHA03247 superfamily
Lacbi2_448047_gm1_12491_g	superfamily	223021	313	530	0.00356	41.0773	cl33720	PHA03247 superfamily
Suisib1_719026_CE719025_9762	superfamily	223021	272	676	2.74E-11	67.656	cl33720	PHA03247 superfamily
Suiplo1_55650_CE55649_9389	superfamily	223021	203	514	0.000146	45.6997	cl33720	PHA03247 superfamily
Suisib1_2279997_estExt_fgenesh1_pg_C_240070	superfamily	223021	272	676	4.96E-11	66.8856	cl33720	PHA03247 superfamily
Suitom1_781148_gm1_14773_g	superfamily	223021	203	623	4.98E-07	53.7889	cl33720	PHA03247 superfamily
Suifus1_986343_fgenesh1_pg_114_15	superfamily	223021	253	572	6.59E-07	53.4037	cl33720	PHA03247 superfamily
Paxin1_101260_estExt_Genemark1_C_430009	superfamily	223021	572	864	0.000141	46.0849	cl33720	PHA03247 superfamily
Suisubl1_1925720_gm1_13052_g	superfamily	223021	203	526	4.34E-07	53.7889	cl33720	PHA03247 superfamily
PhlFC14_2_1988220_MIX17022_16914_77	superfamily	223021	224	638	2.29E-07	54.9445	cl33720	PHA03247 superfamily
Suivis2_1552108_fgenesh1_pg_248_17	superfamily	223021	337	648	6.49E-08	56.4853	cl33720	PHA03247 superfamily
Suiplac1_161061_CE161060_12586	superfamily	223021	203	515	2.17E-07	54.9445	cl33720	PHA03247 superfamily
Suipse1_665534_CE665533_3791	superfamily	223021	203	656	2.53E-07	54.5593	cl33720	PHA03247 superfamily
SuiFC429_1_1362418_gm1_8783_g	superfamily	223021	203	507	2.36E-07	54.5593	cl33720	PHA03247 superfamily
Suipic1_1461634_estExt_fgenesh1_pg_C_1140004	superfamily	223021	272	639	8.94E-10	62.6485	cl33720	PHA03247 superfamily
Suisuba1_223969_MIX26747_69_39	superfamily	223021	224	666	4.67E-08	57.2557	cl33720	PHA03247 superfamily
Suibov1_752028_CE752027_17150	superfamily	223021	324	715	1.02E-06	53.0185	cl33720	PHA03247 superfamily
Suisu1_697657_estExt_Genewise1Plus_C_120022	superfamily	223021	267	505	8.71E-06	48.7813	cl33720	PHA03247 superfamily
Suisub1_717628_CE717627_6986	superfamily	223021	247	676	5.72E-10	63.4189	cl33720	PHA03247 superfamily
Suicli1_571473_CE571472_1840	superfamily	223021	224	579	4.85E-10	63.0337	cl33720	PHA03247 superfamily

Suielb1_1095410_fgenesh1_pg_118_7	superfamily	223021	337	648	1.49E-07	55.3297	cl33720	PHA03247 superfamily
Suiocc1_970829_estExt_Genemark1_C_1100015	superfamily	223021	265	626	3.18E-06	51.0925	cl33720	PHA03247 superfamily
Suipal1_1050696_e_gw1_16_280_1	superfamily	223021	228	547	4.14E-07	53.4037	cl33720	PHA03247 superfamily
Auramp1_569146_gm1_6541_g	superfamily	223021	538	762	0.002683	41.8477	cl33720	PHA03247 superfamily
Botbo1_177452_gm1_8987_g	superfamily	223021	258	732	0.000338	44.5441	cl33720	PHA03247 superfamily
Schrad1_77061_e_gw1_268_33_1	superfamily	223021	261	745	4.02E-07	54.5593	cl33720	PHA03247 superfamily
SchcoUNK_1_43169_e_gw1_39_53_1	superfamily	223021	261	737	8.45E-06	49.9369	cl33720	PHA03247 superfamily
SchcoZB2_52085_e_gw1_50_31_1	superfamily	223021	219	635	0.001298	43.0033	cl33720	PHA03247 superfamily
Schrad1_73154_e_gw1_183_45_1	superfamily	223021	261	739	0.000295	44.9293	cl33720	PHA03247 superfamily
Daequ1_321421_CE321420_4888	superfamily	223021	278	694	0.002922	41.4625	cl33720	PHA03247 superfamily
Suipunc1_52268_CE52267_7866	superfamily	223021	200	509	4.5E-05	47.2405	cl33720	PHA03247 superfamily
Kurarg1_117906_CE117905_3182	superfamily	223021	397	682	5.26E-07	53.7889	cl33720	PHA03247 superfamily
Jaaar1_70879_estExt_fgenesh1_pm_C_150131	superfamily	223021	353	693	1.68E-08	58.4113	cl33720	PHA03247 superfamily
Cylto1_366949_estExt_Genewise1_C_100108	superfamily	223021	245	507	4.78E-08	56.8705	cl33720	PHA03247 superfamily
Tulsp419_1_1324975_CE1324974_35255	superfamily	223021	438	619	9.29E-05	46.4701	cl33720	PHA03247 superfamily
Panst_LUM_1_1_870624_CE870623_1779	superfamily	223021	250	425	0.000172	44.1589	cl33720	PHA03247 superfamily
Mycmet1_1688523_estExt_fgenesh1_pg_C_360034	superfamily	223021	204	440	0.004399	39.5365	cl33720	PHA03247 superfamily
LacbiDR170_1_1070519_estExt_fgenesh1_pg_C_12030001	superfamily	223021	400	617	0.000943	43.0033	cl33720	PHA03247 superfamily
LedB17_3_135991_GENE07118_1	superfamily	223021	206	279	0.008819	39.9217	cl33720	PHA03247 superfamily
Slotsu1_479308_CE479307_6125	superfamily	223021	254	411	1.7E-06	50.7073	cl33720	PHA03247 superfamily
Rhoba1_1_42518_gm1_2083_g	superfamily	223021	208	415	0.001302	41.8477	cl33720	PHA03247 superfamily
Rhoglu91_1_39000_gm1_1370_g	superfamily	223021	193	507	0.000603	42.6181	cl33720	PHA03247 superfamily
Rhoto_IFO0880_4_15761_CE720556_7022_mRNA	superfamily	223021	174	488	0.00058	42.6181	cl33720	PHA03247 superfamily
Sporo1_31687_estExt_fgenesh1_pg_C_20107	superfamily	223021	914	1059	7.78E-05	47.2405	cl33720	PHA03247 superfamily
Mereb1_352854_gm1_1776_g	superfamily	223021	157	419	3.15E-05	49.9369	cl33720	PHA03247 superfamily
Hethyg1_462811_estExt_fgenesh1_pg_C_100129	superfamily	223021	403	820	1.09E-06	52.6333	cl33720	PHA03247 superfamily
Moean1_1021_rna_PAN0_002c1030	superfamily	223021	291	616	0.000206	44.9293	cl33720	PHA03247 superfamily
Moeaph1_2229_PaG_05153T0	superfamily	223021	292	618	0.001156	42.6181	cl33720	PHA03247 superfamily
Psean1_1_80251_PANT_7c00294	superfamily	223021	292	618	0.000254	44.9293	cl33720	PHA03247 superfamily
Psefl1_2417_rna_PFL1_04961	superfamily	223021	301	780	1.61E-08	58.7965	cl33720	PHA03247 superfamily
Tescy1_86388_CE86387_10361	superfamily	223021	328	788	5.3E-06	50.3221	cl33720	PHA03247 superfamily
Ustbr1_4410_transcript_SAM75730	superfamily	223021	436	850	1.71E-06	52.2481	cl33720	PHA03247 superfamily
Tilwa1_340956_gm1_5278_g	superfamily	223021	423	659	1.72E-08	58.4113	cl33720	PHA03247 superfamily
Cersp1_345285_gm1_1455_g	superfamily	223021	494	724	0.000424	44.1589	cl33720	PHA03247 superfamily
Melen1_158247_CE158246_6168	superfamily	223021	219	664	2E-08	58.4113	cl33720	PHA03247 superfamily
Mycgale1_638387_CE638386_1733	superfamily	223025	210	335	0.009958	38.387	cl29984	PHA03253 superfamily
Cerbo1_1824_transcript_CEH13785	superfamily	223039	421	723	3.65E-05	47.4733	cl33723	PHA03307 superfamily
Ustsp1_129792_CE129791_6750	superfamily	223039	352	629	1.2E-05	49.0141	cl33723	PHA03307 superfamily
Micld1_2085_MVLG_02359T0	superfamily	223065	311	485	2.6E-05	50.0656	cl33729	PHA03378 superfamily
Schrad1_73154_e_gw1_183_45_1	superfamily	235906	607	880	0.001434	42.5292	cl35530	PRK07003 superfamily
Pycpun1_512725_fgenesh1_pm_3_65	superfamily	236090	260	440	0.008282	39.5841	cl35613	PRK07764 superfamily
Tralj1_1020596_estExt_Genewise1_C_270097	superfamily	236090	301	460	0.007193	39.1989	cl35613	PRK07764 superfamily
Amylap1_1_824162_gm1_6530_g	superfamily	236090	565	724	0.000619	43.0509	cl35613	PRK07764 superfamily
Porspa1_1857417_estExt_Genewise1_C_190202	superfamily	236090	469	673	3.33E-05	47.6733	cl35613	PRK07764 superfamily
Rhitru1_344191_CE344190_6430	superfamily	236090	257	454	0.001321	42.2805	cl35613	PRK07764 superfamily
Ganluc1_360691_e_gw1_24_215_1	superfamily	236090	583	719	3.24E-07	53.8365	cl35613	PRK07764 superfamily
Rhisa1_871052_estExt_fgenesh1_pm_C_100013	superfamily	236090	254	452	0.000479	43.8213	cl35613	PRK07764 superfamily
Suivari1_1033365_CE1033364_5370	superfamily	236090	227	438	0.001139	42.2805	cl35613	PRK07764 superfamily
Suidis1_781775_gm1_15463_g	superfamily	236090	227	438	0.00606	39.5841	cl35613	PRK07764 superfamily
Auramp1_569146_gm1_6541_g	superfamily	236090	535	619	2.18E-05	48.4437	cl35613	PRK07764 superfamily
Schrad1_77061_e_gw1_268_33_1	superfamily	236090	687	891	0.009869	39.5841	cl35613	PRK07764 superfamily
Schco_TatD_1_268567_estExt_Genewise1_C_840033	superfamily	236090	688	900	0.001427	42.6657	cl35613	PRK07764 superfamily
SchcoUNK_1_43169_e_gw1_39_53_1	superfamily	236090	684	896	0.006499	40.3545	cl35613	PRK07764 superfamily
SchcoZB1_61313_e_gw1_96_110_1	superfamily	236090	687	899	0.001842	42.2805	cl35613	PRK07764 superfamily
SchcoZB2_52085_e_gw1_50_31_1	superfamily	236090	509	738	1.04E-05	49.5993	cl35613	PRK07764 superfamily
SchcoZB2_52085_e_gw1_50_31_1	superfamily	236090	687	899	0.002611	41.5101	cl35613	PRK07764 superfamily
Schfas1_66389_gm1_6929_g	superfamily	236090	495	698	8.76E-06	49.9845	cl35613	PRK07764 superfamily
Schrad1_73154_e_gw1_183_45_1	superfamily	236090	534	725	0.000104	46.1325	cl35613	PRK07764 superfamily
Rhisol1_607_transcript_EUC54060	superfamily	236090	899	1083	0.000281	44.9769	cl35613	PRK07764 superfamily
Wolco1_121729_estExt_Genewise1_C_6_t30169	superfamily	236090	371	445	0.000129	44.9769	cl35613	PRK07764 superfamily
Auramp1_577871_gm1_15266_g	superfamily	236090	577	757	9.88E-05	46.5177	cl35613	PRK07764 superfamily
Porchr1_157083_CE157082_6106	superfamily	236090	426	625	0.000197	44.5917	cl35613	PRK07764 superfamily
Porchr1_425602_CE425601_6082	superfamily	236090	426	625	0.000197	44.5917	cl35613	PRK07764 superfamily
Punst1_116033_fgenesh1_pm_14_33	superfamily	236090	285	383	0.002688	41.5101	cl35613	PRK07764 superfamily
Pycci1_2814_scf184795_g2	superfamily	236090	274	448	0.004194	39.9693	cl35613	PRK07764 superfamily
Fibra1_7604_FIBRA_06256m_01	superfamily	236090	515	692	0.003363	40.7397	cl35613	PRK07764 superfamily
Onnsc1_354243_CE354242_7719	superfamily	236669	452	655	0.000261	44.3055	cl35903	PRK10263 superfamily
Lenrap1_155_70288_fgenesh1_pg_190_8	superfamily	236669	295	377	0.005019	41.2239	cl35903	PRK10263 superfamily
Lentinedodes1_10281_LE01Gene05754_t1	superfamily	236669	755	1028	0.006838	40.8387	cl35903	PRK10263 superfamily

	Amarub1_1273017_estExt_fgenesh1_pm_C_490022	superfamily	236669	572	661	0.00337	41.2239	cl35903	PRK10263 superfamily
	Suiplo1_55650_CE55649_9389	superfamily	236669	410	627	0.000511	43.5351	cl35903	PRK10263 superfamily
	Suidis1_781775_gm1_15463_g	superfamily	236669	338	541	3.78E-05	46.6167	cl35903	PRK10263 superfamily
	Suisubl1_1925720_gm1_13052_g	superfamily	236669	409	650	0.00015	45.4611	cl35903	PRK10263 superfamily
	Suiplac1_161061_CE161060_12586	superfamily	236669	410	626	0.00022	45.0759	cl35903	PRK10263 superfamily
	SuiFC429_1_1362418_gm1_8783_g	superfamily	236669	404	620	8.09E-05	46.2315	cl35903	PRK10263 superfamily
	Suipunc1_52268_CE52267_7866	superfamily	236669	407	624	0.00028	44.6907	cl35903	PRK10263 superfamily
	Conapa1_882507_estExt_fgenesh1_pg_C_1030020	superfamily	237057	239	350	0.002544	41.3999	cl36108	PRK12323 superfamily
	Schco_TatD_1_268567_estExt_Genewise1_C_840033	superfamily	237057	528	740	0.000306	44.4815	cl36108	PRK12323 superfamily
	SchcoZB1_61313_e_gw1_96_110_1	superfamily	237057	527	739	0.000334	44.4815	cl36108	PRK12323 superfamily
	Schfas1_66389_gm1_6929_g	superfamily	237057	600	850	7.57E-06	49.8743	cl36108	PRK12323 superfamily
	Mycepi1_151189_CE151188_2862	superfamily	237057	537	665	0.00801	39.8591	cl36108	PRK12323 superfamily
	Uroocc1_54133_CE54132_24278	superfamily	237057	428	609	0.000219	44.4815	cl36108	PRK12323 superfamily
	Psehu1_4469_PHSY_004939m_01	superfamily	237057	392	603	2.64E-09	60.2747	cl36108	PRK12323 superfamily
	Ptegra1_657025_estExt_fgenesh1_pg_C_180048	superfamily	183558	222	341	0.000746	41.778	cl32772	PRK12495 superfamily
	Myc59_1_640918_CE640917_470	superfamily	237865	274	426	0.000272	43.1644	cl36447	PRK14951 superfamily
	Kalbr1_487_transcript_EST10121	superfamily	237865	555	665	0.004913	40.0828	cl36447	PRK14951 superfamily
	Daequ1_321421_CE321420_4888	superfamily	237871	375	447	0.000588	43.1915	cl36453	PRK14965 superfamily
	Rhobu1_113189_CE113188_925	superfamily	185594	273	416	0.000177	44.6807	cl33180	PTZ00395 superfamily
	Suivari1_1033365_CE1033364_5370	superfamily	185594	373	658	0.000732	43.1399	cl33180	PTZ00395 superfamily
	Suilak1_1189890_CE1189889_11290	superfamily	185594	374	640	3.4E-06	50.8439	cl33180	PTZ00395 superfamily
	Boled5_233466_Boled1_CE233465_29745	superfamily	185594	442	650	0.006837	40.0584	cl33180	PTZ00395 superfamily
	Boledp1_1541191_MIX76635_7578_21	superfamily	185594	442	635	0.001572	42.3695	cl33180	PTZ00395 superfamily
	Fompi3_1060568_estExt_Genemark1_C_310088	superfamily	185594	523	683	5.25E-07	53.5403	cl33180	PTZ00395 superfamily
	Paxam1_983512_fgenesh1_pg_171_7	superfamily	185594	487	670	0.002671	41.5991	cl33180	PTZ00395 superfamily
	Spalat1_826655_gm1_5992_g	superfamily	185616	609	738	0.002908	40.7038	cl33183	PTZ00436 superfamily
	Fibra1_7604_FIBRA_06256m_01	superfamily	273167	306	372	0.00246	41.0339	cl36702	rad23 superfamily
	Micld1_2085_MVLG_02359T0	specific	426620	2553	2760	2.16E-59	202.737	pfam02145	Rap_GAP
	Mereb1_352854_gm1_1776_g	specific	426620	2311	2521	2.42E-65	219.686	pfam02145	Rap_GAP
	Pisalb1_2362488_gm1_4686_g	superfamily	227709	566	781	4.18E-05	47.5783	cl34999	ROM1 superfamily
	Cont1119283_101566_fgenesh1_pm_11_67	superfamily	273721	204	288	0.001631	41.8286	cl36939	SF-CC1 superfamily
	Wolco1_121729_estExt_Genewise1_C_6_t30169	superfamily	273721	276	345	0.000711	42.2138	cl36939	SF-CC1 superfamily
	Flafl1_1418277_gm1_9114_g	superfamily	273721	251	330	0.007895	39.5174	cl36939	SF-CC1 superfamily
	Pospl1_127743_estExt_fgenesh3_pg_C_460027	superfamily	434837	1877	1968	0.005269	40.3383	cl44635	SPATA3 superfamily
	Pospl1_127743_estExt_fgenesh3_pg_C_460027	superfamily	444852	79	451	2.27E-84	282.923	cl00342	Trp-synth-beta_II superfamily
	Micld1_2085_MVLG_02359T0	superfamily	427360	2047	2300	4.26E-33	132.831	cl04144	Tuberin superfamily
	Mereb1_352854_gm1_1776_g	superfamily	427360	1773	2033	6.49E-31	126.283	cl04144	Tuberin superfamily
	Myc59_1_2430144_estExt_Genemark1_C_240139	superfamily	455498	298	352	0.004677	40.2301	cl42153	UPF0182 superfamily
	Copang1_514297_CE514296_7177	superfamily	372950	385	426	0.006726	36.7105	cl16686	YppG superfamily
	Pospl1_127743_estExt_fgenesh3_pg_C_460027	superfamily	449370	443	816	0	556.705	cl14876	Zinc_peptidase_like superfamily
Bas-Velvet3	Jaaar1_194263_gm1_6983_g	specific	432889	2	43	0.00242	37.468	pfam12937	F-box-like
	Dactor1_411860_CE411859_1790	superfamily	401645	282	435	7.62E-06	48.4936	cl37801	PAT1 superfamily
	Tulca1_182676_CE95431_2423	superfamily	223021	236	549	1.66E-07	54.5593	cl33720	PHA03247 superfamily
	Phevit1_1765865_gm1_5218_g	superfamily	223021	12	228	0.003596	41.0773	cl33720	PHA03247 superfamily
	Tulsp425_1_888010_CE888009_7623	superfamily	223021	261	555	4.31E-08	56.4853	cl33720	PHA03247 superfamily
	Antsi1_791169_estExt_Genemark1_C_2010004	superfamily	223021	128	337	3.64E-05	46.8553	cl33720	PHA03247 superfamily
	Copci1_15745_fgeneshDR_te_pg_Chr_3_370	superfamily	223021	682	839	0.006977	40.3069	cl33720	PHA03247 superfamily
	Pycsa1_1470746_CE1470745_606	superfamily	165513	131	217	0.001002	40.2722	cl31530	PHA03255 superfamily
	Porchr1_256207_CE256206_10340	superfamily	165527	60	191	0.001485	41.6376	cl29788	PHA03269 superfamily
	Pornie1_2_55756_CE55755_5978	superfamily	165527	62	193	0.000995	42.408	cl29788	PHA03269 superfamily
	Botbo1_28767_fgenesh1_kg_6_75_Locus17234v1rpkm1_20	superfamily	223039	245	485	1.83E-08	57.1033	cl33723	PHA03307 superfamily
	Calco1_17345_CE17344_3571	superfamily	237057	284	441	0.000329	42.9407	cl36108	PRK12323 superfamily
	Schpa1_433496_CE433495_10189	superfamily	237057	3	222	0.001008	42.5555	cl36108	PRK12323 superfamily
	Macfu1_775400_estExt_Genemark1_C_1110008	superfamily	237865	278	386	0.001171	42.0088	cl36447	PRK14951 superfamily
	Jaaar1_194263_gm1_6983_g	specific	350346	1392	1715	3.92E-66	222.115	cd14496	PTP_paladin
	Jaaar1_194263_gm1_6983_g	specific	350346	441	677	9.85E-59	200.929	cd14496	PTP_paladin
	Jaaar1_194263_gm1_6983_g	specific	350346	976	1278	6.31E-55	189.758	cd14496	PTP_paladin
	Pheign1_1_350525_e_gw1_42_66_1	superfamily	185628	40	262	0.00106	42.3694	cl33186	PTZ00449 superfamily
	Pyccin1_1046036_gm1_579_g	superfamily	431141	414	487	0.000605	42.5097	cl25902	Rrn6 superfamily
	Pycci1_1014_scf184601_g21	superfamily	431141	408	481	0.000699	42.1245	cl25902	Rrn6 superfamily
Chy-VelB	Powhir1_97925_CE97924_4828	superfamily	223021	31	215	0.001368	41.4625	cl33720	PHA03247 superfamily
	Gaesem1_592362_gm1_4145_g	superfamily	223021	4	187	2.73E-05	46.4701	cl33720	PHA03247 superfamily
	Powhir1_444218_pg_43_3	superfamily	223021	31	331	0.000296	43.7737	cl33720	PHA03247 superfamily
	Triarc1_603630_kg_2_546_DN1940_c0_g1_i2	superfamily	215299	279	397	0.005814	39.8909	cl30537	PLN02543 superfamily
	Spipu1_2175_SPPG_06849T0	superfamily	236669	3	174	0.004333	39.2979	cl35903	PRK10263 superfamily
	Spipu1_345_SPPG_00327T0	superfamily	435070	192	339	0.001306	41.0689	cl25748	SAP130_C superfamily
	Enthel1_456833_kg_49_135_DN6402_c5_g2_i1	superfamily	428027	285	344	0.00703	37.7938	cl04615	SelP_N superfamily
Chy-Velvet1	NeoGfMa1_640311_MIX33926_16_2	superfamily	237171	578	652	0.003948	41.4279	cl36163	PRK12678 superfamily

	NeoGfMa1_325469_e_gw1_32_716_1	superfamily	173412	434	747	0.009461	40.1243	cl31754	PTZ00121 superfamily
Chy-VosA	Gaesem1_542589_pg_32_7	superfamily	455732	403	605	0.00123	41.9088	cl42387	dermokine superfamily
	Spipu1_6035_SPPG_02528T0	superfamily	455861	269	492	0.002311	40.9644	cl42516	dnaA superfamily
	Batsa1_34_transcript_OON11198	superfamily	273733	307	389	0.005181	40.185	cl25888	hnRNP-L_PTB superfamily
	Hompol1_6859_HPOL_005119-RA	superfamily	130689	450	556	4.06E-05	46.3386	cl31127	PABP-1234 superfamily
	Spipu1_6035_SPPG_02528T0	superfamily	223021	414	640	0.000282	44.5441	cl33720	PHA03247 superfamily
	Gorhay1_201103_kg_3_1230_DN2215_c1_g2_i1	superfamily	223021	213	691	4.18E-12	69.9672	cl33720	PHA03247 superfamily
	Batde5_35531_estExt_pg_C_90153	superfamily	223021	526	961	3.84E-07	54.5593	cl33720	PHA03247 superfamily
	Chyhya1_409027_CE409026_10348	superfamily	236090	173	339	0.001109	42.2805	cl35613	PRK07764 superfamily
	Clapol1_1_600061_gw1_11_24_1	superfamily	184918	176	240	0.004693	38.0003	cl33039	PRK14954 superfamily
Muc-VelB	MomiAD069_1_2737_BG005_002768_T1	superfamily	450167	29	252	3.97E-16	78.154	cl17173	AdoMet_MTases superfamily
	MoclAM1000_1_6680_BGZ81_006774_T1	superfamily	450167	8	60	2.26E-05	44.0933	cl17173	AdoMet_MTases superfamily
	MoveN2611_1_5620_BGZ59_005681_T1	superfamily	450167	37	108	0.000264	41.3969	cl17173	AdoMet_MTases superfamily
	MoveTTC192_1_5070_CPC16_005137_T1	superfamily	450167	37	108	0.000264	41.3969	cl17173	AdoMet_MTases superfamily
	MoveAD079_1_8320_CPB97_008399_T1	superfamily	450167	534	763	3.42E-16	79.6948	cl17173	AdoMet_MTases superfamily
	HabiNA12553_1_6962_BGZ52_006998_T1	superfamily	450167	16	114	0.000211	43.3229	cl17173	AdoMet_MTases superfamily
	Mucam1_8607_transcript_GAN09185	superfamily	227952	64	212	0.003466	39.2698	cl35042	COG5665 superfamily
	Rhich1_14842_A14829	superfamily	227278	541	681	0.000575	43.1721	cl34844	EnvC superfamily
	MoalAD072_1_9894_BGZ68_009999_T1	superfamily	227455	314	428	3.21E-16	76.5789	cl34916	FRQ1 superfamily
	MospGBA30_1_5037_BGX28_005067_T1	superfamily	227455	211	343	3.7E-16	76.1937	cl34916	FRQ1 superfamily
	HabiNA12553_1_6962_BGZ52_006998_T1	specific	430048	367	395	1.6E-07	48.1628	pfam08518	GIT_SHD
	HabiNA12553_1_6962_BGZ52_006998_T1	specific	430048	415	440	2.04E-05	42.3848	pfam08518	GIT_SHD
	HabiNA12553_1_6962_BGZ52_006998_T1	superfamily	432399	1013	1142	4.51E-14	69.2516	cl20096	GIT1_C superfamily
	Mucci3_1562578_fgenesh1_pm_3_792	superfamily	452895	449	520	0.000342	44.0805	cl28910	MFS superfamily
	MoveAD079_1_8320_CPB97_008399_T1	superfamily	215476	3	471	6.9E-159	480.312	cl33589	PLN02881 superfamily
	Mycind1_633139_estExt_Genewise1_C_7_t20141	superfamily	236669	220	308	0.000303	44.3055	cl35903	PRK10263 superfamily
	Rhisto1_723857_gm1_1012_g	specific	411031	210	351	4.36E-31	119.39	cd21044	Rab11BD_RAB3IP_like
	Spoumb1_588632_estExt_Genemark1_C_670009	specific	425753	419	623	1.85E-59	202.593	pfam00566	RabGAP-TBC
	Rhisto1_772580_estExt_Genemark1_C_90224	specific	425753	358	561	5.52E-61	206.445	pfam00566	RabGAP-TBC
	MoalAD072_1_9894_BGZ68_009999_T1	superfamily	273167	584	701	3.4E-05	46.8119	cl36702	rad23 superfamily
	Rhisto1_723857_gm1_1012_g	superfamily	428938	127	178	3.34E-08	51.0313	cl05764	Sec2p superfamily
	HabiNA12553_1_6962_BGZ52_006998_T1	superfamily	224117	541	729	8.35E-07	53.5648	cl34174	Smc superfamily
	Rhisto1_723857_gm1_1012_g	superfamily	224117	11	210	0.000887	42.394	cl34174	Smc superfamily
	Spoumb1_588632_estExt_Genemark1_C_670009	superfamily	224117	716	896	3.1E-06	51.2536	cl34174	Smc superfamily
	Rhich1_14842_A14829	superfamily	224117	522	806	0.006045	40.0828	cl34174	Smc superfamily
	Muccirc1_2940_transcript_EPB90136	superfamily	224117	863	1035	1.63E-05	49.3276	cl34174	Smc superfamily
	Mucci3_1562578_fgenesh1_pm_3_792	superfamily	274008	772	965	0.00834	40.4251	cl37069	SMC_prok_B superfamily
Muc-Velvet1	Bifad1_3271_transcript_OZJ04790	specific	235422	132	631	1.6E-163	486.588	PRK05342	clpX
	HapNA10739_1_4850_BGZ50_004910_T1	superfamily	455861	10	108	0.00197	40.5792	cl42516	dnaA superfamily
	HapNA10996_1_682_BGZ51_000693_T1	superfamily	455861	10	108	0.00197	40.5792	cl42516	dnaA superfamily
	Rhich1_208_A00137	superfamily	433564	139	395	6.17E-26	109.26	cl16461	Glyco_transf_49 superfamily
	Chocucu1_477852_estExt_Genewise1Plus_C_160108	superfamily	433564	166	391	2.83E-22	98.4749	cl16461	Glyco_transf_49 superfamily
	Mucfus1_366818_Mf86594_m_Sexual	superfamily	433564	281	496	8.23E-16	79.2149	cl16461	Glyco_transf_49 superfamily
	Mycind1_728354_gm1_8311_g	superfamily	433564	165	407	8.29E-24	103.097	cl16461	Glyco_transf_49 superfamily
	Cokrec1_456470_estExt_Genewise1_C_170110	superfamily	433564	138	376	2.34E-14	74.5925	cl16461	Glyco_transf_49 superfamily
	Muclan1_10587_Ml20231_m_Sexual	superfamily	433564	173	403	2.23E-25	107.334	cl16461	Glyco_transf_49 superfamily
	Parpar1_385187_estExt_Genewise1Plus_C_310029	superfamily	433564	150	395	6.77E-24	103.097	cl16461	Glyco_transf_49 superfamily
	Sakvas1_6240_SVAS_05245_R0	superfamily	433564	235	458	7.67E-19	88.4597	cl16461	Glyco_transf_49 superfamily
	Pilano1_435905_estExt_fgenesh1_pm_C_270051	superfamily	433564	142	382	4.89E-26	109.26	cl16461	Glyco_transf_49 superfamily
	Lichy1_166366_estExt_Genewise1Plus_C_1430014	superfamily	433564	197	437	1.61E-29	119.661	cl16461	Glyco_transf_49 superfamily
	Kircor1_436390_fgenesh1_pm_66_20	superfamily	433564	141	382	2.3E-31	124.668	cl16461	Glyco_transf_49 superfamily
	Radspe1_502696_e_gw1_12_236_1	superfamily	433564	213	452	1.08E-24	105.408	cl16461	Glyco_transf_49 superfamily
	Lenpar1_767464_e_gw1_4_51_1	superfamily	433564	209	443	7.88E-12	66.8885	cl16461	Glyco_transf_49 superfamily
	Rhior3_7750_RO3G_07452	superfamily	433564	140	383	6.79E-26	108.875	cl16461	Glyco_transf_49 superfamily
	Amyrou1_518916_e_gw1_13_308_1	superfamily	433564	140	383	3E-25	106.949	cl16461	Glyco_transf_49 superfamily
	Rhimi_ATCC52814_1_208481_e_gw1_83_107_1	superfamily	433564	139	395	1.48E-24	105.023	cl16461	Glyco_transf_49 superfamily
	Rhimi_ATCC11559_1_178732_e_gw1_64_113_1	superfamily	433564	139	395	4.89E-26	109.26	cl16461	Glyco_transf_49 superfamily
	Umbsp_AD052_1_274229_e_gw1_6_468_1	superfamily	433564	224	470	1.12E-25	108.49	cl16461	Glyco_transf_49 superfamily
	Umban1_299519_e_gw1_6_1140_1	superfamily	433564	224	470	4.03E-23	100.786	cl16461	Glyco_transf_49 superfamily

	Lenzyc1_741989_e_gw1_7_1655_1	superfamily	433564	205	441	2.06E-16	80.7557	cl16461	Glyco_transf_49 superfamily
	Umbelo1_419457_e_gw1_2_1071_1	superfamily	433564	241	487	2.54E-24	104.638	cl16461	Glyco_transf_49 superfamily
	Blatri_F921_1_387317_e_gw1_30_219_1	superfamily	433564	149	374	4.62E-24	103.482	cl16461	Glyco_transf_49 superfamily
	Blatri_F986_2_341746_e_gw1_6_168_1	superfamily	433564	149	374	4.62E-24	103.482	cl16461	Glyco_transf_49 superfamily
	Blatri1_332830_e_gw1_47_125_1	superfamily	433564	149	374	4.62E-24	103.482	cl16461	Glyco_transf_49 superfamily
	Mucmuc1_414754_e_gw1_16_121_1	superfamily	433564	151	375	7.59E-23	100.016	cl16461	Glyco_transf_49 superfamily
	Gilper1_580993_fgenesh1_pm_41_12	superfamily	433564	156	382	5.55E-25	106.179	cl16461	Glyco_transf_49 superfamily
	Ellano2_366625_e_gw1_11_77_1	superfamily	433564	212	449	4.49E-12	67.6589	cl16461	Glyco_transf_49 superfamily
	Kircor1_262296_e_gw1_3_666_1	superfamily	433564	148	388	3.06E-16	80.3705	cl16461	Glyco_transf_49 superfamily
	PnitS607_1_795931_e_gw1_1_601_1	superfamily	433564	133	354	1.21E-08	56.8733	cl16461	Glyco_transf_49 superfamily
	Phybl_L51_1_1629923_e_gw1_1_3721_1	superfamily	433564	104	340	1.81E-14	74.5925	cl16461	Glyco_transf_49 superfamily
	PhyblU21_2_1728340_e_gw1_1_1599_1	superfamily	433564	104	340	1.81E-14	74.5925	cl16461	Glyco_transf_49 superfamily
	Benpoi1_490130_e_gw1_7_1160_1	superfamily	433564	148	386	1.14E-20	93.4673	cl16461	Glyco_transf_49 superfamily
	Ellano2_368721_e_gw1_13_126_1	superfamily	433564	170	401	1.53E-23	101.942	cl16461	Glyco_transf_49 superfamily
	Phybla1_1516203_e_gw1_1_191_1	superfamily	433564	104	340	1.81E-14	74.5925	cl16461	Glyco_transf_49 superfamily
	Chabre1_168301_fgenesh1_pm_1_175	superfamily	433564	151	422	5.58E-25	106.179	cl16461	Glyco_transf_49 superfamily
	Umbisa1_492395_e_gw1_7_191_1	superfamily	433564	132	378	1.31E-28	116.964	cl16461	Glyco_transf_49 superfamily
	Thaele1_339597_e_gw1_11_512_1	superfamily	433564	138	378	6.21E-24	103.097	cl16461	Glyco_transf_49 superfamily
	Helpul1_645883_estExt_fgenesh1_pg_C_70177	superfamily	433564	139	379	5.88E-24	103.097	cl16461	Glyco_transf_49 superfamily
	Mycafr1_827687_e_gw1_12_912_1	superfamily	433564	166	398	1.04E-22	99.6305	cl16461	Glyco_transf_49 superfamily
	Zyghet1_371117_e_gw1_150_14_1	superfamily	433564	129	394	1.28E-26	111.186	cl16461	Glyco_transf_49 superfamily
	Dicrob1_482721_e_gw1_3_671_1	superfamily	433564	118	357	1.16E-20	93.4673	cl16461	Glyco_transf_49 superfamily
	Pilumb1_841938_fgenesh1_pm_5_102	superfamily	433564	139	361	2.52E-14	74.2073	cl16461	Glyco_transf_49 superfamily
	MoreMES2147_1_9744_BGZ65_009858_T1	superfamily	425360	479	586	1.3E-05	48.5014	cl41729	KLF1_2_4_N superfamily
	MohuKOD1050_1_2835_BG004_002842_T1	superfamily	425360	86	140	0.003478	40.027	cl41729	KLF1_2_4_N superfamily
	Morve1_4122_MVEG_11562T0	superfamily	425360	65	112	0.006793	38.4862	cl41729	KLF1_2_4_N superfamily
	MoclAM1000_1_2055_BGZ81_002095_T1	superfamily	227458	133	214	0.00232	39.6428	cl44195	MAK16 superfamily
	MoalCK1249_1_10067_BGZ70_010154_T1	superfamily	223021	112	251	8.93E-05	45.6997	cl33720	PHA03247 superfamily
	MobeAD1040_1_4573_BGZ76_004613_T1	superfamily	223021	17	134	0.009222	38.3809	cl33720	PHA03247 superfamily
	MochAD033_1_4348_BGZ79_004375_T1	superfamily	332875	41	113	0.001701	39.2241	cl28054	Pox_Ag35 superfamily
	MochN2769_1_6353_BGZ80_006390_T1	superfamily	332875	41	113	0.001568	39.2241	cl28054	Pox_Ag35 superfamily
	HaliN2525_1_3335_BGZ46_003354_T1	superfamily	236304	345	479	0.001075	42.0823	cl35718	PRK08581 superfamily
	MoelGBA34_1_10473_BGZ88_010541_T1	superfamily	236669	49	206	0.003665	40.4535	cl35903	PRK10263 superfamily
	MopoKOD948_1_198_BG011_000201_T1	superfamily	236669	10	142	0.000103	45.0759	cl35903	PRK10263 superfamily
	Mocy1230_1_7884_BGZ83_007970_T1	superfamily	237171	163	275	0.001035	41.8131	cl36163	PRK12678 superfamily
	Phybl2_178806_estExt_Genemark1_C_10692	specific	185485	397	568	1.4E-118	347.799	PTZ00156	PTZ00156
	Rhimi59_2_1976649_estExt_fgenesh1_pm_C_5_t10462	specific	425753	312	505	6.02E-36	135.568	pfam00566	RabGAP-TBC
	Rhimi59_2_1976649_estExt_fgenesh1_pm_C_5_t10462	superfamily	224117	683	885	1.09E-07	56.2612	cl34174	Smc superfamily
	HaspN3175_1_2406_BGZ49_002416_T1	superfamily	451284	78	121	0.00556	39.0055	cl21514	TauE superfamily
	MoscN6426_1_8746_BG015_008793_T1	specific	428395	356	522	1.47E-58	191.265	pfam05255	UPF0220
Muc-Velvet2	Kircor1_416181_estExt_Genemark1_C_30348	specific	185498	234	446	2E-151	428.297	PTZ00173	PTZ00173
	Zyghet1_535231_gm1_15889_g	specific	185498	91	303	1.3E-146	410.577	PTZ00173	PTZ00173
Muc-Velvet3	HapNA10996_1_7044_BGZ51_00713	superfamily	448367	982	1429	0	664.306	cl11961	ALDH-SF superfamily
	Rhicl1_17367_transcript_GBC05587	superfamily	455732	677	756	0.001355	41.9088	cl42387	dermokine superfamily
	MorGBAus27b_1_177841_CE177840_	superfamily	455861	711	891	0.000925	42.8904	cl42516	dnaA superfamily
	MospGBA30_1_471_BGX28_000473_	superfamily	414561	350	427	0.000245	43.5963	cl05827	IpaD superfamily
	MospAD094_1_11432_BGX26_01149	superfamily	425360	895	987	0.001597	41.953	cl41729	KLF1_2_4_N superfamily
	RhiirA4_415180_fgenesh1_pg_70_13	superfamily	223021	248	682	1.64E-06	51.8629	cl33720	PHA03247 superfamily
	RhiirA5_398311_estExt_fgenesh1_pg_	superfamily	223021	248	682	6.14E-06	50.3221	cl33720	PHA03247 superfamily
	RhiirB3_523613_estExt_Genemark1_	superfamily	223021	248	682	6.14E-06	50.3221	cl33720	PHA03247 superfamily
	RhiirA1_1_531809_estExt_Genemark1	superfamily	223021	248	682	6.14E-06	50.3221	cl33720	PHA03247 superfamily
	MozyPUSTF9C_1_569_FBU30_00057	superfamily	223021	425	703	0.00547	40.6921	cl33720	PHA03247 superfamily
	MoalAD071_1_6289_BGZ67_006380_	superfamily	223021	504	931	4.52E-09	61.1077	cl33720	PHA03247 superfamily
	MoalAD071_1_6289_BGZ67_006380_	superfamily	223021	222	705	0.000119	46.4701	cl33720	PHA03247 superfamily
	MoalGBA31_1_6683_BGZ72_006758_	superfamily	223021	256	759	4.15E-06	51.0925	cl33720	PHA03247 superfamily
	Morve1_3632_MVEG_11289T0	superfamily	223021	535	909	0.009982	39.9217	cl33720	PHA03247 superfamily
	MoanKOD1229_1_5537_BGZ75_0056	superfamily	223021	442	869	1.00E-07	56.4853	cl33720	PHA03247 superfamily
	MoanKOD1229_1_5537_BGZ75_0056	superfamily	223021	160	643	0.001236	43.0033	cl33720	PHA03247 superfamily
	EctrN5525_1_10927_BX616_010970_	superfamily	223021	302	616	8.72E-05	46.8553	cl33720	PHA03247 superfamily
	HapNA10739_1_8313_BGZ50_00841	superfamily	223021	448	799	1.53E-06	52.6333	cl33720	PHA03247 superfamily
	HapNA10739_1_8313_BGZ50_00841	superfamily	223021	343	475	0.0037	41.4625	cl33720	PHA03247 superfamily
	HapNA10996_1_7044_BGZ51_00713	superfamily	223021	423	783	2.60E-08	59.1817	cl33720	PHA03247 superfamily
	HapNA10996_1_7044_BGZ51_00713	superfamily	223021	327	459	0.000638	44.5441	cl33720	PHA03247 superfamily
	MospNVP41_1_7091_BGX33_007139	superfamily	223021	355	753	0.006411	40.6921	cl33720	PHA03247 superfamily
	MocaN2610_1_11798_BGZ47_01185	superfamily	223021	342	617	0.000555	44.1589	cl33720	PHA03247 superfamily
	Moralp1_1_163976_CE163975_10544	superfamily	223039	540	904	1.62E-06	52.4809	cl33723	PHA03307 superfamily

	MoalCK1249_1_2586_BGZ70_002601	superfamily	223039	280	642	1.99E-06	51.3253	cl33723	PHA03307 superfamily
	MoveTTC192_1_2685_CPC16_00271(	superfamily	223039	573	904	0.007272	40.1545	cl33723	PHA03307 superfamily
	MospAM989_1_4523_BGX27_004572_	superfamily	223039	408	836	0.000378	44.3917	cl33723	PHA03307 superfamily
	HaspN3175_1_1853_BGZ49_001862_	superfamily	223039	544	779	0.002481	41.6953	cl33723	PHA03307 superfamily
	MoglAD054_1_713_BGZ98_000713_T	superfamily	223039	131	479	0.001233	42.8509	cl33723	PHA03307 superfamily
	MospAD031_1_1915_BGX23_001930_	superfamily	223039	509	851	0.000197	45.1621	cl33723	PHA03307 superfamily
	Moralp1_1_163976_CE163975_10544	superfamily	236090	377	580	0.006513	40.3545	cl35613	PRK07764 superfamily
	MoalGBA31_1_6683_BGZ72_006758_	superfamily	236090	575	967	0.001481	42.6657	cl35613	PRK07764 superfamily
	DiorN22417_1_10925_BGZ58_011001	superfamily	236090	313	534	0.000204	44.5917	cl35613	PRK07764 superfamily
	MospGBA30_1_471_BGX28_000473_	superfamily	236669	401	630	0.005445	40.0683	cl35903	PRK10263 superfamily
	MochAD033_1_9183_BGZ79_009253_	superfamily	236669	467	561	0.006622	40.4535	cl35903	PRK10263 superfamily
	Mosp14UC_1_11275_BGW39_011357	superfamily	183558	148	265	0.0019	39.852	cl32772	PRK12495 superfamily
	MozyPUSTF9C_1_569_FBU30_00057	superfamily	173412	690	819	8.69E-09	59.3843	cl31754	PTZ00121 superfamily
	RhiirA4_415180_fgenesh1_pg_70_13	superfamily	227709	648	763	0.008331	39.8743	cl34999	ROM1 superfamily
	RhiirA5_398311_estExt_fgenesh1_pg_	superfamily	227709	648	763	0.008767	39.8743	cl34999	ROM1 superfamily
	RhiirB3_523613_estExt_Genemark1_(	superfamily	227709	648	763	0.008767	39.8743	cl34999	ROM1 superfamily
	RhiirA1_1_531809_estExt_Genemark1	superfamily	227709	648	763	0.008767	39.8743	cl34999	ROM1 superfamily
Muc-VosA	MoelGBA34_1_4074_BGZ88_004102_T1	superfamily	130009	141	427	0.001049	41.8922	cl30043	2a38euk superfamily
	MoelNVP5_1_5204_BGZ90_005257_T1	superfamily	130009	71	357	0.000568	42.6626	cl30043	2a38euk superfamily
	MomuN6456_1_10449_BGZ54_010510_T1	superfamily	455861	203	475	0.005379	39.8088	cl42516	dnaA superfamily
	MohoCK413_1_8428_BG003_008522_T1	superfamily	455861	172	437	9.77E-05	44.8164	cl42516	dnaA superfamily
	MomiNVP1_1_6279_BG006_006355_T1	superfamily	455861	151	403	0.001011	41.7348	cl42516	dnaA superfamily
	MoepAD058_1_3473_BGZ92_003519_T1	superfamily	455861	233	428	0.003851	39.8088	cl42516	dnaA superfamily
	MoveTTC192_1_360_CPC16_000366_T1	superfamily	455861	147	325	0.000583	42.5052	cl42516	dnaA superfamily
	Morve1_6557_MVEG_03275T0	superfamily	455861	187	338	0.007255	38.6532	cl42516	dnaA superfamily
	MospKOD1030_1_2085_BGZ74_002107_T1	superfamily	455861	217	305	0.000895	41.3496	cl42516	dnaA superfamily
	Morve1_11098_MVEG_07715T0	superfamily	455861	172	350	0.000448	42.8904	cl42516	dnaA superfamily
	MoveTTC192_1_6456_CPC16_006540_T1	superfamily	455861	187	338	0.002572	41.3496	cl42516	dnaA superfamily
	MospGBA43_1_4364_BGX31_004398_T1	superfamily	455861	274	406	0.004175	39.8088	cl42516	dnaA superfamily
	MospNVP41_1_5111_BGX33_005149_T1	superfamily	455861	251	468	0.001724	40.9644	cl42516	dnaA superfamily
	MospAD011_1_1476_BGX21_001484_T1	superfamily	455861	129	361	0.000172	43.6608	cl42516	dnaA superfamily
	MozyN2592_1_10324_EC991_010525_T1	superfamily	235553	451	478	0.002972	40.6105	cl35361	dnaE2 superfamily
	EctrN5525_1_7590_BX616_007627_T1	superfamily	223560	958	1050	2.27E-29	121.193	cl33903	DnaJ superfamily
	MoveTTC192_1_6456_CPC16_006540_T1	superfamily	223560	802	900	3.71E-27	114.259	cl33903	DnaJ superfamily
	MoalGBA31_1_7938_BGZ72_008032_T1	superfamily	223560	968	1066	1.16E-26	113.104	cl33903	DnaJ superfamily
	MoalAD071_1_4674_BGZ67_004744_T1	superfamily	223560	742	840	2.06E-26	111.948	cl33903	DnaJ superfamily
	HaliN2525_1_8286_BGZ46_008352_T1	superfamily	223560	903	993	6.74E-28	116.571	cl33903	DnaJ superfamily
	MoalCK1249_1_3613_BGZ70_003637_T1	superfamily	223560	675	773	1.39E-25	109.252	cl33903	DnaJ superfamily
	MohuKOD1050_1_7117_BG004_007147_T1	superfamily	428666	243	319	0.0044	39.8483	cl12320	DUF863 superfamily
	MospGBA35_1_3660_BGX29_003698_T1	superfamily	406402	266	406	0.003182	39.5277	cl25765	G_path_suppress superfamily
	Licra1_1196_LRAMOSA06746_1	superfamily	282904	244	380	0.001737	40.67	cl37540	Herpes_BLLF1 superfamily
	EctrN5525_1_7590_BX616_007627_T1	superfamily	274350	573	989	1.13E-15	82.0536	cl37187	PEP_TPR_lipo superfamily
	MoveTTC192_1_6456_CPC16_006540_T1	superfamily	274350	419	644	4.51E-12	70.1124	cl37187	PEP_TPR_lipo superfamily
	MoalGBA31_1_7938_BGZ72_008032_T1	superfamily	274350	583	1024	7.67E-10	63.1788	cl37187	PEP_TPR_lipo superfamily
	MoalAD071_1_4674_BGZ67_004744_T1	superfamily	274350	339	741	2.2E-10	64.3344	cl37187	PEP_TPR_lipo superfamily
	HaliN2525_1_8286_BGZ46_008352_T1	superfamily	274350	518	935	2.82E-16	83.9796	cl37187	PEP_TPR_lipo superfamily
	MoalCK1249_1_3613_BGZ70_003637_T1	superfamily	274350	305	674	4.73E-11	66.6456	cl37187	PEP_TPR_lipo superfamily
	MomuN6456_1_10449_BGZ54_010510_T1	superfamily	223021	356	611	0.00012	45.3145	cl33720	PHA03247 superfamily
	MoveAD079_1_11444_CPB97_011567_T1	superfamily	223021	204	407	0.002422	40.6921	cl33720	PHA03247 superfamily
	MospAD010_1_11119_BGX20_011197_T1	superfamily	223021	268	551	3.01E-05	47.2405	cl33720	PHA03247 superfamily
	MospAD011_1_3912_BGX21_003934_T1	superfamily	223021	268	551	3.01E-05	47.2405	cl33720	PHA03247 superfamily
	MospGBA30_1_6663_BGX28_006700_T1	superfamily	223021	277	558	2.17E-05	47.6257	cl33720	PHA03247 superfamily
	MospAD031_1_7607_BGX23_007648_T1	superfamily	223021	189	520	0.002453	41.0773	cl33720	PHA03247 superfamily
	Liccor1_1106_LCOR_01054_1	superfamily	223021	247	416	0.002021	40.6921	cl33720	PHA03247 superfamily
	MoalGBA31_1_7938_BGZ72_008032_T1	superfamily	223039	201	558	0.000108	46.3177	cl33723	PHA03307 superfamily
	MoalAD071_1_4674_BGZ67_004744_T1	superfamily	223039	201	332	0.005344	40.5397	cl33723	PHA03307 superfamily

	MoscN6426_1_827_BG015_000832_T1	superfamily	223039	217	433	5.58E-05	46.3177	cl33723	PHA03307 superfamily
	MoeNVP64_1_418301_estExt_fgenesh1_pg_C_210186	superfamily	223039	217	435	0.007642	39.3841	cl33723	PHA03307 superfamily
	MoeAD073_1_246259_CE246258_1128	superfamily	223039	217	519	0.000188	44.3917	cl33723	PHA03307 superfamily
	MozyN2592_1_10324_EC991_010525_T1	superfamily	223039	217	433	0.001952	41.3101	cl33723	PHA03307 superfamily
	Mosp14UC_1_9459_BGW39_009529_T1	superfamily	223039	217	492	0.009202	38.9989	cl33723	PHA03307 superfamily
	MoelGBA40_1_3610_BGZ89_003625_T1	superfamily	223039	212	430	0.005748	39.7693	cl33723	PHA03307 superfamily
	MoclN2760_1_9201_EDD11_009369_T1	superfamily	236669	242	438	9.66E-06	48.9279	cl35903	PRK10263 superfamily
	MogaAD045_1_976_BGZ96_000986_T1	superfamily	183558	187	296	0.000526	41.778	cl32772	PRK12495 superfamily
	MohyN2591_1_9111_EC957_009258_T1	superfamily	183558	203	312	0.003328	39.4668	cl32772	PRK12495 superfamily
	HaliN2525_1_8286_BGZ46_008352_T1	superfamily	185594	236	343	1.85E-05	48.9179	cl33180	PTZ00395 superfamily
	HaspN3175_1_8679_BGZ49_008754_T1	superfamily	185594	236	343	0.000408	43.1399	cl33180	PTZ00395 superfamily
	MohoAD009_1_9167_BG000_009233_T1	superfamily	185628	197	435	1.69E-05	47.7622	cl33186	PTZ00449 superfamily
	MoclAM1000_1_11139_BGZ81_011303_T1	superfamily	185628	196	434	4.68E-05	46.2214	cl33186	PTZ00449 superfamily
	MomiAD069_1_7424_BG005_007508_T1	superfamily	185628	196	431	5.46E-06	49.303	cl33186	PTZ00449 superfamily
	MoelGBA34_1_8169_BGZ88_008227_T1	superfamily	227476	841	1039	1.94E-15	80.9871	cl34920	REB1 superfamily
	MocaN2610_1_1478_BGZ47_001482_T1	superfamily	227476	1004	1126	4.32E-15	80.2167	cl34920	REB1 superfamily
	MogaAD045_1_7260_BGZ96_007323_T1	superfamily	227476	921	1079	9.14E-17	85.2243	cl34920	REB1 superfamily
	MospNVP41_1_6394_BGX33_006438_T1	superfamily	227476	881	1079	9.71E-18	88.3059	cl34920	REB1 superfamily
	MospGBA35_1_2582_BGX29_002610_T1	superfamily	227476	885	1083	1.04E-17	88.3059	cl34920	REB1 superfamily
	MobeAD1040_1_9677_BGZ76_009768_T1	superfamily	227709	235	453	0.000255	43.7263	cl34999	ROM1 superfamily
	MozyPUSTF9C_1_6101_FBU30_006165_T1	superfamily	227709	234	426	0.00047	42.9559	cl34999	ROM1 superfamily
	MospAD094_1_6996_BGX26_007033_T1	superfamily	227709	194	442	5.13E-05	46.0375	cl34999	ROM1 superfamily
	MochAD033_1_2011_BGZ79_002020_T1	superfamily	227709	151	391	0.000287	43.3411	cl34999	ROM1 superfamily
	MospAD010_1_11385_BGX20_011465_T1	superfamily	227709	160	402	0.000243	43.7263	cl34999	ROM1 superfamily
	MoveTTC192_1_6456_CPC16_006540_T1	specific	425728	748	777	0.008095	34.7053	pfam00515	TPR_1
Pez-VeA	Altca1_103524_ACSCTG_1027_jigsaw_mRNA_1	superfamily	197891	465	584	0.003162	39.0025	cl33250	Amelogenin superfamily
	Altcr1_108859_ACRCTG_236_jigsaw_mRNA_1	superfamily	197891	465	584	0.003162	39.0025	cl33250	Amelogenin superfamily
	Asppust1_238666_fgenesh1_pm_196_18	superfamily	427171	270	448	1.05E-06	53.2318	cl38111	Atrophin-1 superfamily
	Asplac1_443942_gm1_3719_g	superfamily	427171	368	492	0.000198	44.3722	cl38111	Atrophin-1 superfamily
	Aspph1_7605_CE7604_2395	superfamily	427171	354	478	0.000304	43.6018	cl38111	Atrophin-1 superfamily
	Pse1611_1_713483_gm1_6625_g	superfamily	427171	273	541	1.55E-05	48.2242	cl38111	Atrophin-1 superfamily
	Aaoar1_203095_CE203094_8103	superfamily	427171	274	559	4.59E-06	49.765	cl38111	Atrophin-1 superfamily
	Neopa1_2412_UCRNP2_10414m_01	superfamily	427171	274	558	0.000314	43.987	cl38111	Atrophin-1 superfamily
	Macpha1_378862_CE378861_30934	superfamily	427171	274	494	1.24E-09	61.321	cl38111	Atrophin-1 superfamily
	Macph1_1625_MPH_01661m_01	superfamily	427171	274	494	1.24E-09	61.321	cl38111	Atrophin-1 superfamily
	Aspaur1_172868_fgenesh1_pm_26_36	superfamily	427171	237	520	0.000209	45.5278	cl38111	Atrophin-1 superfamily
	Lopmy1_565459_gm1_11464_g	superfamily	427171	287	517	2.56E-09	61.7062	cl38111	Atrophin-1 superfamily
	DelFL0756_1_194243_CE194242_34694	superfamily	427171	264	537	0.001029	42.061	cl38111	Atrophin-1 superfamily
	Mytre1_279359_CE279358_4366	superfamily	427171	287	540	3.34E-07	53.617	cl38111	Atrophin-1 superfamily
	Photr1_204921_CE204920_14070	superfamily	427171	254	543	0.001393	41.6758	cl38111	Atrophin-1 superfamily
	PhaPMI808_730417_fgenesh1_pg_54_74	superfamily	427171	250	526	1.96E-08	57.469	cl38111	Atrophin-1 superfamily
	Clamic1_259668_CE259667_15531	superfamily	427171	316	491	0.001127	42.061	cl38111	Atrophin-1 superfamily
	Lashir1_366288_CE366287_39595	superfamily	427171	277	490	1.91E-05	47.4538	cl38111	Atrophin-1 superfamily
	Daces1_451237_e_gw1_18_540_1	superfamily	427171	322	467	1.21E-07	54.3874	cl38111	Atrophin-1 superfamily
	Cypeu1_121696_HMPREF1541_10303T0	superfamily	427171	277	378	0.001842	40.9054	cl38111	Atrophin-1 superfamily
	Aspave1_126501_fgenesh1_pm_7_1	superfamily	448379	771	898	0.008752	38.8599	cl12013	BAR superfamily
	Dibbae1_3888_DIBA_008099_RA	superfamily	434947	305	526	0.002244	40.5318	cl25809	CiPC superfamily
	Asppeni1_123690_CE123689_4995	superfamily	430824	338	455	0.009304	38.6522	cl40852	CytochromB561_N superfamily
	Aspaura1_203970_gm1_7400_g	specific	436606	951	989	0.001313	39.8808	pfam18595	DHR10
	Aspamy1_170918_fgenesh1_pm_20_14	specific	436606	934	975	0.001364	39.8808	pfam18595	DHR10
	Amore1_31023_fgenesh1_pg_12_171	superfamily	455861	295	515	2.16E-05	47.1276	cl42516	dnaA superfamily
	Leuca1_1077986_fgenesh1_pg_74_56	superfamily	455861	320	458	0.001026	42.12	cl42516	dnaA superfamily
	Chalo1_235725_CE235724_44958	superfamily	455861	253	443	0.001418	40.9644	cl42516	dnaA superfamily
	Hyafin1_976942_MIX16197_17877_50	superfamily	455861	298	495	6.28E-05	45.5868	cl42516	dnaA superfamily

MelPMI1271_1_383735_fgenesh1_pg_121_5	superfamily	455861	380	512	0.005337	39.4236	cl42516	dnaA superfamily
Cersam1_421_CE420_6839	superfamily	455861	260	481	2.94E-05	46.7424	cl42516	dnaA superfamily
Podtet1_384337_MIX19354_7401_42	superfamily	455861	261	429	0.000841	41.7348	cl42516	dnaA superfamily
Podan3_206066_mRNA_4894	superfamily	455861	271	417	0.000846	42.12	cl42516	dnaA superfamily
Podans1_153444_CE153443_8149	superfamily	455861	271	417	0.000846	42.12	cl42516	dnaA superfamily
Ant016_1_30474_CE30473_29500	superfamily	455861	287	472	0.002201	40.5792	cl42516	dnaA superfamily
Pezci1_717949_fgenesh1_pm_17_128	superfamily	455861	343	511	0.000579	42.5052	cl42516	dnaA superfamily
Colgo1_692230_fgenesh1_pm_11_105	superfamily	455861	281	497	0.001283	41.3496	cl42516	dnaA superfamily
Colsa1_945018_mrna_7774	superfamily	455861	329	504	7.22E-05	45.5868	cl42516	dnaA superfamily
Colsi1_979553_mrna_7673	superfamily	455861	316	498	0.000169	44.4312	cl42516	dnaA superfamily
Colsp1_7376_mrna_16201	superfamily	455861	345	498	0.003181	40.194	cl42516	dnaA superfamily
Colta1_15813_mrna_3524	superfamily	455861	316	498	0.001244	41.7348	cl42516	dnaA superfamily
Colfi1_284943_mRNA9763	superfamily	455861	316	498	0.000573	42.5052	cl42516	dnaA superfamily
Collup1_786_mrna_786	superfamily	455861	316	498	0.000483	42.8904	cl42516	dnaA superfamily
Colpa1_8441_mrna_16581	superfamily	455861	517	699	0.000141	45.2016	cl42516	dnaA superfamily
Colab1_6480_mrna_6480	superfamily	455861	316	498	0.000526	42.8904	cl42516	dnaA superfamily
Colco1_6075_mrna_14063	superfamily	455861	316	498	0.000478	42.8904	cl42516	dnaA superfamily
Colcu1_11654_mrna_11654	superfamily	455861	316	497	0.000511	42.8904	cl42516	dnaA superfamily
Phiglo1_437934_gm4_5830_g	superfamily	455861	294	417	3.1E-06	49.824	cl42516	dnaA superfamily
Collu1_485900_fgenesh1_pg_3_216	superfamily	455861	316	498	0.000462	42.8904	cl42516	dnaA superfamily
Colme1_6687_mrna_6687	superfamily	455861	316	498	0.000573	42.5052	cl42516	dnaA superfamily
Ophca1_2048_transcript_PHH75799	superfamily	455861	310	446	0.00153	40.9644	cl42516	dnaA superfamily
Horth1_4396_B0A50_05387	superfamily	455861	436	561	0.00441	40.194	cl42516	dnaA superfamily
Trihar1_784206_fgenesh1_kg_469_1_m_23631	superfamily	455861	256	420	0.000595	42.5052	cl42516	dnaA superfamily
Triinh1_59490_CE59489_41700	superfamily	455861	256	420	0.000501	42.8904	cl42516	dnaA superfamily
Trien1_347454_CE347453_11123	superfamily	455861	256	421	0.001969	40.9644	cl42516	dnaA superfamily
Triha1_95531_e_gw1_14_392_1	superfamily	455861	256	420	0.000593	42.5052	cl42516	dnaA superfamily
XylPMI506_126167_CE126166_7602	superfamily	455861	265	439	1.26E-05	47.898	cl42516	dnaA superfamily
TriharT22_1_217059_CE217058_70309	superfamily	455861	256	420	0.000741	42.5052	cl42516	dnaA superfamily
Metac1_39_MAC_00039m_01	superfamily	455861	281	458	7.67E-05	45.2016	cl42516	dnaA superfamily
Metma1_543_XM_014727519_1	superfamily	455861	281	458	0.000588	42.5052	cl42516	dnaA superfamily
Plecucu2_284635_MIX11132_1127_34	superfamily	455861	247	424	0.000239	44.046	cl42516	dnaA superfamily
Trigui1_9566_transcript_OPB39752	superfamily	455861	282	447	0.000416	43.2756	cl42516	dnaA superfamily
MelPMI1271_1_383735_fgenesh1_pg_121_5	superfamily	452645	252	318	0.006232	38.5687	cl26593	DUF2076 superfamily
Lopmy1_565459_gm1_11464_g	superfamily	225711	937	1112	0.005758	41.0523	cl34561	FimV superfamily
Zymps1_795199_Zp221_scaffold_1008_00003	superfamily	367362	301	459	0.002325	41.0866	cl26620	Glutenin_hmw superfamily
Marbr1_2001_MBM_05547	superfamily	282904	1729	1898	0.006821	41.4404	cl37540	Herpes_BLLF1 superfamily
Foner1_1692_transcript_OAP65721	superfamily	237030	374	499	0.005545	39.8738	cl36091	kgd superfamily
Gyrinf1_909834_gm1_3287_g	superfamily	425360	312	390	0.00067	42.3382	cl41729	KLF1_2_4_N superfamily
Gyresc1_508977_gm1_648_g	superfamily	425360	312	390	0.002525	40.4122	cl41729	KLF1_2_4_N superfamily
Acrchr1_5707_ACRE_045050T0	superfamily	453886	348	387	0.003156	38.8078	cl38911	LGIC_TM superfamily
Exome1_108368_PV10_04372T0	superfamily	431183	19	143	0.006949	38.1376	cl10947	Miga superfamily
Claps1_1788_A1O5_07176T0	superfamily	451247	3	190	3.08E-81	259.63	cl21454	NADB_Rossmann superfamily
Penro1_2298_transcript_CDM28508	superfamily	450142	397	456	0.00504	39.3314	cl17037	NBD_sugar-kinase_HSP70_actin superfamily
Altso1_122073_ASLCTG_875_jigsaw_mRNA_3	specific	273555	801	1211	0	679.851	TIGR01326	OAH_OAS_sulfhy
Alttom1_120644_ATMCTG_3360_jigsaw_mRNA_2	specific	273555	735	1145	0	679.466	TIGR01326	OAH_OAS_sulfhy
Altcar1_110133_ACMCTG_2300_jigsaw_mRNA_2	specific	273555	778	1194	0	683.318	TIGR01326	OAH_OAS_sulfhy
Aspcla1_92310_CE92309_4979	superfamily	130689	343	503	0.001398	41.331	cl31127	PABP-1234 superfamily
Aspneoi1_250479_gm1_3109_g	superfamily	130689	325	453	2.85E-05	46.7238	cl31127	PABP-1234 superfamily
Umbpus1_106099_LPUS_04137T0	superfamily	130689	350	485	4.27E-06	49.8054	cl31127	PABP-1234 superfamily
Pleav1_81383_fgenesh1_pm_8_181	superfamily	130689	327	482	1.34E-06	51.3462	cl31127	PABP-1234 superfamily
Geocar1_719842_fgenesh1_pm_50_12	superfamily	130689	427	559	1.04E-05	48.6498	cl31127	PABP-1234 superfamily
Sarco1_317451_CE317450_30874	superfamily	130689	452	522	0.000258	44.0274	cl31127	PABP-1234 superfamily
Settur3_528262_fgenesh1_pg_32_50	superfamily	130689	609	751	0.000182	45.183	cl31127	PABP-1234 superfamily
Stasp1_126978_CE126977_15020	superfamily	130689	275	387	4.29E-06	49.4202	cl31127	PABP-1234 superfamily
Settu3_177499_Settu1_estExt_fgenesh1_pm_C_220032	superfamily	130689	328	470	0.000283	43.6422	cl31127	PABP-1234 superfamily
XylFL0594_14763_CE14762_41968	superfamily	130689	293	427	0.006051	39.0198	cl31127	PABP-1234 superfamily
Pezech1_525593_fgenesh1_pg_12_204	superfamily	401645	251	426	1.16E-07	55.042	cl37801	PAT1 superfamily
Terbo2_781483_e_gw1_9_227_1	superfamily	401645	256	402	0.000585	43.1008	cl37801	PAT1 superfamily
Tercla1_1267604_gm1_3364_g	superfamily	401645	256	402	0.000208	44.6416	cl37801	PAT1 superfamily
Morcra1_867469_fgenesh1_pm_7_197	superfamily	401645	360	561	0.000248	44.2564	cl37801	PAT1 superfamily
Patat1_938356_e_gw1_13_343_1	superfamily	401645	293	473	1.99E-06	50.8048	cl37801	PAT1 superfamily
Phcapit2_271027_CE271026_54591	superfamily	401645	273	392	0.000522	43.1008	cl37801	PAT1 superfamily
Phcap1_117802_CE117801_56220	superfamily	401645	273	392	0.000522	43.1008	cl37801	PAT1 superfamily
Pcapi1_510433_gm1_4462_g	superfamily	401645	273	392	0.000522	43.1008	cl37801	PAT1 superfamily
Phycap1_250501_e_gw1_5_1033_1	superfamily	401645	273	392	0.000522	43.1008	cl37801	PAT1 superfamily
Phycapi2_433598_e_gw1_5_1393_1	superfamily	401645	273	392	0.000522	43.1008	cl37801	PAT1 superfamily
Phcapi1_200860_e_gw1_4_1020_1	superfamily	401645	273	392	0.000522	43.1008	cl37801	PAT1 superfamily
Morana1_342348_CE342347_39092	superfamily	401645	360	560	0.000167	45.0268	cl37801	PAT1 superfamily
Aplpr1_305098_fgenesh1_pg_3_210	superfamily	401645	275	421	1.03E-08	58.1236	cl37801	PAT1 superfamily
Psemus1_11370_transcript_KXT14887	superfamily	401645	362	520	0.000372	43.486	cl37801	PAT1 superfamily

Psemus1_11371_transcript_KXT14889	superfamily	401645	362	520	0.000372	43.486	cl37801	PAT1 superfamily
Psemus1_11372_transcript_KXT14888	superfamily	401645	362	520	0.000372	43.486	cl37801	PAT1 superfamily
Pyrinf1_212081_CE212080_19094	superfamily	401645	292	453	5.73E-07	52.7308	cl37801	PAT1 superfamily
Loppic1_13540_CE13539_6173	superfamily	401645	227	379	7.4E-05	45.7972	cl37801	PAT1 superfamily
Stely1_1772_TW65_01665T0	superfamily	401645	304	438	0.002388	40.7896	cl37801	PAT1 superfamily
Phcit1_245431_fgenesh1_pm_21_22	superfamily	401645	264	470	1.09E-06	51.5752	cl37801	PAT1 superfamily
Pcit17464_601907_fgenesh1_pg_4_554	superfamily	401645	264	470	1.09E-06	51.5752	cl37801	PAT1 superfamily
Pcit129764_388096_e_gw1_7_1368_1	superfamily	401645	241	470	3.52E-06	50.0344	cl37801	PAT1 superfamily
Phcitr1_255200_fgenesh1_pg_5_104	superfamily	401645	241	470	3.59E-06	50.0344	cl37801	PAT1 superfamily
Fenfe1_103585_CE103584_36961	superfamily	401645	340	468	7.48E-06	48.8788	cl37801	PAT1 superfamily
Cucbe1_282185_e_gw1_3_3381_1	superfamily	401645	285	433	9.01E-07	51.9604	cl37801	PAT1 superfamily
Clael1_72108_CE72107_3255	superfamily	401645	273	415	1.86E-07	54.2716	cl37801	PAT1 superfamily
Dotsy1_28118_CE28117_2921	superfamily	401645	323	494	8.17E-05	45.7972	cl37801	PAT1 superfamily
Ophdi1_351325_estExt_Genewise1Plus_C_11_t30006	superfamily	401645	295	455	2.79E-06	50.4196	cl37801	PAT1 superfamily
Stano2_5188_SNOG_01807_3	superfamily	401645	247	455	8.3E-08	55.4272	cl37801	PAT1 superfamily
Elsamp1_87450_CE87449_477	superfamily	401645	323	495	2.29E-05	47.338	cl37801	PAT1 superfamily
Conioc1_1211268_gm1_1916_g	superfamily	401645	305	419	0.001881	41.1748	cl37801	PAT1 superfamily
CadmalM34_1_4977_IFR04_000311_1	superfamily	401645	296	467	0.000205	44.2564	cl37801	PAT1 superfamily
Psehy1_488347_fgenesh1_pg_13_267	superfamily	401645	269	413	6.03E-05	45.7972	cl37801	PAT1 superfamily
Cadsp1_529889_fgenesh1_pg_24_138	superfamily	401645	296	463	5.28E-05	46.1824	cl37801	PAT1 superfamily
Horth1_4396_B0A50_05387	superfamily	401645	314	466	3.26E-07	53.5012	cl37801	PAT1 superfamily
Altca1_103524_ACSCTG_1027_jigsaw_mRNA_1	superfamily	177328	526	645	0.001864	40.8089	cl26622	PHA01929 superfamily
Altcar1_110133_ACMCTG_2300_jigsaw_mRNA_2	superfamily	177328	523	642	0.000335	44.2757	cl26622	PHA01929 superfamily
Altcr1_108859_ACRCTG_236_jigsaw_mRNA_1	superfamily	177328	526	645	0.001864	40.8089	cl26622	PHA01929 superfamily
Altma1_116654_AMRCTG_3100_jigsaw_mRNA_1	superfamily	177328	390	509	0.000969	41.5793	cl26622	PHA01929 superfamily
Asptr1_106461_CE106460_13877	superfamily	222997	342	494	0.005029	39.7459	cl33716	PHA03132 superfamily
Aspacri1_210844_gm1_8769_g	superfamily	223021	251	498	1.82E-05	49.5517	cl33720	PHA03247 superfamily
Aspsim1_1_216309_MIX5629_34_45	superfamily	223021	252	493	1.53E-06	53.0185	cl33720	PHA03247 superfamily
Aspsulp1_71449_CE71448_16168	superfamily	223021	252	493	0.002062	41.0773	cl33720	PHA03247 superfamily
Aspnid1_5654_AN1052	superfamily	223021	221	463	2.02E-06	51.0925	cl33720	PHA03247 superfamily
Aspfove1_146492_fgenesh1_pm_1_155	superfamily	223021	221	463	2.84E-06	50.3221	cl33720	PHA03247 superfamily
Asppac1_77344_CE77343_13443	superfamily	223021	248	495	4.36E-05	46.8553	cl33720	PHA03247 superfamily
Aspcorr1_11061_e_gw1_11_72_1	superfamily	223021	216	463	2.06E-05	47.6257	cl33720	PHA03247 superfamily
Aspnav1_1_163417_CE163416_11985	superfamily	223021	251	537	5.34E-05	46.4701	cl33720	PHA03247 superfamily
Aspsy1_140011_e_gw1_1_554_1	superfamily	223021	266	544	1.7E-05	48.0109	cl33720	PHA03247 superfamily
Aspten1_731120_gm1_1925_g	superfamily	223021	270	504	6.77E-06	51.0925	cl33720	PHA03247 superfamily
Aspang1_948799_fgenesh1_pm_6_47	superfamily	223021	263	504	1.29E-05	49.9369	cl33720	PHA03247 superfamily
Aspaura1_203970_gm1_7400_g	superfamily	223021	261	487	1.44E-05	49.9369	cl33720	PHA03247 superfamily
Asppuu1_242407_MIX1103_477_57	superfamily	223021	263	504	2.39E-06	52.2481	cl33720	PHA03247 superfamily
Aspcreb1_569476_gm1_777_g	superfamily	223021	263	504	1.3E-06	53.4037	cl33720	PHA03247 superfamily
Aspaurlat1_305250_MIX1778_78_46	superfamily	223021	254	550	0.002274	42.6181	cl33720	PHA03247 superfamily
Aspvar1_101094_CE101093_716	superfamily	223021	250	490	1.89E-05	48.0109	cl33720	PHA03247 superfamily
Aspdef1_88007_MIX9481_28_69	superfamily	223021	38	417	3.48E-05	47.2405	cl33720	PHA03247 superfamily
Aspins1_294536_fgenesh1_pm_13_15	superfamily	223021	979	1215	0.000431	44.9293	cl33720	PHA03247 superfamily
Aspegy1_533726_fgenesh1_pm_73_2	superfamily	223021	980	1261	0.000455	44.9293	cl33720	PHA03247 superfamily
Aspspe1_1_182093_fgenesh1_pm_2_66	superfamily	223021	979	1142	0.000328	45.3145	cl33720	PHA03247 superfamily
Aspth1_360369_fgenesh1_pm_21_90	superfamily	223021	992	1229	3.8E-05	48.3961	cl33720	PHA03247 superfamily
Aspoch1_453585_estExt_Genewise1_C_2_t30304	superfamily	223021	253	506	0.001189	42.2329	cl33720	PHA03247 superfamily
Asphey1_11489_fgenesh1_pg_1_159	superfamily	223021	264	505	4.21E-06	51.4777	cl33720	PHA03247 superfamily
Aspramb1_134084_CE134083_4844	superfamily	223021	253	506	0.001189	42.2329	cl33720	PHA03247 superfamily
Aspram1_637_ARAM_003281T0	superfamily	223021	253	506	0.001189	42.2329	cl33720	PHA03247 superfamily
Aspoch1432_1_5577_AOCH_007688T0	superfamily	223021	253	506	0.001189	42.2329	cl33720	PHA03247 superfamily
Aspamy1_170918_fgenesh1_pm_20_14	superfamily	223021	988	1243	9.93E-06	50.3221	cl33720	PHA03247 superfamily
Aspcru1_386169_fgenesh1_pm_8_44	superfamily	223021	958	1106	0.000885	43.7737	cl33720	PHA03247 superfamily
Aspisr1_23698_gm1_2237_g	superfamily	223021	304	514	1.19E-06	53.4037	cl33720	PHA03247 superfamily
Asphet1_65675_CE65674_586	superfamily	223021	257	496	0.000143	44.9293	cl33720	PHA03247 superfamily
Asptan1_277045_CE277044_3113	superfamily	223021	274	493	0.000117	45.3145	cl33720	PHA03247 superfamily
Aspkar1_164961_CE164960_437	superfamily	223021	253	482	4.49E-08	56.4853	cl33720	PHA03247 superfamily
Aspneos1_31051_gm1_1922_g	superfamily	223021	257	480	1.8E-05	48.0109	cl33720	PHA03247 superfamily
Aspave1_126501_fgenesh1_pm_7_1	superfamily	223021	266	475	0.000848	43.7737	cl33720	PHA03247 superfamily
Asptaic1_44404_CE44403_2726	superfamily	223021	275	505	0.000342	43.7737	cl33720	PHA03247 superfamily
Aspca11822_1_403887_CE403886_12045	superfamily	223021	331	567	2.42E-08	57.6409	cl33720	PHA03247 superfamily
Aspca12550_1_279522_CE279521_11743	superfamily	223021	323	567	2.23E-09	61.1077	cl33720	PHA03247 superfamily
Aspca4_47456_CE47455_22447	superfamily	223021	331	567	2.42E-08	57.6409	cl33720	PHA03247 superfamily
Aspstel1_1_190175_fgenesh1_pm_32_25	superfamily	223021	266	447	5.41E-05	48.0109	cl33720	PHA03247 superfamily
Aspbr1_121725_e_gw1_4_363_1	superfamily	223021	272	482	0.000722	42.6181	cl33720	PHA03247 superfamily
Aspfo1_130898_e_gw1_4_1507_1	superfamily	223021	250	493	2.37E-05	47.6257	cl33720	PHA03247 superfamily

Aspflo1_185432_MIX4414_3_7	superfamily	223021	269	565	1.03E-05	50.3221	cl33720	PHA03247 superfamily
Aspaste1_151675_gm1_4656_g	superfamily	223021	250	494	4.44E-06	49.9369	cl33720	PHA03247 superfamily
Aspni7_1041677_CE41677_87	superfamily	223021	267	534	5.15E-05	46.4701	cl33720	PHA03247 superfamily
Aspni_DSM_1_161772_An08g05100m_01	superfamily	223021	251	518	7.25E-05	46.0849	cl33720	PHA03247 superfamily
Aspni_NRRL3_1_11142_NRRL3_11142	superfamily	223021	251	518	7.25E-05	46.0849	cl33720	PHA03247 superfamily
Aspni_bvT_1_371413_estExt_Genemark1_C_220097	superfamily	223021	265	532	3.9E-05	46.8553	cl33720	PHA03247 superfamily
Aspneo1_296797_e_gw1_1_991_1	superfamily	223021	259	480	9.16E-06	48.7813	cl33720	PHA03247 superfamily
Aspka1_1_18069_AKAW_06596	superfamily	223021	250	493	2.37E-05	47.6257	cl33720	PHA03247 superfamily
Aspcos1_39787_CE39786_674	superfamily	223021	250	515	4.96E-07	53.0185	cl33720	PHA03247 superfamily
Asptu1_186752_gm1_943_g	superfamily	223021	250	494	4.44E-06	49.9369	cl33720	PHA03247 superfamily
Aspvad1_458789_gm1_10907_g	superfamily	223021	250	515	6.35E-07	52.6333	cl33720	PHA03247 superfamily
Aspte1_439_ATET_00439	superfamily	223021	269	498	0.000567	44.5441	cl33720	PHA03247 superfamily
Aspsep1_1895_CE1894_4325	superfamily	223021	275	483	0.00048	43.3885	cl33720	PHA03247 superfamily
Asppip1_364706_e_gw1_3_133_1	superfamily	223021	250	493	2.37E-05	47.6257	cl33720	PHA03247 superfamily
Aspscl1_239338_CE239337_3233	superfamily	223021	257	504	4.95E-09	59.5669	cl33720	PHA03247 superfamily
Aspala1_143485_fgenesh1_pm_6_241	superfamily	223021	269	495	0.004452	41.4625	cl33720	PHA03247 superfamily
Aspeuc1_385688_fgenesh1_pg_4_290	superfamily	223021	250	515	2.44E-07	53.7889	cl33720	PHA03247 superfamily
Aspfili1_192200_fgenesh1_pm_27_31	superfamily	223021	963	1224	0.009192	40.6921	cl33720	PHA03247 superfamily
Aspcap1_131223_MIX9090_40_46	superfamily	223021	238	453	4.06E-08	58.4113	cl33720	PHA03247 superfamily
Aspoc2036_1_566126_fgenesh1_pg_1_1087	superfamily	223021	263	492	0.001374	41.8477	cl33720	PHA03247 superfamily
Aspwest1_39939_CE39938_1607	superfamily	223021	285	547	0.007337	39.5365	cl33720	PHA03247 superfamily
Aspoli1_1_216478_fgenesh1_pm_302_5	superfamily	223021	277	535	0.000442	44.9293	cl33720	PHA03247 superfamily
Aspses1_303851_gm1_4873_g	superfamily	223021	285	495	0.004519	40.3069	cl33720	PHA03247 superfamily
Aspspi1_203311_CE203310_1886	superfamily	223021	263	475	0.001405	41.8477	cl33720	PHA03247 superfamily
Aspscle1_60952_CE60951_3310	superfamily	223021	256	478	0.000144	44.9293	cl33720	PHA03247 superfamily
Aspoc7043_1_650028_e_gw1_2_2727_1	superfamily	223021	263	493	0.001317	41.8477	cl33720	PHA03247 superfamily
Asppet1_287634_gm1_4249_g	superfamily	223021	263	493	0.001398	41.8477	cl33720	PHA03247 superfamily
Asppseute1_423105_e_gw1_195_254_1	superfamily	223021	269	498	0.002294	42.6181	cl33720	PHA03247 superfamily
PenchWisc1_1_140054_PCH_Pc13g13200	superfamily	223021	295	454	0.001113	42.2329	cl33720	PHA03247 superfamily
Pench1_71076_e_gw1_2_1867_1	superfamily	223021	295	454	0.001113	42.2329	cl33720	PHA03247 superfamily
Aspamb1_196250_fgenesh1_pm_51_43	superfamily	223021	250	473	0.001553	43.0033	cl33720	PHA03247 superfamily
Aspfu_A1163_1_100335_CADAFUBT00001201m_01	superfamily	223021	349	476	0.004515	40.3069	cl33720	PHA03247 superfamily
Aspfu1_1114_Afu1g12490_mRNA	superfamily	223021	349	476	0.004515	40.3069	cl33720	PHA03247 superfamily
Aspall1_80143_CE80142_5066	superfamily	223021	254	486	0.000199	44.5441	cl33720	PHA03247 superfamily
Aspfumig1_31904_CE31903_927	superfamily	223021	350	485	0.001631	41.4625	cl33720	PHA03247 superfamily
Aspdur1_168944_CE168943_4122	superfamily	223021	256	483	0.000832	42.6181	cl33720	PHA03247 superfamily
Neofi1_2680_7000001156973789	superfamily	223021	270	476	0.000719	42.6181	cl33720	PHA03247 superfamily
Aspniv1_265238_fgenesh1_pm_6_105	superfamily	223021	273	505	1.29E-05	49.9369	cl33720	PHA03247 superfamily
Penja1_462785_gm1_1879_g	superfamily	223021	260	453	0.000394	43.3885	cl33720	PHA03247 superfamily
Aspibe1_361274_e_gw1_34_83_1	superfamily	223021	256	474	0.000278	44.1589	cl33720	PHA03247 superfamily
Aspfla1_245617_fgenesh1_pm_64_5	superfamily	223021	282	489	9.41E-05	47.2405	cl33720	PHA03247 superfamily
Pengri1_8759_PGRI_052490T0	superfamily	223021	247	478	0.008443	39.1513	cl33720	PHA03247 superfamily
Pencop1_8355_PENCOP_c008G00665T0	superfamily	223021	245	471	9.11E-06	48.7813	cl33720	PHA03247 superfamily
Aspaurful1_476126_fgenesh1_pm_17_6	superfamily	223021	263	470	0.000208	46.0849	cl33720	PHA03247 superfamily
Asplup1_212861_fgenesh1_pm_90_8	superfamily	223021	263	470	0.000208	46.0849	cl33720	PHA03247 superfamily
Aspcorea1_310722_CE310721_10467	superfamily	223021	263	475	0.001358	41.8477	cl33720	PHA03247 superfamily
Aspwak1_191096_CE191095_7131	superfamily	223021	270	484	0.003324	40.6921	cl33720	PHA03247 superfamily
Aspgra1_431791_gm1_5108_g	superfamily	223021	160	446	1.03E-06	51.8629	cl33720	PHA03247 superfamily
Penvul1_1379_PENVUL_c012G01962T0	superfamily	223021	251	471	0.000602	43.0033	cl33720	PHA03247 superfamily
Asppterr1_167696_fgenesh1_pm_27_23	superfamily	223021	250	479	0.001852	42.6181	cl33720	PHA03247 superfamily
Aspjap1_193436_CE193435_10940	superfamily	223021	265	499	2.55E-05	47.2405	cl33720	PHA03247 superfamily
Aspvio1_69035_CE69034_4875	superfamily	223021	265	519	3.06E-05	47.2405	cl33720	PHA03247 superfamily
Aspuva1_80164_CE80163_3826	superfamily	223021	263	460	0.000898	42.2329	cl33720	PHA03247 superfamily
Aspacu1_316081_e_gw1_9_447_1	superfamily	223021	263	460	3.24E-05	47.2405	cl33720	PHA03247 superfamily
Aspflor1_211278_gm1_9054_g	superfamily	223021	257	449	5.46E-05	48.0109	cl33720	PHA03247 superfamily
Aspfij1_226471_CE226470_9810	superfamily	223021	263	460	3.5E-05	46.8553	cl33720	PHA03247 superfamily
Aspoer1_40517_CE40516_11574	superfamily	223021	270	476	0.000337	43.7737	cl33720	PHA03247 superfamily
Asppsev1_278958_gm1_9054_g	superfamily	223021	257	476	0.001118	42.2329	cl33720	PHA03247 superfamily
Parbr1_531_PABG_00531m_01	superfamily	223021	260	495	2.57E-06	50.7073	cl33720	PHA03247 superfamily
Byssp1_477_PVAR5_3672	superfamily	223021	367	451	0.004763	39.9217	cl33720	PHA03247 superfamily
Penar1_7045_transcript_OGE56306	superfamily	223021	288	501	0.000148	44.9293	cl33720	PHA03247 superfamily
Asphom1_105274_CE105273_3596	superfamily	223021	265	479	8.49E-06	48.7813	cl33720	PHA03247 superfamily
Penla1_372272_fgenesh1_pg_4_254	superfamily	223021	243	443	0.001558	41.4625	cl33720	PHA03247 superfamily
TalaHC1_1_1_55053_e_gw1_42_36_1	superfamily	223021	257	479	0.000176	44.5441	cl33720	PHA03247 superfamily
Penoc1_8163_transcript_PCH04732	superfamily	223021	257	478	2.94E-06	50.3221	cl33720	PHA03247 superfamily
Talpro1_357920_fgenesh1_pg_5_131	superfamily	223021	259	457	3.16E-06	50.3221	cl33720	PHA03247 superfamily
Aspchev1_6298_CE6297_4290	superfamily	223021	252	473	0.000902	42.2329	cl33720	PHA03247 superfamily
Cocim1_4252_CIMG_06878T0	superfamily	223021	260	469	0.000174	44.5441	cl33720	PHA03247 superfamily
Cocpo1_1_5086_XM_003070039_1	superfamily	223021	260	468	5.98E-05	46.0849	cl33720	PHA03247 superfamily
Cocpos1_9541_transcript_EFW13885	superfamily	223021	260	468	5.98E-05	46.0849	cl33720	PHA03247 superfamily

Aspund1_1_278765_fgenesh1_pm_1_321	superfamily	223021	254	623	5.23E-06	51.4777	cl33720	PHA03247 superfamily
Aspund1_1_278765_fgenesh1_pm_1_321	superfamily	223021	1003	1232	0.000887	43.7737	cl33720	PHA03247 superfamily
Gymau1_166894_gm1_4540_g	superfamily	223021	252	553	0.000957	42.2329	cl33720	PHA03247 superfamily
Aspfre1_153762_fgenesh1_pm_76_17	superfamily	223021	258	593	6.05E-05	47.6257	cl33720	PHA03247 superfamily
Pezech1_525593_fgenesh1_pg_12_204	superfamily	223021	298	578	0.009257	39.5365	cl33720	PHA03247 superfamily
Zoprh1_728341_fgenesh1_pm_37_20	superfamily	223021	277	527	2.44E-05	47.6257	cl33720	PHA03247 superfamily
Delco1_476388_gm1_8841_g	superfamily	223021	262	510	0.00059	43.0033	cl33720	PHA03247 superfamily
Terbo2_781483_e_gw1_9_227_1	superfamily	223021	348	533	7.37E-05	46.0849	cl33720	PHA03247 superfamily
Pseel1_9633_g9265_t1	superfamily	223021	246	513	1.4E-05	48.3961	cl33720	PHA03247 superfamily
Tubcan1_941566_gm1_7117_g	superfamily	223021	386	664	0.008815	39.5365	cl33720	PHA03247 superfamily
Trepe1_264100_CE264099_38956	superfamily	223021	244	527	3.41E-06	50.3221	cl33720	PHA03247 superfamily
Artol1_8726_AOL_s00169g18m_01	superfamily	223021	241	535	2.28E-05	47.6257	cl33720	PHA03247 superfamily
Linin1_223675_e_gw1_14_456_1	superfamily	223021	248	535	2.97E-06	50.7073	cl33720	PHA03247 superfamily
Perma1_510121_e_gw1_1_175_1	superfamily	223021	238	514	2.06E-06	51.0925	cl33720	PHA03247 superfamily
Meltu1_674186_MIX21012_52815_93	superfamily	223021	286	516	2.6E-07	54.1741	cl33720	PHA03247 superfamily
Spafl1_891_NODE_11817_length_3265_cov_107_g7573_t1	superfamily	223021	316	451	0.000132	45.3145	cl33720	PHA03247 superfamily
Bissp1_688847_gm1_9344_g	superfamily	223021	285	470	0.000909	42.2329	cl33720	PHA03247 superfamily
Tryvi1_440647_e_gw1_6_229_1	superfamily	223021	406	508	0.003451	40.6921	cl33720	PHA03247 superfamily
Aplpr1_305098_fgenesh1_pg_3_210	superfamily	223021	246	565	0.00343	40.6921	cl33720	PHA03247 superfamily
Wesor1_239609_CE239608_7653	superfamily	223021	243	509	0.000546	43.3885	cl33720	PHA03247 superfamily
XylPMI703_1_50236_CE50235_11322	superfamily	223021	250	499	0.002118	41.0773	cl33720	PHA03247 superfamily
Didsa1_87328_CE87327_10519	superfamily	223021	244	529	9E-08	55.7149	cl33720	PHA03247 superfamily
Phisu1_13225_transcript_CZR53787	superfamily	223021	295	533	0.000471	43.3885	cl33720	PHA03247 superfamily
Plemel1_105900_CE105899_14980	superfamily	223021	401	573	0.006352	39.9217	cl33720	PHA03247 superfamily
Myrian1_113607_CE113606_24589	superfamily	223021	245	497	0.000145	44.9293	cl33720	PHA03247 superfamily
Macan1_426874_fgenesh1_pm_1_104	superfamily	223021	243	533	0.000658	43.0033	cl33720	PHA03247 superfamily
Podcur1_471017_estExt_fgenesh1_pm_C_2000008	superfamily	223021	280	480	7.81E-07	52.2481	cl33720	PHA03247 superfamily
Lopmy1_565459_gm1_11464_g	superfamily	223021	279	796	0.009531	40.6921	cl33720	PHA03247 superfamily
Bauco1_467987_CE306884_33891	superfamily	223021	246	556	0.002089	41.4625	cl33720	PHA03247 superfamily
Brefa1_321669_CE321668_14658	superfamily	223021	295	532	0.006303	39.9217	cl33720	PHA03247 superfamily
Torra1_29471_CE29470_6013	superfamily	223021	306	480	0.000107	44.9293	cl33720	PHA03247 superfamily
Acema1_744223_gm1_9971_g	superfamily	223021	295	550	4.45E-05	46.8553	cl33720	PHA03247 superfamily
Thian1_376562_e_gw1_5_559_1	superfamily	223021	273	470	0.000456	43.3885	cl33720	PHA03247 superfamily
Stely1_1772_TW65_01665T0	superfamily	223021	241	562	0.00364	40.3069	cl33720	PHA03247 superfamily
Clamic1_259668_CE259667_15531	superfamily	223021	267	400	0.000277	44.1589	cl33720	PHA03247 superfamily
Conli1_5219_NODE_2333_length_98482_cov_22_g3682_t1	superfamily	223021	281	569	3.03E-05	47.2405	cl33720	PHA03247 superfamily
Pyrly1_572587_gm1_8264_g	superfamily	223021	235	447	2.71E-05	47.2405	cl33720	PHA03247 superfamily
Fenfe1_103585_CE103584_36961	superfamily	223021	262	533	0.000395	43.7737	cl33720	PHA03247 superfamily
Coclu2_16930_fgenesh1_pg_3_118	superfamily	223021	253	541	0.001422	41.8477	cl33720	PHA03247 superfamily
Frisi1_753_B0A55_13085	superfamily	223021	251	533	0.00164	41.8477	cl33720	PHA03247 superfamily
Endpal1_8037_EPA1_010118_RA	superfamily	223021	337	544	1.62E-05	48.0109	cl33720	PHA03247 superfamily
EndpusZ1_700_EPUS_01261T0	superfamily	223021	344	505	4.66E-06	49.9369	cl33720	PHA03247 superfamily
PlePMI138_1_17761_CE17760_9229	superfamily	223021	367	505	0.000861	42.6181	cl33720	PHA03247 superfamily
Bombom1_28346_CE28345_31502	superfamily	223021	260	492	2.63E-05	47.2405	cl33720	PHA03247 superfamily
Podcom1_105258_CE105257_8637	superfamily	223021	272	520	0.000379	43.3885	cl33720	PHA03247 superfamily
Acrst1_1319294_fgenesh1_pg_3_519	superfamily	223021	285	468	0.00029	43.7737	cl33720	PHA03247 superfamily
Cercau1_539525_fgenesh1_pm_6_360	superfamily	223021	265	519	2.82E-06	50.3221	cl33720	PHA03247 superfamily
Thiap1_657363_fgenesh1_pm_10_52	superfamily	223021	293	520	0.000529	43.3885	cl33720	PHA03247 superfamily
Sarstr1_318237_gm1_7241_g	superfamily	223021	285	468	0.00029	43.7737	cl33720	PHA03247 superfamily
Thiar1_720655_e_gw1_49_42_1	superfamily	223021	311	558	0.000311	43.7737	cl33720	PHA03247 superfamily
Lasov1_531640_e_gw1_5_695_1	superfamily	223021	266	454	7.87E-05	45.6997	cl33720	PHA03247 superfamily
Bombar1_479584_fgenesh1_pg_2_1372	superfamily	223021	266	504	0.001747	41.4625	cl33720	PHA03247 superfamily
Podfim1_229309_CE229308_21346	superfamily	223021	271	532	1.8E-05	48.0109	cl33720	PHA03247 superfamily
Ternu1_200085_CE200084_22302	superfamily	223021	247	444	1.53E-07	54.9445	cl33720	PHA03247 superfamily
Melva1_584285_gm1_9590_g	superfamily	223021	245	503	0.008219	39.1513	cl33720	PHA03247 superfamily
Phisc1_676539_fgenesh1_pg_30_238	superfamily	223021	350	520	4.78E-05	46.4701	cl33720	PHA03247 superfamily
Blugr2_1228_BLGH_00654_mRNA_1	superfamily	223021	79	622	0.00194	41.4625	cl33720	PHA03247 superfamily
Ceraff1_204517_CE204516_13089	superfamily	223021	256	462	7.24E-05	45.6997	cl33720	PHA03247 superfamily
Frisi1_9218_B0A55_01134	superfamily	223021	251	538	1.87E-05	48.0109	cl33720	PHA03247 superfamily
Apope1_536162_fgenesh1_pg_6_822	superfamily	223021	268	459	0.000165	44.5441	cl33720	PHA03247 superfamily
Conioc1_1211268_gm1_1916_g	superfamily	223021	354	594	0.005076	39.9217	cl33720	PHA03247 superfamily
LecAK0013_1_445782_fgenesh1_pm_1_821	superfamily	223021	265	522	7.78E-07	52.2481	cl33720	PHA03247 superfamily
Corsim1_36236_e_gw1_8_179_1	superfamily	223021	291	507	0.006761	39.5365	cl33720	PHA03247 superfamily
Humgr1_8376_Humgr2_005231_RA	superfamily	223021	287	504	1.7E-06	51.0925	cl33720	PHA03247 superfamily
Rhier1_711537_gm1_15712_g	superfamily	223021	248	472	2.38E-06	50.7073	cl33720	PHA03247 superfamily
Lashi1_593299_fgenesh1_pm_7_192	superfamily	223021	263	487	2.32E-07	53.7889	cl33720	PHA03247 superfamily
Beaba1_5501_BBA_05501m_01	superfamily	223021	216	463	1.07E-07	54.9445	cl33720	PHA03247 superfamily
Corbr1_271_transcript_OAA52430	superfamily	223021	216	464	2.22E-06	50.7073	cl33720	PHA03247 superfamily
Trias1_27876_gm1_5845_g	superfamily	223021	217	465	2.76E-05	47.2405	cl33720	PHA03247 superfamily
Fusmo1_297981_MIX209_22048_88	superfamily	223021	318	460	3.65E-06	49.9369	cl33720	PHA03247 superfamily
Rutfi1_709150_estExt_Genemark1_C_5_t10354	superfamily	223021	270	531	0.004025	40.3069	cl33720	PHA03247 superfamily
Trikon1_147686_CE147685_25566	superfamily	223021	250	475	0.000169	44.5441	cl33720	PHA03247 superfamily
Tritai1_35748_CE35747_33467	superfamily	223021	250	471	1.6E-06	51.0925	cl33720	PHA03247 superfamily
Triei1_375552_gm1_7643_g	superfamily	223021	217	467	0.00076	42.6181	cl33720	PHA03247 superfamily
Triev1_193703_CE193702_27587	superfamily	223021	217	466	0.000127	44.9293	cl33720	PHA03247 superfamily

Trigam1_7763_TGAM01_07763T0	superfamily	223021	325	470	0.000583	43.0033	cl33720	PHA03247 superfamily
Apiver1_32826_CE32825_3250	superfamily	223021	266	547	7.47E-05	45.6997	cl33720	PHA03247 superfamily
Triatr1_496815_gm1_8124_g	superfamily	223021	251	469	0.000107	45.3145	cl33720	PHA03247 superfamily
Triatro1_441594_fgenesh1_pg_10_356	superfamily	223021	251	469	0.000109	45.3145	cl33720	PHA03247 superfamily
Triatrov1_477853_MIX5214_25609_90	superfamily	223021	251	469	0.000107	45.3145	cl33720	PHA03247 superfamily
Colny1_1022682_mrna_14402	superfamily	223021	255	458	0.002023	41.0773	cl33720	PHA03247 superfamily
Myrin1_79802_CE79801_61490	superfamily	223021	216	467	3.45E-08	56.4853	cl33720	PHA03247 superfamily
Triat2_42972_Triat1_e_gw1_7_841_1	superfamily	223021	251	469	0.000121	44.9293	cl33720	PHA03247 superfamily
Fusma1_4031_transcript_CVL03167	superfamily	223021	315	472	0.000304	43.7737	cl33720	PHA03247 superfamily
Ilysp1_1084539_CE1084538_21146	superfamily	223021	311	452	4.63E-07	53.0185	cl33720	PHA03247 superfamily
Ilyeu1_674004_gm1_9830_g	superfamily	223021	311	452	4.63E-07	53.0185	cl33720	PHA03247 superfamily
Fuspro1_1677_transcript_CZR35398	superfamily	223021	315	472	0.000299	43.7737	cl33720	PHA03247 superfamily
Ilyro1_145191_CE145190_3148	superfamily	223021	213	528	4E-08	56.8705	cl33720	PHA03247 superfamily
Cloros1_11678_CRV2T00007275_1	superfamily	223021	213	528	4.17E-08	56.4853	cl33720	PHA03247 superfamily
Tolpa1_4862_transcript_POR31872	superfamily	223021	443	584	9.31E-05	45.6997	cl33720	PHA03247 superfamily
FoxF207_1_9830_CE9829_20519	superfamily	223021	317	474	0.001078	41.8477	cl33720	PHA03247 superfamily
FusoxFo47_680_rna_gnl_WGS_AFMM_mrna_FOZG_00553T0	superfamily	223021	317	474	0.001078	41.8477	cl33720	PHA03247 superfamily
FusoxFo47_2_7966_CE7965_11391	superfamily	223021	317	474	0.001078	41.8477	cl33720	PHA03247 superfamily
Fusoxys1_817586_gm1_467_g	superfamily	223021	315	472	0.001305	41.8477	cl33720	PHA03247 superfamily
FoxMRL8996_17519_CE17518_37530	superfamily	223021	317	474	0.001075	41.8477	cl33720	PHA03247 superfamily
Fusox32931_5166_rna_gnl_WGS_AFML_mrna_FOYG_16257T0	superfamily	223021	317	474	0.001075	41.8477	cl33720	PHA03247 superfamily
Fusoxalb1_841828_gm1_2377_g	superfamily	223021	317	474	0.001078	41.8477	cl33720	PHA03247 superfamily
Fusoxcon1_18427_rna_gnl_WGS_AGNF_mrna_FOPG_01271T1	superfamily	223021	317	474	0.001144	41.8477	cl33720	PHA03247 superfamily
Fusoxcub1_507_rna_gnl_WGS_AGND_mrna_FOIG_00370T0	superfamily	223021	316	473	0.001085	41.8477	cl33720	PHA03247 superfamily
Fusox2_7216_FOXG_11273T0	superfamily	223021	317	474	0.001075	41.8477	cl33720	PHA03247 superfamily
Fusoxlyc1_22724_rna_gnl_WGS_AGBH_mrna_FOWG_08668T0	superfamily	223021	317	474	0.001078	41.8477	cl33720	PHA03247 superfamily
FoxPHW726_1_56310_CE56309_30812	superfamily	223021	317	474	0.001078	41.8477	cl33720	PHA03247 superfamily
Fusoxmel1_681_rna_gnl_WGS_AGNE_mrna_FOMG_00548T1	superfamily	223021	317	474	0.001075	41.8477	cl33720	PHA03247 superfamily
FoxF1003_1_10862_CE10861_19680	superfamily	223021	317	474	0.001078	41.8477	cl33720	PHA03247 superfamily
FoxF1004_1_857176_fgenesh1_pg_3_484	superfamily	223021	317	474	0.001078	41.8477	cl33720	PHA03247 superfamily
FoxF1008_1_672978_e_gw1_1_4245_1	superfamily	223021	317	474	0.001078	41.8477	cl33720	PHA03247 superfamily
FoxF201_1_10463_CE10462_37755	superfamily	223021	317	474	0.001078	41.8477	cl33720	PHA03247 superfamily
FoxF202_1_202498_CE202497_25562	superfamily	223021	317	474	0.001078	41.8477	cl33720	PHA03247 superfamily
FoxF204_1_22202_CE22201_9918	superfamily	223021	317	474	0.001078	41.8477	cl33720	PHA03247 superfamily
FoxF206_1_91647_CE91646_17913	superfamily	223021	317	474	0.001078	41.8477	cl33720	PHA03247 superfamily
Fusco1_568337_fgenesh1_pg_1_1549	superfamily	223021	306	473	0.000252	44.1589	cl33720	PHA03247 superfamily
Lepor2_291340_CE291339_6287	superfamily	223021	279	507	0.001331	41.8477	cl33720	PHA03247 superfamily
Leptod1_107922_CE107921_2780	superfamily	223021	279	507	0.001308	41.8477	cl33720	PHA03247 superfamily
Fusoxpis1_2823_rna_gnl_WGS_AGBI_mrna_FOVG_01708T0	superfamily	223021	317	474	0.001144	41.8477	cl33720	PHA03247 superfamily
Fusoxrap1_22710_rna_gnl_WGS_AGNG_mrna_FOQG_05045T1	superfamily	223021	317	474	0.001144	41.8477	cl33720	PHA03247 superfamily
FusoxFo47_679_rna_gnl_WGS_AFMM_mrna_FOZG_00553T1	superfamily	223021	317	474	0.001078	41.8477	cl33720	PHA03247 superfamily
Fusox32931_5164_rna_gnl_WGS_AFML_mrna_FOYG_16257T1	superfamily	223021	317	474	0.001075	41.8477	cl33720	PHA03247 superfamily
Fusoxcon1_18425_rna_gnl_WGS_AGNF_mrna_FOPG_01271T0	superfamily	223021	317	474	0.001144	41.8477	cl33720	PHA03247 superfamily
Fusoxcub1_506_rna_gnl_WGS_AGND_mrna_FOIG_00370T1	superfamily	223021	316	473	0.001085	41.8477	cl33720	PHA03247 superfamily
Fusox2_7214_FOXG_11273T1	superfamily	223021	317	474	0.001075	41.8477	cl33720	PHA03247 superfamily
Fusoxmel1_680_rna_gnl_WGS_AGNE_mrna_FOMG_00548T0	superfamily	223021	317	474	0.001075	41.8477	cl33720	PHA03247 superfamily
FopisF105_1_363183_fgenesh1_pm_22_16	superfamily	223021	317	474	0.001144	41.8477	cl33720	PHA03247 superfamily
Fusoxpis1_2825_rna_gnl_WGS_AGBI_mrna_FOVG_01708T1	superfamily	223021	317	474	0.001144	41.8477	cl33720	PHA03247 superfamily
Fusoxrad1_4480_rna_gnl_WGS_AGNB_mrna_FOCG_12388T0	superfamily	223021	317	474	0.001078	41.8477	cl33720	PHA03247 superfamily
Fusoxrap1_22711_rna_gnl_WGS_AGNG_mrna_FOQG_05045T0	superfamily	223021	317	474	0.001144	41.8477	cl33720	PHA03247 superfamily
FoxII5_1014246_CE14246_12356	superfamily	223021	316	473	0.001085	41.8477	cl33720	PHA03247 superfamily
Trimi1_149191_CE149190_14445	superfamily	223021	250	472	2.91E-06	50.3221	cl33720	PHA03247 superfamily
Trieur1_274747_gm1_4621_g	superfamily	223021	250	474	9.49E-08	55.3297	cl33720	PHA03247 superfamily
Ilyrob1_530997_gm1_6101_g	superfamily	223021	311	452	5.93E-07	52.6333	cl33720	PHA03247 superfamily
Neora1_583931_e_gw1_13_553_1	superfamily	223021	312	452	0.000201	44.1589	cl33720	PHA03247 superfamily
Fusfu1_442_FFUJ_01649m_01	superfamily	223021	315	472	0.000296	43.7737	cl33720	PHA03247 superfamily
Fuspr1_5014_transcript_CVL04312	superfamily	223021	315	472	0.000396	43.3885	cl33720	PHA03247 superfamily
Nieex1_820865_gm1_1041_g	superfamily	223021	234	469	3.8E-08	56.4853	cl33720	PHA03247 superfamily
Ophsi1_208_rna_G6O67_000189	superfamily	223021	347	489	0.000139	44.9293	cl33720	PHA03247 superfamily
TrireMAT11_1_4374_TRC1_004047_T1	superfamily	223021	267	505	0.000119	45.3145	cl33720	PHA03247 superfamily
TrireMAT12_1_4546_TRC2_004187_T1	superfamily	223021	267	505	0.000119	45.3145	cl33720	PHA03247 superfamily
Trire2_122284_estExt_fgenesh5_pg_C_110199	superfamily	223021	267	511	1.57E-05	48.0109	cl33720	PHA03247 superfamily

Trilo3_1340338_e_gw1_14_551_1	superfamily	223021	322	499	8.03E-05	45.6997	cl33720	PHA03247 superfamily
Trior1_80298_CE80297_37724	superfamily	223021	322	508	0.00122	41.8477	cl33720	PHA03247 superfamily
Trilon1_201835_CE201834_35868	superfamily	223021	322	508	0.00122	41.8477	cl33720	PHA03247 superfamily
Tripar1_5949_rna8778	superfamily	223021	216	486	0.000948	42.2329	cl33720	PHA03247 superfamily
Trica1_389511_gm1_930_g	superfamily	223021	327	477	2.23E-06	50.7073	cl33720	PHA03247 superfamily
Tricr1_279114_MIX3423_15528_26	superfamily	223021	250	460	6.55E-09	58.7965	cl33720	PHA03247 superfamily
Fustri1_619034_gm1_12299_g	superfamily	223021	312	478	0.000294	43.7737	cl33720	PHA03247 superfamily
Durrog2_93144_CE93143_18538	superfamily	223021	265	455	5.1E-05	46.0849	cl33720	PHA03247 superfamily
Cormi1_4520_CCM_04531m_01	superfamily	223021	607	796	1.62E-06	52.2481	cl33720	PHA03247 superfamily
Fusoxvas1_24674_rna_gnl_WGS_AGNC_mrna_FOTG_05490T1	superfamily	223021	317	474	0.001069	41.8477	cl33720	PHA03247 superfamily
Fusoxvas1_24675_rna_gnl_WGS_AGNC_mrna_FOTG_05490T0	superfamily	223021	317	474	0.001069	41.8477	cl33720	PHA03247 superfamily
Trist1_319629_gm1_2381_g	superfamily	223021	216	482	0.002169	41.0773	cl33720	PHA03247 superfamily
FoxF1010_1_19694_CE19693_3882	superfamily	223021	317	474	0.001069	41.8477	cl33720	PHA03247 superfamily
Fusve2_4999_FVEG_09521T0	superfamily	223021	316	473	0.000533	43.0033	cl33720	PHA03247 superfamily
Triveli1_95866_CE95865_14382	superfamily	223021	243	462	0.000171	44.5441	cl33720	PHA03247 superfamily
Triros1_971536_gm4_8713_g	superfamily	223021	245	472	7.61E-09	58.7965	cl33720	PHA03247 superfamily
Trsp14807_92247_CE92246_9178	superfamily	223021	250	483	6.3E-05	46.0849	cl33720	PHA03247 superfamily
TriTUC5505_1_383686_fgenesh1_pm_29_3	superfamily	223021	250	472	0.000353	43.7737	cl33720	PHA03247 superfamily
Trispi1_2_22129_gm1_9947_g	superfamily	223021	216	490	4.87E-11	65.73	cl33720	PHA03247 superfamily
Triiv1_84136_CE84135_16229	superfamily	223021	245	472	2.02E-06	51.0925	cl33720	PHA03247 superfamily
Tripare1_183132_CE183131_17030	superfamily	223021	316	469	0.000231	44.1589	cl33720	PHA03247 superfamily
TriharM10_1_111581_CE111580_25685	superfamily	223021	326	482	0.000272	44.1589	cl33720	PHA03247 superfamily
Tricae1_276620_fgenesh1_pm_20_25	superfamily	223021	238	423	0.001722	41.4625	cl33720	PHA03247 superfamily
Tricam1_79553_CE79552_12088	superfamily	223021	250	483	6.3E-05	46.0849	cl33720	PHA03247 superfamily
Trice1_97673_CE97672_20410	superfamily	223021	247	476	0.001433	41.8477	cl33720	PHA03247 superfamily
Trichl1_54856_CE54855_19526	superfamily	223021	232	486	1.35E-07	54.9445	cl33720	PHA03247 superfamily
Tricre1_29731_CE29730_19030	superfamily	223021	258	500	3.84E-07	53.4037	cl33720	PHA03247 superfamily
FoxFo5176_237899_e_gw1_1_3805_1	superfamily	223021	317	474	0.001116	41.8477	cl33720	PHA03247 superfamily
Fusre1_533157_fgenesh1_pg_1_1533	superfamily	223021	316	473	0.000459	43.3885	cl33720	PHA03247 superfamily
Marbr1_2001_MBM_05547	superfamily	223021	753	878	0.006926	41.8477	cl33720	PHA03247 superfamily
Ophau1_7668_transcript_PHH61977	superfamily	223021	281	443	0.000942	42.2329	cl33720	PHA03247 superfamily
Corco1_4062_transcript_OAA79537	superfamily	223021	247	459	8.95E-07	51.8629	cl33720	PHA03247 superfamily
Annbov1_15394_CE15393_55186	superfamily	223021	246	441	6.71E-05	45.6997	cl33720	PHA03247 superfamily
Tolop1_2918_transcript_KND94420	superfamily	223021	324	464	3.12E-05	46.8553	cl33720	PHA03247 superfamily
DalEC12_1_15846_fgenesh1_pg_38_93	superfamily	223021	264	501	3.08E-05	46.8553	cl33720	PHA03247 superfamily
Dales1_231116_CE231115_24740	superfamily	223021	264	501	3.19E-05	46.8553	cl33720	PHA03247 superfamily
Trivel1_116405_CE116404_27813	superfamily	223021	244	459	9.75E-05	45.3145	cl33720	PHA03247 superfamily
Daldec1_76352_CE76351_11485	superfamily	223021	267	506	3.26E-05	46.8553	cl33720	PHA03247 superfamily
DallocAZ0526_2_491174_fgenesh1_pm_4_45	superfamily	223021	264	503	6.57E-05	45.6997	cl33720	PHA03247 superfamily
Dalloc1_7370_CE7369_27415	superfamily	223021	267	506	6.19E-05	46.0849	cl33720	PHA03247 superfamily
Dallocu1_385168_gm1_3136_g	superfamily	223021	246	503	0.000135	44.9293	cl33720	PHA03247 superfamily
Dalver1_255059_fgenesh1_pm_52_7	superfamily	223021	264	503	1.99E-05	47.6257	cl33720	PHA03247 superfamily
AnFL0455_493303_gm1_4931_g	superfamily	223021	253	428	1.13E-05	48.3961	cl33720	PHA03247 superfamily
Anntru1_459783_gm1_4831_g	superfamily	223021	253	428	2.74E-05	47.2405	cl33720	PHA03247 superfamily
Annmae1_45012_CE45011_28794	superfamily	223021	253	428	0.007951	39.1513	cl33720	PHA03247 superfamily
Roster1_491119_gm1_3272_g	superfamily	223021	253	428	5.56E-05	46.0849	cl33720	PHA03247 superfamily
Fox26365_1_219679_e_gw1_102_202_1	superfamily	223021	317	474	0.001194	41.8477	cl33720	PHA03247 superfamily
Dalba1_206614_CE206613_121492	superfamily	223021	264	503	4.96E-05	46.4701	cl33720	PHA03247 superfamily
Dalcal1_455708_gm1_1768_g	superfamily	223021	246	502	0.001204	41.8477	cl33720	PHA03247 superfamily
DaFL1419_1_263004_CE263003_20389	superfamily	223021	243	481	6.72E-10	61.8781	cl33720	PHA03247 superfamily
Hypvin1_420688_gm1_7240_g	superfamily	223021	242	463	1.64E-05	47.6257	cl33720	PHA03247 superfamily
Hypcer1_28304_CE28303_27475	superfamily	223021	294	426	0.002579	40.6921	cl33720	PHA03247 superfamily
Trifer1_124037_CE124036_3129	superfamily	223021	250	490	4.68E-08	56.1001	cl33720	PHA03247 superfamily
HypEC38_3_218143_CE218142_6775	superfamily	223021	264	465	0.007316	39.1513	cl33720	PHA03247 superfamily
Hyptru1_257438_CE257437_19076	superfamily	223021	262	465	1.72E-05	47.6257	cl33720	PHA03247 superfamily
Hypmon1_151734_CE151733_33905	superfamily	223021	241	431	0.000865	42.2329	cl33720	PHA03247 superfamily
HypCI4A_1_11670_gm1_5097_g	superfamily	223021	241	444	9.65E-06	48.3961	cl33720	PHA03247 superfamily
HyNC0597_1_665168_gm1_6352_g	superfamily	223021	253	453	0.004135	39.9217	cl33720	PHA03247 superfamily
Torhe1_7864_VHEMI07320_1	superfamily	223021	222	474	0.005621	39.5365	cl33720	PHA03247 superfamily
Biscog1_582007_fgenesh1_pm_5_97	superfamily	223021	263	474	0.000333	43.3885	cl33720	PHA03247 superfamily
Stachl1_1953_transcript_KFA68696	superfamily	223021	241	477	4.69E-05	46.4701	cl33720	PHA03247 superfamily
Stach1_3582_transcript_KFA78415	superfamily	223021	214	476	0.000305	43.7737	cl33720	PHA03247 superfamily
Tri5649_1_410667_MIX764_13999_28	superfamily	223021	234	455	1.64E-06	51.0925	cl33720	PHA03247 superfamily
Tricec1_93302_CE93301_46071	superfamily	223021	249	470	0.004624	39.9217	cl33720	PHA03247 superfamily
HyNC1633_2_645930_MIX145_47814_43	superfamily	223021	262	460	0.000253	44.1589	cl33720	PHA03247 superfamily
Bisma1_8065_CE8064_29551	superfamily	223021	280	442	1.18E-06	51.4777	cl33720	PHA03247 superfamily
Stael1_407963_fgenesh1_pg_5_308	superfamily	223021	242	475	1.07E-05	48.3961	cl33720	PHA03247 superfamily
Bisme1_497477_CE497476_71981	superfamily	223021	263	426	0.001645	41.4625	cl33720	PHA03247 superfamily
Bismed1_554000_CE553999_75360	superfamily	223021	263	426	0.000342	43.3885	cl33720	PHA03247 superfamily
Bisnum1_629819_gm1_117_g	superfamily	223021	264	475	7.04E-06	49.1665	cl33720	PHA03247 superfamily
Aspac1_56272_fgenesh1_pg_1_499	superfamily	223021	223	418	0.000105	45.3145	cl33720	PHA03247 superfamily
XylFL1019_157986_CE157985_13922	superfamily	223021	249	470	1.75E-07	54.1741	cl33720	PHA03247 superfamily
Hypsub2_39212_CE39211_136770	superfamily	223021	232	423	0.000489	43.0033	cl33720	PHA03247 superfamily
Plecuc1_325164_gm1_2614_g	superfamily	223021	236	514	0.000933	42.2329	cl33720	PHA03247 superfamily

XyFL1777_1_789937_MIX310_22736_36	superfamily	223021	320	505	0.000113	45.3145	cl33720	PHA03247 superfamily
Nemani1_447901_gm1_2068_g	superfamily	223021	255	430	0.002327	40.6921	cl33720	PHA03247 superfamily
Exosi1_113065_PV11_01710T0	superfamily	223021	286	537	0.000724	42.6181	cl33720	PHA03247 superfamily
Xylint1_212825_CE212824_24453	superfamily	223021	302	513	0.002131	41.0773	cl33720	PHA03247 superfamily
NsAZ0576_1_331099_fgenesh1_pm_9_65	superfamily	223021	261	403	0.000644	42.6181	cl33720	PHA03247 superfamily
Aspwel1_165777_gm1_1026_g	superfamily	223021	204	437	0.000292	43.7737	cl33720	PHA03247 superfamily
Achstr1_486122_fgenesh1_pm_5_452	superfamily	223021	226	463	1.45E-08	57.6409	cl33720	PHA03247 superfamily
Necsp1_470146_estExt_fgenesh1_pg_C_130260	superfamily	223021	300	432	8.58E-06	48.7813	cl33720	PHA03247 superfamily
Melsp1_168542_fgenesh1_pg_2_849	superfamily	223021	272	513	3.95E-05	46.8553	cl33720	PHA03247 superfamily
Asptri1_201493_fgenesh1_pm_666_1	superfamily	223021	232	429	2.65E-05	48.7813	cl33720	PHA03247 superfamily
Cytch1_183573_CE183572_13320	superfamily	223021	298	529	5.69E-06	49.5517	cl33720	PHA03247 superfamily
Diahe1_13017_transcript_POS72540	superfamily	223021	288	497	2.03E-05	47.6257	cl33720	PHA03247 superfamily
DiaPMI573_1_600869_fgenesh1_pm_6_368	superfamily	223021	260	483	8.02E-07	52.2481	cl33720	PHA03247 superfamily
Neodi1_11955_AK830_g6177T0	superfamily	223021	251	455	3.19E-06	50.3221	cl33720	PHA03247 superfamily
Phiatt1_6889_AB675_2981T0	superfamily	223021	125	351	0.009293	38.3809	cl33720	PHA03247 superfamily
Phach1_4759_rna4758	superfamily	223021	305	458	0.004414	39.9217	cl33720	PHA03247 superfamily
Colch1_2705_mrna_6672	superfamily	223021	270	476	1.01E-09	61.4929	cl33720	PHA03247 superfamily
Diaam1_9062_rna9092	superfamily	223021	204	433	0.001677	41.0773	cl33720	PHA03247 superfamily
Fusoxpis1_2824_rna_gnl_WGS_AGBI_mrna_FOVG_01708T2	superfamily	223021	194	351	0.000599	42.2329	cl33720	PHA03247 superfamily
Fusoxrap1_22712_rna_gnl_WGS_AGNG_mrna_FOQG_05045T2	superfamily	223021	194	351	0.000599	42.2329	cl33720	PHA03247 superfamily
Fusoxvas1_24673_rna_gnl_WGS_AGNC_mrna_FOTG_05490T2	superfamily	223021	194	351	0.000599	42.2329	cl33720	PHA03247 superfamily
Fusox32931_5165_rna_gnl_WGS_AFML_mrna_FOYG_16257T2	superfamily	223021	194	351	0.000581	42.2329	cl33720	PHA03247 superfamily
Fusoxcon1_18426_rna_gnl_WGS_AGNF_mrna_FOPG_01271T2	superfamily	223021	194	351	0.000599	42.2329	cl33720	PHA03247 superfamily
Fusoxcub1_505_rna_gnl_WGS_AGND_mrna_FOIG_00370T2	superfamily	223021	193	350	0.000538	42.6181	cl33720	PHA03247 superfamily
Fusox2_7215_FOXG_11273T2	superfamily	223021	194	351	0.000581	42.2329	cl33720	PHA03247 superfamily
Fusoxmel1_682_rna_gnl_WGS_AGNE_mrna_FOMG_00548T2	superfamily	223021	194	351	0.000581	42.2329	cl33720	PHA03247 superfamily
FusoxFo47_678_rna_gnl_WGS_AFMM_mrna_FOZG_00553T2	superfamily	223021	194	351	0.000569	42.2329	cl33720	PHA03247 superfamily
Aspspe1_1_182093_fgenesh1_pm_2_66	superfamily	165527	280	416	0.001282	42.7932	cl29788	PHA03269 superfamily
Asphey1_11489_fgenesh1_pg_1_159	superfamily	165527	1041	1166	1.26E-05	49.3416	cl29788	PHA03269 superfamily
Asphor1_149261_fgenesh1_pm_8_33	superfamily	165527	273	424	0.00015	45.8748	cl29788	PHA03269 superfamily
Crymi1_11110_B0A49_09347	superfamily	165527	413	538	0.008259	38.9412	cl29788	PHA03269 superfamily
Fusoxy1_597928_fgenesh1_pm_15_59	superfamily	165527	352	425	0.008209	38.556	cl29788	PHA03269 superfamily
FopisF109_1_845875_fgenesh1_pm_5_299	superfamily	165527	327	443	0.000332	43.1784	cl29788	PHA03269 superfamily
FopisF23_1_365624_MIX1515_347_26	superfamily	165527	327	443	0.000332	43.1784	cl29788	PHA03269 superfamily
FopisF79_1_630649_fgenesh1_pm_33_63	superfamily	165527	327	443	0.000332	43.1784	cl29788	PHA03269 superfamily
FusoxT415_591516_gm1_1648_g	superfamily	165527	327	443	0.000332	43.1784	cl29788	PHA03269 superfamily
Fobas23_1_530936_fgenesh1_pm_1_351	superfamily	165527	327	443	0.000332	43.1784	cl29788	PHA03269 superfamily
Foxbas1_466001_fgenesh1_pm_18_178	superfamily	165527	327	443	0.000332	43.1784	cl29788	PHA03269 superfamily
Cryan3_116812_CE116811_9156	superfamily	165527	424	542	0.000128	44.7192	cl29788	PHA03269 superfamily
Verdah1_161901_CE161900_8651	superfamily	165527	301	391	0.006776	39.3264	cl29788	PHA03269 superfamily
Verda1_2281_VDAG_06763T0	superfamily	165527	301	391	0.006776	39.3264	cl29788	PHA03269 superfamily
Verdah1_131402_CE131401_8651	superfamily	165527	301	391	0.006776	39.3264	cl29788	PHA03269 superfamily
Verlo1_3793_transcript_CRK14601	superfamily	165527	301	391	0.006735	39.3264	cl29788	PHA03269 superfamily
Cryan3_349102_CE349101_9152	superfamily	165527	423	541	0.000133	44.7192	cl29788	PHA03269 superfamily
Asphai1_405932_fgenesh1_pm_168_3	superfamily	223039	359	689	5.14E-06	50.9401	cl33723	PHA03307 superfamily
Aspcap1_131223_MIX9090_40_46	superfamily	223039	265	726	0.00389	41.6953	cl33723	PHA03307 superfamily
Aspfla1_245617_fgenesh1_pm_64_5	superfamily	223039	348	730	0.001121	43.6213	cl33723	PHA03307 superfamily
Aspaurful1_476126_fgenesh1_pm_17_6	superfamily	223039	350	774	0.007192	40.9249	cl33723	PHA03307 superfamily
Asplup1_212861_fgenesh1_pm_90_8	superfamily	223039	350	774	0.007192	40.9249	cl33723	PHA03307 superfamily
Colph1_458935_fgenesh1_pm_5_125	superfamily	223039	228	497	0.000798	42.4657	cl33723	PHA03307 superfamily
Dreco1_2321_transcript_KYK61174	superfamily	223039	229	449	3.8E-05	46.7029	cl33723	PHA03307 superfamily
Chame1_432602_MIX12905_26049_89	superfamily	223061	259	384	0.00481	39.5973	cl25753	PHA03369 superfamily
Cylol1_87008_CE87007_18919	superfamily	223061	313	419	0.004419	39.5973	cl25753	PHA03369 superfamily
Plecu1_139916_CE139915_7477	superfamily	223061	315	445	0.000376	43.0641	cl25753	PHA03369 superfamily
Lopnit1_1_412517_CE412516_5305	superfamily	177614	315	503	0.001771	41.1916	cl31823	PHA03377 superfamily
Altlo1_257016_ALGCTG_2604_jigsaw_mRNA_59	superfamily	177614	233	508	0.001831	41.1916	cl31823	PHA03377 superfamily
Apibac1_412807_gm1_6953_g	superfamily	177614	271	499	0.00119	41.962	cl31823	PHA03377 superfamily
Colorb1_138431_mrna_4951	superfamily	177614	232	527	0.000747	42.3472	cl31823	PHA03377 superfamily
Lompr1_5587_transcript_PKS06982	superfamily	223065	322	485	0.006383	39.28	cl33729	PHA03378 superfamily
Delst1_367146_fgenesh1_pg_3_413	superfamily	223065	295	484	0.001305	41.5912	cl33729	PHA03378 superfamily
Metro1_1712_MAA_01811T0	superfamily	223066	219	496	3.63E-05	46.5901	cl33730	PHA03379 superfamily
Metbr1_4315_XM_014691346_1	superfamily	223066	219	496	0.000159	44.6641	cl33730	PHA03379 superfamily
Metani1_7547_transcript_KID67184	superfamily	223066	219	495	0.001076	41.9677	cl33730	PHA03379 superfamily
Aspstel1_1_190175_fgenesh1_pm_32_25	superfamily	215533	287	361	3.6E-05	47.1413	cl33616	PLN02983 superfamily

Aspcali1_664842_fgenesh1_pm_125_27	superfamily	215641	843	1084	0.002866	41.7715	cl33668	PLN03237 superfamily
Aspchris1_498928_fgenesh1_pm_5_27	superfamily	235175	819	901	0.001054	43.1288	cl35229	PRK03918 superfamily
Aspaurlat1_305250_MIX1778_78_46	superfamily	235540	309	343	0.000595	41.7716	cl35354	PRK05641 superfamily
Spoin1_38_transcript_OAA67843	superfamily	235540	421	454	0.006661	37.5344	cl35354	PRK05641 superfamily
Trihel1_251742_CE251741_17211	superfamily	235826	322	359	0.007592	36.7136	cl35488	PRK06549 superfamily
Tricec1_93302_CE93301_46071	superfamily	235826	321	360	0.004099	37.484	cl35488	PRK06549 superfamily
Asppal1_1_333678_fgenesh1_pm_17_76	superfamily	235906	237	457	4.59E-06	49.4628	cl35530	PRK07003 superfamily
AurpulNBB1_81351_gm1_4483_g	superfamily	236090	245	399	0.003859	40.3545	cl35613	PRK07764 superfamily
Triaspe1_201723_CE201722_14091	superfamily	236090	251	433	4.18E-05	46.5177	cl35613	PRK07764 superfamily
Triasper1_102949_CE102948_4880	superfamily	236090	251	433	6.65E-05	45.7473	cl35613	PRK07764 superfamily
Triasp1_467483_gm1_6887_g	superfamily	236090	251	433	6.65E-05	45.7473	cl35613	PRK07764 superfamily
Tricom1_312750_fgenesh1_pm_18_11	superfamily	236090	251	421	0.002172	40.7397	cl35613	PRK07764 superfamily
Triag1_88576_CE88575_2814	superfamily	236090	251	422	0.005031	39.5841	cl35613	PRK07764 superfamily
Aspcl1_7285_7000001156880341	superfamily	236138	351	458	0.008774	39.0786	cl35634	PRK07994 superfamily
Totfu1_176308_CE176307_22727	superfamily	236138	394	507	0.000122	44.8566	cl35634	PRK07994 superfamily
Rhili1_41735_e_gw1_8_643_1	superfamily	236138	409	484	0.001376	41.775	cl35634	PRK07994 superfamily
Pennord1_6088_rna6087	superfamily	236333	384	539	0.007742	38.9214	cl35739	PRK08691 superfamily
Asphethal1_273971_fgenesh1_pm_4_107	superfamily	236669	290	454	0.007887	40.8387	cl35903	PRK10263 superfamily
Aspcam1_314862_gm1_1037_g	superfamily	236669	293	493	0.0045	40.0683	cl35903	PRK10263 superfamily
Aspell1_435257_gm1_10825_g	superfamily	236669	353	491	4.98E-05	46.6167	cl35903	PRK10263 superfamily
Aspbrev1_84337_CE84336_11112	superfamily	236669	255	487	0.001396	41.6091	cl35903	PRK10263 superfamily
Asppha1_36304_fgenesh1_pm_90_11	superfamily	236669	272	400	0.008098	40.8387	cl35903	PRK10263 superfamily
Penswi1_403498_MIX14692_3843_79	superfamily	236669	297	453	0.008299	39.2979	cl35903	PRK10263 superfamily
Pencam1_13258_transcript_CRL31084	superfamily	236669	297	446	0.008148	39.2979	cl35903	PRK10263 superfamily
Aspfumis1_339395_fgenesh1_pm_14_26	superfamily	236669	332	451	0.009531	38.9127	cl35903	PRK10263 superfamily
Pencit1_491645_gm1_10248_g	superfamily	236669	312	451	0.008299	39.2979	cl35903	PRK10263 superfamily
Penca1_200091_CE65481_7327	superfamily	236669	297	487	5.81E-05	46.2315	cl35903	PRK10263 superfamily
Penanta1_4282_CE4281_619	superfamily	236669	297	500	0.002971	40.8387	cl35903	PRK10263 superfamily
Penant1_4248_PENANT_c002G11587T0	superfamily	236669	297	500	0.002971	40.8387	cl35903	PRK10263 superfamily
Emmcr1_4936_transcript_PGH36816	superfamily	236669	259	408	0.003753	40.4535	cl35903	PRK10263 superfamily
Pendec1_3646_PENDEC_c025G01942T0	superfamily	236669	314	464	0.006997	39.2979	cl35903	PRK10263 superfamily
Monpu1_152560_CE152559_36224	superfamily	236669	344	500	0.006269	39.6831	cl35903	PRK10263 superfamily
Aspcost1_134003_CE134002_810	superfamily	236669	281	451	8.02E-05	45.8463	cl35903	PRK10263 superfamily
Hisca1_1811_HCAG_01708T0	superfamily	236669	347	465	0.000281	43.9203	cl35903	PRK10263 superfamily
Elagr1_3790_transcript_OXV07753	superfamily	236669	258	453	0.000509	43.1499	cl35903	PRK10263 superfamily
Asptep1_1_29620_fgenesh1_pm_171_5	superfamily	236669	297	489	0.000181	46.2315	cl35903	PRK10263 superfamily
Umbpus1_106099_LPUS_04137T0	superfamily	236669	263	579	0.004182	40.4535	cl35903	PRK10263 superfamily
Uncre1_3463_URET_03609	superfamily	236669	267	397	0.000272	43.9203	cl35903	PRK10263 superfamily
Gymci1_1_148462_CE148461_16745	superfamily	236669	276	436	0.007401	39.2979	cl35903	PRK10263 superfamily
Aspven1_382610_fgenesh1_pm_96_	superfamily	236669	226	427	0.001129	43.5351	cl35903	PRK10263 superfamily
Polfu1_142689_CE142688_2993	superfamily	236669	359	531	2.02E-06	51.2391	cl35903	PRK10263 superfamily
Lopma1_588457_e_gw1_10_199_1	superfamily	236669	245	464	3.27E-07	53.5503	cl35903	PRK10263 superfamily
Symko1_924027_fgenesh1_pm_9_270	superfamily	236669	238	449	0.003839	40.4535	cl35903	PRK10263 superfamily
Xanpa2_1181751_CE1181750_31604	superfamily	236669	345	481	0.002503	41.2239	cl35903	PRK10263 superfamily
PseVKM4514_1_6686_transcript_KFY50790	superfamily	236669	269	442	0.00176	41.2239	cl35903	PRK10263 superfamily
Bysci1_235741_CE235740_10874	superfamily	236669	245	502	8.73E-06	48.9279	cl35903	PRK10263 superfamily
Rhiund1_813664_gm1_15810_g	superfamily	236669	342	511	4.48E-05	47.0019	cl35903	PRK10263 superfamily
Lopnu1_487714_e_gw1_7_506_1	superfamily	236669	237	509	9.52E-07	52.0095	cl35903	PRK10263 superfamily
Bimnz1_486664_e_gw1_91_226_1	superfamily	236669	275	441	1.27E-07	55.0911	cl35903	PRK10263 superfamily
Tirniv1_437511_CE437510_6177	superfamily	236669	373	505	0.0005	43.5351	cl35903	PRK10263 superfamily
Tercla1_1267604_gm1_3364_g	superfamily	236669	348	501	2.49E-05	47.7723	cl35903	PRK10263 superfamily
Aurpu_var_mel1_42819_e_gw1_4_795_1	superfamily	236669	244	415	0.001255	41.9943	cl35903	PRK10263 superfamily
Corca1_367650_CE367649_37241	superfamily	236669	284	516	9.2E-06	48.9279	cl35903	PRK10263 superfamily
Currey1_120598_CE120597_17889	superfamily	236669	312	540	6.83E-08	55.8615	cl35903	PRK10263 superfamily
Grascr1_1815_GST1_002658_RA	superfamily	236669	274	538	2.1E-05	47.7723	cl35903	PRK10263 superfamily
Darbet1_199659_CE199658_41150	superfamily	236669	235	512	1.74E-08	57.7875	cl35903	PRK10263 superfamily
Veren1_245083_CE245082_8299	superfamily	236669	262	543	4E-10	63.1803	cl35903	PRK10263 superfamily
Denna1_188722_CE188721_16666	superfamily	236669	251	498	5.04E-06	49.6983	cl35903	PRK10263 superfamily
Daral1_773998_e_gw1_5_394_1	superfamily	236669	229	512	4.05E-08	56.6319	cl35903	PRK10263 superfamily
Daralp1_661448_e_gw1_5_304_1	superfamily	236669	229	512	2.31E-08	57.4023	cl35903	PRK10263 superfamily
Phcapit2_271027_CE271026_54591	superfamily	236669	357	518	3.79E-05	47.0019	cl35903	PRK10263 superfamily
Phcap1_117802_CE117801_56220	superfamily	236669	357	518	3.79E-05	47.0019	cl35903	PRK10263 superfamily
Pcapi1_510433_gm1_4462_g	superfamily	236669	357	518	3.79E-05	47.0019	cl35903	PRK10263 superfamily
Phycap1_250501_e_gw1_5_1033_1	superfamily	236669	357	518	3.79E-05	47.0019	cl35903	PRK10263 superfamily
Phycapi2_433598_e_gw1_5_1393_1	superfamily	236669	357	518	3.79E-05	47.0019	cl35903	PRK10263 superfamily
Phcapi1_200860_e_gw1_4_1020_1	superfamily	236669	357	518	3.79E-05	47.0019	cl35903	PRK10263 superfamily
Aqupat1_661758_fgenesh1_pm_11_297	superfamily	236669	229	509	1.49E-05	48.1575	cl35903	PRK10263 superfamily
Lopnit1_1_412517_CE412516_5305	superfamily	236669	315	388	0.001355	41.6091	cl35903	PRK10263 superfamily
Ascim1_304059_fgenesh1_pg_16_117	superfamily	236669	269	423	0.001454	41.6091	cl35903	PRK10263 superfamily
Amnli1_294988_CE294987_21643	superfamily	236669	272	515	4.36E-08	56.6319	cl35903	PRK10263 superfamily
Bulin1_803694_gm1_3868_g	superfamily	236669	286	470	0.000336	43.5351	cl35903	PRK10263 superfamily
Masph1_480044_e_gw1_1_1805_1	superfamily	236669	272	591	2.1E-05	47.7723	cl35903	PRK10263 superfamily
Microd1_451380_CE451379_26109	superfamily	236669	333	505	9.52E-07	52.0095	cl35903	PRK10263 superfamily

Claaq1_128268_FUN_002853_T1	superfamily	236669	262	539	9.9E-11	65.1063	cl35903	PRK10263 superfamily
Lenfl1_149818_CE149817_34677	superfamily	236669	228	512	5.99E-09	59.3283	cl35903	PRK10263 superfamily
Cloaq1_588537_fgenesh1_pg_71_51	superfamily	236669	234	547	7.94E-05	45.8463	cl35903	PRK10263 superfamily
Herpot1_1_42070_CE42069_24086	superfamily	236669	260	468	4.16E-09	59.7135	cl35903	PRK10263 superfamily
Clagr3_7751_CLAGR_007674_RA	superfamily	236669	338	458	0.003874	40.4535	cl35903	PRK10263 superfamily
Parsp1_222445_CE222444_3318	superfamily	236669	286	514	1.35E-07	54.7059	cl35903	PRK10263 superfamily
Cenge3_688999_fgenesh1_pm_40_58	superfamily	236669	236	397	0.000132	45.0759	cl35903	PRK10263 superfamily
Cenge3_688999_fgenesh1_pm_40_58	superfamily	236669	339	497	0.001279	41.9943	cl35903	PRK10263 superfamily
Pse03VT05_1_9849_transcript_OBT81446	superfamily	236669	252	522	4.04E-05	46.6167	cl35903	PRK10263 superfamily
Melpu1_235860_e_gw1_73_46_1	superfamily	236669	229	511	1.12E-08	58.5579	cl35903	PRK10263 superfamily
Plesi1_375738_e_gw1_5_1386_1	superfamily	236669	229	509	8.73E-10	62.0247	cl35903	PRK10263 superfamily
Maseb1_507332_estExt_fgenesh1_pg_C_90212	superfamily	236669	471	806	5.32E-09	60.0987	cl35903	PRK10263 superfamily
Acrvag1_13361_CE13360_14207	superfamily	236669	312	601	2.34E-05	47.7723	cl35903	PRK10263 superfamily
Lorma1_549405_gm1_9427_g	superfamily	236669	253	546	0.000373	43.5351	cl35903	PRK10263 superfamily
Greabi1_413335_fgenesh1_pg_22_151	superfamily	236669	733	871	4.54E-05	47.3871	cl35903	PRK10263 superfamily
Karrh1_35677_CE35676_6966	superfamily	236669	285	513	8.13E-06	48.9279	cl35903	PRK10263 superfamily
Altso1_122073_ASLCTG_875_jigsaw_mRNA_3	superfamily	236669	469	807	9.59E-05	47.0019	cl35903	PRK10263 superfamily
Alttom1_120644_ATMCTG_3360_jigsaw_mRNA_2	superfamily	236669	424	772	8.7E-05	47.0019	cl35903	PRK10263 superfamily
Altcar1_110133_ACMCTG_2300_jigsaw_mRNA_2	superfamily	236669	385	731	0.000439	44.6907	cl35903	PRK10263 superfamily
Lorju1_391698_gw1_21_45_1	superfamily	236669	269	478	0.002405	40.4535	cl35903	PRK10263 superfamily
Boeex1_103679_CE103678_4355	superfamily	236669	371	523	0.000819	42.3795	cl35903	PRK10263 superfamily
Glost2_401370_fgenesh1_pm_NODE_7478_7	superfamily	236669	236	393	8.37E-05	45.8463	cl35903	PRK10263 superfamily
Phomu1_263058_CE263057_3388	superfamily	236669	367	492	0.000528	43.1499	cl35903	PRK10263 superfamily
ConPMI546_671717_CE671716_31551	superfamily	236669	303	526	1.99E-05	47.7723	cl35903	PRK10263 superfamily
Pseudest1_3676_VC83_00890T0	superfamily	236669	272	438	0.005448	39.2979	cl35903	PRK10263 superfamily
Epini1_331387_e_gw1_10_693_1	superfamily	236669	270	425	2.94E-06	50.4687	cl35903	PRK10263 superfamily
Leppa1_297828_e_gw1_159_18_1	superfamily	236669	300	487	4.19E-05	46.6167	cl35903	PRK10263 superfamily
Pyrsp1_607587_fgenesh1_pm_2_22	superfamily	236669	230	489	7.75E-09	58.5579	cl35903	PRK10263 superfamily
Altte1_119648_AT2CTG_637_jigsaw_mRNA_171	superfamily	236669	279	599	1.87E-06	51.2391	cl35903	PRK10263 superfamily
Altal1_251246_CE251245_83928	superfamily	236669	280	600	1.83E-06	51.2391	cl35903	PRK10263 superfamily
Altar1_113069_AABCTG_2978_jigsaw_mRNA_1	superfamily	236669	318	638	1.82E-06	51.2391	cl35903	PRK10263 superfamily
Altfr1_243159_AFGCTG_701_jigsaw_mRNA_1	superfamily	236669	279	599	1.42E-06	51.6243	cl35903	PRK10263 superfamily
Altlo1_257016_ALGCTG_2604_jigsaw_mRNA_59	superfamily	236669	327	449	4.47E-05	46.6167	cl35903	PRK10263 superfamily
Alalt1_112159_ATNCTG_56_jigsaw_mRNA_172	superfamily	236669	386	706	1.18E-06	52.0095	cl35903	PRK10263 superfamily
Alalte1_110008_AATCTG_420_jigsaw_mRNA_70	superfamily	236669	299	619	2.12E-06	50.8539	cl35903	PRK10263 superfamily
Aalte1_112641_AA2CTG_134_jigsaw_mRNA_167	superfamily	236669	279	599	1.58E-06	51.2391	cl35903	PRK10263 superfamily
Altalt1_227087_CE227086_18892	superfamily	236669	280	600	3.08E-06	50.4687	cl35903	PRK10263 superfamily
ColoR110_1_162177_estExt_fgenesh1_pg_C_1230004	superfamily	236669	346	522	7.44E-05	45.8463	cl35903	PRK10263 superfamily
DelFL0756_1_194243_CE194242_34694	superfamily	236669	264	398	3.1E-05	47.3871	cl35903	PRK10263 superfamily
Altli1_251267_ATKCTG_2192_jigsaw_mRNA_5	superfamily	236669	279	599	3.37E-06	50.4687	cl35903	PRK10263 superfamily
Sceap1_4492_transcript_KEZ46129	superfamily	236669	252	441	0.000456	43.1499	cl35903	PRK10263 superfamily
Phycitr1_620275_fgenesh1_pm_54_11	superfamily	236669	353	501	0.001922	41.2239	cl35903	PRK10263 superfamily
Phycpc1_483960_fgenesh1_pm_31_63	superfamily	236669	353	501	0.001922	41.2239	cl35903	PRK10263 superfamily
Parch1_38214_CE38213_17049	superfamily	236669	286	590	7.83E-08	55.4763	cl35903	PRK10263 superfamily
Parchr1_438799_e_gw1_6_933_1	superfamily	236669	286	590	1.43E-07	54.7059	cl35903	PRK10263 superfamily
Calful1_768127_fgenesh1_pg_70_22	superfamily	236669	389	540	5.7E-05	46.6167	cl35903	PRK10263 superfamily
Spofi1_481125_gm1_8175_g	superfamily	236669	234	422	4.22E-05	46.6167	cl35903	PRK10263 superfamily
Spofi1_481125_gm1_8175_g	superfamily	236669	371	515	0.000657	42.7647	cl35903	PRK10263 superfamily
Pyrtr1_149332_PTRT_03646	superfamily	236669	289	530	0.0006	43.1499	cl35903	PRK10263 superfamily
Pcit120373_276358_e_gw1_37_80_1	superfamily	236669	363	518	6.32E-05	46.2315	cl35903	PRK10263 superfamily
Pcit11120_567181_e_gw1_45_88_1	superfamily	236669	363	518	6.81E-05	45.8463	cl35903	PRK10263 superfamily
Pcit122670_533140_e_gw1_45_32_1	superfamily	236669	363	518	6.64E-05	46.2315	cl35903	PRK10263 superfamily
Pcit131864_796156_e_gw1_46_14_1	superfamily	236669	363	518	6.64E-05	46.2315	cl35903	PRK10263 superfamily
Pcit141352_550346_e_gw1_72_8_1	superfamily	236669	363	518	6.64E-05	46.2315	cl35903	PRK10263 superfamily
Phy27169_388894_fgenesh1_pm_79_9	superfamily	236669	360	508	0.001793	41.6091	cl35903	PRK10263 superfamily
Phypa1_507687_e_gw1_32_21_1	superfamily	236669	363	518	6.64E-05	46.2315	cl35903	PRK10263 superfamily
Phycit1_213906_CE213905_38770	superfamily	236669	362	519	0.000777	42.7647	cl35903	PRK10263 superfamily
Pcitri2_232290_CE232289_12775	superfamily	236669	362	519	0.000777	42.7647	cl35903	PRK10263 superfamily
Cocsa1_258482_CE53923_12395	superfamily	236669	227	525	6.31E-05	46.2315	cl35903	PRK10263 superfamily
Chalo1_235725_CE235724_44958	superfamily	236669	350	469	0.001197	41.6091	cl35903	PRK10263 superfamily
Paront1_36026_e_gw1_44_129_1	superfamily	236669	300	494	1.01E-06	52.0095	cl35903	PRK10263 superfamily
Lepmi1_353822_e_gw1_9_827_1	superfamily	236669	275	455	7.24E-06	49.3131	cl35903	PRK10263 superfamily
Stasp1_126978_CE126977_15020	superfamily	236669	277	590	4.74E-06	49.6983	cl35903	PRK10263 superfamily
Setho1_78401_e_gw1_137_63_1	superfamily	236669	256	587	8.26E-06	48.9279	cl35903	PRK10263 superfamily
Decga1_204733_CE204732_18188	superfamily	236669	310	523	1.64E-07	54.7059	cl35903	PRK10263 superfamily
Phapo1_602669_e_gw1_2_798_1	superfamily	236669	274	596	0.000288	43.9203	cl35903	PRK10263 superfamily
Altro1_194625_CE194624_13192	superfamily	236669	311	488	5.19E-05	46.6167	cl35903	PRK10263 superfamily

Sorbr1_252747_CE252746_15970	superfamily	236669	252	459	7.22E-05	45.8463	cl35903	PRK10263 superfamily
Magor1_7389_rna_MGG_08556T0	superfamily	236669	348	559	0.00054	43.1499	cl35903	PRK10263 superfamily
CopCBS38678_1_337827_e_gw1_134_23_1	superfamily	236669	292	439	0.009904	38.9127	cl35903	PRK10263 superfamily
Sorhu1_37672_CE37671_3344	superfamily	236669	297	423	0.003203	40.4535	cl35903	PRK10263 superfamily
Sorma1_5067_transcript_CCC07417	superfamily	236669	297	423	0.003203	40.4535	cl35903	PRK10263 superfamily
Conioc1_1210519_gm1_1167_g	superfamily	236669	374	521	0.001871	41.2239	cl35903	PRK10263 superfamily
Conioc1_1211268_gm1_1916_g	superfamily	236669	366	458	0.000681	42.7647	cl35903	PRK10263 superfamily
Mabrmo1_2765_g3750_t1	superfamily	236669	349	533	0.008161	39.2979	cl35903	PRK10263 superfamily
Myrdu1_318633_estExt_Genemark1_C_2_t10445	superfamily	236669	307	448	0.000364	43.5351	cl35903	PRK10263 superfamily
Sclbo1_3923_transcript_ESZ95703	superfamily	236669	334	553	0.008175	39.2979	cl35903	PRK10263 superfamily
Delst1_367146_fgenesh1_pg_3_413	superfamily	236669	286	382	0.0002	44.3055	cl35903	PRK10263 superfamily
Lophiu1_245295_CE245294_33806	superfamily	236669	230	425	2.15E-06	50.8539	cl35903	PRK10263 superfamily
Sclsan1_154033_CE154032_4218	superfamily	236669	253	459	2.9E-07	53.5503	cl35903	PRK10263 superfamily
Neobo1_511409_e_gw1_19_91_1	superfamily	236669	321	453	0.003774	40.0683	cl35903	PRK10263 superfamily
Trieva1_107016_CE107015_46155	superfamily	236669	321	470	0.000128	45.0759	cl35903	PRK10263 superfamily
Stobe1_85660_fgenesh1_pm_7_83	superfamily	236669	287	486	7.37E-07	52.3947	cl35903	PRK10263 superfamily
Altsp012_1_265874_CE265873_14578	superfamily	236669	329	481	0.003593	40.4535	cl35903	PRK10263 superfamily
Altsp017_1_254432_CE254431_8397	superfamily	236669	292	596	0.007165	39.2979	cl35903	PRK10263 superfamily
Phimu1_351158_CE351157_50339	superfamily	236669	299	451	2.84E-05	47.0019	cl35903	PRK10263 superfamily
Cryan3_116812_CE116811_9156	superfamily	236669	307	525	0.001044	42.3795	cl35903	PRK10263 superfamily
Cadosp1_455305_e_gw1_11_139_1	superfamily	236669	275	495	0.00016	44.6907	cl35903	PRK10263 superfamily
Theglo1_144858_CE144857_41804	superfamily	236669	266	441	0.001849	41.2239	cl35903	PRK10263 superfamily
AcreTS7_1_508495_CE508494_14679	superfamily	236669	243	407	1.87E-06	50.4687	cl35903	PRK10263 superfamily
Marbr1_2001_MBM_05547	superfamily	236669	175	321	0.001367	43.9203	cl35903	PRK10263 superfamily
Tribre1_123693_g10222_t1	superfamily	236669	248	455	9.53E-05	45.4611	cl35903	PRK10263 superfamily
Tribrev1_416765_fgenesh1_pm_20_122	superfamily	236669	248	455	9.53E-05	45.4611	cl35903	PRK10263 superfamily
Triaru1_5241_rna5936	superfamily	236669	248	455	0.000172	44.6907	cl35903	PRK10263 superfamily
Cryan3_349102_CE349101_9152	superfamily	236669	306	524	0.001026	42.3795	cl35903	PRK10263 superfamily
Racan1_11240_transcript_OQN99911	superfamily	236669	288	580	0.002199	41.2239	cl35903	PRK10263 superfamily
Crypo1_476118_gm1_7604_g	superfamily	236669	381	516	0.001435	41.6091	cl35903	PRK10263 superfamily
Alikh1_653463_e_gw1_417_42_1	superfamily	236669	160	287	3.43E-05	46.6167	cl35903	PRK10263 superfamily
Aspaurlat1_305250_MIX1778_78_46	superfamily	236768	1081	1189	0.000465	43.5177	cl35954	PRK10819 superfamily
Aspamoe1_219996_gm1_1720_g	superfamily	237057	294	500	0.006338	41.0147	cl36108	PRK12323 superfamily
Aspve1_78996_gm1_1464_g	superfamily	237057	294	500	0.006744	40.6295	cl36108	PRK12323 superfamily
Aspaus1_957779_fgenesh1_pm_11_72	superfamily	237057	294	500	0.006836	40.6295	cl36108	PRK12323 superfamily
Aspind1_464315_MIX2028_2160_32	superfamily	237057	264	441	0.00254	40.6295	cl36108	PRK12323 superfamily
Xylhe1_240511_fgenesh1_pg_9_209	superfamily	237057	289	453	1.37E-06	51.4151	cl36108	PRK12323 superfamily
FvanT33_1_317272_fgenesh1_pm_27_27	superfamily	237057	254	456	1.17E-05	47.9483	cl36108	PRK12323 superfamily
FvanT36_1_30538_CE30537_38119	superfamily	237057	254	456	1.18E-05	47.9483	cl36108	PRK12323 superfamily
FvanT77_1_366473_gm1_12667_g	superfamily	237057	254	456	1.18E-05	47.9483	cl36108	PRK12323 superfamily
FvanT78_1_303387_MIX14106_9913_88	superfamily	237057	254	456	1.18E-05	47.9483	cl36108	PRK12323 superfamily
FvanT95_1_282311_fgenesh1_pm_6_241	superfamily	237057	254	456	1.18E-05	47.9483	cl36108	PRK12323 superfamily
FusspF11_1_913638_gm1_374_g	superfamily	237057	254	456	1.18E-05	47.9483	cl36108	PRK12323 superfamily
FvanT110_1_335862_CE335861_85793	superfamily	237057	254	456	1.18E-05	47.9483	cl36108	PRK12323 superfamily
FvanT126_1_444349_gm1_5114_g	superfamily	237057	254	456	1.18E-05	47.9483	cl36108	PRK12323 superfamily
FvanT159_1_478216_gm1_230_g	superfamily	237057	254	456	1.18E-05	47.9483	cl36108	PRK12323 superfamily
Fusva1_581107_MIX5788_21615_91	superfamily	237057	254	456	1.17E-05	47.9483	cl36108	PRK12323 superfamily
Fusvan1_249026_CE249025_59849	superfamily	237057	254	456	1.18E-05	47.9483	cl36108	PRK12323 superfamily
FvanT200_1_50449_CE50448_11327	superfamily	237057	254	456	1.17E-05	47.9483	cl36108	PRK12323 superfamily
FvanT219_1_339734_gm1_7692_g	superfamily	237057	254	456	1.17E-05	47.9483	cl36108	PRK12323 superfamily
FvanT23_1_390999_fgenesh1_pm_49_66	superfamily	237057	254	427	2E-05	47.1779	cl36108	PRK12323 superfamily
FvanT299_1_346769_fgenesh1_pg_11_518	superfamily	237057	254	456	1.17E-05	47.9483	cl36108	PRK12323 superfamily
FvanT31_1_2669_CE2668_10684	superfamily	237057	254	456	1.18E-05	47.9483	cl36108	PRK12323 superfamily
Tristr1_341931_gm1_7708_g	superfamily	237057	225	444	1.28E-05	47.9483	cl36108	PRK12323 superfamily
Triint1_304420_CE304419_25048	superfamily	237057	225	465	5.42E-06	49.1039	cl36108	PRK12323 superfamily
Triaet1_161752_CE161751_14399	superfamily	237057	310	502	3.52E-05	46.7927	cl36108	PRK12323 superfamily
TriviGv29_8_2_164251_e_gw1_91_653_1	superfamily	237057	331	464	1.71E-06	50.6447	cl36108	PRK12323 superfamily
Trifla1_309717_CE309716_18569	superfamily	237057	325	481	0.000136	44.8667	cl36108	PRK12323 superfamily
Triam1_347408_CE347407_16788	superfamily	237057	315	482	0.000429	43.3259	cl36108	PRK12323 superfamily
Tripleu1_63953_CE63952_18853	superfamily	237057	315	482	0.000429	43.3259	cl36108	PRK12323 superfamily
Tri5640_1_102838_CE102837_9337	superfamily	237057	320	475	0.000112	44.8667	cl36108	PRK12323 superfamily
Trich5757_1_289516_CE289515_10885	superfamily	237057	313	481	0.00376	40.2443	cl36108	PRK12323 superfamily
Triafa1_64506_CE64505_13312	superfamily	237057	313	481	0.001958	41.0147	cl36108	PRK12323 superfamily
Dacma1_450764_CE450763_40139	superfamily	237057	305	450	0.008061	38.7035	cl36108	PRK12323 superfamily
Trialn1_333503_fgenesh1_pm_7_126	superfamily	237057	320	476	0.00028	43.7111	cl36108	PRK12323 superfamily
Asptri1_201493_fgenesh1_pm_666_1	superfamily	183854	953	1023	0.001	42.6977	cl32851	PRK13042 superfamily
Aspund1_1_278765_fgenesh1_pm_1_321	superfamily	139494	1086	1186	0.000152	45.5022	cl31400	PRK13335 superfamily
Porpun1_592424_MIX1098_29595_36	superfamily	184281	328	419	0.000207	43.6583	cl42933	PRK13729 superfamily
Aspaura1_203970_gm1_7400_g	superfamily	237864	1079	1214	0.005747	40.9484	cl36446	PRK14950 superfamily
Penac1_441878_fgenesh1_pm_2_446	superfamily	237864	366	457	0.005448	39.4076	cl36446	PRK14950 superfamily
Claps1_1788_A1O5_07176T0	superfamily	237864	562	645	0.000158	45.1856	cl36446	PRK14950 superfamily
Talma1_2_3120_PMAA_068430	superfamily	237874	373	469	0.000195	44.3811	cl36455	PRK14971 superfamily

	Aspnov1_431072_fgenesh1_pg_4_553	superfamily	237874	302	411	0.006124	39.3735	cl36455	PRK14971 superfamily
	Plemel1_105900_CE105899_14980	superfamily	237874	512	586	0.000462	43.2255	cl36455	PRK14971 superfamily
	Aspbre1_59200_CE59199_1577	superfamily	185594	332	534	0.001018	41.9843	cl33180	PTZ00395 superfamily
	Tryvi1_440647_e_gw1_6_229_1	superfamily	185594	253	451	0.001306	41.9843	cl33180	PTZ00395 superfamily
	Rhyag1_13472_transcript_CZT07453	superfamily	185594	248	478	2.21E-05	47.3771	cl33180	PTZ00395 superfamily
	Zasce1_69855_fgenesh1_pm_42_24	superfamily	185594	344	494	0.003173	40.4436	cl33180	PTZ00395 superfamily
	Rhyco1_8923_transcript_CZT09395	superfamily	185594	248	452	0.0001	45.4511	cl33180	PTZ00395 superfamily
	Rhyse1_7053_transcript_CZT48850	superfamily	185594	248	478	3.22E-06	50.0735	cl33180	PTZ00395 superfamily
	Verga1_121575_PV09_08469T0	superfamily	185594	214	447	0.005407	39.6732	cl33180	PTZ00395 superfamily
	Hymvar1_278074_CE278073_24053	superfamily	185594	412	577	0.005162	40.0584	cl33180	PTZ00395 superfamily
	Plecto1_55328_CE55327_24102	superfamily	185594	266	382	0.000794	42.3695	cl33180	PTZ00395 superfamily
	Plecuc1_325164_gm1_2614_g	superfamily	185594	312	390	0.00017	44.6807	cl33180	PTZ00395 superfamily
	Astsub1_153682_CE153681_27034	superfamily	185594	276	429	0.003956	40.0584	cl33180	PTZ00395 superfamily
	Xylhel2_504945_fgenesh1_pm_14_60	superfamily	185594	297	470	5.31E-06	49.3031	cl33180	PTZ00395 superfamily
	Podjug1_571548_gm1_3394_g	superfamily	185594	236	360	0.001786	40.8288	cl33180	PTZ00395 superfamily
	Nemsp1_23131_fgenesh1_pg_8_80	superfamily	185594	241	400	0.004748	39.6732	cl33180	PTZ00395 superfamily
	Asphey1_11489_fgenesh1_pg_1_159	superfamily	185628	985	1268	0.000201	45.8362	cl33186	PTZ00449 superfamily
	Asphor1_149261_fgenesh1_pm_8_33	superfamily	185628	1017	1228	0.000128	46.2214	cl33186	PTZ00449 superfamily
	Aspala1_143485_fgenesh1_pm_6_241	superfamily	185628	1029	1240	6.58E-05	47.377	cl33186	PTZ00449 superfamily
	Asppseute1_423105_e_gw1_195_254_1	superfamily	185628	1033	1244	0.000135	46.2214	cl33186	PTZ00449 superfamily
	Lizem1_101759_e_gw1_5_1050_1	superfamily	185628	381	565	0.000928	42.3694	cl33186	PTZ00449 superfamily
	Verped1_331869_e_gw1_27_134_1	superfamily	185628	352	488	0.000734	42.3694	cl33186	PTZ00449 superfamily
	Color1_5452_CORC01_13689T0	superfamily	185628	332	406	0.00338	40.4434	cl33186	PTZ00449 superfamily
	Colto1_5511_CT0861_01833T0	superfamily	185628	327	501	7.73E-05	45.451	cl33186	PTZ00449 superfamily
	Colce1_776991_gm1_989_g	superfamily	185628	322	476	0.000957	41.9842	cl33186	PTZ00449 superfamily
	Colsu1_492272_CE492271_15446	superfamily	185628	321	529	0.000298	43.525	cl33186	PTZ00449 superfamily
	Colsub1_7642_transcript_KDN70510	superfamily	185628	321	529	0.000298	43.525	cl33186	PTZ00449 superfamily
	Coler1_524697_e_gw1_14_145_1	superfamily	185628	321	529	0.000507	43.1398	cl33186	PTZ00449 superfamily
	Acrchr1_5707_ACRE_045050T0	superfamily	185628	317	487	0.004748	39.673	cl33186	PTZ00449 superfamily
	Colhig2_13941_CH63R_12733T0	superfamily	185628	323	509	2.64E-05	46.9918	cl33186	PTZ00449 superfamily
	Triver1_2238_FLIJena_TRV_00043	superfamily	273167	466	526	0.006608	39.1079	cl36702	rad23 superfamily
	Aspspe1_1_182093_fgenesh1_pm_2_66	superfamily	236766	1068	1226	0.005677	41.1785	cl35953	rne superfamily
	Aspegy1_533726_fgenesh1_pm_73_2	superfamily	445260	916	985	0.001502	38.7666	cl01090	SlyX superfamily
	Aspins1_294536_fgenesh1_pm_13_15	superfamily	274009	749	976	0.000398	44.6735	cl37070	SMC_prok_A superfamily
	Aspth1_360369_fgenesh1_pm_21_90	superfamily	274009	762	989	0.001882	42.7475	cl37070	SMC_prok_A superfamily
	Asphethal1_273971_fgenesh1_pm_4_107	superfamily	274009	791	1026	0.000425	44.6735	cl37070	SMC_prok_A superfamily
	XylFL0016_426616_MIX4792_21201_86	superfamily	434592	251	412	0.000515	42.1003	cl25880	SOBP superfamily
	PhaeoFL0889_1_310332_CE310331_40217	superfamily	425992	332	401	0.001028	41.4814	cl03073	TYA superfamily
	Phisc1_1899_CE1898_951	superfamily	452900	600	671	2.49E-11	59.497	cl28922	Ubl1_cv_Nsp3_N-like superfamily
	Chove1_1787637_fgenesh1_pg_17_140	superfamily	113583	383	463	0.004258	39.4965	cl04774	Umbravirus_LDM superfamily
	Tubae1_7625_GSTUAT00000625001	superfamily	113583	384	464	0.006831	38.7261	cl04774	Umbravirus_LDM superfamily
	Tubmes1_3624216_fgenesh1_pg_75_49	superfamily	113583	384	464	0.007681	38.7261	cl04774	Umbravirus_LDM superfamily
	Aspegy1_533726_fgenesh1_pm_73_2	superfamily	432760	584	717	0.004969	40.7772	cl15068	Vac7 superfamily
	Phisc1_1899_CE1898_951	specific	395120	503	537	7.07E-11	57.3077	pfam00172	Zn_clus
PeZ-VelB	OphPMI507_1_406450_fgenesh1_pm_6_306	superfamily	397207	1	52	5.4E-05	43.5043	cl37681	Amelogenin superfamily
	Matter1_275085_CE275084_8590	superfamily	427171	3	59	0.001186	40.9054	cl38111	Atrophin-1 superfamily
	Tirniv1_28098_CE28097_13283	superfamily	427171	7	70	0.002452	39.7498	cl38111	Atrophin-1 superfamily
	Annnit1_350601_CE350600_16102	superfamily	227547	4	56	0.005047	38.9596	cl34947	COG5222 superfamily
	AnFL0455_28318_CE28317_52013	superfamily	227547	4	56	0.007501	38.5744	cl34947	COG5222 superfamily
	Anntru1_164755_CE164754_11841	superfamily	227547	4	56	0.007344	38.5744	cl34947	COG5222 superfamily
	HyFL0890_1_164840_CE164839_15242	superfamily	227547	4	56	0.006947	38.5744	cl34947	COG5222 superfamily
	HyNC0597_1_547653_CE547652_53993	superfamily	227547	4	56	0.00728	38.5744	cl34947	COG5222 superfamily
	HyFL0543_1_208292_CE208291_9727	superfamily	227547	4	56	0.007194	38.5744	cl34947	COG5222 superfamily
	Venin1_14690_atg2237_t1	superfamily	227637	7	605	6.6E-136	434.51	cl34984	COG5329 superfamily
	Pseel1_9490_g9136_t1	superfamily	451501	24	258	6.49E-49	173.304	cl23717	crotonase-like superfamily
	Rutfi1_622838_fgenesh1_pg_6_218	superfamily	455861	6	131	0.003286	39.8088	cl42516	dnaA superfamily
	Aspber1_106503_CE106502_457	superfamily	455861	147	215	0.006115	38.6532	cl42516	dnaA superfamily
	Hypinv1_686988_gm4_6360_g	superfamily	455861	2	112	0.004097	39.4236	cl42516	dnaA superfamily
	HyFL1284_2_348230_CE348229_33362	superfamily	455861	3	114	0.000419	42.5052	cl42516	dnaA superfamily
	HyFL1857_1_139956_CE139955_6331	superfamily	455861	2	112	0.001376	40.9644	cl42516	dnaA superfamily
	Hypcer1_156440_CE156439_6657	superfamily	455861	2	114	8.54E-05	44.8164	cl42516	dnaA superfamily
	DimP5P66_572132_e_gw1_25_348_1	superfamily	455861	213	334	0.000761	42.12	cl42516	dnaA superfamily
	HypEC38_3_246525_CE246524_21698	superfamily	455861	2	112	0.004097	39.4236	cl42516	dnaA superfamily
	Hypfra2_526887_MIX214_49028_81	superfamily	455861	2	114	0.000924	41.7348	cl42516	dnaA superfamily
	XylFL0662B_640885_MIX8298_28787_64	superfamily	455861	2	112	4.01E-05	45.972	cl42516	dnaA superfamily
	Jumvir1_332470_gm4_1252_g	superfamily	455861	2	112	0.000831	41.7348	cl42516	dnaA superfamily
	Aspor1_1247_AO090005000898_mRNA	superfamily	455861	100	168	0.00918	37.4976	cl42516	dnaA superfamily
	Schcon1_251754_e_gw1_10_244_1	superfamily	455861	190	283	0.007746	38.6532	cl42516	dnaA superfamily
	AcreTS7_1_143193_CE143192_6343	superfamily	455861	3	128	0.001036	41.3496	cl42516	dnaA superfamily

Diadis1_852619_gm4_11879_g	superfamily	455861	7	103	0.008256	38.6532	cl42516	dnaA superfamily
FoxMRL8996_20006_CE20005_21708	superfamily	455861	258	336	0.003001	40.194	cl42516	dnaA superfamily
Mictr1_40713_CE40712_4471	superfamily	455861	192	329	0.000136	44.4312	cl42516	dnaA superfamily
Hisca1_4559_HCAG_03289T0	specific	372351	131	193	0.006052	36.6427	pfam12868	DUF3824
Atrpi1_427855_gw1_26_319_1	specific	372351	112	187	0.000141	41.6503	pfam12868	DUF3824
Venin1_14690_atg2237_t1	superfamily	444936	628	930	5.9E-149	458.728	cl00490	EEP superfamily
Thiap1_629811_fgenesh1_pg_1_607	superfamily	425360	2	79	0.001771	40.0167	cl41729	KLF1_2_4_N superfamily
Triatro1_496069_estExt_Genemark1_C_4_t20416	superfamily	425360	41	137	0.006992	38.4862	cl41729	KLF1_2_4_N superfamily
Triat2_175759_gw1_24_1198_1	superfamily	425360	20	116	0.005982	38.8714	cl41729	KLF1_2_4_N superfamily
Tristr1_330518_fgenesh1_pm_4_433	superfamily	425360	41	138	0.000894	41.5678	cl41729	KLF1_2_4_N superfamily
Thipu1_4259_transcript_KKA31188	superfamily	425360	4	90	8.65E-07	50.4274	cl41729	KLF1_2_4_N superfamily
Coltof1_151894_mrna_11962	superfamily	425360	12	124	0.00351	39.6418	cl41729	KLF1_2_4_N superfamily
Trsp14807_422414_gm1_1034_g	superfamily	425360	41	118	0.000443	42.3382	cl41729	KLF1_2_4_N superfamily
Trich5757_1_363375_gm1_2451_g	superfamily	425360	45	116	0.003189	39.6418	cl41729	KLF1_2_4_N superfamily
Trichpleur1_115589_CE115588_9372	superfamily	425360	45	116	0.003197	39.6418	cl41729	KLF1_2_4_N superfamily
TriTUC5505_1_380557_fgenesh1_pm_11_233	superfamily	425360	41	118	0.000452	42.3382	cl41729	KLF1_2_4_N superfamily
Trihar1_765194_estExt_Genewise1Plus_C_7160016	superfamily	425360	42	118	0.005981	38.8714	cl41729	KLF1_2_4_N superfamily
Triinh1_244336_CE244335_20274	superfamily	425360	41	118	0.000255	43.1086	cl41729	KLF1_2_4_N superfamily
Trigui1_9755_transcript_OPB43501	superfamily	425360	41	120	0.000685	41.953	cl41729	KLF1_2_4_N superfamily
Trichl1_201667_CE201666_11505	superfamily	425360	44	123	0.000122	44.2642	cl41729	KLF1_2_4_N superfamily
Triam1_118465_CE118464_10836	superfamily	425360	45	116	0.004081	39.2566	cl41729	KLF1_2_4_N superfamily
Tripleu1_428883_gm1_9837_g	superfamily	425360	45	116	0.004081	39.2566	cl41729	KLF1_2_4_N superfamily
PseVKM4514_1_3566_transcript_KFY49258	superfamily	312941	1	132	9.89E-05	44.9992	cl26621	Med15 superfamily
Hymvar1_383815_e_gw1_23_406_1	superfamily	130689	157	291	0.000941	41.331	cl31127	PABP-1234 superfamily
Chafu1_19811_CE19810_6907	superfamily	130689	1	91	0.00073	41.7162	cl31127	PABP-1234 superfamily
Cha1176438_49774_gm1_2568_g	superfamily	130689	1	91	0.00073	41.7162	cl31127	PABP-1234 superfamily
Rutfi1_622838_fgenesh1_pg_6_218	superfamily	130689	217	347	0.00142	40.9458	cl31127	PABP-1234 superfamily
Sceap1_6147_transcript_KEZ44234	superfamily	130689	236	348	0.001471	40.9458	cl31127	PABP-1234 superfamily
Neo1551_1_516674_fgenesh1_pg_7_468	superfamily	130689	3	102	0.004098	39.405	cl31127	PABP-1234 superfamily
Lompr1_7146_transcript_PKS10546	superfamily	130689	211	337	9.65E-05	44.7978	cl31127	PABP-1234 superfamily
Madmy1_8370_transcript_KXX75111	superfamily	130689	1	93	1.27E-05	47.4942	cl31127	PABP-1234 superfamily
Apope1_526361_fgenesh1_pg_1_257	superfamily	130689	14	118	0.000663	42.1014	cl31127	PABP-1234 superfamily
Lopnit1_1_882391_estExt_fgenesh1_pg_C_200005	superfamily	130689	203	312	0.001191	41.331	cl31127	PABP-1234 superfamily
Metani1_2354_transcript_KID62818	superfamily	130689	11	130	0.000781	42.1014	cl31127	PABP-1234 superfamily
Metac1_4580_MAC_04580m_01	superfamily	130689	66	155	0.000316	42.8718	cl31127	PABP-1234 superfamily
Verdah1_134688_CE134687_6187	superfamily	130689	5	123	0.000366	42.8718	cl31127	PABP-1234 superfamily
Verda1_5063_VDAG_08715T0	superfamily	130689	5	123	0.000366	42.8718	cl31127	PABP-1234 superfamily
Verlo1_11709_transcript_CRK29048	superfamily	130689	5	123	0.000432	42.8718	cl31127	PABP-1234 superfamily
Plecuc1_16114_CE16113_6414	superfamily	130689	72	146	0.000998	41.331	cl31127	PABP-1234 superfamily
Amyenc1_483564_gm1_4464_g	superfamily	130689	186	316	3.99E-06	49.035	cl31127	PABP-1234 superfamily
Colto1_12009_CT0861_12714T0	superfamily	130689	17	122	0.002861	39.7902	cl31127	PABP-1234 superfamily
Coltof1_151894_mrna_11962	superfamily	130689	78	183	0.002829	40.1754	cl31127	PABP-1234 superfamily
XyFL0641_1_188314_CE188313_22407	superfamily	130689	6	109	0.00215	40.1754	cl31127	PABP-1234 superfamily
Sepmu1_151278_estExt_fgenesh1_kg_C_90397	superfamily	130689	97	192	0.000211	43.6422	cl31127	PABP-1234 superfamily
Mictr1_40713_CE40712_4471	superfamily	130689	3	107	0.00026	43.257	cl31127	PABP-1234 superfamily
Chain1_1910_g9825_t1	superfamily	130689	4	115	0.000714	42.1014	cl31127	PABP-1234 superfamily
Zalva1_485242_e_gw1_16_406_1	superfamily	130689	184	289	0.000277	43.257	cl31127	PABP-1234 superfamily
Metma1_3325_XM_014722953_1	superfamily	401645	23	151	0.000486	42.7156	cl37801	PAT1 superfamily
Metro1_5307_MAA_00244T0	superfamily	401645	23	151	0.000609	42.3304	cl37801	PAT1 superfamily
Trimi1_164_CE163_26028	superfamily	401645	45	161	0.00132	41.56	cl37801	PAT1 superfamily
Metbr1_9792_XM_014688378_1	superfamily	401645	23	151	0.00057	42.3304	cl37801	PAT1 superfamily
Bissp1_5097_CE5096_6905	superfamily	401645	117	275	0.000235	43.486	cl37801	PAT1 superfamily
Verlo1_4104_transcript_CRK15280	superfamily	401645	3	114	5.9E-06	48.8788	cl37801	PAT1 superfamily
Triple1_114784_g8068_t1	superfamily	401645	47	143	0.001969	40.7896	cl37801	PAT1 superfamily
Clasph1_6086_CSPH_05655_R0	superfamily	227507	97	138	0.001761	40.4775	cl34930	PBP1 superfamily
Crymi1_2023_B0A49_11289	superfamily	227507	144	243	1.41E-05	47.0259	cl34930	PBP1 superfamily
Colgr1_1810_GLRG_04463T0	superfamily	226193	2	85	0.000231	42.9417	cl43925	PcoB superfamily
XylFL1019_137951_CE137950_18915	superfamily	330753	21	114	0.001841	40.4564	cl25932	Peptidase_S21 superfamily
Annbov1_251920_CE251919_14019	superfamily	223021	5	144	0.001541	41.0773	cl33720	PHA03247 superfamily
Annmin1_329283_gm1_490_g	superfamily	223021	5	145	0.000462	43.0033	cl33720	PHA03247 superfamily
Annsty1_155322_CE155321_7353	superfamily	223021	5	145	0.003174	40.3069	cl33720	PHA03247 superfamily
Roster1_494467_gm1_6620_g	superfamily	223021	5	145	0.003174	40.3069	cl33720	PHA03247 superfamily
Daldec1_224570_CE224569_8271	superfamily	223021	6	145	0.00364	39.9217	cl33720	PHA03247 superfamily
Dales1_423710_MIX294_11467_71	superfamily	223021	6	145	0.003852	39.9217	cl33720	PHA03247 superfamily
DallocAZ0526_2_490751_fgenesh1_pm_2_85	superfamily	223021	6	145	0.002816	40.3069	cl33720	PHA03247 superfamily
Dalloc1_313432_gm1_2561_g	superfamily	223021	6	145	0.002234	40.6921	cl33720	PHA03247 superfamily
Dallocu1_388845_gm1_6813_g	superfamily	223021	6	145	0.003899	39.9217	cl33720	PHA03247 superfamily
DaFL1419_1_367082_MIX5342_4334_60	superfamily	223021	6	145	0.00217	40.6921	cl33720	PHA03247 superfamily
Dalver1_267486_MIX156_2696_43	superfamily	223021	6	145	0.002034	40.6921	cl33720	PHA03247 superfamily
Hypmon1_287138_MIX702_18639_70	superfamily	223021	5	145	0.001708	41.0773	cl33720	PHA03247 superfamily
Hyprub1_179356_CE179355_14308	superfamily	223021	18	147	0.001381	41.4625	cl33720	PHA03247 superfamily
Hypsub2_156754_CE156753_20272	superfamily	223021	5	145	0.005171	39.5365	cl33720	PHA03247 superfamily
Hypcro1_458351_MIX184_25796_68	superfamily	223021	5	142	0.000655	42.2329	cl33720	PHA03247 superfamily
Hypfus1_15792_CE15791_11059	superfamily	223021	7	147	0.000117	44.9293	cl33720	PHA03247 superfamily
Hypvin1_356358_fgenesh1_pg_1_757	superfamily	223021	7	157	0.00084	42.2329	cl33720	PHA03247 superfamily
Hyptru1_380867_CE380866_10070	superfamily	223021	5	145	0.003691	39.9217	cl33720	PHA03247 superfamily

	HyprubER1909_1_3805_CE3804_5993	superfamily	223021	7	147	0.001267	41.4625	cl33720	PHA03247 superfamily
	HyFL1150_1_80045_CE80044_37373	superfamily	223021	18	147	0.00243	40.6921	cl33720	PHA03247 superfamily
	Whamic1_482316_CE482315_15215	superfamily	223021	5	147	0.000822	42.2329	cl33720	PHA03247 superfamily
	Tryvi1_528452_gm1_798_g	superfamily	223021	179	244	0.000247	43.3885	cl33720	PHA03247 superfamily
	Xylarb124340_1_478397_MIX1243_1729_29	superfamily	223021	5	150	0.000287	43.3885	cl33720	PHA03247 superfamily
	Truan1_653227_gm1_1078_g	superfamily	223021	12	149	0.005487	39.5365	cl33720	PHA03247 superfamily
	Sclbo1_1351_transcript_ESZ98255	superfamily	223021	9	312	0.006973	39.1513	cl33720	PHA03247 superfamily
	Humgr1_5688_Humgr2_002523_RA	superfamily	223021	7	139	0.002308	40.3069	cl33720	PHA03247 superfamily
	ExoJF061F_1_574228_fgenesh1_pm_13_150	superfamily	223021	6	96	0.007345	38.7661	cl33720	PHA03247 superfamily
	XyFL0821_1_108665_CE108664_65662	superfamily	223021	6	108	0.000915	41.8477	cl33720	PHA03247 superfamily
	XylFL0016_361606_CE361605_8416	superfamily	223021	2	105	0.007029	39.1513	cl33720	PHA03247 superfamily
	XyFL0804_2_218388_CE218387_17711	superfamily	165527	6	109	0.007883	38.556	cl29788	PHA03269 superfamily
	Cadsp1_424734_e_gw1_76_23_1	superfamily	177614	67	191	0.001705	40.4212	cl31823	PHA03377 superfamily
	Bombom1_335583_CE335582_4658	superfamily	223065	52	132	0.007901	37.7392	cl33729	PHA03378 superfamily
	NeoP8C63_1_97988_CE97987_10630	superfamily	236669	183	323	2.16E-05	47.0019	cl35903	PRK10263 superfamily
	Delst1_395606_gm1_1540_g	superfamily	236669	135	242	0.001127	41.2239	cl35903	PRK10263 superfamily
	Spoth2_113912_Spoth1_estExt_fgenesh1_pm_C_20389	superfamily	236669	2	96	0.001032	41.6091	cl35903	PRK10263 superfamily
	Corsim1_58666_fgenesh1_pg_4_383	superfamily	236669	2	97	8.73E-05	45.0759	cl35903	PRK10263 superfamily
	Lepor2_497062_e_gw1_29_3_1	superfamily	236669	154	285	0.006505	38.9127	cl35903	PRK10263 superfamily
	Leptod1_303346_e_gw1_69_144_1	superfamily	236669	154	285	0.006505	38.9127	cl35903	PRK10263 superfamily
	Annmor1_456183_gm1_3848_g	superfamily	236669	7	147	0.003892	39.6831	cl35903	PRK10263 superfamily
	Phisu1_1167_transcript_CZR51295	superfamily	236669	190	311	0.005487	39.2979	cl35903	PRK10263 superfamily
	Phiglo1_14671_CE14670_2614	superfamily	236669	4	83	0.002258	40.4535	cl35903	PRK10263 superfamily
	Phisc1_694098_fgenesh1_pm_3_428	superfamily	236669	163	266	0.006783	38.9127	cl35903	PRK10263 superfamily
	Macph1_8076_MPH_08247m_01	superfamily	236669	129	224	0.002987	39.6831	cl35903	PRK10263 superfamily
	Macpha1_239582_CE239581_4114	superfamily	236669	129	224	0.002962	39.6831	cl35903	PRK10263 superfamily
	Veral1_7293_VDBG_04492T0	superfamily	236669	1	152	0.00166	40.4535	cl35903	PRK10263 superfamily
	Triaspe1_175715_CE175714_15240	superfamily	236669	56	157	0.00942	38.9127	cl35903	PRK10263 superfamily
	Ilysp1_688718_CE688717_4882	superfamily	236669	65	177	0.000151	43.5351	cl35903	PRK10263 superfamily
	Botci1_15389_BC1T_11858	superfamily	236669	63	203	0.004235	38.9127	cl35903	PRK10263 superfamily
	Ilyeu1_164583_CE164582_12239	superfamily	236669	41	153	0.000131	43.5351	cl35903	PRK10263 superfamily
	Sclsc1_11781_SS1G_04810T0	superfamily	236669	159	294	0.003178	40.0683	cl35903	PRK10263 superfamily
	Helsp1_265149_CE265148_4333	superfamily	236669	134	261	0.000174	43.9203	cl35903	PRK10263 superfamily
	Ilyrob1_339539_e_gw1_2_1828_1	superfamily	236669	234	346	0.00098	41.9943	cl35903	PRK10263 superfamily
	Tricre1_359307_fgenesh1_pm_88_17	superfamily	236669	21	140	0.001327	41.6091	cl35903	PRK10263 superfamily
	Znioc1_1223138_gm1_13786_g	superfamily	236669	201	296	0.003013	40.0683	cl35903	PRK10263 superfamily
	Exool1_141753_PV06_05994T0	superfamily	237865	16	135	0.001222	41.2384	cl36447	PRK14951 superfamily
	Chame1_15830_CE15829_4335	superfamily	184923	37	83	0.002141	40.4335	cl33044	PRK14959 superfamily
	Veral1_7293_VDBG_04492T0	superfamily	227505	87	158	0.000486	42.2725	cl34928	PRP8 superfamily
	Loppic1_106226_CE106225_3135	superfamily	185594	183	330	0.001797	40.8288	cl33180	PTZ00395 superfamily
	XylFL2044_770840_fgenesh1_pm_14_15	superfamily	185594	2	121	0.000589	42.3695	cl33180	PTZ00395 superfamily
	Amore1_29369_fgenesh1_pg_9_17	superfamily	185594	160	313	0.001063	41.5991	cl33180	PTZ00395 superfamily
	Horac1_312459_fgenesh1_pg_9_192	superfamily	185594	5	113	0.00127	41.2139	cl33180	PTZ00395 superfamily
	Psene1_2233944_fgenesh1_pm_11_17	superfamily	227709	64	192	0.0007	41.4151	cl34999	ROM1 superfamily
	Oidneo1_278_rna_gnl_WGS_MCFK_OnM2_107002T	superfamily	227709	205	333	0.000783	42.1855	cl34999	ROM1 superfamily
	Erypi2_2313634_e_gw1_35_62_1	superfamily	227709	192	320	0.00824	38.7187	cl34999	ROM1 superfamily
	Erypu1_4448_transcript_POS88000	superfamily	227709	198	326	0.000559	42.5707	cl34999	ROM1 superfamily
	Aspcej1_44738_CE44737_49	superfamily	435798	162	219	0.000354	39.332	cl29068	SGP superfamily
	Cryan3_374345_CE374344_3122	superfamily	400730	171	223	0.000604	41.9996	cl25487	SWI-SNF_Ssr4 superfamily
	Crymi1_8611_B0A49_04274	superfamily	400730	150	202	0.005148	38.918	cl25487	SWI-SNF_Ssr4 superfamily
	Xylbam139988_1_638787_fgenesh1_pm_49_60	superfamily	425992	91	158	0.002771	39.1702	cl03073	TYA superfamily
	Neora1_565526_e_gw1_3_1060_1	superfamily	425992	274	363	0.000652	41.8666	cl03073	TYA superfamily
Pez-VelC	Trikon1_14646_CE14645_5374	superfamily	427171	58	231	6.65E-06	48.6094	cl38111	Atrophin-1 superfamily
	Tri5649_1_14918_CE14917_10587	superfamily	427171	41	232	9.73E-05	44.7574	cl38111	Atrophin-1 superfamily
	Trigam1_10529_TGAM01_02329T0	superfamily	427171	5	234	1.66E-06	50.5354	cl38111	Atrophin-1 superfamily
	Triatr1_215179_CE215178_19387	superfamily	427171	56	231	4.51E-05	45.913	cl38111	Atrophin-1 superfamily
	Triatro1_258993_CE258992_14165	superfamily	427171	56	231	4.51E-05	45.913	cl38111	Atrophin-1 superfamily
	Triatrov1_178878_CE178877_3097	superfamily	427171	56	231	4.51E-05	45.913	cl38111	Atrophin-1 superfamily
	Tritai1_82852_CE82851_5785	superfamily	427171	41	232	9.73E-05	44.7574	cl38111	Atrophin-1 superfamily
	Triaus1_319816_fgenesh1_pm_11_47	superfamily	427171	15	217	0.000125	44.7574	cl38111	Atrophin-1 superfamily
	Fusps1_10113_FPSE_11893T0	superfamily	427171	4	182	0.001615	40.9054	cl38111	Atrophin-1 superfamily
	Trigha1_468975_fgenesh1_pm_5_128	superfamily	427171	192	432	1.82E-07	54.3874	cl38111	Atrophin-1 superfamily
	Kalbru1_871751_fgenesh1_pm_65_1	superfamily	427171	1	141	4.47E-07	52.0762	cl38111	Atrophin-1 superfamily
	Tubbr1_1_891583_e_gw1_288_18_1	superfamily	427171	15	167	0.000519	42.4462	cl38111	Atrophin-1 superfamily
	Trifer1_67891_CE67890_1573	superfamily	427171	19	226	5.85E-08	55.1578	cl38111	Atrophin-1 superfamily
	Triheli1_7208_CE7207_3904	superfamily	427171	19	233	3.1E-09	59.395	cl38111	Atrophin-1 superfamily
	Triaru1_3934_rna4755	superfamily	427171	7	215	0.002544	40.135	cl38111	Atrophin-1 superfamily
	Triev1_16638_CE16637_3445	superfamily	427171	41	230	6.32E-05	45.5278	cl38111	Atrophin-1 superfamily
	Cylol1_57769_CE57768_2273	superfamily	427171	35	246	2.42E-08	56.6986	cl38111	Atrophin-1 superfamily
	Lepmu1_7241_Lema_T121860_1	superfamily	227270	28	180	0.002866	40.5126	cl34841	COG4934 superfamily
	Aspins1_179956_CE179955_622	superfamily	227430	113	203	0.002882	40.115	cl34901	COG5099 superfamily
	Tubae1_3358_GSTUAT00001721001	superfamily	227430	18	189	0.000414	42.8114	cl34901	COG5099 superfamily
	Trepe1_565784_gm1_8776_g	superfamily	455861	19	168	8.33E-05	44.8164	cl42516	dnaA superfamily
	Hypmon1_279203_gm1_5127_g	superfamily	455861	27	252	0.006264	39.0384	cl42516	dnaA superfamily
	Tristr1_233395_CE233394_6826	superfamily	455861	73	211	0.00163	40.9644	cl42516	dnaA superfamily

Triint1_75035_CE75034_3341	superfamily	455861	101	240	0.000303	43.2756	cl42516	dnaA superfamily
HypCO275_1_11909_fgenesh1_pg_54_35	superfamily	455861	40	240	0.000108	44.4312	cl42516	dnaA superfamily
Hypinv1_688242_gm4_7614_g	superfamily	455861	40	240	0.000125	44.4312	cl42516	dnaA superfamily
Lizem1_36413_gw1_27_338_1	superfamily	455861	21	248	9.81E-06	47.898	cl42516	dnaA superfamily
Apiver1_70619_CE70618_245	superfamily	455861	42	213	5.75E-05	45.5868	cl42516	dnaA superfamily
DaFL1419_1_345842_gm1_2794_g	superfamily	455861	67	248	0.007289	38.6532	cl42516	dnaA superfamily
Colna1_302265_CE302264_5477	superfamily	455861	26	152	4.31E-05	45.972	cl42516	dnaA superfamily
Dalgra1_311392_CE311391_8671	superfamily	455861	67	236	0.000219	43.6608	cl42516	dnaA superfamily
Plecu1_246869_CE246868_25627	superfamily	455861	12	211	2.79E-07	52.9056	cl42516	dnaA superfamily
Aqupat1_699847_gm1_11594_g	superfamily	455861	2	218	2.95E-08	55.9871	cl42516	dnaA superfamily
Phomu1_476075_gm1_9353_g	superfamily	455861	21	224	1.55E-06	50.5944	cl42516	dnaA superfamily
Epini1_337701_e_gw1_17_9_1	superfamily	455861	21	223	0.000781	41.7348	cl42516	dnaA superfamily
Didma1_15877_g1981_t1	superfamily	455861	21	167	1.27E-06	52.5204	cl42516	dnaA superfamily
Didsa1_488267_gm1_7399_g	superfamily	455861	5	160	0.002029	40.5792	cl42516	dnaA superfamily
Macan1_457067_gm1_4121_g	superfamily	455861	21	167	2.21E-05	46.7424	cl42516	dnaA superfamily
Plecucu2_11109_CE11108_738	superfamily	455861	33	198	1.04E-08	57.1427	cl42516	dnaA superfamily
Didex1_6159_gm1_6159_g	superfamily	455861	1	223	4.78E-05	45.5868	cl42516	dnaA superfamily
Altsp012_1_510500_fgenesh1_pg_5_785	superfamily	455861	16	232	1.36E-06	50.5944	cl42516	dnaA superfamily
Altsp017_1_123168_CE123167_7233	superfamily	455861	16	232	1.31E-06	50.5944	cl42516	dnaA superfamily
Altte1_111178_AT2CTG_122_jigsaw_mRNA_141	superfamily	455861	66	245	1.8E-05	47.1276	cl42516	dnaA superfamily
Amnli1_164115_CE164114_2601	superfamily	455861	19	200	6.01E-08	55.2167	cl42516	dnaA superfamily
Ampqui1_471313_estExt_Genewise1_C_3_t20278	superfamily	455861	24	245	0.000112	44.4312	cl42516	dnaA superfamily
Altfr1_234601_AFGCTG_165_jigsaw_mRNA_1	superfamily	455861	66	245	2.18E-05	46.7424	cl42516	dnaA superfamily
Altli1_253221_ATKCTG_335_jigsaw_mRNA_5	superfamily	455861	66	245	2.12E-05	46.7424	cl42516	dnaA superfamily
Altlo1_255229_ALGCTG_20_jigsaw_mRNA_31	superfamily	455861	66	245	3.45E-05	46.3572	cl42516	dnaA superfamily
Altro1_80517_CE80516_5885	superfamily	455861	20	183	4.27E-05	45.972	cl42516	dnaA superfamily
Alalt1_121975_ATNCTG_79_jigsaw_mRNA_90	superfamily	455861	66	245	2.12E-05	46.7424	cl42516	dnaA superfamily
Alalte1_113147_AATCTG_71_jigsaw_mRNA_77	superfamily	455861	66	245	2.12E-05	46.7424	cl42516	dnaA superfamily
Aalte1_122331_AA2CTG_80_jigsaw_mRNA_96	superfamily	455861	66	245	2.12E-05	46.7424	cl42516	dnaA superfamily
Altalt1_355135_e_gw1_5_738_1	superfamily	455861	66	245	2.12E-05	46.7424	cl42516	dnaA superfamily
Altal1_34254_CE34253_6289	superfamily	455861	66	245	2.12E-05	46.7424	cl42516	dnaA superfamily
Altci1_113738_ACTCTG_1024_jigsaw_mRNA_5	superfamily	455861	66	245	2.12E-05	46.7424	cl42516	dnaA superfamily
Amal3063_116065_AMLCTG_1295_jigsaw_mRNA_14	superfamily	455861	67	246	2.04E-05	47.1276	cl42516	dnaA superfamily
Amal3064_119548_AM2CTG_211_jigsaw_mRNA_14	superfamily	455861	66	245	2.12E-05	46.7424	cl42516	dnaA superfamily
Altga1_267021_AGSCTG_1827_jigsaw_mRNA_1	superfamily	455861	66	245	2.18E-05	46.7424	cl42516	dnaA superfamily
Pyrinf1_56837_CE56836_7349	superfamily	455861	20	186	0.000124	44.4312	cl42516	dnaA superfamily
Pyrsp1_284712_CE284711_8520	superfamily	455861	20	186	0.000122	44.4312	cl42516	dnaA superfamily
PlePMI138_1_294216_estExt_Genewise1_C_21_t10035	superfamily	455861	21	145	0.001664	40.9644	cl42516	dnaA superfamily
Plesi1_496476_gm1_258_g	superfamily	455861	1	233	7.27E-05	45.2016	cl42516	dnaA superfamily
PhaPMI808_777953_gm1_12249_g	superfamily	455861	20	245	0.000416	42.8904	cl42516	dnaA superfamily
Denna1_93401_CE93400_2622	superfamily	455861	54	277	0.000107	44.8164	cl42516	dnaA superfamily
Fenfe1_150748_CE150747_5247	superfamily	455861	32	188	4.23E-05	45.972	cl42516	dnaA superfamily
Melpu1_368259_gm1_13282_g	superfamily	455861	15	212	1.27E-05	47.5128	cl42516	dnaA superfamily
Linin1_82629_CE82628_435	superfamily	455861	62	188	1.75E-07	53.6759	cl42516	dnaA superfamily
Masph1_603996_gm1_3561_g	superfamily	455861	19	177	1.95E-05	47.1276	cl42516	dnaA superfamily
Lepmi1_490062_gm1_9883_g	superfamily	455861	1	224	0.003036	39.8088	cl42516	dnaA superfamily
Ophdi1_303097_e_gw1_22_348_1	superfamily	455861	24	188	0.000376	42.8904	cl42516	dnaA superfamily
Boeex1_414344_gm1_7117_g	superfamily	455861	21	225	9.39E-05	44.8164	cl42516	dnaA superfamily
Perma1_514955_e_gw1_10_385_1	superfamily	455861	26	149	1.57E-07	53.2907	cl42516	dnaA superfamily
Phapo1_386153_CE386152_3326	superfamily	455861	24	247	0.001135	41.3496	cl42516	dnaA superfamily
Stasp1_43754_CE43753_2824	superfamily	455861	24	247	0.000712	42.12	cl42516	dnaA superfamily
Daral1_846723_fgenesh1_pg_8_92	superfamily	455861	30	236	0.000952	41.7348	cl42516	dnaA superfamily
PseVKM4246_1_5087_transcript_KFY06812	specific	403112	409	606	5.04E-28	112.173	pfam11807	DUF3328
Verlo1_7189_transcript_CRK21276	specific	427294	757	986	2.32E-92	297.441	pfam03441	FAD_binding_7
Pse1611_1_87933_CE87932_36050	superfamily	235904	47	125	0.002297	40.3347	cl35529	flhF superfamily
Stach1_6950_transcript_KFA75101	superfamily	235904	61	190	0.001187	41.1051	cl35529	flhF superfamily
Aspcru1_117840_CE117839_1508	superfamily	406402	87	225	6E-05	44.9205	cl25765	G_path_suppress superfamily
Clasph1_3210_CSPH_06797_R0	superfamily	282904	11	211	2.6E-06	51.4556	cl37540	Herpes_BLLF1 superfamily
Xylint1_531850_fgenesh1_pm_55_26	superfamily	282904	26	192	0.001046	41.4404	cl37540	Herpes_BLLF1 superfamily
Tubmes1_3274166_e_gw1_62_18_1	superfamily	430088	22	244	0.000113	44.4357	cl25816	KAR9 superfamily
Thest1_6454_NODE_644_length_767692_cov_86_g3751_t1	superfamily	425360	271	351	0.000804	42.374	cl41729	KLF1_2_4_N superfamily
Fustri1_583163_fgenesh1_pm_2_734	superfamily	425360	18	91	0.007258	38.101	cl41729	KLF1_2_4_N superfamily
Gyrinf1_909377_gm1_2830_g	superfamily	312941	44	131	0.006282	38.836	cl26621	Med15 superfamily
Dalcal1_451435_fgenesh1_pm_10_55	superfamily	130689	25	153	0.000478	42.4866	cl31127	PABP-1234 superfamily
Tricer1_163848_CE163847_24685	superfamily	130689	50	209	0.002463	40.1754	cl31127	PABP-1234 superfamily
Torhe1_5577_VHEMI05184_1	superfamily	130689	97	218	0.00513	39.0198	cl31127	PABP-1234 superfamily
Tricre1_59734_CE59733_3478	superfamily	130689	50	183	0.002785	40.1754	cl31127	PABP-1234 superfamily
Psemus1_11545_transcript_KXT14792	superfamily	401645	6	246	5.14E-05	45.7972	cl37801	PAT1 superfamily
Geocar1_718935_fgenesh1_pm_32_57	superfamily	401645	3	136	0.000472	42.7156	cl37801	PAT1 superfamily
Cormi1_2376_CCM_02384m_01	superfamily	401645	5	156	0.000121	43.486	cl37801	PAT1 superfamily

CodFL1790_1_97065_CE97064_7752	superfamily	401645	40	279	0.000284	43.486	cl37801	PAT1 superfamily
Fusve2_16812_FVEG_05214T0	superfamily	401645	1	172	3.13E-06	49.6492	cl37801	PAT1 superfamily
Neo1551_1_372221_CE372220_3621	superfamily	401645	49	150	0.001503	41.1748	cl37801	PAT1 superfamily
Trichl1_184709_CE184708_7657	superfamily	401645	55	195	0.004549	39.634	cl37801	PAT1 superfamily
Neobo1_508674_e_gw1_11_141_1	superfamily	330753	56	165	0.000729	41.9972	cl25932	Peptidase_S21 superfamily
Micbo1_170925_fgenesh1_pg_1_796	superfamily	223021	7	195	8.36E-05	45.3145	cl33720	PHA03247 superfamily
Maseb1_413359_e_gw1_42_55_1	superfamily	223021	4	129	1.27E-05	48.0109	cl33720	PHA03247 superfamily
Lopma1_677411_fgenesh1_pg_40_43	superfamily	223021	47	243	0.000677	42.6181	cl33720	PHA03247 superfamily
Lenfl1_317782_e_gw1_46_187_1	superfamily	223021	24	227	2.02E-06	50.3221	cl33720	PHA03247 superfamily
Hyppol1_518568_fgenesh1_pm_6_268	superfamily	223021	4	231	0.000149	44.5441	cl33720	PHA03247 superfamily
Jahaq1_94727_CE94726_4172	superfamily	223021	21	276	6.19E-06	49.1665	cl33720	PHA03247 superfamily
Corsim1_65308_fgenesh1_pm_1_172	superfamily	223021	51	223	1.32E-06	51.4777	cl33720	PHA03247 superfamily
AnFL0455_111911_CE111910_5870	superfamily	223021	9	236	2.08E-07	53.7889	cl33720	PHA03247 superfamily
Anntru1_311532_CE311531_13896	superfamily	223021	9	201	9.65E-06	48.3961	cl33720	PHA03247 superfamily
Pse1611_1_87933_CE87932_36050	superfamily	223021	15	242	0.004146	39.9217	cl33720	PHA03247 superfamily
Sacpr1_47109_e_gw1_22_187_1	superfamily	223021	8	145	7.51E-07	51.4777	cl33720	PHA03247 superfamily
Meltu1_652512_estExt_fgenesh1_pm_C_100296	superfamily	223021	21	223	0.000722	42.2329	cl33720	PHA03247 superfamily
HyFL0890_1_293355_gm1_2204_g	superfamily	223021	33	248	0.000448	43.0033	cl33720	PHA03247 superfamily
Ophun1_6569_transcript_PFH61685	superfamily	223021	120	252	0.00333	39.9217	cl33720	PHA03247 superfamily
Chafu1_182160_CE182159_2477	superfamily	223021	45	240	1.61E-05	48.0109	cl33720	PHA03247 superfamily
Cenge3_540295_e_gw1_5_1528_1	superfamily	223021	6	154	9.61E-08	54.5593	cl33720	PHA03247 superfamily
Dalver1_24769_CE24768_1376	superfamily	223021	7	251	0.001653	41.0773	cl33720	PHA03247 superfamily
Cha1176438_53013_gm1_5807_g	superfamily	223021	45	240	1.61E-05	48.0109	cl33720	PHA03247 superfamily
Annmin1_341649_fgenesh1_pm_34_	superfamily	223021	2	232	0.006351	39.1513	cl33720	PHA03247 superfamily
Cerbe1_10364_transcript_PPJ54732	superfamily	223021	2	181	1.63E-05	47.6257	cl33720	PHA03247 superfamily
XyFL0641_1_768576_fgenesh1_pm_5_65	superfamily	223021	11	228	1.26E-05	48.0109	cl33720	PHA03247 superfamily
Clamic1_59444_CE59443_1829	superfamily	223021	46	287	0.000139	44.9293	cl33720	PHA03247 superfamily
Phiglo1_111995_CE111994_469	superfamily	223021	42	293	0.00935	38.7661	cl33720	PHA03247 superfamily
TrireMAT11_1_8311_TRC1_007685_T1	superfamily	223021	45	226	3.03E-05	46.8553	cl33720	PHA03247 superfamily
TrireMAT12_1_8306_TRC2_007634_T1	superfamily	223021	5	186	2.98E-05	46.4701	cl33720	PHA03247 superfamily
Hyprub1_447125_gm1_4737_g	superfamily	223021	34	235	0.001746	41.0773	cl33720	PHA03247 superfamily
Chafi1_460122_MIX12583_1875_69	superfamily	223021	47	231	0.000514	43.0033	cl33720	PHA03247 superfamily
Zasce1_63324_fgenesh1_pm_4_90	superfamily	223021	7	209	1.32E-06	51.0925	cl33720	PHA03247 superfamily
Chagl1_600993_gm1_4196_g	superfamily	223021	47	238	5.12E-06	49.5517	cl33720	PHA03247 superfamily
Horwer1_14319_HWER_06770_R0	superfamily	223021	48	227	0.007052	39.1513	cl33720	PHA03247 superfamily
HyFL1857_1_185055_gm1_1114_g	superfamily	223021	37	202	0.000121	44.9293	cl33720	PHA03247 superfamily
Seppo1_103637_e_gw1_42_7_1	superfamily	223021	3	217	0.001472	41.4625	cl33720	PHA03247 superfamily
Thihy1_543052_MIX14763_493_91	superfamily	223021	30	224	8.62E-06	48.7813	cl33720	PHA03247 superfamily
Zymbr1_9476_Tl39_contig604g00003T0	superfamily	223021	42	229	3.12E-05	46.8553	cl33720	PHA03247 superfamily
Chame1_427882_MIX8185_3816_97	superfamily	223021	54	231	0.002015	41.0773	cl33720	PHA03247 superfamily
Grocl1_7162_CMQ_7778m_01	superfamily	223021	14	223	0.004289	39.9217	cl33720	PHA03247 superfamily
NeFL0916_1_315058_CE315057_4411	superfamily	223021	37	294	0.00258	40.3069	cl33720	PHA03247 superfamily
Trigu1_432746_CE432745_22045	superfamily	223021	15	185	0.001286	41.0773	cl33720	PHA03247 superfamily
Durrog2_14479_CE14478_7664	superfamily	223021	2	245	0.000764	42.2329	cl33720	PHA03247 superfamily
Melsp1_542306_CE318833_832	superfamily	223021	31	195	0.005094	39.5365	cl33720	PHA03247 superfamily
Thiap1_446666_CE446665_1818	superfamily	223021	30	232	0.003292	40.3069	cl33720	PHA03247 superfamily
Helsp1_440697_gm4_8125_g	superfamily	223021	2	195	0.000331	43.3885	cl33720	PHA03247 superfamily
Myrdu1_289103_gm1_166_g	superfamily	223021	5	247	4.52E-06	49.5517	cl33720	PHA03247 superfamily
TerNC1134_1_830775_fgenesh1_pm_9_198	superfamily	223021	6	197	0.001573	41.0773	cl33720	PHA03247 superfamily
Madmy1_7026_transcript_KXX76444	superfamily	223021	65	278	5.69E-07	52.6333	cl33720	PHA03247 superfamily
Leptod1_168748_CE168747_709	superfamily	223021	25	233	0.000889	41.8477	cl33720	PHA03247 superfamily
Zymar1_770827_Za111_scaffold_342_0002	superfamily	223021	3	219	6.78E-05	45.6997	cl33720	PHA03247 superfamily
Zymps1_797210_Zp221_scaffold_155_00048	superfamily	223021	57	228	7.38E-07	51.8629	cl33720	PHA03247 superfamily
Clafu1_195193_scf7180000130809_G10000	superfamily	223021	7	297	1E-05	48.3961	cl33720	PHA03247 superfamily
Amore1_15389_gm1_1058_g	superfamily	223021	16	227	0.003388	39.9217	cl33720	PHA03247 superfamily
Atrpi1_38886_CE38885_6051	superfamily	223021	3	194	1.59E-05	47.2405	cl33720	PHA03247 superfamily
Mycgr3_103692_estExt_fgenesh1_kg_C_chr_30193	superfamily	223021	57	230	1.14E-05	48.0109	cl33720	PHA03247 superfamily
Rac5018_1_16821_transcript_OQO32207	superfamily	223021	7	202	5.47E-05	46.0849	cl33720	PHA03247 superfamily
Dotse1_71382_estExt_fgenesh1_kg_C_4_t20144	superfamily	223021	28	313	4.56E-06	49.5517	cl33720	PHA03247 superfamily
NeoP8C63_1_439810_CE439809_9043	superfamily	223021	50	325	0.000526	43.0033	cl33720	PHA03247 superfamily
Lacsu1_454676_gm1_2442_g	superfamily	223021	63	191	0.000993	41.4625	cl33720	PHA03247 superfamily
Frisi1_12689_B0A55_11371	superfamily	223021	6	194	0.005761	39.1513	cl33720	PHA03247 superfamily
Xanpa2_907647_CE907646_35285	superfamily	223021	27	213	0.005686	39.5365	cl33720	PHA03247 superfamily
Pezci1_127579_CE127578_9570	superfamily	223021	36	244	5.53E-05	45.6997	cl33720	PHA03247 superfamily
Trire_Chr_116273_TrE0945C	superfamily	223021	275	532	4.42E-05	47.2405	cl33720	PHA03247 superfamily
TrireRUTC30_1_67935_e_gw1_1_2020_1	superfamily	223021	275	532	4.42E-05	47.2405	cl33720	PHA03247 superfamily
Trire2_102737_fgenesh5_pg_C_scaffold_1000370	superfamily	223021	275	532	4.42E-05	47.2405	cl33720	PHA03247 superfamily
Exool1_138297_PV06_03175T0	superfamily	223021	42	228	0.001031	41.8477	cl33720	PHA03247 superfamily
Podcom1_2912_CE2911_1256	superfamily	223021	75	238	0.006952	39.1513	cl33720	PHA03247 superfamily
Asphir1_165862_gm1_5490_g	superfamily	223021	34	392	0.008751	38.7661	cl33720	PHA03247 superfamily

Colfi1_275694_mRNA514	superfamily	223021	9	205	0.007862	39.1513	cl33720	PHA03247 superfamily
Colsi1_972199_mrna_319	superfamily	223021	9	205	0.006933	39.1513	cl33720	PHA03247 superfamily
Micmi1_36440_CE36439_7282	superfamily	223021	4	209	3.06E-05	46.8553	cl33720	PHA03247 superfamily
Morcra1_214498_CE214497_3469	superfamily	223021	5	230	0.004838	39.5365	cl33720	PHA03247 superfamily
Colac2_716140_fgenesh1_pm_10_19	superfamily	223021	9	200	0.00289	40.3069	cl33720	PHA03247 superfamily
Mychet1_189815_e_gw1_1_308_1	superfamily	223021	48	241	0.001868	41.4625	cl33720	PHA03247 superfamily
Myche1_215200_CE215199_731	superfamily	223021	48	241	0.001868	41.4625	cl33720	PHA03247 superfamily
Ceraff1_167243_CE167242_2038	superfamily	223021	18	288	7.94E-06	48.7813	cl33720	PHA03247 superfamily
Colgr1_9659_GLRG_02266T0	superfamily	223021	4	228	2.18E-06	50.7073	cl33720	PHA03247 superfamily
NsAZ0576_1_337305_fgenesh1_pm_152_2	superfamily	223021	80	250	0.000536	42.6181	cl33720	PHA03247 superfamily
Disven1_83874_CE83873_9512	superfamily	223021	3	146	0.000896	42.2329	cl33720	PHA03247 superfamily
Xylgra1_138919_CE138918_2302	superfamily	223021	75	241	0.006069	39.1513	cl33720	PHA03247 superfamily
Leuca1_140736_CE140735_6125	superfamily	223021	3	221	4.26E-05	46.0849	cl33720	PHA03247 superfamily
Umbpus1_103340_LPUS_09921T0	superfamily	223021	6	172	0.00011	44.5441	cl33720	PHA03247 superfamily
Echmac1_70536_CE70535_2200	superfamily	223021	72	226	0.001392	41.4625	cl33720	PHA03247 superfamily
NeFL0031_2_555829_gm1_1928_g	superfamily	223021	14	242	0.000423	43.0033	cl33720	PHA03247 superfamily
Tubbor1_1098298_fgenesh1_pm_4_17	superfamily	223021	15	261	0.002087	40.6921	cl33720	PHA03247 superfamily
Nemabo1_275432_fgenesh1_pm_16_27	superfamily	223021	14	241	0.00914	38.7661	cl33720	PHA03247 superfamily
Thite2_2145927_fgenesh1_pm_chromosome_4_228	superfamily	223021	29	221	0.000203	44.1589	cl33720	PHA03247 superfamily
Xyllon1_225773_CE225772_6999	superfamily	223021	9	246	0.000771	42.2329	cl33720	PHA03247 superfamily
Purli1_7337_transcript_OAQ89112	superfamily	223021	61	216	0.000972	41.8477	cl33720	PHA03247 superfamily
Tolca1_1425_transcript_PNY25538	superfamily	223021	80	242	0.003332	40.3069	cl33720	PHA03247 superfamily
Xylscr1_353604_CE353603_9632	superfamily	223021	4	245	0.00522	39.5365	cl33720	PHA03247 superfamily
Tubma1_314402_fgenesh1_kg_21_264_Tmag110907_c6416	superfamily	223021	3	221	2.11E-05	46.8553	cl33720	PHA03247 superfamily
Tubin1_1_478062_e_gw1_9_135_1	superfamily	223021	21	180	0.000295	43.3885	cl33720	PHA03247 superfamily
Erebi1_214840_CE214839_1284	superfamily	223021	8	229	0.000798	42.2329	cl33720	PHA03247 superfamily
Neodi1_7436_AK830_g3061T0	superfamily	223021	13	222	9.19E-06	48.3961	cl33720	PHA03247 superfamily
Bulin1_802888_gm1_3062_g	superfamily	223021	11	208	0.000503	42.6181	cl33720	PHA03247 superfamily
Sposc1_9834_transcript_KJR80798	superfamily	223021	87	303	1.68E-05	48.0109	cl33720	PHA03247 superfamily
Podcur1_26789_CE26788_985	superfamily	223021	23	290	0.000281	43.7737	cl33720	PHA03247 superfamily
Neora1_44308_CE44307_9474	superfamily	223021	1	200	8.52E-06	48.3961	cl33720	PHA03247 superfamily
OphPMI507_1_419987_estExt_fgenesh1_pm_C_2_t10151	superfamily	223021	97	319	4.22E-05	46.4701	cl33720	PHA03247 superfamily
Annsty1_412332_gm1_38_g	superfamily	223021	2	231	0.00012	44.9293	cl33720	PHA03247 superfamily
Annnit1_27170_CE27169_3519	superfamily	223021	6	235	0.000557	42.6181	cl33720	PHA03247 superfamily
Pyrtt1_7407_PTTG_15273_1	superfamily	223021	20	262	0.000693	42.6181	cl33720	PHA03247 superfamily
Humgr1_3818_Humgr2_001553_RA	superfamily	223021	42	321	0.000832	42.6181	cl33720	PHA03247 superfamily
Annmae1_110697_CE110696_2560	superfamily	223021	33	229	0.000134	44.5441	cl33720	PHA03247 superfamily
Bimnz1_569189_fgenesh1_pg_32_86	superfamily	223021	8	221	6.84E-05	45.3145	cl33720	PHA03247 superfamily
Triiv1_186121_CE186120_3612	superfamily	223021	19	233	6.63E-07	52.2481	cl33720	PHA03247 superfamily
Trila1_240648_CE240647_6326	superfamily	223021	28	210	5.15E-06	49.1665	cl33720	PHA03247 superfamily
Triple1_116322_g9618_t1	superfamily	223021	19	240	0.00022	44.1589	cl33720	PHA03247 superfamily
Triflo1_169804_CE169803_2226	superfamily	223021	13	205	3.67E-07	52.6333	cl33720	PHA03247 superfamily
TriharM10_1_267760_CE267759_2450	superfamily	223021	4	227	1.16E-05	48.0109	cl33720	PHA03247 superfamily
TriharT22_1_18404_CE18403_3495	superfamily	223021	4	227	8.63E-06	48.3961	cl33720	PHA03247 superfamily
Tricae1_34120_CE34119_2522	superfamily	223021	28	205	3.09E-06	49.9369	cl33720	PHA03247 superfamily
Tricam1_139660_CE139659_2913	superfamily	223021	18	227	2.61E-05	46.8553	cl33720	PHA03247 superfamily
Trice1_284663_CE284662_6734	superfamily	223021	12	230	2.15E-05	47.2405	cl33720	PHA03247 superfamily
Trien1_65374_CE65373_8609	superfamily	223021	20	228	2.31E-05	47.2405	cl33720	PHA03247 superfamily
Trialn1_83150_CE83149_6515	superfamily	223021	19	230	0.000109	44.9293	cl33720	PHA03247 superfamily
Tripleu1_474714_MIX7386_3386_45	superfamily	223021	19	229	0.000431	43.0033	cl33720	PHA03247 superfamily
Triasper1_169167_MIX9084_582_99	superfamily	223021	32	232	0.000791	42.2329	cl33720	PHA03247 superfamily
Triafa1_316405_fgenesh1_pm_22_101	superfamily	223021	18	227	2.61E-05	46.8553	cl33720	PHA03247 superfamily
Triaspe1_362366_CE362365_5158	superfamily	223021	32	232	0.000791	42.2329	cl33720	PHA03247 superfamily
XylFL0662B_627247_fgenesh1_pm_33_10	superfamily	223021	4	266	0.00083	42.2329	cl33720	PHA03247 superfamily
Trivel1_315310_CE315309_12223	superfamily	223021	21	231	1.46E-06	51.0925	cl33720	PHA03247 superfamily
Triveli1_266865_CE266864_6384	superfamily	223021	21	231	1.61E-06	50.7073	cl33720	PHA03247 superfamily
Triros1_425884_CE425883_9031	superfamily	223021	19	233	6.63E-07	52.2481	cl33720	PHA03247 superfamily
Trsp14807_237073_CE237072_3983	superfamily	223021	18	227	2.61E-05	46.8553	cl33720	PHA03247 superfamily
Tri5640_1_232218_CE232217_4839	superfamily	223021	20	231	0.000321	43.3885	cl33720	PHA03247 superfamily
Trich5757_1_253265_CE253264_2606	superfamily	223021	18	227	2.61E-05	46.8553	cl33720	PHA03247 superfamily
TriTUC5505_1_199247_CE199246_8885	superfamily	223021	18	227	2.48E-05	46.8553	cl33720	PHA03247 superfamily
Trispi1_2_27020_fgenesh1_pm_6_206	superfamily	223021	56	212	0.005934	39.1513	cl33720	PHA03247 superfamily
Trist1_271402_CE271401_21161	superfamily	223021	41	227	4.74E-06	49.1665	cl33720	PHA03247 superfamily
Dotsy1_389026_gm1_8859_g	superfamily	223021	24	234	0.000288	43.3885	cl33720	PHA03247 superfamily
Parsp1_1147235_estExt_Genewise1Plus_C_6_t10497	superfamily	223021	8	156	7.67E-06	48.0109	cl33720	PHA03247 superfamily
Achstr1_304862_CE304861_2266	superfamily	223021	48	274	0.001722	41.4625	cl33720	PHA03247 superfamily
Alikh1_674291_fgenesh1_pg_54_8	superfamily	223021	69	195	6.16E-08	55.3297	cl33720	PHA03247 superfamily
Hypfra2_517827_gm1_1503_g	superfamily	223021	4	221	0.004531	39.5365	cl33720	PHA03247 superfamily
Gaegr1_2046_GGTG_02013T0	superfamily	223021	113	523	6.7E-05	46.4701	cl33720	PHA03247 superfamily
Acrst1_1316475_fgenesh1_pg_1_698	superfamily	223021	6	228	5.69E-05	45.6997	cl33720	PHA03247 superfamily
Annmor1_369100_CE369099_3753	superfamily	223021	6	236	3.09E-06	49.9369	cl33720	PHA03247 superfamily
Sarstr1_220873_fgenesh1_pg_2_77	superfamily	223021	6	228	5.69E-05	45.6997	cl33720	PHA03247 superfamily
Whamic1_575337_gm1_470_g	superfamily	223021	30	280	0.001002	41.8477	cl33720	PHA03247 superfamily
ConPMI546_459205_CE459204_1249	superfamily	223021	84	261	0.000295	43.3885	cl33720	PHA03247 superfamily
Colgl1_7540_transcript_EQB49187	superfamily	223021	6	247	5.58E-07	52.2481	cl33720	PHA03247 superfamily

HyFL0543_1_13076_CE13075_10139	superfamily	223021	18	252	0.000393	43.0033	cl33720	PHA03247 superfamily
Gloci1_1198112_CE1138344_3496	superfamily	223021	32	227	7.41E-06	48.3961	cl33720	PHA03247 superfamily
Ant016_1_642530_gm1_6831_g	superfamily	223021	118	451	5.52E-07	53.4037	cl33720	PHA03247 superfamily
PseVKM4520_1_2473_transcript_KFZ16882	superfamily	223021	7	310	0.009503	38.7661	cl33720	PHA03247 superfamily
Triasp1_466260_gm1_5664_g	superfamily	223021	32	232	0.000832	42.2329	cl33720	PHA03247 superfamily
Wesor1_362405_gw1_6_868_1	superfamily	223021	28	265	0.000258	43.7737	cl33720	PHA03247 superfamily
Diadis1_293164_CE293163_7746	superfamily	223021	9	228	1.38E-06	51.0925	cl33720	PHA03247 superfamily
Conap1_99568_W97_07635T0	superfamily	223021	6	209	0.003382	39.9217	cl33720	PHA03247 superfamily
HypCl4A_1_99088_CE21875_4379	superfamily	223021	6	242	0.0034	39.9217	cl33720	PHA03247 superfamily
Macpha1_695309_fgenesh1_pg_4_75	superfamily	223021	2	152	0.000436	43.0033	cl33720	PHA03247 superfamily
Macph1_11642_MPH_11893m_01	superfamily	223021	2	150	0.000516	43.0033	cl33720	PHA03247 superfamily
ChetPR9b_2_371283_estExt_Genemark1_C_12_t20003	superfamily	223021	2	231	0.000338	43.3885	cl33720	PHA03247 superfamily
ChetPR9b_4_376936_MIX11120_1065_98	superfamily	223021	26	255	0.000342	43.3885	cl33720	PHA03247 superfamily
Cocmi1_5675_gm1_5675_g	superfamily	223021	2	229	2.48E-08	56.4853	cl33720	PHA03247 superfamily
Cocsa1_119375_estExt_Genewise1_C_8_t30427	superfamily	223021	26	253	1.57E-08	57.2557	cl33720	PHA03247 superfamily
Clael1_374849_estExt_Genewise1_C_350060	superfamily	223021	23	252	0.000727	42.2329	cl33720	PHA03247 superfamily
Cocca1_10111_gm1_10111_g	superfamily	223021	2	229	8.73E-05	45.3145	cl33720	PHA03247 superfamily
CocheC4_1_151118_e_gw1_33_40_1	superfamily	223021	2	231	0.000338	43.3885	cl33720	PHA03247 superfamily
CocheC4_3_151118_CocheC4_1_e_gw1_33_40_1	superfamily	223021	2	231	0.000338	43.3885	cl33720	PHA03247 superfamily
CocheC4_6_391188_MIX15653_1065_98	superfamily	223021	26	255	0.000342	43.3885	cl33720	PHA03247 superfamily
ChetHAW225_1_457027_estExt_Genemark1_C_120436	superfamily	223021	2	231	0.000338	43.3885	cl33720	PHA03247 superfamily
ChetHAW225_4_412140_MIX16495_1065_98	superfamily	223021	26	255	0.000342	43.3885	cl33720	PHA03247 superfamily
ChetHm540_1_642_g5359	superfamily	223021	26	255	0.000136	44.5441	cl33720	PHA03247 superfamily
ChetPR1x412_2_386485_estExt_Genemark1_C_9_t10054	superfamily	223021	2	231	0.000338	43.3885	cl33720	PHA03247 superfamily
Bismed1_648168_fgenesh1_pm_8_5	superfamily	223021	11	229	9.42E-05	45.3145	cl33720	PHA03247 superfamily
Biscog1_584209_fgenesh1_pm_29_28	superfamily	223021	4	214	0.000545	42.6181	cl33720	PHA03247 superfamily
Spobr1_14950_transcript_KIH88305	superfamily	223021	89	305	6.7E-06	49.1665	cl33720	PHA03247 superfamily
Totfu1_369620_CE369619_962	superfamily	223021	3	249	1.27E-07	54.5593	cl33720	PHA03247 superfamily
Triei1_161811_CE161810_9224	superfamily	223021	53	213	0.001958	41.0773	cl33720	PHA03247 superfamily
Settu3_1467594_estExt_Genemark1_C_5_t10053	superfamily	223021	24	226	1.9E-06	50.7073	cl33720	PHA03247 superfamily
Settur3_547525_gm1_4691_g	superfamily	223021	24	226	1.9E-06	50.7073	cl33720	PHA03247 superfamily
Venpi1_218568_VP_s81_g8664_t1	superfamily	223021	31	239	0.003109	40.3069	cl33720	PHA03247 superfamily
VenspP74_1_553684_CE553683_3494	superfamily	223021	13	253	4.44E-05	46.4701	cl33720	PHA03247 superfamily
Cryan3_646578_CE646577_4055	superfamily	223021	4	197	0.000629	42.6181	cl33720	PHA03247 superfamily
Decga1_78859_CE78858_4609	superfamily	223021	47	204	0.000593	42.6181	cl33720	PHA03247 superfamily
Cryan3_946493_fgenesh1_pg_2_95	superfamily	223021	4	197	0.001419	41.4625	cl33720	PHA03247 superfamily
ChetHm338_1_421297_estExt_Genemark1_C_130037	superfamily	223021	2	231	0.000338	43.3885	cl33720	PHA03247 superfamily
Cocvi1_31414_gm1_10018_g	superfamily	223021	2	229	7.61E-05	45.3145	cl33720	PHA03247 superfamily
ChetHm338_1_423430_estExt_Genemark1_C_590014	superfamily	223021	2	231	0.000338	43.3885	cl33720	PHA03247 superfamily
Lopmy1_456931_e_gw1_6_1066_1	superfamily	223021	16	190	2.43E-06	50.3221	cl33720	PHA03247 superfamily
Bysci1_200971_CE200970_3130	superfamily	223021	4	232	1.09E-05	48.0109	cl33720	PHA03247 superfamily
Psehy1_496118_gm1_849_g	superfamily	223021	1	208	1.68E-05	47.6257	cl33720	PHA03247 superfamily
Pleav1_82440_fgenesh1_pm_11_116	superfamily	223021	41	210	0.001004	41.8477	cl33720	PHA03247 superfamily
Trikoni1_102763_CE102762_19086	superfamily	223021	56	226	5.16E-05	46.0849	cl33720	PHA03247 superfamily
Trigil1_139312_CE139311_12060	superfamily	223021	54	227	2.92E-05	46.8553	cl33720	PHA03247 superfamily
Trilo3_1164775_CE164775_13147	superfamily	223021	108	277	8.77E-06	48.7813	cl33720	PHA03247 superfamily
Trior1_300172_CE300171_6531	superfamily	223021	108	277	8.77E-06	48.7813	cl33720	PHA03247 superfamily
Trici4_1116812_e_gw1_5_319_1	superfamily	223021	56	226	4.96E-07	52.6333	cl33720	PHA03247 superfamily
Trilon1_477812_e_gw1_288_4_1	superfamily	223021	57	226	8.59E-06	48.3961	cl33720	PHA03247 superfamily
Tripar1_5584_rna1595	superfamily	223021	57	230	2.2E-05	47.2405	cl33720	PHA03247 superfamily
Trigra1_426073_gm1_1392_g	superfamily	223021	94	273	2.94E-07	53.4037	cl33720	PHA03247 superfamily
Stobe1_71457_gm1_1608_g	superfamily	223021	2	248	3.83E-06	49.9369	cl33720	PHA03247 superfamily
Triaet1_10205_CE10204_6651	superfamily	223021	57	226	2.6E-06	50.3221	cl33720	PHA03247 superfamily
Venin1_23636_atg11275_t1	superfamily	223021	34	289	7.65E-05	45.6997	cl33720	PHA03247 superfamily
Trisin1_279331_CE279330_2857	superfamily	223021	56	227	2.04E-05	47.2405	cl33720	PHA03247 superfamily
Daralp1_363826_CE363825_19304	superfamily	223021	41	269	0.00014	44.5441	cl33720	PHA03247 superfamily
FvanT343_1_375750_fgenesh1_pm_6_131	superfamily	223021	8	203	0.000105	44.9293	cl33720	PHA03247 superfamily
EurotioJF033F_1_670307_MIX82_2103_29	superfamily	165527	3	172	0.000216	43.5636	cl29788	PHA03269 superfamily
EurotioJF034F_1_423868_fgenesh1_pg_16_120	superfamily	165527	3	172	0.000216	43.5636	cl29788	PHA03269 superfamily
Crypto1_517308_gm1_3612_g	superfamily	223039	15	216	0.000503	42.4657	cl33723	PHA03307 superfamily
XylFL0016_258239_CE258238_2631	superfamily	223039	53	219	0.004448	39.7693	cl33723	PHA03307 superfamily
Rosne1_3830_transcript_GAP89218	superfamily	223039	37	240	0.00097	41.6953	cl33723	PHA03307 superfamily
Xylbam139988_1_160222_CE160221_8509	superfamily	223039	18	245	4.23E-05	46.3177	cl33723	PHA03307 superfamily
Ophpic1_487_rna486	superfamily	223039	63	245	0.002995	40.1545	cl33723	PHA03307 superfamily
Altso1_121502_ASLCTG_729_jigsaw_mRNA_21	superfamily	223039	48	249	0.000713	42.0805	cl33723	PHA03307 superfamily
Alttom1_114441_ATMCTG_118_jigsaw_mRNA_1	superfamily	223039	48	249	1.9E-05	47.4733	cl33723	PHA03307 superfamily

Altca1_114494_ACSCTG_929_jigsaw_mRNA_5	superfamily	223039	49	250	0.000115	44.7769	cl33723	PHA03307 superfamily
Altcar1_119785_ACMCTG_914_jigsaw_mRNA_1	superfamily	223039	48	249	0.000152	44.3917	cl33723	PHA03307 superfamily
Altma1_118665_AMRCTG_755_jigsaw_mRNA_1	superfamily	223039	48	249	0.00011	44.7769	cl33723	PHA03307 superfamily
Altda1_114626_ADCCTG_407_jigsaw_mRNA_5	superfamily	223039	48	249	0.000193	44.0065	cl33723	PHA03307 superfamily
Zoprh1_113072_CE113071_5868	superfamily	223039	42	246	0.000143	44.3917	cl33723	PHA03307 superfamily
Lopnu1_506561_e_gw1_47_193_1	superfamily	223065	48	251	0.000592	42.3616	cl33729	PHA03378 superfamily
Bisma1_419188_fgenesh1_pm_47_3	superfamily	223065	7	145	2.92E-05	46.5988	cl33729	PHA03378 superfamily
HyNC1633_2_628896_fgenesh1_pm_6_3	superfamily	223065	9	271	7.71E-05	45.058	cl33729	PHA03378 superfamily
Porpun1_277924_CE277923_7712	superfamily	223065	105	253	0.006126	39.28	cl33729	PHA03378 superfamily
Tolinf1_197073_TINF07095_mRNA_1	superfamily	223065	200	415	2.41E-05	47.3692	cl33729	PHA03378 superfamily
Cerzm1_29152_gw1_5_946_1	superfamily	223065	1	181	0.000674	42.3616	cl33729	PHA03378 superfamily
DallocAZ0526_2_221055_CE221054_16448	superfamily	223065	4	236	0.001372	41.206	cl33729	PHA03378 superfamily
Coclu2_56229_fgenesh1_pm_26_34	superfamily	223065	27	265	0.000275	43.5172	cl33729	PHA03378 superfamily
Acrvag1_525712_CE525711_1567	superfamily	223065	63	254	5.87E-06	48.91	cl33729	PHA03378 superfamily
Verlo1_7189_transcript_CRK21276	superfamily	223492	444	987	3.39E-70	243.768	cl43137	PhrB superfamily
Capcor1_350_A1O1_00352T0	superfamily	215130	7	87	0.000222	43.5392	cl33436	PLN02217 superfamily
Exode1_6083_HMPREF1120_05878T0	superfamily	215130	50	117	0.000745	41.9984	cl33436	PLN02217 superfamily
Asptran1_153856_gm1_8692_g	superfamily	215130	72	163	0.00042	43.154	cl33436	PLN02217 superfamily
Aspchris1_491872_gm1_4862_g	superfamily	215130	72	153	0.00375	39.6872	cl33436	PLN02217 superfamily
Clasph1_3210_CSPH_06797_R0	superfamily	215186	429	972	0	779.593	cl33465	PLN02327 superfamily
Didma1_15877_g1981_t1	superfamily	215186	490	1035	0	830.824	cl33465	PLN02327 superfamily
Stely1_6668_TW65_97109T0	superfamily	215186	469	1012	0	824.661	cl33465	PLN02327 superfamily
Aurpu_var_sub1_3702_gm1_3702_g	superfamily	178748	44	195	0.00013	44.1467	cl25752	PLN03209 superfamily
Capfu1_223502_MIX1467_177_100	superfamily	235906	5	238	0.000154	44.07	cl35530	PRK07003 superfamily
Hypcro1_445098_gm1_5606_g	superfamily	236090	40	212	0.000965	41.5101	cl35613	PRK07764 superfamily
Apope1_198129_CE198128_3691	superfamily	236090	61	236	0.002087	40.7397	cl35613	PRK07764 superfamily
Podap1_30093_CE30092_701	superfamily	236090	140	271	0.001845	41.1249	cl35613	PRK07764 superfamily
ColoR110_1_90609_estExt_Genewise1Plus_C_290088	superfamily	236090	48	241	0.000254	43.8213	cl35613	PRK07764 superfamily
Roster1_151755_CE151754_7395	superfamily	236090	5	203	5.08E-06	48.8289	cl35613	PRK07764 superfamily
HyprubER1909_1_254078_gm1_1626_g	superfamily	236138	74	220	1.05E-05	47.9382	cl35634	PRK07994 superfamily
Cadosp1_561021_estExt_Genemark4_C_3_t10189	superfamily	236333	91	266	0.004533	39.3066	cl35739	PRK08691 superfamily
Morste1_487689_CE487688_2475	superfamily	236333	4	174	0.000925	41.6178	cl35739	PRK08691 superfamily
Morpal1_319283_CE319282_2402	superfamily	236333	4	174	0.000925	41.6178	cl35739	PRK08691 superfamily
FvanT23_1_394662_fgenesh1_pm_139_7	superfamily	236669	14	188	0.001109	41.6091	cl35903	PRK10263 superfamily
FvanT36_1_297991_fgenesh1_pm_2_409	superfamily	236669	14	188	0.001109	41.6091	cl35903	PRK10263 superfamily
Aulhe2_421138_gm1_5273_g	superfamily	236669	24	162	0.000416	42.7647	cl35903	PRK10263 superfamily
Trieff1_474649_CE474648_7098	superfamily	236669	113	224	0.000269	43.5351	cl35903	PRK10263 superfamily
Leppa1_390814_fgenesh1_pg_69_21	superfamily	236669	7	246	0.000711	41.9943	cl35903	PRK10263 superfamily
HyFL1284_2_228178_CE228177_11130	superfamily	236669	38	193	4.3E-06	49.3131	cl35903	PRK10263 superfamily
Lophiu1_113392_CE113391_4005	superfamily	236669	46	253	1.69E-05	47.3871	cl35903	PRK10263 superfamily
Aaoar1_93849_CE93848_9637	superfamily	236669	41	285	0.002409	40.8387	cl35903	PRK10263 superfamily
Patat1_987049_fgenesh1_pg_14_117	superfamily	236669	7	168	0.000588	42.3795	cl35903	PRK10263 superfamily
Pcapi1_206177_CE206176_24194	superfamily	236669	47	165	5.86E-05	45.8463	cl35903	PRK10263 superfamily
Phycap1_351286_gm1_3145_g	superfamily	236669	47	165	5.86E-05	45.8463	cl35903	PRK10263 superfamily
Pcit129764_489400_gm1_7490_g	superfamily	236669	26	148	0.000464	42.7647	cl35903	PRK10263 superfamily
Phcitr1_306714_gm1_1049_g	superfamily	236669	26	148	0.000464	42.7647	cl35903	PRK10263 superfamily
Phcapit2_543119_gm1_3153_g	superfamily	236669	47	165	5.86E-05	45.8463	cl35903	PRK10263 superfamily
Phcap1_168250_CE168249_9134	superfamily	236669	47	165	6.17E-05	45.8463	cl35903	PRK10263 superfamily
Phycapi2_219400_CE219399_1857	superfamily	236669	47	165	0.00013	44.6907	cl35903	PRK10263 superfamily
Phcapi1_291824_estExt_fgenesh1_pm_C_3_t10407	superfamily	236669	33	151	4.95E-05	45.8463	cl35903	PRK10263 superfamily
DalEC12_1_17712_gm1_320_g	superfamily	236669	34	232	1.37E-05	47.7723	cl35903	PRK10263 superfamily
Dales1_5996_CE5995_4861	superfamily	236669	38	237	6.58E-06	48.5427	cl35903	PRK10263 superfamily
Hyptru1_24467_CE24466_13293	superfamily	236669	120	217	0.003273	40.0683	cl35903	PRK10263 superfamily
FusoxFo47_12978_rna_gnl_WGS_AFMM_mrna_FOZG_05187T0	superfamily	236669	20	219	3.57E-05	46.2315	cl35903	PRK10263 superfamily
Fusoxrad1_994_rna_gnl_WGS_AGNB_mrna_FOCG_00688T0	superfamily	236669	20	219	3.57E-05	46.2315	cl35903	PRK10263 superfamily
FusoxFo47_2_289919_MIX7086_701_98	superfamily	236669	20	219	3.57E-05	46.2315	cl35903	PRK10263 superfamily
Fox26365_1_347348_fgenesh1_pm_Chr_508_59	superfamily	236669	3	184	0.000577	42.3795	cl35903	PRK10263 superfamily
Fusoxlyc1_10350_rna_gnl_WGS_AGBH_mrna_FOWG_02698T0	superfamily	236669	20	219	3.57E-05	46.2315	cl35903	PRK10263 superfamily
Lepor2_77718_CE77717_586	superfamily	236669	23	235	6.07E-05	45.4611	cl35903	PRK10263 superfamily
CadmalM221_1_286_IFR05_003681_1	superfamily	236669	28	199	2.22E-05	47.0019	cl35903	PRK10263 superfamily
Wilmi1_591550_estExt_Genewise1_C_2280035	superfamily	236669	100	264	0.002299	40.4535	cl35903	PRK10263 superfamily
Colca1_113980_CE113979_2077	superfamily	236669	18	164	1.11E-07	54.3207	cl35903	PRK10263 superfamily
Gloac1_1357794_gm1_12903_g	superfamily	236669	1	152	0.009327	38.5275	cl35903	PRK10263 superfamily
Coler1_617967_fgenesh1_pg_37_59	superfamily	236669	1	145	0.00356	40.0683	cl35903	PRK10263 superfamily
Colso1_241763_CE241762_1348	superfamily	236669	4	172	2.87E-05	46.6167	cl35903	PRK10263 superfamily
Colsu1_680404_gm1_4911_g	superfamily	236669	1	145	0.004542	39.6831	cl35903	PRK10263 superfamily
Colsub1_7241_transcript_KDN70858	superfamily	236669	1	145	0.004542	39.6831	cl35903	PRK10263 superfamily
Colzo1_245175_CE245174_1392	superfamily	236669	18	159	1.91E-07	53.5503	cl35903	PRK10263 superfamily
Colfa1_78916_CE78915_1747	superfamily	236669	9	140	0.000498	42.3795	cl35903	PRK10263 superfamily

	Color1_1128_CORC01_10899T0	superfamily	236669	9	210	3.61E-06	49.6983	cl35903	PRK10263 superfamily
	Anttom1_387379_fgenesh1_pm_3_361	superfamily	236669	9	159	0.007946	38.9127	cl35903	PRK10263 superfamily
	Colch1_8855_mrna_4388	superfamily	236669	9	145	1.41E-05	47.7723	cl35903	PRK10263 superfamily
	Lompr1_6758_transcript_PKS10158	superfamily	236669	167	294	0.000411	43.1499	cl35903	PRK10263 superfamily
	LobpulAf1_1160656_MIX37499_1261_30	superfamily	236669	5	170	0.001339	41.2239	cl35903	PRK10263 superfamily
	LobpulSp1_155974_CE155973_18235	superfamily	236669	5	170	0.00124	41.2239	cl35903	PRK10263 superfamily
	LobpulSw1_2886809_CE2886808_42086	superfamily	236669	5	170	0.001339	41.2239	cl35903	PRK10263 superfamily
	Colto1_266_CT0861_07648T0	superfamily	236669	6	144	2.2E-06	50.4687	cl35903	PRK10263 superfamily
	Coltof1_155909_mrna_2160	superfamily	236669	6	144	2.2E-06	50.4687	cl35903	PRK10263 superfamily
	Colin1_7920_mrna_7920	superfamily	236669	6	138	5.38E-08	55.4763	cl35903	PRK10263 superfamily
	Tripare1_302794_CE302793_4242	superfamily	236669	38	212	0.000214	43.9203	cl35903	PRK10263 superfamily
	Trihar1_55701_CE55700_44869	superfamily	236669	4	185	5.17E-05	45.4611	cl35903	PRK10263 superfamily
	Trica1_287933_CE287932_9112	superfamily	236669	35	238	0.000559	42.3795	cl35903	PRK10263 superfamily
	Triag1_384580_CE384579_6480	superfamily	236669	38	227	0.000121	44.6907	cl35903	PRK10263 superfamily
	Ilyrob1_525858_gm1_962_g	superfamily	236669	6	193	7.66E-05	45.4611	cl35903	PRK10263 superfamily
	Tribrev1_162712_CE162711_4200	superfamily	236669	26	211	0.000134	44.6907	cl35903	PRK10263 superfamily
	Tribre1_124724_g10182_t1	superfamily	236669	26	211	0.000134	44.6907	cl35903	PRK10263 superfamily
	Altta1_122362_ALMCTG_441_jigsaw_mRNA_5	superfamily	236669	20	306	8.84E-05	45.0759	cl35903	PRK10263 superfamily
	Cloaq1_626910_fgenesh1_pm_128_21	superfamily	236669	18	221	8.55E-06	48.5427	cl35903	PRK10263 superfamily
	Stano2_6175_SNOG_12917_3	superfamily	236669	72	269	0.000193	43.9203	cl35903	PRK10263 superfamily
	Stely1_6668_TW65_97109T0	superfamily	236669	19	304	7.55E-06	50.0835	cl35903	PRK10263 superfamily
	Dalloc1_184715_CE184714_47817	superfamily	236669	5	157	0.000707	41.9943	cl35903	PRK10263 superfamily
	Delco1_260495_CE260494_3054	superfamily	236669	104	252	0.000783	41.9943	cl35903	PRK10263 superfamily
	Glonio1_91381_CE91380_6007	superfamily	236669	18	219	4.19E-07	52.7799	cl35903	PRK10263 superfamily
	Photr1_434446_estExt_Genewise1_C_580035	superfamily	236669	34	273	0.002693	40.4535	cl35903	PRK10263 superfamily
	Paront1_56984_gm4_492_g	superfamily	236669	43	220	0.001142	41.6091	cl35903	PRK10263 superfamily
	Rhiund1_813034_gm1_15180_g	superfamily	236669	41	234	0.001095	41.6091	cl35903	PRK10263 superfamily
	Polfu1_566286_gm1_8335_g	superfamily	236669	8	178	0.008836	38.5275	cl35903	PRK10263 superfamily
	Trifla1_250364_CE250363_3355	superfamily	236669	14	226	0.000185	43.9203	cl35903	PRK10263 superfamily
	Veren1_539327_fgenesh1_pg_3_78	superfamily	236669	19	190	0.001031	41.6091	cl35903	PRK10263 superfamily
	HyFL1150_1_461858_gm1_693_g	superfamily	236669	1	223	0.001662	40.8387	cl35903	PRK10263 superfamily
	FvanT77_1_381303_fgenesh1_pm_17_152	superfamily	236669	14	188	0.001109	41.6091	cl35903	PRK10263 superfamily
	FvanT78_1_267704_fgenesh1_pm_33_40	superfamily	236669	14	188	0.001109	41.6091	cl35903	PRK10263 superfamily
	FvanT95_1_10819_CE10818_4168	superfamily	236669	32	206	0.001304	41.2239	cl35903	PRK10263 superfamily
	FusspF11_1_917740_gm1_4476_g	superfamily	236669	32	206	0.001304	41.2239	cl35903	PRK10263 superfamily
	Necha2_79226_fgenesh1_pg_sca_3_chr4_2_0000627	superfamily	236669	14	188	0.001109	41.6091	cl35903	PRK10263 superfamily
	FvanT110_1_519667_fgenesh1_pm_10_326	superfamily	236669	14	188	0.001109	41.6091	cl35903	PRK10263 superfamily
	FvanT126_1_435157_fgenesh1_pm_32_98	superfamily	236669	14	188	0.001109	41.6091	cl35903	PRK10263 superfamily
	FvanT159_1_474576_fgenesh1_pm_19_106	superfamily	236669	25	130	0.002111	40.4535	cl35903	PRK10263 superfamily
	Fusva1_408761_e_gw1_23_340_1	superfamily	236669	14	188	0.001109	41.6091	cl35903	PRK10263 superfamily
	Fusvan1_527675_fgenesh1_pm_2_651	superfamily	236669	14	188	0.001109	41.6091	cl35903	PRK10263 superfamily
	FvanT200_1_291867_fgenesh1_pm_13_88	superfamily	236669	14	188	0.001109	41.6091	cl35903	PRK10263 superfamily
	FvanT219_1_327855_fgenesh1_pm_28_87	superfamily	236669	14	188	0.001109	41.6091	cl35903	PRK10263 superfamily
	FvanT299_1_349400_fgenesh1_pg_17_119	superfamily	236669	14	188	0.001109	41.6091	cl35903	PRK10263 superfamily
	FvanT31_1_365445_gm1_7552_g	superfamily	236669	32	206	0.001304	41.2239	cl35903	PRK10263 superfamily
	FvanT33_1_28398_CE28397_5619	superfamily	236669	32	206	0.001304	41.2239	cl35903	PRK10263 superfamily
	Fusso1_460384_fgenesh1_pg_3_681	superfamily	236669	10	188	0.001053	41.6091	cl35903	PRK10263 superfamily
	Fusmo1_294118_gm1_9940_g	superfamily	236669	16	162	0.006927	38.9127	cl35903	PRK10263 superfamily
	Rhesp1_267548_CE267547_14851	superfamily	236797	26	144	0.00824	38.1224	cl35972	PRK10927 superfamily
	Bombom1_494078_e_gw1_4_1521_1	superfamily	236797	129	254	0.000889	41.204	cl35972	PRK10927 superfamily
	Dallocu1_20247_CE20246_10827	superfamily	237057	58	251	0.000144	44.0963	cl36108	PRK12323 superfamily
	Dalba1_95645_CE95644_5177	superfamily	237057	56	253	1.04E-05	47.9483	cl36108	PRK12323 superfamily
	Tricap1_409356_fgenesh1_pm_3_1	superfamily	237057	249	415	0.000721	42.5555	cl36108	PRK12323 superfamily
	Thiap1_446666_CE446665_1818	superfamily	184287	117	195	0.000542	42.8102	cl42934	PRK13735 superfamily
	Achstr1_304862_CE304861_2266	superfamily	237865	181	297	0.000633	42.394	cl36447	PRK14951 superfamily
	Ascsa1_4923_4923_t	superfamily	237874	52	160	0.004563	39.3735	cl36455	PRK14971 superfamily
	Darbet1_37294_CE37293_5697	superfamily	185594	100	227	0.001026	41.9843	cl33180	PTZ00395 superfamily
	Photr1_118101_CE118100_165	superfamily	185628	26	106	0.002816	40.4434	cl33186	PTZ00449 superfamily
	Plecto1_682281_gm1_5247_g	superfamily	185628	35	206	1.12E-05	47.7622	cl33186	PTZ00449 superfamily
	XylFL2044_787654_gm1_12274_g	superfamily	185628	129	245	0.007203	38.9026	cl33186	PTZ00449 superfamily
	Trinov1_760538_fgenesh1_pm_7_19	superfamily	185628	3	239	0.00085	41.9842	cl33186	PTZ00449 superfamily
	Aspnut1_176459_CE176458_7976	superfamily	227709	2	154	0.005851	39.4891	cl34999	ROM1 superfamily
	Trigui1_3403_transcript_OPB42291	superfamily	227709	2	253	0.000116	44.8819	cl34999	ROM1 superfamily
	Triha1_81433_e_gw1_4_2099_1	superfamily	227709	2	253	2.67E-06	49.8895	cl34999	ROM1 superfamily
	Penatra1_75682_gm1_9769_g	superfamily	445932	288	371	0.000123	43.4338	cl02808	RT_like superfamily
Pez-VosA	Byssp1_5217_PVAR5_7753	superfamily	436461	569	609	0.002207	36.5446	cl39730	CSN7a_helixI superfamily
	Uncre1_5118_URET_05255	superfamily	412063	405	491	0.008694	37.6974	cl41678	cubilin_NTD superfamily
	Crypto1_455589_fgenesh1_pg_11_399	superfamily	430824	341	513	0.002197	40.5782	cl40852	CytochromB561_N superfamily
	CadmalM34_1_8343_IFR04_000561_1	superfamily	455861	261	451	0.002426	40.5792	cl42516	dnaA superfamily
	Aspfalc1_41868_CE41867_244	superfamily	455861	113	248	0.001485	39.4236	cl42516	dnaA superfamily
	Aspneoc1_179146_gm1_3773_g	superfamily	455861	264	467	0.001191	41.3496	cl42516	dnaA superfamily

Aspneoi1_108394_CE108393_7219	superfamily	455861	262	428	0.002229	40.194	cl42516	dnaA superfamily
Aspsep1_353862_gm1_519_g	superfamily	455861	288	469	0.003238	39.8088	cl42516	dnaA superfamily
Tubae1_7570_GSTUAT00000570001	superfamily	455861	206	427	9.63E-07	51.3648	cl42516	dnaA superfamily
Morsem1_780854_gm1_1210_g	superfamily	455861	219	404	1.37E-05	47.5128	cl42516	dnaA superfamily
Mordis1_541789_CE541788_18590	superfamily	455861	219	404	1.37E-05	47.5128	cl42516	dnaA superfamily
Mordun1_680394_gm1_2291_g	superfamily	455861	219	404	1.85E-06	50.5944	cl42516	dnaA superfamily
Morgal1_724471_gm1_10013_g	superfamily	455861	219	404	1.41E-05	47.5128	cl42516	dnaA superfamily
Morkaki1_926045_gm1_2820_g	superfamily	455861	219	404	1.37E-05	47.5128	cl42516	dnaA superfamily
Morcra1_717040_e_gw1_7_985_1	superfamily	455861	214	398	2.38E-06	50.2092	cl42516	dnaA superfamily
Aspamy1_4984_CE4983_790	superfamily	436069	271	429	0.004042	39.5148	cl39316	DUF5585 superfamily
Aspspe1_1_114624_CE114623_1257	superfamily	436069	317	428	0.003958	39.5148	cl39316	DUF5585 superfamily
Podcur1_422056_gm1_6359_g	superfamily	436069	297	384	0.001409	40.6704	cl39316	DUF5585 superfamily
Uncre1_5118_URET_05255	specific	213391	539	863	1.5E-25	110.612	cd12148	fungal_TF_MHR
Myrdu1_266973_fgenesh1_pg_4_218	superfamily	406402	346	447	0.008803	37.9869	cl25765	G_path_suppress superfamily
Bisme1_471275_CE471274_29115	superfamily	282904	298	475	0.001436	41.4404	cl37540	Herpes_BLLF1 superfamily
Bismed1_226541_CE226540_3449	superfamily	282904	298	475	0.00054	42.596	cl37540	Herpes_BLLF1 superfamily
Biscog1_585681_fgenesh1_pm_56_24	superfamily	282904	295	522	0.001376	41.4404	cl37540	Herpes_BLLF1 superfamily
CtCBS203_1_560099_CE560098_4446	superfamily	282904	296	473	0.001681	41.0552	cl37540	Herpes_BLLF1 superfamily
Bisma1_407090_gm1_3391_g	superfamily	282904	309	472	0.000965	41.8256	cl37540	Herpes_BLLF1 superfamily
XyFL1272_2_169956_CE169955_9926	superfamily	282904	276	426	0.000413	42.9812	cl37540	Herpes_BLLF1 superfamily
Jumvir1_84304_CE84303_4923	superfamily	282904	301	511	3.18E-05	46.8332	cl37540	Herpes_BLLF1 superfamily
Lolmi1_137768_CE137767_1045	superfamily	282904	250	481	0.001439	41.4404	cl37540	Herpes_BLLF1 superfamily
Neo1551_1_464410_e_gw1_13_392_1	superfamily	282904	283	445	0.006451	39.1292	cl37540	Herpes_BLLF1 superfamily
Magor1_9726_rna_MGG_00617T0	superfamily	273733	322	458	0.002461	40.185	cl25888	hnRNP-L_PTB superfamily
Aspfloc1_75202_CE75201_445	superfamily	312941	260	389	9.32E-05	44.614	cl26621	Med15 superfamily
Asptsu1_327914_CE327913_3092	superfamily	312941	277	465	0.009674	38.4508	cl26621	Med15 superfamily
Aspthe1_205577_CE205576_789	superfamily	312941	268	465	0.001203	41.5324	cl26621	Med15 superfamily
Aspuda1_6286_AUD_2434	superfamily	312941	266	458	0.00222	40.3768	cl26621	Med15 superfamily
Penant1_9045_PENANT_c005G02727T0	superfamily	312941	244	395	0.000655	41.9176	cl26621	Med15 superfamily
Aspell1_321357_e_gw1_44_139_1	superfamily	312941	240	433	0.005827	38.836	cl26621	Med15 superfamily
Asppust1_241990_gm1_234_g	superfamily	130689	239	381	0.005177	39.0198	cl31127	PABP-1234 superfamily
Aspaus1_276540_CE276539_22863	superfamily	130689	251	371	0.003565	39.405	cl31127	PABP-1234 superfamily
Asppuu1_223613_fgenesh1_pm_25_70	superfamily	130689	251	372	0.000274	43.257	cl31127	PABP-1234 superfamily
Aspoli1_1_6250_CE6249_657	superfamily	130689	243	405	0.001401	40.9458	cl31127	PABP-1234 superfamily
Aspve1_50183_fgenesh1_pm_2_510	superfamily	130689	251	371	0.003414	39.7902	cl31127	PABP-1234 superfamily
Aspegy1_551680_MIX3662_607_41	superfamily	130689	258	391	9.43E-05	44.4126	cl31127	PABP-1234 superfamily
PenchWisc1_1_147883_PCH_Pc22g06890	superfamily	130689	211	359	0.008389	38.2494	cl31127	PABP-1234 superfamily
Pench1_82669_e_gw1_5_1961_1	superfamily	130689	211	359	0.008389	38.2494	cl31127	PABP-1234 superfamily
Aspsublat1_266359_gm1_214_g	superfamily	130689	376	485	0.000333	43.257	cl31127	PABP-1234 superfamily
Greab1_512346_gm1_4707_g	superfamily	130689	246	389	0.003569	39.7902	cl31127	PABP-1234 superfamily
Asppac1_380648_CE380647_8768	superfamily	130689	192	301	0.002665	39.405	cl31127	PABP-1234 superfamily
Aspele1_405398_MIX9454_238_56	superfamily	130689	260	406	0.001065	41.331	cl31127	PABP-1234 superfamily
Aspste1_29431_CE29430_987	superfamily	130689	260	406	0.001065	41.331	cl31127	PABP-1234 superfamily
Penth1_215024_MIX4352_33_55	superfamily	130689	174	338	0.006009	39.0198	cl31127	PABP-1234 superfamily
Penfla1_2487_PENFLA_c016G00194T0	superfamily	130689	284	432	0.008245	38.6346	cl31127	PABP-1234 superfamily
Asplaci1_168252_CE168251_518	superfamily	130689	260	376	0.001898	40.5606	cl31127	PABP-1234 superfamily
Aspall1_110655_CE110654_1123	superfamily	130689	262	394	0.00231	40.1754	cl31127	PABP-1234 superfamily
Asppet1_303650_fgenesh1_pm_269_7	superfamily	130689	260	403	0.001935	40.5606	cl31127	PABP-1234 superfamily
Aspnaka1_250958_MIX6022_1239_43	superfamily	130689	260	403	0.001655	40.5606	cl31127	PABP-1234 superfamily
Pencop1_5028_PENCOP_c003G08035T0	superfamily	130689	250	358	0.000222	43.6422	cl31127	PABP-1234 superfamily
Penvul1_7577_PENVUL_c005G02799T0	superfamily	130689	264	354	0.002515	40.1754	cl31127	PABP-1234 superfamily
Penja1_427213_fgenesh1_pg_19_117	superfamily	130689	240	367	0.004431	39.0198	cl31127	PABP-1234 superfamily
Penox1_6666_EPS31735	superfamily	130689	254	374	0.000345	42.8718	cl31127	PABP-1234 superfamily
Asphet1_385899_estExt_fgenesh1_pm_C_60218	superfamily	130689	265	405	0.006289	39.0198	cl31127	PABP-1234 superfamily
Aspsil1_65863_CE65862_1215	superfamily	130689	240	403	9.97E-05	44.4126	cl31127	PABP-1234 superfamily
Mordim1_640321_fgenesh1_pm_4_189	superfamily	130689	220	357	0.000521	42.4866	cl31127	PABP-1234 superfamily
Kalbru1_871301_fgenesh1_pm_45_11	superfamily	130689	332	463	1.73E-05	47.109	cl31127	PABP-1234 superfamily
Aspniv1_268600_fgenesh1_pm_28_119	superfamily	130689	257	389	0.005454	39.0198	cl31127	PABP-1234 superfamily
Collu1_169917_CE169916_1745	superfamily	130689	200	328	0.001496	40.5606	cl31127	PABP-1234 superfamily
Collup1_110_mrna_110	superfamily	130689	182	310	0.001622	40.1754	cl31127	PABP-1234 superfamily
Colta1_12878_mrna_10737	superfamily	130689	200	328	0.00147	40.5606	cl31127	PABP-1234 superfamily
Colme1_4403_mrna_4403	superfamily	130689	200	328	0.00147	40.5606	cl31127	PABP-1234 superfamily
Colny1_1022328_mrna_14048	superfamily	130689	182	310	0.001983	40.1754	cl31127	PABP-1234 superfamily
Colpa1_2583_mrna_12400	superfamily	130689	200	328	0.00147	40.5606	cl31127	PABP-1234 superfamily
Colsi1_981014_mrna_9134	superfamily	130689	182	310	0.001622	40.1754	cl31127	PABP-1234 superfamily
Colsp1_16062_mrna_10460	superfamily	130689	200	328	0.001458	40.5606	cl31127	PABP-1234 superfamily
Colab1_2590_mrna_2590	superfamily	130689	200	328	0.00147	40.5606	cl31127	PABP-1234 superfamily
Colac2_413842_CE413841_4233	superfamily	130689	200	328	0.001203	40.9458	cl31127	PABP-1234 superfamily
Colco1_2466_mrna_10454	superfamily	130689	200	328	0.00147	40.5606	cl31127	PABP-1234 superfamily
Pseudest1_4175_VC83_07417T0	superfamily	130689	308	427	0.000393	43.257	cl31127	PABP-1234 superfamily
Aspcorr1_17087_fgenesh1_pg_2_71	superfamily	130689	276	385	0.001087	41.331	cl31127	PABP-1234 superfamily
PseVKM4520_1_8623_transcript_KFZ04145	superfamily	130689	299	483	9.1E-05	45.183	cl31127	PABP-1234 superfamily

Colgo1_593752_estExt_Genewise1_C_700088	superfamily	401645	113	220	0.000409	41.9452	cl37801	PAT1 superfamily
Morpun1_621604_gm1_12914_g	superfamily	401645	265	411	4.06E-06	49.264	cl37801	PAT1 superfamily
Dibbae1_12106_DIBA_005317_RA	superfamily	401645	257	509	1.18E-05	48.1084	cl37801	PAT1 superfamily
Pyrom1_170259_CE170258_704	superfamily	401645	338	454	0.002259	40.7896	cl37801	PAT1 superfamily
Disven1_216070_CE216069_7120	superfamily	401645	331	476	0.000688	42.3304	cl37801	PAT1 superfamily
Gyrinf1_909748_gm1_3201_g	superfamily	401645	306	455	3.28E-05	46.5676	cl37801	PAT1 superfamily
Morpun1_305293_CE305292_7373	superfamily	401645	314	460	2.27E-06	50.4196	cl37801	PAT1 superfamily
Morsem1_780854_gm1_1210_g	superfamily	401645	337	459	7.11E-07	51.9604	cl37801	PAT1 superfamily
Morsep1_638235_gm1_8211_g	superfamily	401645	336	458	0.000151	44.2564	cl37801	PAT1 superfamily
Mordis1_541789_CE541788_18590	superfamily	401645	337	459	7.11E-07	51.9604	cl37801	PAT1 superfamily
Mordun1_680394_gm1_2291_g	superfamily	401645	337	459	4.74E-07	52.3456	cl37801	PAT1 superfamily
Morexim1_669366_gm4_3327_g	superfamily	401645	311	456	3.13E-06	49.6492	cl37801	PAT1 superfamily
Morgal1_724471_gm1_10013_g	superfamily	401645	337	459	2.66E-07	53.116	cl37801	PAT1 superfamily
Morhis1_593900_gm1_3542_g	superfamily	401645	337	459	8.97E-07	51.5752	cl37801	PAT1 superfamily
Morimp1_459131_CE459130_2521	superfamily	401645	253	456	2.09E-06	50.4196	cl37801	PAT1 superfamily
Morkaki1_926045_gm1_2820_g	superfamily	401645	337	459	7.11E-07	51.9604	cl37801	PAT1 superfamily
Morpop1_743234_gm1_3876_g	superfamily	401645	312	458	4.41E-06	49.264	cl37801	PAT1 superfamily
Morarb1_809916_gm1_3983_g	superfamily	401645	336	458	7.52E-07	51.9604	cl37801	PAT1 superfamily
Morcon1_112030_CE112029_2693	superfamily	401645	336	458	0.000151	44.2564	cl37801	PAT1 superfamily
Byssp1_8598_PVAR5_3070	superfamily	227507	291	362	0.003166	40.0923	cl34930	PBP1 superfamily
Paevar1_446888_fgenesh1_pg_10_284	superfamily	227507	257	331	0.000115	44.7147	cl34930	PBP1 superfamily
Asptran1_51458_CE51457_955	superfamily	227507	297	349	0.000476	42.7887	cl34930	PBP1 superfamily
Asppsev1_141556_CE141555_2824	superfamily	227507	267	333	0.004081	39.7071	cl34930	PBP1 superfamily
Aspthe1_205577_CE205576_789	superfamily	227507	278	344	0.000518	42.4035	cl34930	PBP1 superfamily
Theau2_114413_CE114412_1037	superfamily	227507	278	389	0.000529	42.4035	cl34930	PBP1 superfamily
Aspdim1_324561_CE324560_2992	superfamily	227507	278	320	0.007988	38.5515	cl34930	PBP1 superfamily
Aspsep1_353862_gm1_519_g	superfamily	227507	267	369	4.5E-05	45.8703	cl34930	PBP1 superfamily
Cercau1_498346_fgenesh1_pg_5_892	superfamily	188093	305	443	0.005206	39.2951	cl28216	PCC superfamily
Asppul1_480791_fgenesh1_pm_21_77	superfamily	330753	194	362	0.001939	40.4564	cl25932	Peptidase_S21 superfamily
Aspamb1_60919_CE60918_588	superfamily	330753	194	351	0.002611	40.0712	cl25932	Peptidase_S21 superfamily
Asplep1_202044_MIX5308_613_43	superfamily	330753	207	355	0.000158	43.9232	cl25932	Peptidase_S21 superfamily
Horth1_7120_B0A50_07715	superfamily	222914	187	357	0.003326	39.5337	cl14378	PHA02666 superfamily
Bissp1_590921_fgenesh1_pg_8_307	superfamily	177476	289	321	0.006711	38.5691	cl14386	PHA02694 superfamily
Bissp1_681968_gm1_2465_g	superfamily	177476	289	321	0.006711	38.5691	cl14386	PHA02694 superfamily
XylFL0043_310258_gm1_1083_g	superfamily	165099	305	472	0.001336	41.6621	cl28620	PHA02732 superfamily
XyFL0064_1_194375_CE194374_25869	superfamily	165099	305	472	0.003694	40.1213	cl28620	PHA02732 superfamily
XylFL0933_397879_gm1_4623_g	superfamily	165099	305	472	0.001043	42.0473	cl28620	PHA02732 superfamily
Clabul1_919033_fgenesh1_pm_2_592	superfamily	165099	266	354	0.002428	40.1213	cl28620	PHA02732 superfamily
XylFL0662B_180039_CE180038_13105	superfamily	223021	195	431	4.26E-05	46.4701	cl33720	PHA03247 superfamily
Aspcro1_1_180735_gm1_2689_g	superfamily	223021	158	433	9.84E-05	44.9293	cl33720	PHA03247 superfamily
Aspdese1_119105_CE119104_857	superfamily	223021	158	416	0.001398	41.0773	cl33720	PHA03247 superfamily
Asprec1_1_191908_fgenesh1_pm_8_110	superfamily	223021	145	403	0.007804	38.7661	cl33720	PHA03247 superfamily
Whamic1_278726_CE278725_9429	superfamily	223021	243	456	2.03E-05	47.6257	cl33720	PHA03247 superfamily
Aspmul1_1_76639_CE76638_1411	superfamily	223021	185	416	3.83E-05	46.0849	cl33720	PHA03247 superfamily
Aspsy1_141197_e_gw1_1_3398_1	superfamily	223021	185	415	0.007937	38.7661	cl33720	PHA03247 superfamily
Hypmon1_280077_gm1_6001_g	superfamily	223021	251	451	0.003215	40.3069	cl33720	PHA03247 superfamily
Aspcreb1_562272_fgenesh1_pm_19_136	superfamily	223021	185	415	0.007937	38.7661	cl33720	PHA03247 superfamily
Aspang1_236049_CE236048_16335	superfamily	223021	185	415	0.008428	38.7661	cl33720	PHA03247 superfamily
Aspten1_724032_fgenesh1_pm_18_108	superfamily	223021	185	415	0.005683	39.1513	cl33720	PHA03247 superfamily
Aurpu_var_sub1_151699_CE37585_2806	superfamily	223021	215	561	2.17E-07	54.1741	cl33720	PHA03247 superfamily
HyFL1284_2_494377_CE494376_14687	superfamily	223021	259	453	0.000262	44.1589	cl33720	PHA03247 superfamily
HyFL0543_1_254717_CE254716_4703	superfamily	223021	270	454	0.000955	42.2329	cl33720	PHA03247 superfamily
Hyptru1_275948_CE275947_5910	superfamily	223021	289	450	5.66E-05	46.0849	cl33720	PHA03247 superfamily
DaFL1419_1_345443_gm1_2395_g	superfamily	223021	253	497	0.004858	39.9217	cl33720	PHA03247 superfamily
HyFL1150_1_342001_CE342000_17983	superfamily	223021	245	450	0.009489	38.7661	cl33720	PHA03247 superfamily
Durrog2_329871_CE329870_6300	superfamily	223021	318	481	0.00169	41.4625	cl33720	PHA03247 superfamily
Diadis1_433914_CE433913_3160	superfamily	223021	236	459	0.00555	39.5365	cl33720	PHA03247 superfamily
AurpulNBB1_61416_fgenesh1_pg_Chr_3_937	superfamily	223021	220	522	0.001584	41.4625	cl33720	PHA03247 superfamily
Aurpu_var_pul1_125056_CE125055_833	superfamily	223021	202	504	0.003359	40.3069	cl33720	PHA03247 superfamily
Morpun1_621604_gm1_12914_g	superfamily	223021	148	369	0.000837	41.8477	cl33720	PHA03247 superfamily
Xylhe1_58943_CE58942_4440	superfamily	223021	164	513	0.001252	41.8477	cl33720	PHA03247 superfamily
Trigu1_1079894_gm1_687_g	superfamily	223021	233	512	0.000482	43.0033	cl33720	PHA03247 superfamily
Aspres1_155452_fgenesh1_pm_44_23	superfamily	223021	188	437	0.003047	40.3069	cl33720	PHA03247 superfamily
Aspneoca1_420959_fgenesh1_pm_48_28	superfamily	223021	188	436	0.002451	40.3069	cl33720	PHA03247 superfamily
Aspparac1_23117_fgenesh1_pm_19_61	superfamily	223021	198	422	0.000569	42.6181	cl33720	PHA03247 superfamily
Aspcapp1_640448_MIX5729_12396_39	superfamily	223021	185	438	1.87E-06	50.7073	cl33720	PHA03247 superfamily
Aspcost1_14161_CE14160_477	superfamily	223021	185	438	2.58E-06	49.9369	cl33720	PHA03247 superfamily
Aspcri1_817_SI65_00818T0	superfamily	223021	185	438	5.55E-05	45.6997	cl33720	PHA03247 superfamily
Aspxer1_433916_fgenesh1_pm_206_6	superfamily	223021	195	432	0.000333	43.3885	cl33720	PHA03247 superfamily

Aspneoa1_168768_MIX2340_395_42	superfamily	223021	156	366	0.009599	38.3809	cl33720	PHA03247 superfamily
Aspmeg1_684705_fgenesh1_pm_328_2	superfamily	223021	185	438	2.11E-06	50.3221	cl33720	PHA03247 superfamily
Aspgl1_207532_CE24847_5914	superfamily	223021	185	438	2.76E-06	49.9369	cl33720	PHA03247 superfamily
Aspend1_191559_MIX5366_11314_30	superfamily	223021	185	438	4.71E-06	49.1665	cl33720	PHA03247 superfamily
Aspapp1_408155_fgenesh1_pm_14_54	superfamily	223021	185	438	3.96E-06	49.5517	cl33720	PHA03247 superfamily
Aspcib1_17512_fgenesh1_pm_226_1	superfamily	223021	185	438	2.11E-06	50.3221	cl33720	PHA03247 superfamily
Aspumb1_258685_fgenesh1_pm_12_28	superfamily	223021	185	438	3.89E-06	49.5517	cl33720	PHA03247 superfamily
Aspspir1_529994_fgenesh1_pm_627_1	superfamily	223021	185	438	1.3E-06	51.0925	cl33720	PHA03247 superfamily
Eurhe1_445917_e_gw1_1_76_1	superfamily	223021	185	415	4.1E-06	49.1665	cl33720	PHA03247 superfamily
Aspses1_304000_gm1_5022_g	superfamily	223021	259	434	0.006847	39.1513	cl33720	PHA03247 superfamily
Aspoc7043_1_263276_CE263275_3164	superfamily	223021	153	405	0.003078	40.3069	cl33720	PHA03247 superfamily
Penswi1_394680_MIX5874_498_78	superfamily	223021	184	439	0.005932	39.1513	cl33720	PHA03247 superfamily
Penbr2_59855_gm1_7018_g	superfamily	223021	185	375	0.003283	39.9217	cl33720	PHA03247 superfamily
PenbrAgRF18_1_352932_gm1_7044_g	superfamily	223021	185	375	0.003283	39.9217	cl33720	PHA03247 superfamily
Penbi1_395577_gm1_2196_g	superfamily	223021	176	421	0.000379	43.0033	cl33720	PHA03247 superfamily
Pencit1_254762_CE254761_2471	superfamily	223021	199	423	3.13E-06	49.5517	cl33720	PHA03247 superfamily
Pengl1_108744_CE108743_3476	superfamily	223021	197	407	0.009101	38.3809	cl33720	PHA03247 superfamily
Aspdim1_324561_CE324560_2992	superfamily	223021	195	451	0.009198	38.7661	cl33720	PHA03247 superfamily
Penci1_68756_fgenesh1_pm_1_55	superfamily	223021	186	417	0.007102	38.7661	cl33720	PHA03247 superfamily
Tubgib1_792848_CE792847_7656	superfamily	223021	213	485	1.1E-11	67.656	cl33720	PHA03247 superfamily
Morste1_980513_fgenesh1_pg_42_56	superfamily	223021	219	484	0.000109	44.9293	cl33720	PHA03247 superfamily
Morulm1_217933_CE217932_11673	superfamily	223021	216	490	2.65E-05	47.2405	cl33720	PHA03247 superfamily
Morpal1_665669_fgenesh1_pg_7_155	superfamily	223021	219	484	0.000109	44.9293	cl33720	PHA03247 superfamily
Morper1_529511_gm1_2481_g	superfamily	223021	216	490	6.78E-05	45.6997	cl33720	PHA03247 superfamily
Morame1_289268_CE289267_25619	superfamily	223021	215	490	0.000137	44.9293	cl33720	PHA03247 superfamily
Acastr1_126086_AST1_003652_RA	superfamily	223021	175	488	3.63E-06	49.9369	cl33720	PHA03247 superfamily
Rhiund1_233681_CE233680_7786	superfamily	223021	197	517	1.48E-06	51.0925	cl33720	PHA03247 superfamily
Tubcan1_380855_CE380854_3366	superfamily	223021	173	474	8.52E-09	58.4113	cl33720	PHA03247 superfamily
Tubma1_358792_estExt_fgenesh1_pm_C_590028	superfamily	223021	196	494	5.81E-08	55.7149	cl33720	PHA03247 superfamily
Tubmel1_1564813_CE1564812_2534	superfamily	223021	204	495	3.75E-08	56.4853	cl33720	PHA03247 superfamily
Morpun1_305293_CE305292_7373	superfamily	223021	197	418	0.00041	43.3885	cl33720	PHA03247 superfamily
Mortrid1_302523_CE302522_7959	superfamily	223021	216	480	0.002913	40.3069	cl33720	PHA03247 superfamily
Morpop1_743234_gm1_3876_g	superfamily	223021	195	416	0.000569	42.6181	cl33720	PHA03247 superfamily
Wilmi1_23576_CE23575_26409	superfamily	223021	195	480	0.000275	43.7737	cl33720	PHA03247 superfamily
Symko1_912548_gm1_2423_g	superfamily	223021	187	497	1.1E-08	58.0261	cl33720	PHA03247 superfamily
Chove1_1826598_fgenesh1_pm_17_34	superfamily	223021	196	454	4.54E-06	49.5517	cl33720	PHA03247 superfamily
Drest1_4164_transcript_EWC44790	superfamily	223021	204	502	0.001655	41.4625	cl33720	PHA03247 superfamily
Monha1_3262_H072_3272m_01	superfamily	223021	188	505	1.56E-06	51.0925	cl33720	PHA03247 superfamily
Artol1_3367_AOL_s00054g700m_01	superfamily	223021	191	492	4.67E-07	52.6333	cl33720	PHA03247 superfamily
Rhesp1_478824_fgenesh1_pg_9_739	superfamily	223021	287	465	0.000912	42.2329	cl33720	PHA03247 superfamily
XylFL1651_14110_CE14109_2030	superfamily	223021	305	473	0.00456	39.9217	cl33720	PHA03247 superfamily
Aspsul1_272486_MIX4213_69_29	superfamily	165527	262	337	0.005984	38.9412	cl29788	PHA03269 superfamily
Sposc1_1671_transcript_KJR88247	superfamily	223039	198	520	0.004637	39.7693	cl33723	PHA03307 superfamily
Pezech1_522757_fgenesh1_pg_4_233	superfamily	223039	181	448	0.000741	42.4657	cl33723	PHA03307 superfamily
Xylni1_489422_gm1_3787_g	superfamily	223039	246	470	0.008445	38.9989	cl33723	PHA03307 superfamily
Lepor2_18801_CE18800_899	superfamily	223065	256	461	0.002347	40.4356	cl33729	PHA03378 superfamily
Leptod1_121175_CE121174_236	superfamily	223065	255	460	0.00255	40.4356	cl33729	PHA03378 superfamily
Aspsublat1_266359_gm1_214_g	superfamily	223065	307	535	0.007338	39.28	cl33729	PHA03378 superfamily
Asppha1_9206_e_gw1_21_6_1	superfamily	223065	183	342	0.008359	38.5096	cl33729	PHA03378 superfamily
Penbra1_2892_PMG11_10838_1	superfamily	223066	197	415	0.001052	41.5825	cl33730	PHA03379 superfamily
DalEC12_1_16361_fgenesh1_pg_45_62	superfamily	215130	369	447	0.002168	40.8428	cl33436	PLN02217 superfamily
Dales1_408390_gm1_3308_g	superfamily	215130	369	447	0.002168	40.8428	cl33436	PLN02217 superfamily
Ascim1_142889_CE142888_20276	superfamily	178748	357	552	0.003603	39.9095	cl25752	PLN03209 superfamily
Aspala1_66912_CE66911_923	superfamily	235738	199	327	0.009404	38.1514	cl29858	PRK06199 superfamily
Aspflo1_170160_fgenesh1_pm_193_3	superfamily	235738	199	319	0.001654	40.4626	cl29858	PRK06199 superfamily
Asppseute1_515099_gm1_696_g	superfamily	235738	199	327	0.007755	38.1514	cl29858	PRK06199 superfamily
Asppterr1_57289_CE57288_800	superfamily	235738	199	327	0.007755	38.1514	cl29858	PRK06199 superfamily
Aspisr1_11271_e_gw1_52_33_1	superfamily	236090	179	377	0.002479	40.3545	cl35613	PRK07764 superfamily
DimP5P66_683541_gm1_1866_g	superfamily	236669	235	348	1.2E-05	48.1575	cl35903	PRK10263 superfamily
Atrpi1_262617_CE262616_1724	superfamily	236669	347	476	1.54E-05	47.7723	cl35903	PRK10263 superfamily
Helsp1_214291_CE214290_1000	superfamily	236669	317	462	3.21E-05	47.0019	cl35903	PRK10263 superfamily
Rhili1_70431_gm1_598_g	superfamily	236669	314	496	0.000791	42.7647	cl35903	PRK10263 superfamily
Ascsa1_3256_3256_t	superfamily	236669	233	434	0.007611	39.2979	cl35903	PRK10263 superfamily
NeoP8C63_1_502875_gw1_8_967_1	superfamily	236669	186	304	0.00129	40.4535	cl35903	PRK10263 superfamily
Penox1_6666_EPS31735	superfamily	236669	175	290	0.004099	39.6831	cl35903	PRK10263 superfamily
Morpra1_659604_gm1_3795_g	superfamily	236669	260	358	0.009584	38.1423	cl35903	PRK10263 superfamily
Disven1_216070_CE216069_7120	superfamily	236669	249	402	1.29E-06	51.2391	cl35903	PRK10263 superfamily
Mortrid1_302523_CE302522_7959	superfamily	236669	270	398	2.58E-05	47.0019	cl35903	PRK10263 superfamily
Morhis1_593900_gm1_3542_g	superfamily	236669	269	374	0.003848	40.0683	cl35903	PRK10263 superfamily
Morarb1_809916_gm1_3983_g	superfamily	236669	269	373	0.000115	45.0759	cl35903	PRK10263 superfamily
Morcra1_717040_e_gw1_7_985_1	superfamily	236669	350	462	0.000142	44.6907	cl35903	PRK10263 superfamily
Kalbru1_871301_fgenesh1_pm_45_11	superfamily	236669	250	379	0.008033	38.9127	cl35903	PRK10263 superfamily
Drest1_4164_transcript_EWC44790	superfamily	236669	235	358	8.14E-05	45.4611	cl35903	PRK10263 superfamily
Usnflo1_883971_gm1_180_g	superfamily	236669	276	500	3.45E-06	49.6983	cl35903	PRK10263 superfamily
Clasam1_183810_e_gw1_114_31_1	superfamily	236669	271	379	0.001335	41.2239	cl35903	PRK10263 superfamily

Pse03VT05_1_10084_transcript_OBT81208	superfamily	236669	449	601	0.000183	45.0759	cl35903	PRK10263 superfamily
PseVKM103_1_3106_transcript_KFY70960	superfamily	236669	296	421	0.000973	42.3795	cl35903	PRK10263 superfamily
Psever1_5459_transcript_OBT96375	superfamily	236669	296	421	0.000973	42.3795	cl35903	PRK10263 superfamily
Racan1_16771_transcript_OQO11207	superfamily	236776	305	434	0.007895	38.4688	cl35960	PRK10856 superfamily
Vercon1_515741_gm1_4728_g	superfamily	184281	252	304	6.02E-05	45.5843	cl42933	PRK13729 superfamily
Cocim1_1365_CIMG_01530T0	superfamily	184918	119	257	0.00117	40.3115	cl33039	PRK14954 superfamily
Cocpo1_1_701_XM_003065653_1	superfamily	184918	119	257	0.005968	38.0003	cl33039	PRK14954 superfamily
Cocpos1_1642_transcript_EFW21485	superfamily	184918	119	257	0.005968	38.0003	cl33039	PRK14954 superfamily
Thiar1_799126_gm1_5654_g	superfamily	184923	316	399	0.000304	43.1299	cl33044	PRK14959 superfamily
Diahe1_11252_transcript_POS73860	superfamily	237874	281	390	0.002256	40.1439	cl36455	PRK14971 superfamily
Aspfu_A1163_1_105763_CADAFUBT00006741m_01	superfamily	237874	314	420	0.006039	38.9883	cl36455	PRK14971 superfamily
Aspfu1_5533_Afu4g10860_mRNA	superfamily	237874	314	420	0.006039	38.9883	cl36455	PRK14971 superfamily
Aspfumis1_355574_gm1_9052_g	superfamily	237874	293	399	0.005294	38.9883	cl36455	PRK14971 superfamily
Talis1_3287_transcript_CRG84753	superfamily	237874	347	424	0.009585	38.2179	cl36455	PRK14971 superfamily
Delst1_397656_gm1_3590_g	superfamily	140276	242	387	2.4E-05	46.9486	cl25531	PTZ00249 superfamily
Cocst1_652272_gm1_6102_g	superfamily	185594	216	520	0.000141	45.0659	cl33180	PTZ00395 superfamily
Dalgra1_449902_gm1_6286_g	superfamily	185594	255	462	5.19E-05	46.2215	cl33180	PTZ00395 superfamily
Cadosp1_19220_CE19219_2329	superfamily	185594	283	464	0.006799	39.288	cl33180	PTZ00395 superfamily
Anntru1_450935_fgenesh1_pm_10_44	superfamily	185594	307	467	0.001623	41.2139	cl33180	PTZ00395 superfamily
Annnit1_486340_fgenesh1_pm_4_141	superfamily	185594	301	461	0.000307	43.5251	cl33180	PTZ00395 superfamily
AnFL0455_488237_fgenesh1_pm_11_17	superfamily	185594	307	467	0.005367	39.6732	cl33180	PTZ00395 superfamily
Roster1_238892_CE238891_7903	superfamily	185594	265	469	1.65E-05	47.7623	cl33180	PTZ00395 superfamily
Dalcal1_454946_gm1_1006_g	superfamily	185594	295	486	0.000402	43.1399	cl33180	PTZ00395 superfamily
Nemsp1_7155_e_gw1_14_99_1	superfamily	185594	302	481	0.00353	40.0584	cl33180	PTZ00395 superfamily
Annbov1_186727_CE186726_4036	superfamily	185594	254	478	4.49E-06	49.6883	cl33180	PTZ00395 superfamily
Annmin1_336594_gm1_7801_g	superfamily	185594	308	464	0.006821	39.288	cl33180	PTZ00395 superfamily
Ternu1_356180_gm1_9443_g	superfamily	185594	381	456	0.009673	38.5176	cl33180	PTZ00395 superfamily
DallocAZ0526_2_245442_CE245441_4272	superfamily	185594	255	462	0.004099	40.0584	cl33180	PTZ00395 superfamily
Dalloc1_208028_CE208027_5292	superfamily	185594	255	462	0.00403	40.0584	cl33180	PTZ00395 superfamily
Dallocu1_390419_gm1_8387_g	superfamily	185594	255	462	0.001261	41.5991	cl33180	PTZ00395 superfamily
Dalver1_16357_CE16356_3947	superfamily	185594	255	462	0.000398	43.1399	cl33180	PTZ00395 superfamily
Daldec1_393854_gm1_9767_g	superfamily	185594	255	461	0.000645	42.7547	cl33180	PTZ00395 superfamily
ThoPMI491_1_832405_gm1_17937_g	superfamily	185594	232	425	1.2E-05	47.7623	cl33180	PTZ00395 superfamily
Hyppol1_515196_fgenesh1_pm_1_908	superfamily	185594	266	477	8E-07	51.9995	cl33180	PTZ00395 superfamily
HypEC38_3_48899_CE48898_7281	superfamily	185594	186	399	0.00631	39.288	cl33180	PTZ00395 superfamily
Hypsub2_597084_gm1_11347_g	superfamily	185594	313	489	0.000277	43.9103	cl33180	PTZ00395 superfamily
Hypfus1_70173_CE70172_3691	superfamily	185594	251	475	0.000266	43.9103	cl33180	PTZ00395 superfamily
DalEC12_1_16361_fgenesh1_pg_45_62	superfamily	185594	299	468	0.004121	40.0584	cl33180	PTZ00395 superfamily
Dales1_408390_gm1_3308_g	superfamily	185594	299	468	0.004121	40.0584	cl33180	PTZ00395 superfamily
DaFL1419_1_345443_gm1_2395_g	superfamily	185594	353	469	0.000717	42.3695	cl33180	PTZ00395 superfamily
Dalba1_464621_CE464620_5566	superfamily	185594	299	468	0.000187	44.2955	cl33180	PTZ00395 superfamily
CodFL1790_1_478297_CE478296_8621	superfamily	185594	198	410	3.27E-05	46.6067	cl33180	PTZ00395 superfamily
Hypcro1_250519_CE250518_1983	superfamily	185594	300	479	1.13E-06	51.6143	cl33180	PTZ00395 superfamily
Khuory1_486037_gm1_7291_g	superfamily	185594	291	481	0.000914	41.9843	cl33180	PTZ00395 superfamily
Microd1_393019_CE393018_808	superfamily	185594	255	372	9.91E-05	44.6807	cl33180	PTZ00395 superfamily
HyNC1633_2_285756_CE285755_4352	superfamily	185594	291	460	0.000143	44.6807	cl33180	PTZ00395 superfamily
Aulhe2_145701_CE145700_2094	superfamily	185594	289	408	5.87E-07	52.7699	cl33180	PTZ00395 superfamily
XylFL2044_528640_CE528639_10210	superfamily	185594	253	479	0.002288	40.8288	cl33180	PTZ00395 superfamily
Hypcer1_444443_gm1_8998_g	superfamily	185594	305	466	0.001038	41.9843	cl33180	PTZ00395 superfamily
Sepmu1_150595_estExt_fgenesh1_kg_C_80121	superfamily	185594	185	371	6.59E-05	45.4511	cl33180	PTZ00395 superfamily
Seppo1_104957_e_gw1_49_50_1	superfamily	185594	185	371	5.43E-05	45.8363	cl33180	PTZ00395 superfamily
Aspfla1_10994_CE10993_1982	superfamily	185594	210	415	2.78E-05	46.6067	cl33180	PTZ00395 superfamily
Asplup1_30430_CE30429_806	superfamily	185594	210	428	0.001482	41.2139	cl33180	PTZ00395 superfamily
Aspaurful1_66057_CE66056_4904	superfamily	185594	210	428	0.001482	41.2139	cl33180	PTZ00395 superfamily
LecAK0013_1_428236_gm1_5036_g	superfamily	185594	355	423	0.007076	38.9028	cl33180	PTZ00395 superfamily
Conioc1_1218938_gm1_9586_g	superfamily	185594	316	419	0.008005	38.9028	cl33180	PTZ00395 superfamily
Rhier1_107831_CE107830_2851	superfamily	185594	329	539	0.000395	43.5251	cl33180	PTZ00395 superfamily
Calpu1_4735_NODE_3258_length_38655_cov_28_g5970_t1	superfamily	185594	253	469	0.000764	42.3695	cl33180	PTZ00395 superfamily
Astsub1_449165_fgenesh1_pm_10_114	superfamily	185594	321	514	0.000325	43.5251	cl33180	PTZ00395 superfamily
Rosne1_5623_transcript_GAP86838	superfamily	185594	322	505	0.00232	40.8288	cl33180	PTZ00395 superfamily
Xylcur114988_1_309955_CE309954_31611	superfamily	185594	321	515	0.000981	41.9843	cl33180	PTZ00395 superfamily
Kalpfe1_790107_gm1_2759_g	superfamily	185628	198	431	2.95E-05	46.9918	cl33186	PTZ00449 superfamily
Chathe1_4717_rna4029_CTHT_0040580	superfamily	185628	270	358	0.005352	38.9026	cl33186	PTZ00449 superfamily
Aspind2_1_51049_CE51048_481	superfamily	273167	297	406	0.004283	39.1079	cl36702	rad23 superfamily
Aspden1_306100_fgenesh1_pm_31_32	superfamily	273167	312	415	0.008974	37.9523	cl36702	rad23 superfamily
Aspnid1_7660_AN1959	superfamily	273167	312	415	0.008974	37.9523	cl36702	rad23 superfamily
Aspsim1_1_210230_fgenesh1_pm_268_3	superfamily	273167	310	413	0.003747	39.4931	cl36702	rad23 superfamily
Aspquaf1_141330_fgenesh1_pm_53_8	superfamily	273167	310	413	0.009905	37.9523	cl36702	rad23 superfamily

	Aspquag1_206370_fgenesh1_pm_1_116	superfamily	273167	310	413	0.008839	37.9523	cl36702	rad23 superfamily
	Aspne1_174748_fgenesh1_pm_14_125	superfamily	273167	310	413	0.003285	39.4931	cl36702	rad23 superfamily
	Nemabo1_276610_fgenesh1_pm_36_32	superfamily	227709	258	437	0.004442	39.8743	cl34999	ROM1 superfamily
	Xylcas124033_1_536617_fgenesh1_pm_1_33	superfamily	227709	255	437	0.000433	42.9559	cl34999	ROM1 superfamily
	Stano2_12077_SNOG_06311_3	superfamily	227709	209	404	0.001952	40.6447	cl34999	ROM1 superfamily
	Xylcub1_115841_CE115840_8851	superfamily	227709	226	408	0.005432	39.4891	cl34999	ROM1 superfamily
	Xyllon1_324705_CE324704_6320	superfamily	227709	255	437	0.001762	41.0299	cl34999	ROM1 superfamily
	Xylpal1_13187_CE13186_16498	superfamily	227709	230	437	0.002324	40.6447	cl34999	ROM1 superfamily
	Xylpal124036_1_502044_CE502043_22996	superfamily	227709	259	437	0.000851	42.1855	cl34999	ROM1 superfamily
	Xylscr1_65814_CE65813_19150	superfamily	227709	255	437	0.009432	38.7187	cl34999	ROM1 superfamily
	XyFL1777_1_188759_CE188758_29744	superfamily	227709	230	437	0.002324	40.6447	cl34999	ROM1 superfamily
	Xylacu1_386729_CE386728_33109	superfamily	227709	291	436	0.004318	39.8743	cl34999	ROM1 superfamily
	Xylber1_610185_fgenesh1_pm_22_73	superfamily	227709	255	437	0.000121	44.8819	cl34999	ROM1 superfamily
	Xylcube1_404787_CE404786_17919	superfamily	227709	255	437	0.006149	39.4891	cl34999	ROM1 superfamily
	Xylcur1_163938_CE163937_19555	superfamily	227709	241	439	0.000167	44.4967	cl34999	ROM1 superfamily
	CopCBS38678_1_396434_gm1_8166_g	superfamily	435789	309	360	0.007251	36.0387	cl25441	SdrD_B superfamily
	Atrpi1_262617_CE262616_1724	superfamily	273740	476	540	0.005579	39.23	cl36947	sex-lethal superfamily
	Helsp1_214291_CE214290_1000	superfamily	273740	454	518	0.003178	39.6152	cl36947	sex-lethal superfamily
	Clasph1_3161_CSPH_06747_R0	superfamily	431280	209	362	0.001846	40.8572	cl25817	SOG2 superfamily
	Cerbe1_9556_transcript_PPJ55552	superfamily	431280	178	333	0.004322	39.3164	cl25817	SOG2 superfamily
	Aspdese1_119105_CE119104_857	superfamily	425992	246	343	0.000542	41.8666	cl03073	TYA superfamily
	Aspstec1_1_285561_MIX1500_3147_72	superfamily	425992	200	328	7.61E-05	44.563	cl03073	TYA superfamily
	Asprec1_1_191908_fgenesh1_pm_8_110	superfamily	425992	233	330	0.000237	43.0222	cl03073	TYA superfamily
	Aspind2_1_51049_CE51048_481	superfamily	425992	212	342	0.000398	42.2518	cl03073	TYA superfamily
	Aspauran1_592382_fgenesh1_pm_62_4	superfamily	425992	205	335	6.16E-05	44.9482	cl03073	TYA superfamily
	Aspestel1_162011_gm1_1077_g	superfamily	425992	202	332	0.000528	41.8666	cl03073	TYA superfamily
	Aspsulp1_600936_MIX11217_22819_41	superfamily	425992	222	352	0.000632	41.8666	cl03073	TYA superfamily
	Aspden1_306100_fgenesh1_pm_31_32	superfamily	425992	227	357	0.000615	41.8666	cl03073	TYA superfamily
	Aspnid1_7660_AN1959	superfamily	425992	227	357	0.000615	41.8666	cl03073	TYA superfamily
	ConPMI546_971001_gm1_8133_g	superfamily	425992	268	395	0.006285	38.3998	cl03073	TYA superfamily
	Aspsim1_1_210230_fgenesh1_pm_268_3	superfamily	425992	225	355	0.000127	43.7926	cl03073	TYA superfamily
	Aspquaf1_141330_fgenesh1_pm_53_8	superfamily	425992	225	355	0.000606	41.8666	cl03073	TYA superfamily
	Aspquag1_206370_fgenesh1_pm_1_116	superfamily	425992	225	355	0.000616	41.8666	cl03073	TYA superfamily
	Aspfove1_148079_fgenesh1_pm_8_53	superfamily	425992	225	355	0.000715	41.4814	cl03073	TYA superfamily
	Aspne1_174748_fgenesh1_pm_14_125	superfamily	425992	225	355	0.000143	43.7926	cl03073	TYA superfamily
Sac-Tap-VelB	Cybfa1_4877_transcript:ONH66347	superfamily	436858	27	59	0.009882	37.5133	cl40954	DUF5695 superfamily
	Saico1_6726_gm1_2578_g	superfamily	237874	112	192	0.007569	38.9883	cl36455	PRK14971 superfamily
Sac-VeA	Lipst1_1_267564_CE146447_5148	superfamily	236669	348	502	0.002075	41.2239	cl35903	PRK10263 superfamily
	Cybfa1_2210_transcript:ONH68672	superfamily	236669	289	394	0.002756	40.0683	cl35903	PRK10263 superfamily
