# Supplementary material for: Phylogenomics analysis of velvet regulators in the fungal kingdom
Source: Microbiol Spectr. 2024 Jan 5;12(2):e03717-23. doi: 10.1128/spectrum.03717-23 (PMC10845976; doi:10.1128/spectrum.03717-23)
Supplement: Table S2 — Top 300 similar structures of VosA velvet domain in the PDB database by the Dali server. [file spectrum.03717-23-s0003.pdf]

Table S2. Top 300 similar structures of VosA velvet domain in the PDB database by the Dali server

	Chain	Z	RMSD	lali	nres	Sequence identity %	Description
1	1bvo-A	10.6	2.5	112	175	15	DNA DUPLEX;
2	3do7-B	10.4	2.7	118	293	18	AVIAN RETICULOENDOTHELIOSIS VIRAL (V-REL)
3	1ooa-B	10.4	2.7	118	313	17	RNA APTAMER;
4	1a3q-B	10.4	2.8	115	285	17	DNA (5'-D(*GP*GP*GP*GP*AP*AP*TP*CP*CP*CP*C)-3');
5	1ooa-A	10.4	2.6	116	313	16	RNA APTAMER;
6	3gut-F	10.3	2.9	120	312	17	TRANSCRIPTION FACTOR P65;
7	3gut-B	10.3	2.8	119	312	17	TRANSCRIPTION FACTOR P65;
8	1a3q-A	10.3	2.7	116	285	19	DNA (5'-D(*GP*GP*GP*GP*AP*AP*TP*CP*CP*CP*C)-3');
9	2o61-B	10.3	2.8	118	314	17	36-MER;
10	2v2t-B	10.2	2.8	118	313	18	TRANSCRIPTION FACTOR RELB;
11	1imh-D	10	2.9	120	281	17	TonEBP/DNA COMPLEX
12	4y5u-B	9.9	3.5	117	438	11	SIGNAL TRANSDUCER AND ACTIVATOR OF TRANSCRIPTION
13	1nfk-B	9.9	3	119	312	17	DNA (5'-D(*TP*GP*GP*GP*AP*AP*TP*TP*CP*CP*C)-3');
14	1owr-M	9.9	3.2	121	284	19	NFAT1 MONOMERIC BINDING SITE, PLUS STRAND;
15	1imh-C	9.9	3	121	281	17	TonEBP/DNA COMPLEX
16	1p7h-N	9.9	3.3	121	286	19	5'-D(*AP*AP*TP*GP*GP*GP*GP*AP*CP*TP*TP*TP*CP*CP*A
17	1owr-Q	9.9	3.2	123	284	20	NFAT1 MONOMERIC BINDING SITE, PLUS STRAND;
18	1owr-P	9.8	3.3	123	284	20	NFAT1 MONOMERIC BINDING SITE, PLUS STRAND;
19	1pzu-I	9.8	3.1	119	276	19	5'-D(*TP*TP*GP*AP*GP*GP*AP*AP*TP*TP*TP*CP*CP*A)-
20	1owr-N	9.8	3.3	123	284	19	NFAT1 MONOMERIC BINDING SITE, PLUS STRAND;
21	1pzu-B	9.8	3.1	119	276	19	5'-D(*TP*TP*GP*AP*GP*GP*AP*AP*TP*TP*TP*CP*CP*A)-
22	1pzu-D	9.8	3.2	119	276	19	5'-D(*TP*TP*GP*AP*GP*GP*AP*AP*TP*TP*TP*CP*CP*A)-
23	1pzu-M	9.8	3.1	119	276	19	5'-D(*TP*TP*GP*AP*GP*GP*AP*AP*TP*TP*TP*CP*CP*A)-
24	4y5u-A	9.7	3.4	116	445	11	SIGNAL TRANSDUCER AND ACTIVATOR OF TRANSCRIPTION
25	3qrf-N	9.7	3.4	124	286	19	NUCLEAR FACTOR OF ACTIVATED T-CELLS, CYTOPLASMIC
26	1pzu-L	9.7	3.7	123	276	19	5'-D(*TP*TP*GP*AP*GP*GP*AP*AP*TP*TP*TP*CP*CP*A)-
27	1pzu-H	9.7	3.1	119	276	19	5'-D(*TP*TP*GP*AP*GP*GP*AP*AP*TP*TP*TP*CP*CP*A)-
28	1p7h-O	9.7	3.2	121	286	19	5'-D(*AP*AP*TP*GP*GP*GP*GP*AP*CP*TP*TP*TP*CP*CP*A
29	3qrf-M	9.7	3.4	123	286	20	NUCLEAR FACTOR OF ACTIVATED T-CELLS, CYTOPLASMIC
30	1p7h-M	9.7	3.2	120	286	19	5'-D(*AP*AP*TP*GP*GP*GP*GP*AP*CP*TP*TP*TP*CP*CP*A
31	2as5-M	9.6	3.3	123	287	20	Structure of the DNA binding domains of NFAT and FOXP2 bound specifically to DNA
32	2as5-N	9.6	3.3	123	287	20	Structure of the DNA binding domains of NFAT and FOXP2 bound specifically to DNA
33	2o93-M	9.6	3.3	121	284	19	KAPPAB ENHANCER ELEMENT, DNA 25-MER;
34	4y5w-A	9.5	3.6	121	478	11	SIGNAL TRANSDUCER AND ACTIVATOR OF TRANSCRIPTION
35	1yvl-A	9.5	3.4	124	652	10	SIGNAL TRANSDUCER AND ACTIVATOR OF TRANSCRIPTION
36	6mbz-B	9.5	3.7	124	522	11	SIGNAL TRANSDUCER AND ACTIVATOR OF TRANSCRIPTION
37	1s9k-C	9.5	3.4	121	280	17	HUMAN IL-2 ARRE1 PROMOTER ELEMENT, PLUS STRAND;
38	2o93-L	9.4	3.4	122	287	19	KAPPAB ENHANCER ELEMENT, DNA 25-MER;
39	1ikn-A	9.4	3.3	116	282	15	PROTEIN (NF-KAPPA-B P65 SUBUNIT);
40	1lei-B	9.4	2.7	117	312	17	NUCLEAR FACTOR NF-KAPPA-B P65 SUBUNIT
41	5d39-C	9.4	3.7	123	484	10	SIGNAL TRANSDUCER AND ACTIVATOR OF TRANSCRIPTION
42	2o61-A	9.2	3.4	119	498	14	36-MER;
43	1ram-B	9.2	3.3	119	273	14	A NOVEL DNA RECOGNITION MODE BY NF-KB P65 HOMODIMER
44	6ggr-A	9.2	3.1	113	168	15	TRANSCRIPTION FACTOR P65;
45	2ram-B	9.1	3.3	118	273	14	A NOVEL DNA RECOGNITION MODE BY NF-KB P65 HOMODIMER
46	2i9t-A	9.1	3.6	123	275	15	Transcription factor p65
47	5u01-C	9.1	3.6	118	273	15	TRANSCRIPTION FACTOR P65;
48	1a02-N	9.1	3.4	120	280	19	STRUCTURE OF THE DNA BINDING DOMAINS OF NFAT, FOS AND JUN BOUND TO DNA
49	1lei-A	9	3.5	119	273	14	p65 subunit of NF-kappa B
50	5u01-B	9	3.4	119	273	14	TRANSCRIPTION FACTOR P65;
51	6nuq-A	9	3.8	120	522	10	SIGNAL TRANSDUCER AND ACTIVATOR OF TRANSCRIPTION
52	4y5w-D	8.9	3.4	120	472	11	SIGNAL TRANSDUCER AND ACTIVATOR OF TRANSCRIPTION
53	2v2t-A	8.9	3.2	122	279	13	TRANSCRIPTION FACTOR RELB;
54	1y1u-C	8.9	3.5	124	544	14	SIGNAL TRANSDUCER AND ACTIVATOR OF TRANSCRIPTION
55	1y1u-B	8.9	3.6	124	544	14	SIGNAL TRANSDUCER AND ACTIVATOR OF TRANSCRIPTION
56	1y1u-A	8.9	3.6	124	544	14	SIGNAL TRANSDUCER AND ACTIVATOR OF TRANSCRIPTION
57	1vkx-A	8.8	3.4	118	273	14	CRYSTAL STRUCTURE OF THE NFKB P50/P65 HETERODIMER COMPLEXED TO THE IMMUNOGLOBULIN KB DNA
58	1ram-A	8.8	3.6	117	273	15	A NOVEL DNA RECOGNITION MODE BY NF-KB P65 HOMODIMER
59	1le5-A	8.8	3.5	120	274	15	5'-D(*TP*GP*GP*GP*AP*AP*AP*TP*TP*CP*CP*T)-3';

60	3gut-G	8.8	3.6	117	273	15	TRANSCRIPTION FACTOR P65;
61	2ram-A	8.8	3.6	119	273	14	A NOVEL DNA RECOGNITION MODE BY NF-KB P65 HOMODIMER
62	1le9-B	8.8	2.7	120	312	17	5'-D(*TP*GP*GP*GP*AP*CP*TP*TP*TP*CP*CP*T)-3';
63	1gji-A	8.8	2.9	119	275	15	IL-2 CD28RE DNA;
64	1gji-B	8.7	3	122	275	14	IL-2 CD28RE DNA;
65	3gut-C	8.7	3.2	114	273	15	TRANSCRIPTION FACTOR P65;
66	5d39-A	8.7	3.8	125	489	12	SIGNAL TRANSDUCER AND ACTIVATOR OF TRANSCRIPTION
67	5u01-A	8.7	3.7	120	273	15	TRANSCRIPTION FACTOR P65;
68	1vkx-B	8.7	2.8	118	312	16	CRYSTAL STRUCTURE OF THE NFKB P50/P65 HETERODIMER COMPLEXED TO THE IMMUNOGLOBULIN KB DNA
69	1bg1-A	8.7	3.5	121	559	9	TRANSCRIPTION FACTOR STAT3B/DNA COMPLEX
70	6qhd-B	8.7	3.6	122	532	10	SIGNAL TRANSDUCER AND ACTIVATOR OF TRANSCRIPTION
71	3gut-E	8.6	3.6	118	273	14	TRANSCRIPTION FACTOR P65;
72	3gut-A	8.6	3.5	116	273	15	TRANSCRIPTION FACTOR P65;
73	4y5w-B	8.6	3.6	121	477	11	SIGNAL TRANSDUCER AND ACTIVATOR OF TRANSCRIPTION
74	6mbw-B	8.6	3.7	125	523	11	SIGNAL TRANSDUCER AND ACTIVATOR OF TRANSCRIPTION
75	1le9-E	8.5	3.5	118	273	14	5'-D(*TP*GP*GP*GP*AP*CP*TP*TP*TP*CP*CP*T)-3';
76	1le5-E	8.5	3.7	120	274	15	5'-D(*TP*GP*GP*GP*AP*AP*AP*TP*TP*CP*CP*T)-3';
77	1yvl-B	8.5	3.4	124	653	10	SIGNAL TRANSDUCER AND ACTIVATOR OF TRANSCRIPTION
78	6tlc-B	8.5	3.7	122	535	9	SIGNAL TRANSDUCER AND ACTIVATOR OF TRANSCRIPTION
79	1le5-F	8.5	2.9	119	313	17	5'-D(*TP*GP*GP*GP*AP*AP*AP*TP*TP*CP*CP*T)-3';
80	1uur-A	8.5	4.4	120	461	14	STATA PROTEIN;
81	1svc-P	8.4	2.8	118	311	16	NFKB P50 HOMODIMER BOUND TO DNA
82	3brd-A	8.4	3.5	117	427	15	CSL (Lag-1) bound to DNA with Lin-12 RAM peptide, P212121
83	6tlc-A	8.4	3.6	121	533	9	SIGNAL TRANSDUCER AND ACTIVATOR OF TRANSCRIPTION
84	6mbz-A	8.4	3.9	124	536	11	SIGNAL TRANSDUCER AND ACTIVATOR OF TRANSCRIPTION
85	6mbw-A	8.4	3.6	121	524	12	SIGNAL TRANSDUCER AND ACTIVATOR OF TRANSCRIPTION
86	1nfi-A	8.3	3.5	115	294	14	NF-KAPPA-B P65;
87	2i9t-B	8.3	2.9	119	312	16	Structure of NF-kB p65-p50 heterodimer bound to PRDII element of B-interferon promoter
88	3gut-H	8.2	2.7	119	312	17	TRANSCRIPTION FACTOR P65;
89	1nfi-C	8.2	3.5	115	300	14	NF-KAPPA-B P65;
90	2f8x-C	8.2	3.4	113	424	16	Crystal structure of activated Notch, CSL and MAML on HES-1 promoter DNA sequence
91	3cwg-B	8.2	3.7	120	507	10	SIGNAL TRANSDUCER AND ACTIVATOR OF TRANSCRIPTION
92	3gut-D	8.2	2.8	120	312	17	TRANSCRIPTION FACTOR P65;
93	1le9-F	8.2	2.9	120	312	17	5'-D(*TP*GP*GP*GP*AP*CP*TP*TP*TP*CP*CP*T)-3';
94	1le5-B	8.2	3	119	313	17	5'-D(*TP*GP*GP*GP*AP*AP*AP*TP*TP*CP*CP*T)-3';
95	5d39-B	8.2	3.7	123	484	11	SIGNAL TRANSDUCER AND ACTIVATOR OF TRANSCRIPTION
96	6qhd-A	8.2	3.6	122	530	10	SIGNAL TRANSDUCER AND ACTIVATOR OF TRANSCRIPTION
97	1le9-A	8.2	3.6	116	273	15	5'-D(*TP*GP*GP*GP*AP*CP*TP*TP*TP*CP*CP*T)-3';
98	1uus-A	8.1	4.4	121	465	14	STAT PROTEIN;
99	3cwg-A	8.1	3.7	120	501	10	SIGNAL TRANSDUCER AND ACTIVATOR OF TRANSCRIPTION
100	4y5w-C	8	3.4	120	465	10	SIGNAL TRANSDUCER AND ACTIVATOR OF TRANSCRIPTION
101	3iag-C	8	3.3	113	422	17	CSL (RBP-Jk) bound to HES-1 nonconsensus site
102	1ttu-A	8	4.5	117	435	15	Crystal Structure of CSL bound to DNA
103	1nfk-A	8	3.2	119	312	17	DNA (5'-D(*TP*GP*GP*GP*AP*AP*TP*TP*CP*CP*C)-3');
104	3do7-A	7.9	4.4	127	296	14	AVIAN RETICULOENDOTHELIOSIS VIRAL (V-REL)
105	3nbn-A	7.9	3.3	113	423	18	RECOMBINING BINDING PROTEIN SUPPRESSOR OF
106	3brf-A	7.9	3.4	117	423	15	CSL (Lag-1) bound to DNA with Lin-12 RAM peptide, C2221
107	3nbn-D	7.9	3.3	111	423	17	RECOMBINING BINDING PROTEIN SUPPRESSOR OF
108	6py8-C	7.9	3.4	112	429	17	NOTCH-REGULATED ANKYRIN REPEAT-CONTAINING PROTEIN
109	5eg6-C	7.9	2.9	108	413	18	DNA (5'-D(*AP*AP*TP*CP*TP*TP*TP*CP*CP*CP*AP*CP*AP
110	3v79-C	7.8	3.4	113	424	18	NEUROGENIC LOCUS NOTCH HOMOLOG PROTEIN 1;
111	4j2x-C	7.8	3.4	112	421	16	RECOMBINING BINDING PROTEIN SUPPRESSOR OF
112	1eao-B	7.7	2.3	95	124	9	RUNT-RELATED TRANSCRIPTION FACTOR 1;
113	1eao-A	7.7	2.3	95	124	9	RUNT-RELATED TRANSCRIPTION FACTOR 1;
114	1ljm-A	7.7	2.5	98	114	10	RUNX1 TRANSCRIPTION FACTOR;
115	1eaq-B	7.7	2.3	95	125	9	RUNT-RELATED TRANSCRIPTION FACTOR 1;
116	1p7h-L	7.7	3.3	121	286	19	5'-D(*AP*AP*TP*GP*GP*GP*GP*AP*CP*TP*TP*TP*CP*CP*A
117	2o93-O	7.6	3.5	124	287	19	KAPPAB ENHANCER ELEMENT, DNA 25-MER;
118	1eaq-A	7.6	2.2	94	124	10	RUNT-RELATED TRANSCRIPTION FACTOR 1;
119	6dks-G	7.6	3.2	110	422	19	DNA (5'-D(*AP*AP*TP*CP*TP*TP*TP*CP*CP*CP*AP*CP*AP
120	1hjc-A	7.6	2.5	98	118	10	RUNT-RELATED TRANSCRIPTION FACTOR 1;
121	1ean-A	7.5	2.1	94	114	10	RUNT-RELATED TRANSCRIPTION FACTOR 1;
122	4j2x-A	7.5	3.4	112	420	18	RECOMBINING BINDING PROTEIN SUPPRESSOR OF
123	2j6w-A	7.4	2.3	95	124	9	RUNT-RELATED TRANSCRIPTION FACTOR 1;
124	5zhu-C	7.4	3.3	110	180	13	MYELIN REGULATORY FACTOR;

125	1ljm-B	7.4	2.3	95	114	9	RUNX1 TRANSCRIPTION FACTOR;
126	1e50-Q	7.4	2.4	95	110	9	CORE-BINDING FACTOR ALPHA SUBUNIT;
127	3wts-F	7.4	2.5	98	118	10	RUNT-RELATED TRANSCRIPTION FACTOR 1;
128	3wtv-F	7.4	2.5	98	118	10	RUNT-RELATED TRANSCRIPTION FACTOR 1;
129	3wtt-F	7.4	2.5	98	118	10	RUNT-RELATED TRANSCRIPTION FACTOR 1;
130	1hjb-F	7.4	2.5	98	120	10	CCAAT/ENHANCER BINDING PROTEIN BETA;
131	3wtu-F	7.4	2.5	98	118	10	RUNT-RELATED TRANSCRIPTION FACTOR 1;
132	1io4-C	7.4	2.4	97	120	10	CSF-1R PROMOTER;
133	3wty-F	7.4	2.6	98	118	10	RUNT-RELATED TRANSCRIPTION FACTOR 1;
134	5zhu-B	7.3	3.3	111	183	13	MYELIN REGULATORY FACTOR;
135	5e24-E	7.3	4.3	114	424	16	MALTOSE-BINDING PERIPLASMIC PROTEIN;
136	1e50-E	7.3	2.7	100	119	10	CORE-BINDING FACTOR ALPHA SUBUNIT;
137	1e50-R	7.3	2.4	95	110	9	CORE-BINDING FACTOR ALPHA SUBUNIT;
138	3wtt-A	7.3	2.6	98	119	10	RUNT-RELATED TRANSCRIPTION FACTOR 1;
139	3wtw-F	7.3	2.5	98	119	10	RUNT-RELATED TRANSCRIPTION FACTOR 1;
140	1hjc-D	7.3	2.5	98	118	10	RUNT-RELATED TRANSCRIPTION FACTOR 1;
141	3wtu-A	7.3	2.6	98	118	10	RUNT-RELATED TRANSCRIPTION FACTOR 1;
142	3wtx-F	7.3	2.5	98	118	10	RUNT-RELATED TRANSCRIPTION FACTOR 1;
143	3wtx-A	7.3	2.6	98	118	10	RUNT-RELATED TRANSCRIPTION FACTOR 1;
144	3wtv-A	7.3	2.6	98	118	10	RUNT-RELATED TRANSCRIPTION FACTOR 1;
145	3wty-A	7.3	2.6	98	118	10	RUNT-RELATED TRANSCRIPTION FACTOR 1;
146	3wu1-A	7.3	2.5	98	123	10	RUNT-RELATED TRANSCRIPTION FACTOR 1;
147	1e50-G	7.3	2.5	98	119	10	CORE-BINDING FACTOR ALPHA SUBUNIT;
148	6ux2-A	7.2	3	113	619	9	SIGNAL TRANSDUCER AND ACTIVATOR OF TRANSCRIPTION
149	2j6w-B	7.2	2.3	95	124	9	RUNT-RELATED TRANSCRIPTION FACTOR 1;
150	1e50-A	7.2	2.4	95	115	11	CORE-BINDING FACTOR ALPHA SUBUNIT;
151	3x17-A	7.2	3.3	106	548	7	ENDOGLUCANASE;
152	4l18-E	7.2	2.5	98	141	10	RUNT-RELATED TRANSCRIPTION FACTOR 1;
153	4l18-A	7.2	2.5	98	142	10	RUNT-RELATED TRANSCRIPTION FACTOR 1;
154	1h9d-A	7.2	2.5	98	125	10	CORE-BINDING FACTOR ALPHA SUBUNIT1;
155	3wts-A	7.2	2.6	98	119	10	RUNT-RELATED TRANSCRIPTION FACTOR 1;
156	1h9d-C	7.2	2.5	98	125	10	CORE-BINDING FACTOR ALPHA SUBUNIT1;
157	4l0z-A	7.2	2.5	98	147	10	RUNT-RELATED TRANSCRIPTION FACTOR 1;
158	3wtw-A	7.2	2.6	98	118	10	RUNT-RELATED TRANSCRIPTION FACTOR 1;
159	1hjb-C	7.2	2.5	98	120	10	CCAAT/ENHANCER BINDING PROTEIN BETA;
160	5u01-D	7.1	3.2	116	273	15	TRANSCRIPTION FACTOR P65;
161	3x17-B	7.1	3.2	106	548	8	ENDOGLUCANASE;
162	5h5p-A	7.1	3.2	110	184	13	MYELIN REGULATORY FACTOR;
163	4l0y-A	7.1	2.5	98	128	10	RUNT-RELATED TRANSCRIPTION FACTOR 1;
164	1e50-C	7.1	2.5	98	125	10	CORE-BINDING FACTOR ALPHA SUBUNIT;
165	5e24-F	6.9	4.4	112	422	17	MALTOSE-BINDING PERIPLASMIC PROTEIN;
166	5yhu-B	6.9	3.3	110	182	13	MYELIN REGULATORY FACTOR;
167	5zhu-A	6.9	3.2	110	183	13	MYELIN REGULATORY FACTOR;
168	5yhu-A	6.8	3.5	111	191	13	MYELIN REGULATORY FACTOR;
169	1f02-I	6.8	2.6	94	282	7	INTIMIN;
170	1f00-I	6.7	2.9	94	282	7	INTIMIN;
171	3aaz-A	6.7	3.9	106	228	9	HUMANIZED RECOMBINANT FAB FRAGMENT OF A MURINE;
172	3ly6-A	6.7	3	92	683	7	PROTEIN-GLUTAMINE GAMMA-GLUTAMYLTRANSFERASE 2;
173	3ly6-B	6.7	3.1	92	683	7	PROTEIN-GLUTAMINE GAMMA-GLUTAMYLTRANSFERASE 2;
174	4fxk-B	6.7	3.3	97	697	9	COMPLEMENT C4 BETA CHAIN;
175	4d94-A	6.7	4.6	114	1274	10	THIOESTER-CONTAINING PROTEIN 1;
176	4r7f-A	6.6	3.2	107	397	7	UNCHARACTERIZED PROTEIN;
177	6grt-A	6.6	3.3	102	593	8	PAIRED IMMUNOGLOBULIN-LIKE RECEPTOR B;
178	3aaz-H	6.6	3.8	103	229	10	HUMANIZED RECOMBINANT FAB FRAGMENT OF A MURINE;
179	5uqy-D	6.6	3.4	101	218	8	ENVELOPE GLYCOPROTEIN GP1;
180	5jpn-A	6.6	4.2	114	651	11	COMPLEMENT C4-A;
181	6dks-C	6.5	3.3	113	417	19	DNA (5'-D(*AP*AP*TP*CP*TP*TP*TP*CP*CP*CP*AP*CP*AP
182	3lso-A	6.5	3.1	93	445	6	PUTATIVE MEMBRANE ANCHORED PROTEIN;
183	3lso-B	6.5	3.7	97	445	7	PUTATIVE MEMBRANE ANCHORED PROTEIN;
184	1p84-J	6.5	3.8	103	127	10	UBIQUINOL-CYTOCHROME C REDUCTASE COMPLEX CORE PRO
185	1kyo-U	6.5	3.8	102	127	9	UBIQUINOL-CYTOCHROME C REDUCTASE COMPLEX CORE PRO
186	2ibz-X	6.5	3.8	104	127	10	UBIQUINOL-CYTOCHROME-C REDUCTASE COMPLEX CORE PRO
187	5uqy-H	6.5	3.4	101	220	8	ENVELOPE GLYCOPROTEIN GP1;
188	6ysq-B	6.5	4.5	118	651	12	COMPLEMENT C4 BETA CHAIN;
189	4fxk-A	6.5	4.1	116	651	10	COMPLEMENT C4 BETA CHAIN;
190	5hcc-B	6.5	4.4	117	647	10	COMPLEMENT C5;
191	4e68-A	6.4	3.6	123	559	11	Unphosphorylated STAT3B core protein binding to dsDNA
192	2ice-A	6.4	3.6	106	642	9	COMPLEMENT C3 BETA CHAIN;

193	4b0m-M	6.4	4.3	105	212	10	F1 CAPSULE-ANCHORING PROTEIN;
194	6grq-A	6.4	3.3	102	536	7	PAIRED IMMUNOGLOBULIN-LIKE RECEPTOR B;
195	6h3j-C	6.4	3.7	108	397	8	PROTEIN INVOLVED IN GLIDING MOTILITY SPRA;
196	5weq-B	6.4	3.2	100	219	10	MR78 MUTANT LIGHT CHAIN;
197	3mln-A	6.4	2.7	109	206	12	TRANSCRIPTION FACTOR COE1;
198	5fo9-A	6.4	3.2	98	642	8	COMPLEMENT C3 BETA CHAIN;
199	4f37-F	6.4	3.8	102	225	9	COLICIN-E7 IMMUNITY PROTEIN;
200	4yzf-G	6.4	3.6	99	223	8	BAND 3 ANION TRANSPORT PROTEIN;
201	5a16-C	6.4	3.6	99	226	7	FAB4201 HEAVY CHAIN;
202	4yr6-A	6.4	3.4	99	215	10	HEAVY CHAIN OF 5G6;
203	4irz-H	6.4	3.8	102	217	9	INTEGRIN ALPHA4 SUBUNIT;
204	2erj-B	6.4	3.6	94	204	3	INTERLEUKIN-2 RECEPTOR ALPHA CHAIN;
205	4cn4-B	6.4	3.3	113	649	7	ALPHA-1,4-GLUCAN\:MALTOSE-1-PHOSPHATE MALTOSYLTRA
206	4fxg-B	6.4	3.4	97	740	9	COMPLEMENT C4 BETA CHAIN;
207	5fgb-E	6.4	3.6	100	213	9	ANTI-HCV E2 FAB HC84-1 LIGHT CHAIN;
208	4w2o-G	6.4	3.8	99	118	6	ANTI-MARBURGVIRUS NUCLEOPROTEIN SINGLE DOMAIN ANT
209	1ezv-X	6.4	3.7	102	127	10	UBIQUINOL-CYTOCHROME C REDUCTASE COMPLEX CORE
210	3kls-A	6.4	4.4	115	1622	10	COMPLEMENT C5;
211	3km9-A	6.4	4.4	117	1459	10	COMPLEMENT C5;
212	4e0s-A	6.4	3.8	113	1552	12	COMPLEMENT C5;
213	3pvm-A	6.4	4.1	116	1627	12	COMPLEMENT C5;
214	5hcd-B	6.4	4.4	117	647	10	COMPLEMENT C5;
215	5hce-B	6.4	4.4	117	647	10	COMPLEMENT C5;
216	4u48-A	6.3	3.9	107	1538	19	PUTATIVE INNER MEMBRANE LIPOPROTEIN;
217	5lgv-A	6.3	3.3	113	649	7	ALPHA-1,4-GLUCAN:MALTOSE-1-PHOSPHATE MALTOSYLTRAN
218	4yzf-E	6.3	3.5	99	223	8	BAND 3 ANION TRANSPORT PROTEIN;
219	5cvs-B	6.3	3.3	113	649	7	ALPHA-1,4-GLUCAN:MALTOSE-1-PHOSPHATE MALTOSYLTRAN
220	5a16-E	6.3	3.4	99	226	8	FAB4201 HEAVY CHAIN;
221	5a16-A	6.3	3.4	99	226	8	FAB4201 HEAVY CHAIN;
222	4yzf-K	6.3	3.9	106	223	8	BAND 3 ANION TRANSPORT PROTEIN;
223	5a16-G	6.3	3.3	99	226	8	FAB4201 HEAVY CHAIN;
224	5lgv-B	6.3	3.3	113	649	7	ALPHA-1,4-GLUCAN:MALTOSE-1-PHOSPHATE MALTOSYLTRAN
225	3mlp-A	6.3	2.6	107	337	12	TRANSCRIPTION FACTOR COE1;
226	4cn6-B	6.3	3.3	113	649	7	ALPHA-1,4-GLUCAN\:MALTOSE-1-PHOSPHATE MALTOSYLTRA
227	4cn6-A	6.3	3.3	113	649	7	ALPHA-1,4-GLUCAN\:MALTOSE-1-PHOSPHATE MALTOSYLTRA
228	1fgv-H	6.3	3.6	102	120	7	H52 FV (LIGHT CHAIN);
229	2or9-I	6.3	3.5	105	228	9	MONOCLONAL ANTI-C-MYC ANTIBODY 9E10;
230	4x7e-C	6.3	4	103	121	8	CAPSID PROTEIN;
231	4w2o-A	6.3	3.6	100	118	7	ANTI-MARBURGVIRUS NUCLEOPROTEIN SINGLE DOMAIN ANT
232	6i2g-A	6.3	3.7	103	121	9	ALFA NANOBODY;
233	3cu7-A	6.3	4.4	116	1625	12	COMPLEMENT C5;
234	4s0h-A	6.3	3.4	106	176	8	T-BOX TRANSCRIPTION FACTOR TBX5;
235	3km9-B	6.3	4.4	115	1459	10	COMPLEMENT C5;
236	4hjj-H	6.2	3.8	99	341	8	INTERLEUKIN-18;
237	1cwv-A	6.2	2.4	86	484	8	INVASIN;
238	6njs-A	6.2	3.7	120	523	9	SIGNAL TRANSDUCER AND ACTIVATOR OF TRANSCRIPTION
239	5d39-D	6.2	3.7	124	487	10	SIGNAL TRANSDUCER AND ACTIVATOR OF TRANSCRIPTION
240	5ow3-C	6.2	3	98	446	7	PROTEIN HAPLESS 2;
241	3qrg-H	6.2	3.7	100	220	10	FAB LIGHT CHAIN;
242	3ly6-C	6.2	3	92	683	7	PROTEIN-GLUTAMINE GAMMA-GLUTAMYLTRANSFERASE 2;
243	2xwj-C	6.2	3.7	103	640	9	COMPLEMENT C3 BETA CHAIN;
244	3mlo-A	6.2	2.8	110	212	13	TRANSCRIPTION FACTOR COE1;
245	3vrl-E	6.2	3.6	102	222	12	A10F9 FAB HEAVY CHAIN;
246	3rkd-D	6.2	3.4	101	218	7	CAPSID PROTEIN;
247	1ztx-H	6.2	3.8	102	219	8	ENVELOPE PROTEIN;
248	2co6-B	6.2	4.2	105	208	8	PUTATIVE OUTER MEMBRANE PROTEIN;
249	5dqj-H	6.2	3.6	105	222	9	S55-5 FAB (IGG1 KAPPA) LIGHT CHAIN;
250	2co7-B	6.2	3.8	100	197	9	SAFA PILUS SUBUNIT;
251	5dqj-A	6.2	3.5	104	222	9	S55-5 FAB (IGG1 KAPPA) LIGHT CHAIN;
252	3j42-I	6.2	3.6	103	216	11	ENVELOPE PROTEIN E;
253	4hd9-A	6.2	3.6	101	197	12	MUCOSAL ADDRESSIN CELL ADHESION MOLECULE 1;
254	4x7e-D	6.2	3.9	102	120	8	CAPSID PROTEIN;
255	4hcr-B	6.2	3.3	99	194	12	PF-547659 HEAVY CHAIN;

256	3so3-C	6.2	3.6	102	220	11	SUPPRESSOR OF TUMORIGENICITY 14 PROTEIN;
257	1t03-H	6.2	3.7	101	225	10	SYNTHETIC OLIGONUCLEOTIDE TEMPLATE;
258	4w2o-E	6.2	3.6	98	118	7	ANTI-MARBURGVIRUS NUCLEOPROTEIN SINGLE DOMAIN ANT
259	4p2c-J	6.2	4	105	127	8	SHIGA TOXIN 2E, SUBUNIT A;
260	1p53-A	6.2	3.2	91	250	12	INTERCELLULAR ADHESION MOLECULE-1;
261	3hs0-A	6.2	3.9	106	613	7	COBRA VENOM FACTOR;
262	1nc4-B	6.2	3.6	96	219	8	MONOCLONAL ANTIBODY 2D12.5, LAMBDA LIGHT CHAIN;
263	5jtw-A	6.2	4.3	112	651	11	COMPLEMENT C4-A;
264	3bpl-B	6.1	3.9	98	202	7	INTERLEUKIN-4;
265	1bqs-A	6.1	3.6	102	209	11	PROTEIN (MUCOSAL ADDRESSIN CELL ADHESION MOLECULE
266	4rtd-A	6.1	3.8	104	1122	16	UNCHARACTERIZED LIPOPROTEIN YFHM;
267	3cmg-A	6.1	6.2	87	661	3	PUTATIVE BETA-GALACTOSIDASE;
268	4g6k-H	6.1	3.7	104	220	8	HEAVY CHAIN OF GEVOKIZUMAB ANTIBODY BINDING
269	2gcy-B	6.1	3.7	102	218	9	HUC25 FAB FRAGMENT LIGHT CHAIN;
270	4yzf-I	6.1	3.6	98	223	8	BAND 3 ANION TRANSPORT PROTEIN;
271	3klh-D	6.1	3.7	100	225	11	REVERSE TRANSCRIPTASE/RIBONUCLEASE H;
272	3mln-B	6.1	2.7	106	213	12	TRANSCRIPTION FACTOR COE1;
273	4oi9-A	6.1	3.1	98	378	11	INTERCELLULAR ADHESION MOLECULE 5;
274	6ysq-A	6.1	4.5	99	651	9	COMPLEMENT C4 BETA CHAIN;
275	3mlp-B	6.1	2.8	108	332	12	TRANSCRIPTION FACTOR COE1;
276	4hix-H	6.1	3.8	103	219	8	HUMANIZED 3D6 FAB HEAVY CHAIN;
277	3mlp-E	6.1	2.7	108	303	12	TRANSCRIPTION FACTOR COE1;
278	4hzl-H	6.1	3.8	102	217	10	FAB HEAVY CHAIN;
279	3mlp-F	6.1	2.8	108	298	12	TRANSCRIPTION FACTOR COE1;
280	4uao-C	6.1	3.8	103	223	9	APICAL MEROZOITE ANTIGEN 1;
281	5dq9-C	6.1	3.6	102	217	9	S55-3 FAB (IGG2B) HEAVY CHAIN;
282	1z7z-I	6.1	3.1	97	434	11	HUMAN COXSACKIEVIRUS A21;
283	4r4b-B	6.1	3.8	104	220	11	FAB 2.2C LIGHT CHAIN;
284	3j42-K	6.1	3.7	104	216	11	ENVELOPE PROTEIN E;
285	1ad9-H	6.1	4	97	219	5	IGG CTM01 FAB (LIGHT CHAIN);
286	3p0g-B	6.1	3.8	102	121	6	BETA-2 ADRENERGIC RECEPTOR, LYSOZYME;
287	4cdg-D	6.1	4	101	122	7	BLOOM SYNDROME PROTEIN;
288	4zff-A	6.1	3.5	101	206	8	FRAGMENT ANTIGEN BINDING (FAB) 5A12 HEAVY CHAIN;
289	3tnm-H	6.1	3.7	100	221	10	FAB HEAVY CHAIN OF HUMAN ANTI-HIV-1 ENV ANTIBODY
290	4u6v-H	6.1	3.7	101	218	9	FAB, ANTIGEN BINDING FRAGMENT, HEAVY CHAIN;
291	6h7k-D	6.1	3.5	93	120	5	THIOREDOXIN 1;
292	1p53-B	6.1	3	91	250	11	INTERCELLULAR ADHESION MOLECULE-1;
293	1gig-H	6.1	3.7	101	221	9	IGG1-KAPPA HC19 FAB (LIGHT CHAIN);
294	5o04-D	6.1	3.9	101	119	7	CAPSID PROTEIN;
295	3inu-H	6.1	3.6	104	226	11	KZ52 ANTIBODY FRAGMENT HEAVY CHAIN;
296	4oga-C	6.1	3.6	102	118	9	INSULIN A CHAIN;
297	2fjf-H	6.1	3.7	101	218	8	LIGHT CHAIN OF A VEGF BINDING ANTIBODY;
298	4zff-H	6.1	3.5	101	211	8	FRAGMENT ANTIGEN BINDING (FAB) 5A12 HEAVY CHAIN;
299	6apq-A	6.1	3.2	96	119	7	ANTI-MARBURGVIRUS NUCLEOPROTEIN SINGLE DOMAIN ANT
300	4ldl-B	6.1	3.8	97	120	7	LYSOZYME, BETA-2 ADRENERGIC RECEPTOR;
