# Supplementary material for: Phylogenomics analysis of velvet regulators in the fungal kingdom
Source: Microbiol Spectr. 2024 Jan 5;12(2):e03717-23. doi: 10.1128/spectrum.03717-23 (PMC10845976; doi:10.1128/spectrum.03717-23)
Supplement: Table S3 — Similar structures of VosA velvet domain in the 3D macromolecular structures database of NCBI by VAST+. [file spectrum.03717-23-s0004.pdf]

Table S3. Similar structures of VosA velvet domain in the 3D macromolecular structures database of NCBI by VAST+

	PDB ID	Description	Taxonomy	RMSD	Aligned residues	Sequence identity
1	3QRF	Structure of a domain-swapped FOXP3 dimer	Homo sapiens	3.20Å	120	18%
2	1A02	STRUCTURE OF THE DNA BINDING DOMAINS OF NFAT, FOS AND JUN BOUND TO DNA	Others	3.24Å	117	18%
3	1PZU	An Asymmetric Nfat1-Rhr Homodimer On A Pseudo-Palindromic, Kappa-B Site	Others	2.42Å	110	19%
4	2V2T	X-Ray Structure Of A Nf-Kb P50-Relb-Dna Complex	Others	3.21Å	106	14%
5	1S9K	Crystal Structure Of Human Nfat1 And Fos-Jun On The Il-2 Arre1 Site	Others	2.38Å	103	19%
6	1LE9	Crystal structure of a NF-kB heterodimer bound to the Ig/HIV-kB siti	Others	2.53Å	100	18%
7	1GJI	Crystal Structure Of C-Rel Bound To Dna	Others	2.69Å	99	16%
8	2I9T	Structure of NF-kB p65-p50 heterodimer bound to PRDII element of B-interferon promoter	Mus musculus	2.73Å	98	19%
9	1LE5	Crystal structure of a NF-kB heterodimer bound to an IFNb-kB	Others	2.15Å	97	19%
10	2O6D	Structure of native rTp34 from Treponema pallidum	Treponema pallidum	3.42Å	97	10%
11	6MBZ	Structure of Transcription Factor	Homo sapiens	2.40Å	96	14%
12	3PJL	The Crystal Structure Of Tp34 Bound To Co (Ii) Ion At Ph 7.5	Treponema pallidum	3.39Å	96	10%
13	1LEI	The kB DNA sequence from the HLV-LTR functions as an allosteric regulator of HIV transcription	Others	1.99Å	95	19%
14	3DO7	X-Ray Structure Of A Nf-Kb P52RELBDNA COMPLEX	Others	2.00Å	95	21%
15	1VKX	Crystal Structure Of The Nfkb P50P65 HETERODIMER COMPLEXED To The Immunoglobulin Kb Dna	Others	2.26Å	95	20%
16	1IKN	IkappabalphaNF-Kappab Complex	Homo sapiens/Mus musculus	2.70Å	94	16%
17	6MBW	Structure of Transcription Factor	Homo sapiens	2.21Å	93	13%
18	5ZHU	Crystal structure of the DNA-binding domain of human myelin-gene regulatory factor	Homo sapiens	2.57Å	93	15%
19	2O6F	Structure of metal- free rTp34 from Treponema pallidum	Treponema pallidum	3.29Å	93	11%
20	1BVO	DORSAL HOMOLOGUE GAMBIF1 BOUND TO DNA	Anopheles gambiae	2.03Å	92	16%
21	1NFK	STRUCTURE OF THE NUCLEAR FACTOR KAPPA-B (NF-KB) P50 HOMODIMER	Mus musculus	2.10Å	92	20%
22	4Y5W	Transcription Factor-dna Complex	Homo sapiens	2.11Å	92	11%
23	1EAO	The Runx1 Runt Domain At 1.4a Resolution: A Structural Switch And Specifically Bound Chloride Ions Modulate Dna Binding	Mus musculus	2.19Å	92	11%
24	3LZR	Crystal Structure Analysis Of Manganese Treated P19 Protein From Campylobacter Jejuni At 2.73 A At Ph 9 And Manganese Peak Wavelength (1.893 A)	Campylobacter jejuni subsp. jejuni 81-176	3.75Å	92	12%
25	5D39	Transcription Factor-dna Complex	Homo sapiens	2.12Å	91	11%
26	5U01	Cooperative DNA binding by two RelA dimers	Mus musculus/Synthesium	2.25Å	91	16%
27	1E50	AML1/CBFbeta complex	Homo sapiens	2.31Å	91	10%
28	6HQC	Structural investigation of the TasA anchoring protein TapA from Bacillus subtilis	Bacillus subtilis	2.94Å	91	3%
29	2XTJ	The crystal structure of PCSK9 in complex with 1D05 Fab	Homo sapiens	3.11Å	91	8%
30	5TVZ	Solution NMR structure of Saccharomyces cerevisiae Pom152 Ig-like repeat, residues 718-820	Saccharomyces cerevisiae	3.90Å	91	10%
31	1A3Q	Human Nf-Kappa-B P52 Bound To Dna	Others	1.77Å	90	22%
32	3GUT	Crystal structure of a higher-order complex of p50:RelA bound to the HIV-1 LTR	Others	2.20Å	90	17%
33	6GGR	Crystal structure of Salmonella zinc metalloprotease effector GtgA in complex with p65	Others	2.26Å	90	17%
34	1HJC	CRYSTAL STRUCTURE OF RUNX-1/AML1/CBFALPHA RUNT DOMAIN BOUND TO A DNA FRAGMENT FROM THE CSF-1R PROMOTER	Homo sapiens/Mus musculus	2.29Å	90	10%
35	1IO4	Crystal Structure Of Runx-1AML1CBFALPHA RUNT DOMAIN- Cbfbeta Core Domain Heterodimer And CEBPBETA BZIP Homodimer Bound To A Dna Fragment From The Csf-1r Promoter	Others	2.31Å	90	10%
36	1HJB	CRYSTAL STRUCTURE OF RUNX-1/AML1/CBFALPHA RUNT DOMAIN AND C/EBPBETA BZIP HOMODIMER BOUND TO A DNA FRAGMENT FROM THE CSF-1R PROMOTER	Homo sapiens/Mus musculus	2.33Å	90	10%

37	1LJM	Dna Recognition Is Mediated By Conformational Transition And By Dna Bending	Homo sapiens	2.36Å	90	9%
38	3WTT	Crystal structure of the complex comprised of phosphorylated ETS1, RUNX1, CBFBETA, and the tcralpha gene enhancer DNA	Others	2.36Å	90	10%
39	3WTY	Crystal structure of the complex comprised of ETS1(G333P), RUNX1, CBFBETA, and the tcralpha gene enhancer DNA	Others	2.37Å	90	10%
40	3WTS	Crystal structure of the complex comprised of ETS1, RUNX1, CBFBETA, and the tcralpha gene enhancer DNA	Others	2.38Å	90	10%
41	6F58	Crystal structure of human Brachyury (T) in complex with DNA	Homo sapiens	2.57Å	90	6%
42	2RAM	A Novel Dna Recognition Mode By Nf-Kb P65 Homodimer	Others	2.04Å	89	17%
43	1EAQ	The Runx1 Runt Domain At 1.25a Resolution: A Structural Switch And Specifically Bound Chloride Ions Modulate Dna Binding	Mus musculus	2.07Å	89	10%
44	2J6W	R164n Mutant Of The Runx1 Runt Domain	Mus musculus	2.14Å	89	10%
45	4L18	Crystal Structure Of Runx1 And Ets1 Bound To Tcr Alpha Promoter (crystal Form 3)	Homo sapiens/Mus musculus	2.19Å	89	9%
46	4L0Y	Crystal Structure Of Runx1 And Ets1 Bound To Tcr Alpha Promoter (crystal Form 1)	Homo sapiens/Mus musculus	2.20Å	89	10%
47	3WTU	Crystal structure of the complex comprised of ETS1 (V170A), RUNX1, CBFBETA, and the tcralpha gene enhancer DNA	Others	2.23Å	89	10%
48	3WTX	Crystal structure of the complex comprised of ETS1(Y329A), RUNX1, CBFBETA, and the tcralpha gene enhancer DNA	Others	2.25Å	89	10%
49	4L0Z	Crystal Structure Of Runx1 And Ets1 Bound To Tcr Alpha Promoter (crystal Form 2)	Homo sapiens/Mus musculus	2.31Å	89	10%
50	1H9D	Aml1/cbf-beta/dna complex	Homo sapiens	2.32Å	89	10%
51	6F59	Crystal structure of human Brachyury (T) G177D variant in complex with DNA	Homo sapiens	2.42Å	89	7%
52	3WU1	Crystal structure of the ETS1-RUNX1-DNA ternary complex	Others	2.17Å	88	10%
53	3WTV	Crystal structure of the complex comprised of ETS1(V170G), RUNX1, CBFBETA, and the tcralpha gene enhancer DNA	Others	2.35Å	88	10%
54	3E9U	Crystal Structure Of Calx Cbd2 Domain	Drosophila melanogaster	4.30Å	88	2%
55	1YVL	Structure Of Unphosphorylated Stat1	Others	1.56Å	87	13%
56	4Y5U	Transcription Factor	Homo sapiens	1.92Å	87	11%
57	1EAN	The Runx1 Runt Domain At 1.70a Resolution: A Structural Switch And Specifically Bound Chloride Ions Modulate Dna Binding	Mus musculus	2.17Å	86	9%
58	5QSA	PanDDA analysis group deposition -- Crystal Structure of human Brachyury G177D variant in complex with Z2856434778	Homo sapiens	2.22Å	86	7%
59	3WTW	Crystal structure of the complex comprised of ETS1(K167A), RUNX1, CBFBETA, and the tcralpha gene enhancer DNA	Others	2.22Å	86	10%
60	2JHS	Crystal Structure Of Rhogdi K135h,K138h,K141h Mutant	Homo sapiens	3.26Å	86	9%
61	2X6V	Crystal Structure Of Human Tbx5 In The Dna-Bound And Dna- Free Form	Others	2.07Å	85	9%
62	3PJN	The Crystal Structure Of Tp34 Bound To Zn(Ii) Ion At Ph 7.5	Treponema pallidum	2.80Å	85	12%
63	2O6E	Structure of native rTp34 from Treponema pallidum from zinc-soaked crystals	Treponema pallidum	2.82Å	85	12%
64	2OZN	The Cohesin-Dockerin Complex Of Nagj And Nagh From Clostridium Perfringens	Clostridium perfringens/Clostridium perfringens ATCC 13124	3.04Å	85	12%
65	1Z8Y	Mapping the E2 Glycoprotein of Alphaviruses	Sindbis virus	3.45Å	84	8%
66	5FLV	Crystal Structure Of Nkx2-5 And Tbx5 Bound To The Nppa Promoter Region	Mus musculus	2.16Å	81	9%
67	6D4A	Cell Surface Receptor with Bound Ligand at 1.75-A Resolution	Homo sapiens	3.10Å	81	6%
68	6TLC	Unphosphorylated human STAT3 in complex with MS3-6 monobody	Homo sapiens	1.36Å	80	13%

69	2LT9	The solution structure of Ca2+ binding domain 2B of the third isoform of the Na+/Ca2+ exchanger	Mus musculus	3.92Å	80	4%
70	3MQI	Human Early B-Cell Factor 1 (Ebf1) IptTIG DOMAIN	Homo sapiens	3.08Å	79	9%
71	6D48	Cell Surface Receptor	Homo sapiens	3.19Å	79	6%
72	4NLH	Skich Domain Of Human Tax1bp1	Homo sapiens	2.54Å	78	9%
73	4HUM	Mate Transporter Norm-ng In Complex With Ethidium And Monobody	Escherichia coli/Neisseria gonorrhoeae TCDC-NG08107	2.96Å	78	5%
74	6P1E	Cu-bound PmoF1 PCuAC domain (dimer)	Methylocystis sp. ATCC 49242	3.01Å	78	8%
75	5Z7G	Crystal structure of TAX1BP1 SKICH region in complex with NAP1	Homo sapiens	2.53Å	77	9%
76	5Z7A	Crystal structure of NDP52 SKICH region	Homo sapiens	2.58Å	77	5%
77	3LZN	Crystal Structure Analysis Of The Apo P19 Protein From Campylobacter Jejuni At 1.59 A At Ph 9	Campylobacter jejuni subsp. jejuni 81-176	3.07Å	77	5%
78	3K2M	Crystal Structure of Monobody HA4/Abl1 SH2 Domain Complex	Homo sapiens	3.13Å	76	3%
79	2NMS	The Crystal Structure Of The Extracellular Domain Of The Inhibitor Receptor Expressed On Myeloid Cells Irem-1	Homo sapiens	2.60Å	75	4%
80	3IDU	Crystal Structure of the CARDB domain of the PF1109 protein in complex with di-metal ions from Pyrococcus furiosus, Northeast Structural Genomics Consortium Target PfR193A	Pyrococcus furiosus	2.68Å	73	11%
81	5I0V	Iron And Copper-bound P19 From Campylobacter Jejuni Under Oxidizing Conditions	Campylobacter jejuni subsp. jejuni 81-176	2.79Å	72	4%
82	4U6T	Crystal structure of the Clostridium histolyticum colH collagenase polycystic kidney disease-like domain 2a at 1.76 Angstrom resolution	Hathewaya histolytica	2.60Å	71	3%
83	4HUN	MATE transporter NorM-NG in complex with R6G and monobody	Escherichia coli/Neisseria gonorrhoeae TCDC-NG08107	2.75Å	71	6%
84	2AW2	Crystal Structure Of The Human Btla-hvem Complex	Homo sapiens	2.79Å	70	14%
85	3VVV	Skich Domain Of Ndp52	Homo sapiens	2.35Å	69	4%
86	6VHI	Crystal structure of the human ILRUN Fw domain	Homo sapiens	2.52Å	69	6%
87	1U36	Crystal structure of WLAC mutant of dimerisation domain of NF-kB p50 transcription factor	Mus musculus	2.53Å	69	14%
88	3TEU	Crystal structure of fibcon	Others	2.53Å	69	7%
89	1U3Y	Crystal structure of ILAC mutant of dimerisation domain of NF-kB p50 transcription factor	Mus musculus	2.57Å	69	14%
90	1U41	Crystal structure of YLGV mutant of dimerisation domain of NF-kB p50 transcription factor	Mus musculus	2.58Å	69	14%
91	2Y72	Crystal Structure Of The Pkd Domain Of Collagenase G From Clostridium Histolyticum At 1.18 Angstrom Resolution	Hathewaya histolytica	2.91Å	69	10%
92	2R39	Crystal Structure Of Fixg-Related Protein From Vibrio Parahaemolyticus	Vibrio parahaemolyticus RIMD 2210633	2.37Å	68	9%
93	4JRW	Crystal structure of Clostridium histolyticum colg collagenase PKD domain 2 at 1.6 Angstrom resolution	Hathewaya histolytica	2.71Å	68	9%
94	4AQO	Crystal Structure Of The Calcium Bound Pkd-like Domain Of Collagenase G From Clostridium Histolyticum At 0.99 Angstrom Resolution	Hathewaya histolytica	2.87Å	68	9%
95	4MMX	Integrin AlphaVBeta3 ectodomain bound to the tenth domain of Fibronectin	Homo sapiens	2.87Å	68	6%
96	3LZQ	Crystal Structure Analysis Of Manganese Treated P19 Protein From Campylobacter Jejuni At 1.41 A At Ph 9	Campylobacter jejuni subsp. jejuni 81-176	2.50Å	67	12%
97	5L74	Plexin A2 extracellular segment domains 4-5 (PSI2-IPT2), resolution 1.36 Angstrom	Mus musculus	2.63Å	67	7%
98	1U3J	Crystal structure of MLAV mutant of dimerisation domain of NF-kB p50 transcription factor	Mus musculus	2.65Å	67	12%
99	6NYP	Crystal structure of UL144/BTLA complex	Others	2.73Å	67	13%
100	4U7K	Crystal structure of the Clostridium histolyticum colH collagenase polycystic kidney disease-like domain 2a in the presence of calcium at 1.9 Angstrom resolution	Hathewaya histolytica	2.90Å	67	3%

101	2LFE	Solution NMR structure of N-terminal domain of human E3 ubiquitin-protein ligase HECW2, Northeast structural genomics consortium (NESG) target ht6306A	Homo sapiens	2.59Å	66	8%
102	4TN9	Crystal structure of Clostridium histolyticum ColG collagenase polycystic kidney disease-like domain at 1.4 Angstrom resolution	Hathewaya histolytica	2.71Å	66	9%
103	4HJI	Structure Of The Cooa Pilin Subunit From Enterotoxigenic Escherichia Coli	Escherichia coli	3.19Å	66	8%
104	5FM8	Structure of the C-terminally extended domain My4 of human myomesin (space group P65)	Homo sapiens	2.27Å	65	11%
105	4N68	Crystal Structure Of An Internal Fn3 Domain From Human Contactin-5 [psi-nysgrc-005804]	Homo sapiens	2.50Å	65	6%
106	5Z7L	Crystal structure of NDP52 SKICH region in complex with NAP1	Homo sapiens	2.58Å	65	8%
107	2W0P	Crystal Structure Of The Filamin A Repeat 21 Complexed With The Migfilin Peptide	Homo sapiens	2.67Å	64	13%
108	2JJT	Structure of human CD47 in complex with human signal regulatory protein (SIRP) alpha	Homo sapiens	2.97Å	64	11%
109	6W8X	Cryo-EM of the S. solfataricus pilus	Saccharolobus solfataricus	4.08Å	64	11%
110	5IUS	Crystal Structure Of Human Pd-l1 In Complex With High Affinity Pd-1 Mutant	Homo sapiens	2.74Å	63	5%
111	1I8L	Human B7-1CTLA-4 Co-Stimulatory Complex	Homo sapiens	2.42Å	62	8%
112	2CD0	Structure Of Human Lambda-6 Light Chain Dimer Wil	Homo sapiens	2.93Å	62	6%
113	6C6Q	Crystal Structure of the Murine Norovirus VP1 P Domain in complex with the CD300lf Receptor	Others	2.45Å	61	2%
114	5OC7	Crystal structure of the pleckstrin-homology domain of Bcr-Abl in complex with monobody Mb(Bcr-PH_4)	Others	2.15Å	60	7%
115	2MOG	Solution structure of the terminal Ig-like domain from Leptospira interrogans LigB	Leptospira interrogans	2.41Å	60	8%
116	2HAZ	Crystal Structure Of The First Fibronectin Domain Of Human Ncam1	Homo sapiens	2.47Å	60	3%
117	3RNK	Crystal structure of the complex between mouse PD-1 mutant and PD-L2 IgV domain	Mus musculus	2.48Å	58	9%
118	6HIG	hPD-1/NBO1a Fab complex	Homo sapiens/Mus musculus	3.03Å	58	10%
119	2N1K	Structure of the Third Type III Domain from Human Fibronectin	Homo sapiens	1.92Å	56	13%
120	6C74	Crystal Structure of Murine CD300lf in complex with phosphocholine	Mus musculus	2.20Å	56	2%
121	7BYR	BD23-Fab in complex with the S ectodomain trimer	Others	2.27Å	55	4%
122	1AC6	Crystal Structure Of A Variable Domain Mutant Of A T-cell Receptor Alpha Chain	Mus musculus	2.41Å	55	11%
123	3B83	Computer-based Redesign Of A Beta Sandwich Protein Suggests That Extensive Negative Design Is Not Required For De Novo Beta Sheet Design	Homo sapiens	3.10Å	55	7%
124	4U3H	Crystal Structure Of Fn3con	Others	2.05Å	54	7%
125	5ECJ	Crystal structure of monobody Mb(S4) bound to Prdm14 in complex with Mtgr1	Homo sapiens/Mus musculus	1.73Å	53	11%
126	1CD0	Structure Of Human Lamda-6 Light Chain Dimer Jto	Homo sapiens	2.65Å	53	9%
127	6BYN	Crystal structure of WDR5-Mb(S4) monobody complex	Homo sapiens	1.88Å	52	8%
128	4JE4	Crystal Structure Of Monobody Nsa1/shp2 N-sh2 Domain Complex	Homo sapiens	2.11Å	51	12%
129	3SO5	Crystal structure of an Immunoglobulin I-set domain of Lrig3 protein (Lrig3) from MUS MUSCULUS at 1.70 A resolution	Mus musculus	1.97Å	50	12%
130	5FQ7	Crystal Structure Of The Suscd Complex Bt2261-2264 From Bacteroides Thetaiotaomicron	Bacteroides thetaiotaomicro n	2.25Å	50	12%
131	3RZW	Crystal Structure of the Monobody ySMB-9 bound to human SUMO1	Homo sapiens	1.92Å	49	10%
132	2MTP	The structure of Filamin repeat 21 bound to integrin	Homo sapiens	2.69Å	44	9%
